# Gene count from target sequence capture places three whole genome duplication events in *Hibiscus* L. (Malvaceae)

**DOI:** 10.1186/s12862-021-01751-7

**Published:** 2021-06-02

**Authors:** J. S. Eriksson, C. D. Bacon, D. J. Bennett, B. E. Pfeil, B. Oxelman, A. Antonelli

**Affiliations:** 1 School of Bioscience, Systems Biology Research Center, 541 45 Skövde, Sweden; 2 Department of Biological and Environmental Sciences, University of Gothenburg, Box 461, 405 30 Gothenburg, Sweden; 3 Gothenburg Global Biodiversity Centre, Box 461, 405 30 Gothenburg, Sweden; 4 Royal Botanic Gardens, Kew, Richmond, Surrey, TW9 3AE UK; 5 Department of Plant Sciences, University of Oxford, South Parks Road, Oxford, OX1 3 RB UK

**Keywords:** Ancient genome duplication, Gene copy, Haplotype, *Hibiscus*, Malvaceae, Paralogy, Polyploidy

## Abstract

**Background:**

The great diversity in plant genome size and chromosome number is partly due to polyploidization (i.e. genome doubling events). The differences in genome size and chromosome number among diploid plant species can be a window into the intriguing phenomenon of past genome doubling that may be obscured through time by the process of diploidization. The genus *Hibiscus* L. (Malvaceae) has a wide diversity of chromosome numbers and a complex genomic history. *Hibiscus* is ideal for exploring past genomic events because although two ancient genome duplication events have been identified, more are likely to be found due to its diversity of chromosome numbers. To reappraise the history of whole-genome duplication events in *Hibiscus*, we tested three alternative scenarios describing different polyploidization events.

**Results:**

Using target sequence capture, we designed a new probe set for *Hibiscus* and generated 87 orthologous genes from four diploid species. We detected paralogues in > 54% putative single-copy genes. 34 of these genes were selected for testing three different genome duplication scenarios using gene counting. All species of *Hibiscus* sampled shared one genome duplication with *H. syriacus*, and one whole genome duplication occurred along the branch leading to *H. syriacus*.

**Conclusions:**

Here, we corroborated the independent genome doubling previously found in the lineage leading to *H. syriacus* and a shared genome doubling of this lineage and the remainder of *Hibiscus*. Additionally, we found a previously undiscovered genome duplication shared by the /Pavonia and /Malvaviscus clades (both nested within *Hibiscus*) with the occurrences of two copies in what were otherwise single-copy genes. Our results highlight the complexity of genomic diversity in some plant groups, which makes orthology assessment and accurate phylogenomic inference difficult.

## Background

Whole-genome duplication (WGD), defined as the doubling of an entire genome [23], is a well-known phenomenon in eukaryotes and is especially prevalent in plants [19,30,43, 55, 57, 58, 68]. Genomic studies in plants have demonstrated multiple WGD events throughout angiosperm evolution [9, 15, 22, 32, 56, 60, 63, 69] and c. 15% of all angiosperm speciation events are considered to be of polyploid origin [74]. Polyploidy causes a great diversity in genome size and chromosome numbers, which can vary considerably even within families and genera [45, 67]. With the increased availability of high-throughput DNA sequence data, recently formed polyploid species that arose from extant progenitor lineages have received more attention in phylogenetic studies [5, 8]. The vast amount of emerging genetic data, however, opens up potential insight into ancient polyploidization.

The challenge of detecting ancient WGD can mainly be explained by diploidization, where polyploid genomes undergo genomic restructuring leading towards a diploid-like state [4, 45, 56, 68]. While some loci are retained as singletons and others as duplicates, diploidization does not return the polyploid to its original diploid state [56]. Examples of mechanisms behind this are gene loss and chromosomal rearrangement [52]. Moreover, mutations leading to shifts in gene expression, such as neofunctionalization and subfunctionalization, will also render the diploidized polyploid unique. Diploidization can also result from entire chromosomes being lost (aneuploidy), where synthetic polyploids have been demonstrated to suffer from an elevated chromosomal instability after genome duplication [56]. Apart from diploidization, fractionation can result in losses of entire chromosomes and copies of gene pairs duplicated through polyploidy (homoeologs). These can occur randomly with respect to either parental genome, but, in some cases, losses predominantly occur in one of the parental genomes [51, 56, 61, 75]. In a phylogenetic context, gene losses can mislead species tree inference, due to mistaken orthology. Repeated cycles of polyploid formation followed by genome rearrangement [56, 69] and fractionation hinder the recognition of ancient WGD [79].

Commonly used methods to place WGD events on a phylogeny include synteny blocks, K_s_-rates and/or phylogenetic approaches. These approaches are powerful but are limited by: a priori information from whole-genome or transcriptome sequencing [49, 78], saturation effects in Ks-based methods which cannot detect ancient WGD events [66], and phylogenetic approaches that require fully bifurcating, single-labeled trees for representing the species relationships [49]. Polyploids are best represented as a species network or a multi-labeled tree (MUL-trees) where a species can occur at multiple tips [20], representing the homoeologues or subgenomes.

Alternative WGD detection approaches are gene count methods, which require a species tree where different hypotheses can be made as to where a WGD event occurred (either along a branch or at a node), together with data on how many copies a species has in different genes. The basic assumption is that WGD events should result in species with extra gene copies/alleles than species not affected by WGD. It should be noted that this approach does not deal with the underlying process leading to genome duplication (i.e. auto- or allopolyploidization). In addition, copies that are not linked to WGD but instead arise from single gene duplications are included in this approach, with rates of birth and loss of copies parameterized. Target sequence capture together with gene counting methods can complement K_s_-rates, synteny and gene tree mapping-based methods that rely heavily on genome and transcriptome data.

A high diversity of recent ploidy levels and a wide range of haploid chromosome numbers in diploids suggest that several rounds of WGD have shaped the genomic history of Malvaceae s.l. subfamily Malvoideae [1, 2, 17, 40, 41, 47]. For example, in cottons, *Gossypium* L.*,* multiple instances of genome duplication have been inferred, indicating that diploid cottons are paleopolyploids [69].This hypothesis was first suggested in the early twentieth century through studies of chromosome pairing during meiosis [12, 33] and supported by recent DNA sequencing [30, 69]. The haploid chromosome number of *x* = 13 is understood to be derived from seven chromosome pairs in an ancestral cotton, which may be as old as 20–40 million years [11, 33, 53, 69]. Regardless, the paleopolyploidization has been inferred to predate the origin of Malvaceae [69]. Further, two additional ancient genome duplications were found in the genome history of cotton [65]. One of the duplication events took place within the lineage *Gossypium* itself, while the other duplication event supports the evidence of a whole-genome triplication (at least two WGDs in short succession; [23]) shared by all eudicots [65].

*Hibiscus* L. is a widely cultivated genus of Malvaceae, characterized by its numerous rounds of polyploidy [30, 47, 72]. The taxonomic delimitation of *Hibiscus* has been unstable ([48] and references therein) with nuclear and chloroplast genes suggesting the traditional circumscription is a paraphyletic group. Phylogenetic work showed that traditionally defined *Hibiscus* includes representatives of other genera that had been classified in the tribes Hibisceae, Malvavisceae (including e.g., *Pavonia*) and Decaschistieae [46]. Pfeil and Crisp [48] proposed to treat the three tribes under *Hibiscus* s.l., which we apply here. Within this classification, unranked clade names preceeded by a forward slash (/) are used to indicate clades nested within *Hibiscus* sensu [48]. Note that not all combinations at the species level have been made in that classification, so we use existing binomials in other genera as necessary.

The diversity of haploid chromosome numbers in *Hibiscus* may reflect ancient genome doubling events followed by diploidization. A group of species within *Hibiscus,* clade /Furcaria*,* is a well-studied group of polyploids [72, 73]. Menzel [39] proposed that the diploid *Hibiscus cannabinus* L. in /Furcaria*,* with a haploid chromosome number of *x* = 18, may have been derived through ancient WGD events with a base chromosome number of either six or nine. *Hibiscus* section /Euhibiscus has a base chromosome number of *x* = 20–22 (e.g. *H. rosa-sinensis* and *H. syriacus* [54]). In addition, the mostly Neotropical clade /Pavonia is hypothesized to originate from either *x* = 7 (shown from a series of seven chromosomes; [54]) or *x* = 14 (suggested from the lowest chromosome count) based on the multiples of chromosome counts inferred by several species ([17], treated under *Pavonia*). Only ~ 29 of c. 220 species of *Pavonia* have been counted ([17] and references therein, [11, 16]). Of these, two are *2n* = 28, 23 are *2n* = 56, and two are *2n* = 112, indicating that many of the species are likely to be higher polyploids.

Two ancient genome doubling events followed by diploidization were identified in the *H. syriacus* L. lineage by constructing synteny and collinearity blocks from genomic data (clade /Euhibiscus; [30]). The two WGD events are considered to be independent and took place after the divergence from the *H. syriacus-G. raimondii* common ancestor [30]. The varying haploid chromosome numbers within *Hibiscus* and between the sister genus *Gossypium*, may reflect varying degrees of diploidization, with chromosome fusion/fission in different lineages after speciation. Whether diploidization is the underlying cause for the diverse base chromosome number found in species of *Hibiscus* is yet to be understood.

**Fig. 1:**
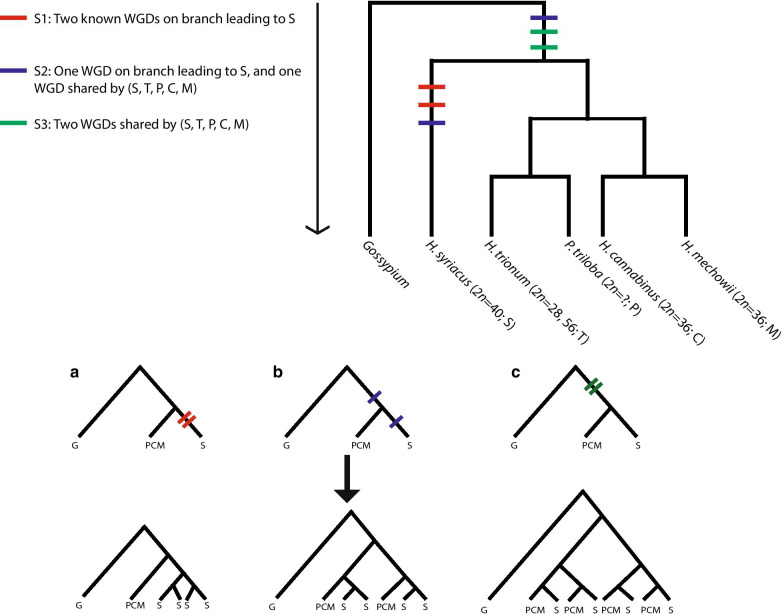
Three hypothetical genome evolution scenarios in *Hibiscus* with *Gossypium raimondii* (G) as outgroup. S is short for for *H. syriacus*; T for *H. trionum*; P for *Pavonia triloba*; C for *H. cannabinus*; and M for *H. mechowii*. The different colors represent three different genome duplication scenarios, **a** where red = S1 (two independent WGD in S), **b** blue = S2 (one WGD shared by all species of *Hibiscus* and one independent in S) and **c** green = S3 (two WGD events shared by all species of *Hibiscus*). Numbers above branches leading to each species are known chromosome counts

In this study, we determine if diploid and polyploid species of *Hibiscus* have signatures of ancient genome duplications, and if these are shared with the WGDs found in *H. syriacus*. Based on previous phylogenetic hypotheses [3, 46, 47], and base chromosome number variation between clades in *Hibiscus*, we present three hypothetical scenarios (Fig. 1; scenario S1–S3) that illustrate the likely genomic origins of *Hibiscus* before diploidization using the two WGD events detected previously in *H. syriacus* (Fig. 1). To test amongst these hypotheses, we use diploid members of *Hibiscus* clade /Furcaria that are assumed to be derived from an ancient genome duplication [40]. We furthermore select a species from /Pavonia*,* given the lack of diploids in this group, their relatively high chromosome numbers (*2n* = 56–112; [17] and references therein) and the unknown base chromosome number. In the first scenario, only *H. syriacus* shows evidence of two WGDs (S1; Fig. 1a). However, considering that the base chromosome numbers vary greatly within *Hibiscus* (e.g. *Hibiscus* section *Trionum x* = 7 or 14 [17], section *Furcaria x* = 18 [54] and /Euhibiscus *x* = 20–22 (e.g. *H. rosa-sinensis* and *H. syriacus*, [54])—the WGD events leading to *H. syriacus* (S1) may involve other species of *Hibiscus*. In the second scenario, we explore if one of the WGD events in *H. syriacus* is shared by all species of *Hibiscus*, and if the second duplication is restricted to *H. syriacus* (S2; Fig. 1b). In the third scenario, we test whether both WGD events in *H. syriacus* are shared by all species of *Hibiscus* (S3; Fig. 1c).

Here, we develop a new analytical framework to identify multiple haplotypes and assemble them into full sequences. Current methods use different approaches to overcome the challenge of connecting alleles/homoeologues/haplotypes by using ambiguity codes where the read depth is too shallow to connect two variants (Kates et al. [24]), or by using a known pedigree [7], Martin et al. [38]). Alternative approaches use a reference genome or construct a de novo reference from read data. However, these algorithms are built on the assumptions that all organisms are diploids and that only two haplotypes exist at a locus. In the presence of more than two haplotypes, such as in polyploid plants, either chimeric haplotypes are produced or the number of haplotypes is underestimated. Our approach makes no assumption regarding the number of sequence copies or ploidy level and does not construct chimeric sequences as a result of more than two copies found in polyploid species.

## Results

### Target capture, mapping and paralogue assembly

A new sequence probe set was designed for *Hibiscus,* spanning 87 orthologous genes (Additional file 1). The mean percentage recovered on target loci was 99.2% (Additional file 2). Data from six individuals were successfully sequenced with a mean number of 1,261,242 reads per individual after trimming (Additional file 2). Out of 87 genes targeted, 14 genes had one sequence copy per species (referred here as SCG) and 20 genes showed more than one sequence copy per species (reffered here as MCG). All genes had contigs that were overlapping for the same region for all species. The mean read depth (coverage) of each assembly ranged between 81 and 413 (Table 1). The final alignments had a mean length of 1972 bp (ranging between 934 and 3151 bp).

**Table 1:** Mean read depth across all base pairs per species per gene

	Mean read depth (coverage across all assemblies) per locus					
Gene	*H. cannabinus*1	*H. cannabinus*2	*H. cannabinus*3	*H. mechowii*	*H. trionum*	*P. triloba*
ABC-C2	206	120	180	324	66	53
ACCS	231	179	172	284	42	38
Acylamino	480	300	330	823	122	76
AglucanP	411	638	597	399	171	112
Ankyrin	239	151	145	315	91	77
Bgalactosidase8	256	193	178	322	57	70
CAD	406	295	270	381	60	57
Calcium11	494	293	315	600	75	158
Calcium-ATPase1	215	195	196	287	73	72
Calcium-ATPase3*	338	217	243	460	87	76
Callose*	93	84	222	91	42	45
CesA1	200	209	147	255	79	116
DEAD-ATP*	324	308	284	150	87	90
EIF-2B	133	96	98	160	23	35
F5H	232	200	172	268	66	52
Formin2	141	117	109	151	31	27
Glutamine	425	302	349	744	158	85
GPDH	297	264	258	459	136	100
Importin4*	413	271	328	538	55	109
Kinesin-KCA2*	268	233	276	291	76	87
Kinesin-Kp1	333	410	398	473	106	44
LOC105792102	187	153	137	221	58	58
MAP3K	385	280	274	441	102	78
Mechanosensitive*	481	467	481	622	195	143
MNS4*	299	276	220	353	91	83
NF-X1-zinc*	274	282	271	429	100	85
Oxysterol-1D*	1103	1224	1095	2002	323	316
Phospholipase	212	178	178	299	62	65
Plasma-ATPase	216	189	174	238	73	56
Polysub2*	428	562	220	798	170	161
RRP5*	404	307	311	410	103	114
SBT3-5*	591	467	451	−	136	102
SD1-1*	628	495	524	685	127	116
TGH*	743	560	570	405	172	162
Mean coverage across all loci	312	261	259	371	87	80

The average read coverage is calculated across all contigs and per exons for each gene. Asterisks (*) refer to genes that have only one sequence copy per species (e.g. single copy genes)

### Occurrence of paralogous genes

Despite targeting low-copy nuclear genes (from transcriptome at hand; *Hibiscus cannabinus,* 1 KP Code OLXF), we found that 54% of the genes contained more than the two variants (i.e. haplotypes) found in one of the diploid *H. cannabinus* accessions (i.e. *H. cannabinus*1). The GPDH gene had ten different DNA sequence variants in *H. cannabinus*1 (the individual sequenced in this study), but only a single variant was found in the *H. cannabinus* transcriptome. However, this gene appeared at eight locations in the *G. raimondii* genome. The glutamine gene (LOC 105 766 149), with three *H. cannabinus*1 variants, was only found as a single contig in the transcriptome and also appeared as a single copy in the *G. raimondii* genome. We consistently observed subtrees that had either one or two or more sequence copies from *H. cannabinus* (Table 2). *Hibiscus syriacus* was often seen to have more than three copies in each subtree, whereas the /Pavonia clade species nearly always had twice as many copies as seen in *H. cannabinus*.

**Table 2:** Gene count data used for likelihood scenario testing

	Number of copies					
Gene name	G	S	C	M	T	P
ABC-c2	1	2	1	1	0	0
ABC-c2	1	1	1	0	1	3
ACCS	1	0	2	1	0	2
ACCS	1	3	2	2	2	1
Acylamino	1	3	3	2	2	5
AglucanP	1	9	2	2	2	12
Ankyrin	3	6	2	2	2	4
Ankyrin	1	1	2	0	0	0
Bgalactosidase8	1	3	2	2	6	4
CAD	4	2	3	2	2	3
CAD	1	1	1	1	1	1
Calcium11	1	4	2	2	0	3
Calcium11	2	5	2	3	6	2
Calcium-atpase1	1	3	1	1	1	2
Calcium-atpase1	1	0	1	1	1	2
CesA1	3	4	2	2	2	4
EIF-2B	2	2	1	1	1	2
EIF-2B	1	2	1	1	1	2
F5H	1	1	1	1	1	2
F5H	1	4	2	2	2	4
F5H	1	0	2	2	1	2
Formin2	1	5	1	1	0	0
Formin2	1	3	2	2	2	2
Formin2	4	7	2	3	2	2
Glutamine	1	5	3	3	2	1
GPDH	1	2	1	0	0	2
GPDH	1	1	2	1	1	1
GPDH	1	2	2	2	2	3
GPDH	1	6	2	2	2	2
GPDH	2	10	1	2	2	4
Kinesin-KP1	1	2	1	1	1	2
Kinesin-KP1	3	2	1	1	1	2
LOC105792102	2	2	1	1	1	2
LOC105792102	1	3	2	2	2	2
LOC105792102	1	2	2	2	2	4
LOC105792102	1	7	2	2	1	0
LOC105792102	1	1	1	1	1	3
MAP3K	1	4	1	1	1	2
MAP3K	1	3	2	2	2	4
Phospolipase	1	1	1	1	0	0
Phospolipase	1	4	2	2	0	0
Phospolipase	1	2	2	0	0	0
Plasma-ATPase	1	2	1	1	0	0
Plasma-ATPase	1	7	2	3	2	0

Duplicated gene name represents separate paralogous clades. The number of copies were counted for each gene as the number of sequences from one individual in a clade that had *Gossypium* (G) as an outgroup. The abbreviations are short for *Hibiscus syriacus* (S), *H. cannabinus* (C), *H. mechowii* (W), *H. trionum* (T) and *Pavonia triloba* (P)

### Phylogenetic inference

**Fig. 2:**
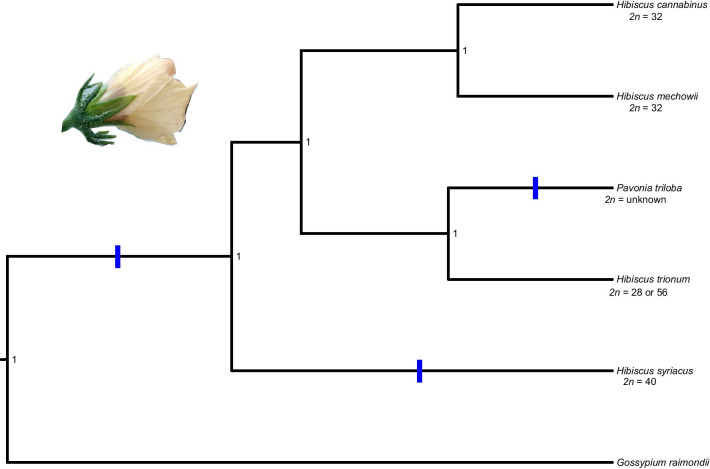
Species tree inferred by StarBEAST using 10 single-copy genes. The blue color indicates the most probable placement of the whole-genome duplication events

For the single-copy gene trees (SCG), 10 out of 14 genes showed the same topological relationships with the /Furcaria clade species forming a clade sister to *H. trionum* + *P. triloba*, and this larger clade in turn sister to *H. syriacus* (Fig. 2), consistent with Pfeil and Crisp [48]. The other four genes often had an extra gene copy from one taxon appearing in a different relationship, indicating either a deep coalescence event or another paralogous copy (e.g. gene Oxysterol-D1, Additional file 3: Figure S1). The phylogenetic trees and subtrees (paralogous clades within one gene tree) strongly support a previously reported relationship [46], with *H. syriacus* sister to /Furcaria + (*H. trionum* + *P. triloba*). In most gene trees, multi-copy genes (MCGs) and SCGs likewise, species of *Pavonia* possessed at least two copies that formed a clade (Additional file 4: Fig. S2, Additional file 5: Fig. S3, Additional file 6: Fig. S4, Additional file 7: Fig. S5, Additional file 8: Fig. S6, Additional file 9: Fig. S7, Additional file 10 Fig. S8, Additional file 11 Fig. S9, Additional file 12: Fig. S10, Additional file 13: Fig. S11, Additional file 14: Fig. S12, Additional file 15: Fig. S13, Additional file 16: Fig. S14, Additional file 17: Fig. S15, Additional file 18: Fig. S16, Additional file 19: Fig. S17, Additional file 20: Fig. S18, Additional file 21: Fig. S19, Additional file 22: Fig. S20, Additional file 23: Fig. S21).

### Scenario testing

A species tree was generated to test amongst three genome evolution hypotheses using the WGDgc R package [49]. All parameters had an ESS value > 200, indicating that the priors had all converged, and a maximum clade credibility tree was created summarizing the clade posterior probabilities on a single tree. The gene count data consisted of 44 data points (subtrees) over 20 MCG (Table 2). The rates of duplication and loss were estimated to be 0.03 and 0.003, respectively. The scenario testing using gene count data showed that S2 (one shared genome duplication with *H. syriacus* and one WGD contained within *H. syriacus*) was the preferred model given the observed distribution of paralogous gene copies in *Hibiscus* (Fig. 2). We found the null-scenario (no WGD events) to be the least likely model to explain the data among the models we evaluated. Through the process of identifying paralogous copies and constructing the gene count data, we found that: (1) /Pavonia species had twice as many copies as /Furcaria species, and (2) the presence of MCG and SCG were congruent with the occurrence of two gene copies within /Pavonia species. Our results thus indicate a third WGD event. All the scenarios had a lower likelihood score with the inclusion of a third WGD event, and the preferred scenario (S2; ωAIC > 0.95) did not change with the inclusion of a third WGD in the clade /Pavonia (Table 3).

**Table 3:** Log-likelihood scores, AIC and weighted AIC from gene count data for each scenario

	loglikelihood	AIC	deltaAIC	rel.LL	AIC weights
*2 WGD*					
S1	− 294.4646	596.9292	8.2118	0.01647	0.0162
S2	− 290.3586	588.7173	0	1	0.9833
S3	− 297.9675	603.9350	15.2176	0.0005	0.0005
Null	− 303.7357	611.4714	22.7540	1.1455e−05	1.1264e−05
*3 WGD*					
S1	− 276.8404	563.6808	13.5126	0.0012	0.0012
S2	− 270.0841	550.1682	0	1	0.9988
S3	− 285.7683	581.5366	31.3684	1.5432e−07	1.5414e−07
Null	− 303.7357	611.4714	61.3032	4.8771e−14	4.8714e−14

Two setups were tested for the three hypothetical WGD scenarios. The first setup tested two WGD events on the three scenarios: S1 where two WGDs are found in *H. syriacus*; S2 where one WGD is shared by all species of *Hibiscus*; and S3 were both WGD are shared by all species of *Hibiscus*. The null hypothesis tests whether no WGD has occurred in *Hibiscus.* The last setup tested an additional WGD within /Pavonia, following the same S1, S2, and S3 scenarios.

## Discussion

While it is widely accepted that recent polyploids originate through complex evolutionary histories, diploid species also often have complicated genomes, preventing accurate phylogenetic inference. In this study, we present evidence that the evolution of *Hibiscus* includes several WGD events. Even diploid species (i.e. not subject to recent polyploidy)—*H. cannabinus* and *H. mechowii*—contained additional copies of genes that were expected to be single copy. Taken together, evidence is consistent with ancient duplications (that duplicate many genes) and the retention of many of these gene lineages, despite a return to diploid genetic state.

We found that WGD events best explain the observed number of sequences in *Hibiscus*. The null-hypothesis—where it is assumed that no WGD events took place in *Hibiscus*—had the lowest likelihood compared to the alternative scenarios. Consequently, single gene duplications are a less likely explanation than WGD for the occurrence of multiple gene copies found within subtrees. Instead, we found that S2 (one genome duplication shared by all *Hibiscus* species and one genome duplication leading to *H. syriacus*), best explained the pattern observed in the trees/gene count data. Within each gene subtree (defined by one *Gossypium* copy as outgroup), *H. syriacus* possessed on average twice as many copies as /Furcaria species, indicating an independent genome duplication leading to *H. syriacus*—consistent with the chromosome number (*2n* = 40; [54]). We corroborate the previous findings of two WGDs in *H. syriacus* [30], but with one modification: one of the duplication events is older than previously presumed (by [30]) and had already occurred somewhere along the branch leading to *Hibiscus*.

### An additional recent polyploid event in *Pavonia*'s past

During the process of identifying sequence copies (alleles and paralogues), we found that /Pavonia*,* within clade /Trionum, always possessed twice as many copies relative to the other species in the clade (i.e. *H. trionum* in our sample). Furthermore, /Pavonia also possessed twice as many copies as /Furcaria, the sister clade to /Trionum, suggesting that a recent genome duplication occurred in /Pavonia. By including a third genome duplication in our scenario testing, we clearly show that part of the data can be explained by an independent genome duplication in /Pavonia. All three scenarios resulted in lower log-likelihood scores when three WGD events were included.

The inferred base chromosome number in /Pavonia—either *n* = 7 or 14—reflects the uncertainty of the genomic history [17]. Here, we found that *P. triloba* underwent a separate genome duplication in addition to the shared one with all species included in *Hibiscus*. However, whether it is a recent duplication within *P. triloba* or a duplication shared with other related species (/Pavonia and /Trionum) cannot be determined here. We infer from our results that the base chromosome number in /Pavonia and /Trionum is likely to be *n* = 14 and not *n* = 7 [17, 34], due to the shared genome duplication with all species in *Hibiscus*. This hypothesis is also supported by the lack of “diploid” species in *Pavonia* with *2n* = 14 [17], if seven is the true haploid chromosome number. No other species in /Trionum have been reported to have *2n* = 14 chromosomes. On the other hand, counts of *2n* = 28 and above have been found *H. trionum* [42], *Malvaviscus arboreus* (incl. in /Pavonia; [62]) and in *Pavonia* species*.*

Additional copies were found within some of the paralogous genes (Additional file 5: Fig. S3, Additional file 6: Fig. S4, Additional file 8: Fig. S10, Additional file 11: Fig. S13) that may either be relicts of older genome duplication events or the consequence of gains of extra copies through independent gene duplication. For example, the Acylamino gene (Additional file 7: Fig. S5) had a third clade consisting of species from /Furcaria and /Pavonia but lacked copies from *H. syriacus* and *Gossypium*. These additional copies suggest an independent gene duplication, or losses of copies in *Gossypium* and *H. syriacus*. Furthermore, in the same gene we found two clades containing *H. cannabinus* gene copies sister to its close relative *H. mechowii* consistent with gene duplication restricted to *H. cannabinus*. These gains and losses of copies are common throughout all the genes and may reflect processes such as independent gene duplications and losses of copies through fractionation or diploidization—complicating an already complex history.

### Data quality

The challenge of separating alleles and copies during sequence read assembly is a crucial one for the success of this study. Current methods typically assume that organisms are diploids and thus can only have two haplotypes at a locus [7]. These assumptions are violated in the presence of more than two haplotypes, such as in polyploid and paleopolyploid plants, where current methods may produce either chimeric haplotypes or an underestimate of the number of haplotypes. Chimeric sequences can also arise through tandem duplications. Cluster analysis is a methodological advance because it identifies the possible number of copies that had been sequenced in the sample, if the sequence copies are distinct from each other in the exon regions (or any used reference region). In contrast, using tools that produce a maximum of two haplotypes/alleles (e.g. Eriksson et al. [14]), we found that most of the copies were not identified and information was lost. One caveat with this approach in this study, however, is the possibility of underestimating the number of copies—sequence copies that we miss due to conserved exon regions, but may have nucleotide differences in the intron regions. While this approach can tease apart distinct haplotypes, it does not separate allelic variants when the polymorphic sites connecting two alleles are too far apart (further away than two paired-end reads can overlap). Thus, possible allelic variants are likely to have been overlooked in this study, as it continues to be impossible to separate variants with current methods.

## Conclusions

Problems with identifying paralogues, homoeologues and allelic variants have negative implications on understanding polyploidy and the processes of diploidization, a common feature found in plants. Previous studies rely on whole genome or transcriptome data to discover ancient genome duplications. We demonstrate here that target sequence capture of a relatively small number of loci can complement existing methods for resolving WGD events. With the information from gene trees and gene count data, new insights into genome duplication were found in diploid and polyploid species of *Hibiscus*. Furthermore, by considering the variation of base chromosome number seen between clades in *Hibiscus*, there are potentially other genome duplications that we have not corroborated in this study. Our results also highlight that even diploid species have complex genomes and that there may be a vast number of diploid species that contain traces of ancient WGDs in other plant groups. Considering the diversity of chromosome numbers in plants, more evidence of ancient genome duplications and processes of diploidization are yet to be uncovered.

## Methods

*Sampling and DNA extraction*—Species with known ploidy were selected to reappraise possible genome duplications in *Hibiscus* (Additional file 2). Two diploid species, with three specimens of *H. cannabinus* L. and one of *H. mechowii* Garcke (both *2n* = 32), were selected from clade /Furcaria (C and M in Fig. 1); *Pavonia triloba* Guill. & Perr. (clade /Pavonia within *Hibiscus*) with unknown chromosome number (P in Fig. 1): *H. trionum* L. from clade /Trionum, a diploid/tetraploid species (*2n* = 28, 56; [9],T in Fig. 1); and two species from previous whole genome sequencing studies: *H. syriacus* from clade /Euhibiscus (GenBank assembly accession GCA_001696755.1; [30]) and *Gossypium raimondii*, the latter not being part of *Hibiscus* (GenBank assembly accession GCF_000327365.1, [44, 77]). Silica dried leaves were collected and DNA was extracted from 25 to 30 mg of plant material using DNeasy Plant mini Kit (Qiagen, Valencia, CA, USA) with two deviations from the manufacturer's protocol: supernatant with AP1 buffer was incubated at 42 °C for 24 h, and a 30 min incubation with AW1 buffer. Samples with excess secondary compounds (polysaccharides) had an additional volume of AP1 buffer added to reduce the viscosity. Samples that discolored the column membrane (e.g. phenol contaminants) incurred an additional step of cleaning with 95% ethanol. Only samples with high quality DNA with an absorbance ratio falling within 1.8–2.0 (260/280 nm and 230/260 nm) were used for the downstream workflow.

### Library preparation

Genomic DNA was sheared using Covaris S220 instrument (Covaris, Woburn, Massachusetts, USA) to a fragment size of 600–800 bp and end-repaired with library NEXTflex rapid DNA-Seq kit (BIOO Scientific, Austin, Texas, USA). End-repaired fragments were barcoded using NEXTflex DNA Barcodes and size selected to optimize recovery of fragments from 600 to 800 bp using Ampure XP beads according to the manufacturer's protocol. A polymerase chain reaction (PCR) was performed using master mix and primer mix provided in the library kit, with the cycling programme: 98 °C, 2′; 14× (98 °C, 30″; 65 °C, 30″; 72 °C, 60″); 72 °C, 4′. PCR products were purified with 0.4 × Agencourt AMPure XP beads (Beckman Coulter) and eluted in 20 µl resuspension buffer.

### Target capture and sequencing

Target gene capture was performed using custom made MYbaits (MYcroarray, Ann Arbor, Michigan), targeting 87 low-copy nuclear genes, designed using the *Hibiscus cannabinus* transcriptome [24, 39, 71, 76] annotated using the *Gossypium raimondii* genome [44, 77]. Probes were selected from regions with exon lengths > 90 bp and intron lengths < 1000 bp. Selected exons were blasted against the *G. raimondii* genome using NCBI megablast with an e-value of 10 (a high e-value was chosen to look for distant homologues between *H. cannabinus* and *G. raimondii*). Only regions with a single copy in the transcriptome and a nucleotide similarity of above 86% to the *Gossypium* genome were accepted.

Six NEXTFlex barcoded libraries were pooled per capture reaction following the protocol from the manufacturer. Each pooled reaction was incubated at 65 °C for 24 h. For libraries prepared from silica dried material, incubation was performed for 16 h. Targeted DNA was captured and purified using Dynabeads MyOne Streptavidin C1 beads (Invitrogen Dynal AS, Oslo, Norway), before PCR amplification with the following programme: 98 °C, 2′; 14x(98 °C, 20″; 65 °C, 30″; 72 °C, 60″); 72 °C, 5′. PCR products were purified using 0.4 × AMPure XP beads. To remove any residue of alcohol, the tubes were air dried until the beads were visibly dry (over-drying beads results in lower yield of captured PCR products) and eluted in 20 µl resuspension buffer. Fragment size length was checked on a Tapestation 2200 (Agilent Technologies) with D1000 tapes and DNA quantity was checked on an Invitrogen™ Qubit™ 3.0 Fluorometer with HS buffer. The sequencing was performed by the SciLifeLab facility in Stockholm, Sweden, on an Illumina MiSeq (San Diego, California, USA) instrument with 300 bp paired-end reads.

### Quality trimming and mapping

The reads were processed with CLC Genomic Workbench (CLC Bio, Aarhus, Denmark) to trim the barcodes and Illumina adaptors from the reads. Low-quality reads (with a phred-score quality threshold of 20) and duplicate reads were removed. Each sample was individually mapped to the targeted probe sequences with a similarity score of 0.7. Mapped probes were sorted using Samtools v.1.3.14 [35], retaining the information of the read names and their position with respect to the probes.

We constructed a pipeline that assembles sequence copies that may be haplotypes/homoeologues, hereafter multiple variants, by mapping to the references in two steps: the first step (URL: https://github.com/DomBennett/Project-cluster) assembles clusters of identical reads corresponding to all the captured target regions. The second step iteratively adds flanking regions where reads support, to build the original genomic sequences without joining parts of sequences together that come from different copies. The first step in the pipeline uses the SAM files and the tool CD-HIT [18, 36] to identify multiple variants by clustering similar reads. In brief, reads mapped to one of the exons are removed when found outside the exon boundaries. CD-HIT then identifies reads that are similar above a certain threshold. We used a 1.0 similarity score and a minimum length of 60 bp. If CD-HIT finds a read that does not have sufficient similarity with a cluster, that read forms a new cluster. Clusters that were represented by only 10 reads or less were deleted.

The second part of the pipeline used the mapping tool in Geneious v11.1.3 (https://www.geneious.com, [29]) to reconstruct full sequences (i.e., containing both exons and introns) from the identified clusters. The exon that had the highest number of clusters was used for constructing full sequences. A consensus was made for each cluster and used as a reference sequence. We used custom settings where full reads (reads that contain both exon and intron sequence data) mapped to the reference had to be without mismatches or gaps, and a word length of 99 characters. Each assembly was iterated five times, where the consensus sequence made from each assembly served as a new reference for the next iteration. We removed copy assemblies that contained positions with polymorphic sites. This assembly step generates sequences in the form ‘exon–intron-exon’ connecting individual exons by adding intervening introns, unless the introns are so long that the iterations do not produce overlapping contigs. Thus, the exons that had fewer clusters were indirectly included by the ‘exon-intron-exon’ assembly step.

The resulting sequences were aligned using MAFFT v7.388 [26, 27] with the auto algorithm (selecting the appropriate method according to the size of data) and default gap penalties. For the gene alignments where sequences did not overlap—due to exons position being too far away and the selection of highest number of clusters may differ between samples—a higher number of iterations (up to 25 times) in the assembly step could in some cases lead to the sequences spanning the entire gene length (all exons). Genomic data from *H. syriacus* and *G. raimondii* were downloaded from NCBI (accession numbers GCA_001696755.1 and GCF_000327365.1, respectively). The probes from each gene were mapped to both genomes to find the location of the singletons or duplicated copies using medium sensitivity/fast settings in Geneious. Sequences from both genomes were added to the alignment using the -add option in MAFFT. Only gene alignments where the sequences overlapped the same exon or the neighboring exons were used for phylogenetic analyses, that resulted in 20 multi-copy genes (MCG, where diploid species have more than one haplotype) and nine single-copy genes (SCG, where diploids only have one haplotype). The rest of the genes were either incomplete due to missing taxa or because of non-overlapping sequences in the alignments.

In one of the gene alignments (Phospholipase), one *Pavonia* copy had the 5′ and the 3′ end of two sequences apparently swapped, likely due to a recombination event between two copies. We inspected the assembly in order to find any indication of chimeric mapping that could be the result of conserved regions—in which reads accidentally map to multiple copies—however no such indication could be found. In such cases we created two sequences by separating the front and back half of the recombined sequence.

### Phylogenetic analysis

Bayesian inference was performed using MrBayes v3.2.6 [50] for 20 MCG and 14 SCG using a reverse model jumping Markov Chain Monte Carlo method (rjMCMC) to average over all 203 possible combinations of substitution models [50]. We allowed among site rate heterogeneity (using a gamma distribution with shape parameter alpha) for all models and genes, as we expected difference in rates between exons and introns as well as codon positions. The branch length prior (brlenpr) was set to unconstrained exponential molecular clock set to 100, to allow for smaller branch length prior means [37]. All other options were set to program defaults. We ran each analysis on two parallel chains for two independent runs of 10 million generations, sampling every 2,000 generations. We applied a burn-in of 10% after checking convergence such that all parameters had an effective sample size (ESS) > 200 with Tracer v1.6 [13]. Trees were annotated using TreeAnnotator v1.8.1 (part of the BEAST package) before being visualized in Figtree v1.4.2 (tree.bio.ed.ac.uk/software/figtree/).

A species tree was constructed for WGD scenario testing using ten SCG that contained one copy per specimen. The analysis was run under the SpeciesTreeUCLN template in BEAST2 [6] with a three-rate substitution model (TR93; [59]) chosen by comparing all tracer files from all genes using a MrBayes v3.2.6 mixed model selection with a mean k-revmat of 3.12 [21]. We employed a birth–death process for the tree prior and an uncorrelated lognormal relaxed molecular clock model [12] set to 0.0055 subs/site/Ma based on a priori information for the family [28, 31, 70] was used. The analysis was run for 40 million generations sampling every 5000 generations. The parameters were checked for convergence in Tracer v1.6 and a burn-in of 10% of the trees was removed using TreeAnnotator in BEAST2 package.

### Species tree and scenario testing using likelihood scores

We compared the log-likelihood scores for the observed gene copy numbers in each taxon from the 20 MCG on three WGD scenarios (Fig. 1) using the WGDgc v1.2 R package [49]. WGDgc uses the number of copies across gene families (defined as a gene that contains more than two gene copies of a given taxa) inferred on a species tree. We counted the number of copies in every gene for each species that formed a clade that had at least one *Gossypium* copy as sister to Hibisceae (i.e., duplications that lead to or are within the Hibisceae lineage, and thus may be linked to gene duplication events). Furthermore, each gene may contain several sequence copies that form subtrees (several clades with *Gossypium* copies sister to Hibisceae copies) that would be each be counted as one data point in the gene count data. Extra clades that were missing a *Gossypium* copy were not used. The number of copies were converted into gene count data manually. We used a Dirac delta prior set to 1 for the number of copies at the root, assuming there is always a single copy present at the root. The starting values of the duplication (birth) and loss (death) rates were set to the default values according to the manual and were estimated using maximum likelihood. The type of conditioning for the likelihood calculation was set to “twoOrMore”, allowing for gene families to have two or more genes. On the species tree, we fixed WGD events to the mid-point of the species tree branches according to our three scenarios (e.g. for S1 the tree will have two independent WGD events on the branch leading to S, and so on, as per Fig. 1). Akaike weights (ωAIC) are calculated by estimating the relative model likelihoods by normalizing with the sum of the likelihoods of all models [64] and can be interpreted as the probability that the model is the most likely given the data (gene count) and candidate models (scenarios) [64]. R code for reproducing our analysis can be found on GitHub (https://github.com/AntonelliLab/WGD-scenario-testing-in-hibiscus).

## Supplementary Information

**Additional file 1:** Probe set information. The probe set is designed to target 87 orthologous genes. Totaling of 521 exons with a 3 × tiling that generated 1544 probes.

**Additional file 2: Table S1.** Species name and accession information. BONN stand for Botanische Gärten der Universität Bonn; The Royal Botanic Gardens, Kew; USDA National Plant Germplasm System. *Hibiscus* *trionum* is kept as a cultivar at Christchurch Botanic Gardens (NZ). Number of reads after quality trimming, in parenthesis the percentage of reduced reads from the original raw data. Percent of GC content after the trimming. Percentage of recovered loci per species and the total number of loci. Percentage of recovered probes (designed over exons) per species. Vouchers are deposited at the Gothenburg herbarium (GB).

**Additional file 3: Fig. S1.** Phylogenetic relationships of a single-copy gene (SCG) Oxysterol-D1 inferred by MrBayes.

**Additional file 4: Fig. S2.** MrBayes trees of multi-copy genes (MSC).

**Additional file 5: Fig. S3.** MrBayes trees of multi-copy genes (MSC).

**Additional file 6: Fig. S4.** MrBayes trees of multi-copy genes (MSC).

**Additional file 7: Fig. S5.** MrBayes trees of multi-copy genes (MSC).

**Additional file 8: Fig. S6.** MrBayes trees of multi-copy genes (MSC).

**Additional file 9: Fig. S7.** MrBayes trees of multi-copy genes (MSC).

**Additional file 10: Fig. S8.** MrBayes trees of multi-copy genes (MSC).

**Additional file 11: Fig. S9.** MrBayes trees of multi-copy genes (MSC).

**Additional file 12: Fig. S10.** MrBayes trees of multi-copy genes (MSC).

**Additional file 13: Fig. S11.** MrBayes trees of multi-copy genes (MSC).

**Additional file 14: Fig. S12.** MrBayes trees of multi-copy genes (MSC).

**Additional file 15: Fig. S13.** MrBayes trees of multi-copy genes (MSC).

**Additional file 16: Fig. S14.** MrBayes trees of multi-copy genes (MSC).

**Additional file 17: Fig. S15.** MrBayes trees of multi-copy genes (MSC).

**Additional file 18: Fig. S16.** MrBayes trees of multi-copy genes (MSC).

**Additional file 19: Fig. S17.** MrBayes trees of multi-copy genes (MSC).

**Additional file 20: Fig. S18.** MrBayes trees of multi-copy genes (MSC).

**Additional file 21: Fig. S19.** MrBayes trees of multi-copy genes (MSC).

**Additional file 22: Fig. S20.** MrBayes trees of multi-copy genes (MSC).

**Additional file 23: Fig. S21.** MrBayes trees of multi-copy genes (MSC).

## Data Availability

The data set(s) supporting the results of this article is available in the European Nucleotide Archive (ENA) repository under project identifier PRJEB42449 (https://www.ebi.ac.uk/ena/browser/view/PRJEB42449). The alignments analysed during the study are available in the Dryad repository (https://doi.org/10.5061/dryad.hqbzkh1fc).
