# Supplementary material for: Gene count from target sequence capture places three whole genome duplication events in Hibiscus L. (Malvaceae)
Source: BMC Ecol Evol. 2021 Jun 2;21:107. doi: 10.1186/s12862-021-01751-7 (PMC8170824; doi:10.1186/s12862-021-01751-7)
Supplement: Supplementary file 1 — Additional file 1: Probe set information. The probe set is designed to target 87 orthologous genes. Totaling of 521 exons with a 3 × tiling that generated 1544 probes. [file 12862_2021_1751_MOESM1_ESM.pdf]

>ABC_F3_exon200_0
CCATGACTGAAGTGGCGAGCTCTGTAGTTCACGAGGTGCTAGGCCGCAGAGCGGAGGATGTAGATCAACCAATCA
TTGATTACATCATCAACGTCCTTGC
>ABC_F3_exon200_18
GCTCTGTAGTTCACGAGGTGCTAGGCCGCAGAGCGGAGGATGTAGATCAACCAATCATTGATTACATCATCAACG
TCCTTGCTGACGAAGATTTCGATTT
>ABC_F3_exon200_36
TGCTAGGCCGCAGAGCGGAGGATGTAGATCAACCAATCATTGATTACATCATCAACGTCCTTGCTGACGAAGATT
TCGATTTTGGAGAAGACGGTGATGG
>ABC_F3_exon600_0
TAGCAACGCCATTTAGGATGAATGAAGGTATGGAGGAGGAGGCTCCAAAGAAAAAGCCAGAGCCGGTTGATGGTC
CATTACTGTCTGAACGCGATAAAAT
>ABC_F3_exon600_21
ATGAAGGTATGGAGGAGGAGGCTCCAAAGAAAAAGCCAGAGCCGGTTGATGGTCCATTACTGTCTGAACGCGATA
AAATGAAGCTTGAGAGGAGGAAGAG
>ABC_F3_exon600_41
GCTCCAAAGAAAAAGCCAGAGCCGGTTGATGGTCCATTACTGTCTGAACGCGATAAAATGAAGCTTGAGAGGAGG
AAGAGAAAGGAGGAGCGCCAAAGAG
>ABC_F3_exon820_0
AGGCACAATACCAAATGCATTTAGCAGAGATGGAAGCAGCCAGGGAAGGAATGCCTGTTGTTTGTGTGAATCATG
ACAATAGTGGAGGAGCAGCTGTTAG
>ABC_F3_exon820_15
TGCATTTAGCAGAGATGGAAGCAGCCAGGGAAGGAATGCCTGTTGTTTGTGTGAATCATGACAATAGTGGAGGAG
CAGCTGTTAGGGATATTCATATGGA
>ABC_F3_exon820_30
TGGAAGCAGCCAGGGAAGGAATGCCTGTTGTTTGTGTGAATCATGACAATAGTGGAGGAGCAGCTGTTAGGGATA
TTCATATGGAGAACTTCAATGTTTC
>ABC_F3_exon1200_0
GGTCTTGTGGGAAGAAATGGTACAGGGAAAACAACTTTCCTTAGGTACATGGCTATGCATGCTATTGAAGGCATT
CCTTCGAACTGTCAGATTCTACATG
>ABC_F3_exon1200_15
AATGGTACAGGGAAAACAACTTTCCTTAGGTACATGGCTATGCATGCTATTGAAGGCATTCCTTCGAACTGTCAG
ATTCTACATGTCGAACAAGAAGTAG
>ABC_F3_exon1200_30
ACAACTTTCCTTAGGTACATGGCTATGCATGCTATTGAAGGCATTCCTTCGAACTGTCAGATTCTACATGTCGAA
CAAGAAGTAGTTGGTGATGATACAA
>ABC_F3_exon1550_0
AATGGAAAAAGTGGCGAAGATCTAAATGGGATGCCTGATAAAGATGCCATCTCGCAAAGACTCGAGCAAATATAC
AAAAGGCTTGAGGTCATTGATGCCG
>ABC_F3_exon1550_17
AGATCTAAATGGGATGCCTGATAAAGATGCCATCTCGCAAAGACTCGAGCAAATATACAAAAGGCTTGAGGTCAT
TGATGCCGATTCCGCTGAGGCTCGT
>ABC_F3_exon1550_34
CTGATAAAGATGCCATCTCGCAAAGACTCGAGCAAATATACAAAAGGCTTGAGGTCATTGATGCCGATTCCGCTG
AGGCTCGTGCCGCTTCTATTCTTGC
>ABC_F3_exon3000_0
GGTCTCAGTTTCTCTCCAGAAATGCAGAAGAGGGCAACAAAAACATTTTCTGGAGGATGGAGAATGCGAATTGCT
CTAGCCCGCGCGCTATTTATAGAAC
>ABC_F3_exon3000_14
TCCAGAAATGCAGAAGAGGGCAACAAAAACATTTTCTGGAGGATGGAGAATGCGAATTGCTCTAGCCCGCGCGCT
ATTTATAGAACCTGATTTGTTGCTT
>ABC_F3_exon3000_28
AGAGGGCAACAAAAACATTTTCTGGAGGATGGAGAATGCGAATTGCTCTAGCCCGCGCGCTATTTATAGAACCTG
ATTTGTTGCTTCTTGATGAACCTAC
>ABC_F3_exon3400_0
GGTGGTTACTGACATAATTCACTTGCAAGGACAAAAATTGACAGCCTACAAAGGAGATTATGATGTCTTTGAGAA
GACAAGGCAGGAACAGATTAAGAAC

>ABC_F3_exon3400_22
TTGCAAGGACAAAAATTGACAGCCTACAAAGGAGATTATGATGTCTTTGAGAAGACAAGGCAGGAACAGATTAAG
AACCAACAGAAAGCAGTTGAGGCAA
>ABC_F3_exon3400_43
GCCTACAAAGGAGATTATGATGTCTTTGAGAAGACAAGGCAGGAACAGATTAAGAACCAACAGAAAGCAGTTGAG
GCAAATGAAAAAGCAAGATCCCACA
>ABC-C2_exon6620_0
TTGTGGTGACTGTGATTTCATTCGGCGTGTTTACTTTGCTTGGAGGCGACTTGACACCTGCTAGGGCATTTACAT
CTCTCTCTCTCTTTTCGGTGCTTCG
>ABC-C2_exon6620_10
TGTGATTTCATTCGGCGTGTTTACTTTGCTTGGAGGCGACTTGACACCTGCTAGGGCATTTACATCTCTCTCTCT
CTTTTCGGTGCTTCGTTTCCCCTTA
>ABC-C2_exon6620_20
TTCGGCGTGTTTACTTTGCTTGGAGGCGACTTGACACCTGCTAGGGCATTTACATCTCTCTCTCTCTTTTCGGTG
CTTCGTTTCCCCTTATTCATGCTTC
>ABC-C2_exon7000_0
CAGGTAGTAAATGCAAATGTATCACTAAAACGTTTGGAGGAGCTGTTCCTGTCTGAAGAGAGAGTTCTTCTGCCA
AATCCTCCTCTTGATCCTGAACTAC
>ABC-C2_exon7000_10
ATGCAAATGTATCACTAAAACGTTTGGAGGAGCTGTTCCTGTCTGAAGAGAGAGTTCTTCTGCCAAATCCTCCTC
TTGATCCTGAACTACCTGCAATCCA
>ABC-C2_exon7000_20
ATCACTAAAACGTTTGGAGGAGCTGTTCCTGTCTGAAGAGAGAGTTCTTCTGCCAAATCCTCCTCTTGATCCTGA
ACTACCTGCAATCCAAATAAGAGAC
>ABC-C2_exon7600_0
GGAAAGACCTCACTTATATCTGCAATGCTGGGGGAGCTTCCTCCAATGCCTGATTCCAGAGTTGTTATCAGAGGA
ACAGTTGCTTATGTTCCTCAAGTTT
>ABC-C2_exon7600_10
CACTTATATCTGCAATGCTGGGGGAGCTTCCTCCAATGCCTGATTCCAGAGTTGTTATCAGAGGAACAGTTGCTT
ATGTTCCTCAAGTTTCATGGATTTT
>ABC-C2_exon7600_20
TGCAATGCTGGGGGAGCTTCCTCCAATGCCTGATTCCAGAGTTGTTATCAGAGGAACAGTTGCTTATGTTCCTCA
AGTTTCATGGATTTTCAATGCAACT
>ABC-C2_exon8200_0
ACTGAGATTGGTGAAAGAGGTGTTAATATTAGTGGTGGGCAGAAGCAAAGGGTTTCCATGGCTAGGGCTGTATAT
TCCAATTCAGACGTGTACGTTTTTG
>ABC-C2_exon8200_10
GTGAAAGAGGTGTTAATATTAGTGGTGGGCAGAAGCAAAGGGTTTCCATGGCTAGGGCTGTATATTCCAATTCAG
ACGTGTACGTTTTTGATGACCCCTT
>ABC-C2_exon8200_20
TGTTAATATTAGTGGTGGGCAGAAGCAAAGGGTTTCCATGGCTAGGGCTGTATATTCCAATTCAGACGTGTACGT
TTTTGATGACCCCTTGAGTGCTCTG
>ABC-C2_exon8900_0
CAGGTTTTTGATAAATGTGTAAAGGGAGAATTGAGAGGCAAAACAAGAGTTCTTGTCACGAACCAGCTTCACTTC
CTTTCACAGGTTGATAGAATTATTC
>ABC-C2_exon8900_10
ATAAATGTGTAAAGGGAGAATTGAGAGGCAAAACAAGAGTTCTTGTCACGAACCAGCTTCACTTCCTTTCACAGG
TTGATAGAATTATTCTGGTTCATGA
>ABC-C2_exon8900_20
AAAGGGAGAATTGAGAGGCAAAACAAGAGTTCTTGTCACGAACCAGCTTCACTTCCTTTCACAGGTTGATAGAAT
TATTCTGGTTCATGAAGGAATGGTG
>ABC-C2_exon9200_0
GGAGTAGCAAATGACATGCCCAAAAGTGCAAGCCAAACAAATAAAAAAAAAGAAGGAAAATCTGTTCTCATTAAG
CAGGAAGAAAGGGAAACAGGAGTTG
>ABC-C2_exon9200_10
ATGACATGCCCAAAAGTGCAAGCCAAACAAATAAAAAAAAAGAAGGAAAATCTGTTCTCATTAAGCAGGAAGAAA
GGGAAACAGGAGTTGTTAGCTGGAA

>ABC-C2_exon9200_20
CAAAAGTGCAAGCCAAACAAATAAAAAAAAAGAAGGAAAATCTGTTCTCATTAAGCAGGAAGAAAGGGAAACAGG
AGTTGTTAGCTGGAATGTTTTAGCG
>ABC-C2_exon10000_0
CCTAACAGAAGTTCTACGAGTTTCAAGCAGCACATGGCTGAGTTCTTGGACAGATCAAAGTTCTACGAAGACACA
TGGACCCGGTTATTACAATTTGGTC
>ABC-C2_exon10000_10
GTTCTACGAGTTTCAAGCAGCACATGGCTGAGTTCTTGGACAGATCAAAGTTCTACGAAGACACATGGACCCGGT
TATTACAATTTGGTCTATTCACTTC
>ABC-C2_exon10000_20
TTTCAAGCAGCACATGGCTGAGTTCTTGGACAGATCAAAGTTCTACGAAGACACATGGACCCGGTTATTACAATT
TGGTCTATTCACTTCTATCATTTGG
>ABC-C2_exon10500_0
ACCAACCCACTTGGTCGGATTATTAACAGATTTGCAAAGGATTTAGGTGACATTGATCGAAATGTTGCTCCATTC
GTCAATATGTTTTTGGGTCAAGTTT
>ABC-C2_exon10500_10
TTGGTCGGATTATTAACAGATTTGCAAAGGATTTAGGTGACATTGATCGAAATGTTGCTCCATTCGTCAATATGT
TTTTGGGTCAAGTTTCGCAGCTCCT
>ABC-C2_exon10500_20
TATTAACAGATTTGCAAAGGATTTAGGTGACATTGATCGAAATGTTGCTCCATTCGTCAATATGTTTTTGGGTCA
AGTTTCGCAGCTCCTTTCTACTTTT
>ABC-C2_exon10900_0
CAGAGTACAGCACGTGAAGTGAAGCGCTTAGATTCTATAACTAGATCACCTGTATATGCACAATTTGGAGAAGCA
TTGAATGGTCTGTCAACTATTCGTG
>ABC-C2_exon10900_10
CACGTGAAGTGAAGCGCTTAGATTCTATAACTAGATCACCTGTATATGCACAATTTGGAGAAGCATTGAATGGTC
TGTCAACTATTCGTGCATATAAAGC
>ABC-C2_exon10900_20
GAAGCGCTTAGATTCTATAACTAGATCACCTGTATATGCACAATTTGGAGAAGCATTGAATGGTCTGTCAACTAT
TCGTGCATATAAAGCTTATGATCGC
>ABC-C2_exon11200_0
AGTTATGCACTAAACATTACAAGTTTATTGACTGCTGTACTCAGGCTCGCCAGTTTGGCTGAAAATAGTTTAAAT
GCTGTTGAGCGTGTTGGTACATATA
>ABC-C2_exon11200_10
TAAACATTACAAGTTTATTGACTGCTGTACTCAGGCTCGCCAGTTTGGCTGAAAATAGTTTAAATGCTGTTGAGC
GTGTTGGTACATATATAGAGTTGCC
>ABC-C2_exon11200_20
AAGTTTATTGACTGCTGTACTCAGGCTCGCCAGTTTGGCTGAAAATAGTTTAAATGCTGTTGAGCGTGTTGGTAC
ATATATAGAGTTGCCTTCAGAAGCT
>ABC-C2_exon11500_0
AGTGACAAGGTAGGCATTGTTGGAAGGACAGGGGCTGGAAAATCAAGCATGTTAAATGCTTTGTTTCGAATTGTA
GAGCTGGAAAGAGGGAGGATCTTGA
>ABC-C2_exon11500_10
TAGGCATTGTTGGAAGGACAGGGGCTGGAAAATCAAGCATGTTAAATGCTTTGTTTCGAATTGTAGAGCTGGAAA
GAGGGAGGATCTTGATTGATGGTTG
>ABC-C2_exon11500_20
TGGAAGGACAGGGGCTGGAAAATCAAGCATGTTAAATGCTTTGTTTCGAATTGTAGAGCTGGAAAGAGGGAGGAT
CTTGATTGATGGTTGTGATATTGCA
>ABCG7_exon3400_0
CAAGATGGTCATACTGTAATTTGCTCAATACATCAGCCTAGAGGTTCAGTGTATGGTAAATTTGACGACATCGTG
CTGCTAACTGAGGGCACACTTGTTT
>ABCG7_exon3400_24
TCAATACATCAGCCTAGAGGTTCAGTGTATGGTAAATTTGACGACATCGTGCTGCTAACTGAGGGCACACTTGTT
TATTCTGGCCCTGCACATGATCAGC
>ABCG7_exon3400_48
GTGTATGGTAAATTTGACGACATCGTGCTGCTAACTGAGGGCACACTTGTTTATTCTGGCCCTGCACATGATCAG
CCACTGGAATACTTCTCCAGATTTG

>ABCG7_exon4300_0
GGTACCAGTGCCCGGATCATGCAAATCCTGCGGAATTTTTGGCAGATCTTATATCCATTGACTACAGTTCTGCTG
ATAGTGTCTACTCTTCTCAGAAAAG
>ABCG7_exon4300_18
ATGCAAATCCTGCGGAATTTTTGGCAGATCTTATATCCATTGACTACAGTTCTGCTGATAGTGTCTACTCTTCTC
AGAAAAGAATTGATGCTCTTGTTGA
>ABCG7_exon4300_36
TTTTGGCAGATCTTATATCCATTGACTACAGTTCTGCTGATAGTGTCTACTCTTCTCAGAAAAGAATTGATGCTC
TTGTTGAAGCATTCTCAACACAGTC
>ABCG7_exon5000_0
GCTTCTCGTGATGGACCAACAAATAAAGTTAGAGCTAGAATGTCAATTGCATCAGCTATAATATTTGGATCTGTT
TTCTGGAGAATGGGAAGATCTCAAA
>ABCG7_exon5000_14
ACCAACAAATAAAGTTAGAGCTAGAATGTCAATTGCATCAGCTATAATATTTGGATCTGTTTTCTGGAGAATGGG
AAGATCTCAAACGTCCATACAAGAC
>ABCG7_exon5000_28
TTAGAGCTAGAATGTCAATTGCATCAGCTATAATATTTGGATCTGTTTTCTGGAGAATGGGAAGATCTCAAACGT
CCATACAAGACAGAATGGGGTTGCT
>ABCG7_exon5800_0
TCAGGTTACTGCAATAAACACCGCAATGGCAGCTCTCACAAAGACTGTGGGTGTATTTCCCAAGGAACGTGCAAT
TGTAGACAGGGAACGTGCTAAGGGA
>ABCG7_exon5800_21
CGCAATGGCAGCTCTCACAAAGACTGTGGGTGTATTTCCCAAGGAACGTGCAATTGTAGACAGGGAACGTGCTAA
GGGATCTTATGCATTGGGACCCTAT
>ABCG7_exon5800_41
AGACTGTGGGTGTATTTCCCAAGGAACGTGCAATTGTAGACAGGGAACGTGCTAAGGGATCTTATGCATTGGGAC
CCTATCTTCTTTCCAAATTGATAGC
>ABCG7_exon6360_0
AGATTTGGAAAGTTCTGTGGAATTGTGACTGTGGAGTCTTTCGCTGCATCTGCTATGGGTCTGACAGTAGGGGCC
ATGGTCCCAACCACTGAAGCAGCAA
>ABCG7_exon6360_9
AAGTTCTGTGGAATTGTGACTGTGGAGTCTTTCGCTGCATCTGCTATGGGTCTGACAGTAGGGGCCATGGTCCCA
ACCACTGAAGCAGCAATGGCATTGG
>ABCG7_exon6360_25
TGACTGTGGAGTCTTTCGCTGCATCTGCTATGGGTCTGACAGTAGGGGCCATGGTCCCAACCACTGAAGCAGCAA
TGGCATTGGGACCCTCTCTTATGAC
>ABCG7_exon7330_0
CTCCTCGAGAAAAACAAACCGAAATACCTGCAGCTTAAGGAGCCTGTATCTGAGCAGATTGAACCAAAGCTAAAG
CTTGAACCTTTATTAGAAACTGACC
>ABCG7_exon7330_24
TACCTGCAGCTTAAGGAGCCTGTATCTGAGCAGATTGAACCAAAGCTAAAGCTTGAACCTTTATTAGAAACTGAC
CAACCTCCTCCGATCAAACAAGATG
>ABCG7_exon7330_47
ATCTGAGCAGATTGAACCAAAGCTAAAGCTTGAACCTTTATTAGAAACTGACCAACCTCCTCCGATCAAACAAGA
TGAACAAAATCAGCAAGTTGAATCA
>ACCS_exon600_0
CACCTTATTTTGATGGATGGAAAGCATATGAAACCAATCCTTTTCACCCTATTGATAGACCTGATGGTGTTATTC
AAATGGGTCTCGCCGAGAATCAGGT
>ACCS_exon850_0
AGCTTTGCTTTGAATTCATCAAAAAGTGGCTAATGGAACACCCAGAGGCCTCCCTTTGCACTCCTGAAGGTGTCG
GCAGGTTCAAGGAGACGGCACTTTT
>ACCS_exon850_5
TGCTTTGAATTCATCAAAAAGTGGCTAATGGAACACCCAGAGGCCTCCCTTTGCACTCCTGAAGGTGTCGGCAGG
TTCAAGGAGACGGCACTTTTCCAGG
>ACCS_exon850_10
TGAATTCATCAAAAAGTGGCTAATGGAACACCCAGAGGCCTCCCTTTGCACTCCTGAAGGTGTCGGCAGGTTCAA
GGAGACGGCACTTTTCCAGGATTAT

>ACCS_exon1000_0
AGACCGCATTGTTATGAGTGGTGGAGCAACAGGCGCTCATGAAACGGTTGCCTTTTGCTTGGCTGATCCTGGTGA
AGCATTACTGGTTCCCACTCCTTAT
>ACCS_exon1000_5
GCATTGTTATGAGTGGTGGAGCAACAGGCGCTCATGAAACGGTTGCCTTTTGCTTGGCTGATCCTGGTGAAGCAT
TACTGGTTCCCACTCCTTATTATCC
>ACCS_exon1000_10
GTTATGAGTGGTGGAGCAACAGGCGCTCATGAAACGGTTGCCTTTTGCTTGGCTGATCCTGGTGAAGCATTACTG
GTTCCCACTCCTTATTATCCTGGGT
>ACCS_exon1700_0
GCAGATTCGATCGTGATTTGAGGTGGAGAACAGGAGTAGAACTTGTTCCAGTGATCTGTGAAAGTTGCAATAATT
TCCAAGTCACTAGAAACGCCTTGGA
>ACCS_exon1700_27
GAACAGGAGTAGAACTTGTTCCAGTGATCTGTGAAAGTTGCAATAATTTCCAAGTCACTAGAAACGCCTTGGAAG
CTGCATATGAAAAGGCTGCAGAAGC
>ACCS_exon1700_54
TCTGTGAAAGTTGCAATAATTTCCAAGTCACTAGAAACGCCTTGGAAGCTGCATATGAAAAGGCTGCAGAAGCTG
GCCTCAGAGTCAAGGGATTGCTCAT
>ACCS_exon1700_79
AGTCACTAGAAACGCCTTGGAAGCTGCATATGAAAAGGCTGCAGAAGCTGGCCTCAGAGTCAAGGGATTGCTCAT
CACAAATCCACCCAATCCTTTGGGA
>Acylamino_exon2100_0
CCCAGATGGAAAGTTCCTTGTGTTTCTGTCTGCAAAAGCCTCAGTGGATTCAGGAGCACACTCTGCAACTGATTC
TCTTCACCGAATTGATTGGCCTACT
>Acylamino_exon2100_19
GTGTTTCTGTCTGCAAAAGCCTCAGTGGATTCAGGAGCACACTCTGCAACTGATTCTCTTCACCGAATTGATTGG
CCTACTGATGGCAAGCTGAGCTCAT
>Acylamino_exon2100_38
CCTCAGTGGATTCAGGAGCACACTCTGCAACTGATTCTCTTCACCGAATTGATTGGCCTACTGATGGCAAGCTGA
GCTCATCTACAAAGATCATTGATGT
>Acylamino_exon2550_0
TTCCTGTTGTGAACTGTGCTGAGGATGGCCAGTTCCCGGGTCTTTACTGTTCAAGTTTCCTTAGTAAGCCATGGC
TTTCTGATGGATGCACTATGGTTTT
>Acylamino_exon2550_21
AGGATGGCCAGTTCCCGGGTCTTTACTGTTCAAGTTTCCTTAGTAAGCCATGGCTTTCTGATGGATGCACTATGG
TTTTATCATCCTATTGGCACAGCTG
>Acylamino_exon2550_42
TTTACTGTTCAAGTTTCCTTAGTAAGCCATGGCTTTCTGATGGATGCACTATGGTTTTATCATCCTATTGGCACA
GCTGTCAAGTGATACTTTCTGTAAA
>Acylamino_exon3000_0
TGTGTAGCAGTCCGGTAGACGTTCCTCAAATCAGATATGGCCGACTTGTAGATAAAGTGAATAATACAACCGGAT
GGCATTGGTTAAATGTCTCAAGCCC
>Acylamino_exon3000_11
CCGGTAGACGTTCCTCAAATCAGATATGGCCGACTTGTAGATAAAGTGAATAATACAACCGGATGGCATTGGTTA
AATGTCTCAAGCCCCATTTTCAAAT
>Acylamino_exon3000_22
TCCTCAAATCAGATATGGCCGACTTGTAGATAAAGTGAATAATACAACCGGATGGCATTGGTTAAATGTCTCAAG
CCCCATTTTCAAATGTTCGGAGAAG
>Acylamino_exon3700_0
TTTTGAGGCAATATTTGTATCTTCCAAAGTAAATGATGCACCTGATCCTCTAATAGTGGTCCTTCATGGCGGCCC
CCATTCCGTTTCCTTGTCAAGCTAC
>Acylamino_exon3700_14
TTGTATCTTCCAAAGTAAATGATGCACCTGATCCTCTAATAGTGGTCCTTCATGGCGGCCCCCATTCCGTTTCCT
TGTCAAGCTACTCAAAGGCATTGGC
>Acylamino_exon3700_27
AGTAAATGATGCACCTGATCCTCTAATAGTGGTCCTTCATGGCGGCCCCCATTCCGTTTCCTTGTCAAGCTACTC
AAAGGCATTGGCATTTCTTTCTTCA

>Acylamino_exon4360_0
CAGGATGTCAATGATGTACTTACAGCAATAGATCATGTCATAGAGAAAGGGCTTGCCAACCCATCTAAAATAGCC
GTTCTTGGTGGTTCCCATGGTGGCT
>Acylamino_exon4360_8
CAATGATGTACTTACAGCAATAGATCATGTCATAGAGAAAGGGCTTGCCAACCCATCTAAAATAGCCGTTCTTGG
TGGTTCCCATGGTGGCTTTCTAACC
>Acylamino_exon4360_23
AGCAATAGATCATGTCATAGAGAAAGGGCTTGCCAACCCATCTAAAATAGCCGTTCTTGGTGGTTCCCATGGTGG
CTTTCTAACCACACACTTAATCGGT
>Acylamino_exon4600_0
AGGCACCGGATAAGTTTGTTGCAGCTGCTGCAAGGAATCCAGTTTGTAATCTTTCATCCATGGTTGGCATAACAG
ATATCCCTGATTGGTGTTACGTGGA
>Acylamino_exon4600_17
GTTGCAGCTGCTGCAAGGAATCCAGTTTGTAATCTTTCATCCATGGTTGGCATAACAGATATCCCTGATTGGTGT
TACGTGGAATCTTACGGAACCAATG
>Acylamino_exon4600_33
GGAATCCAGTTTGTAATCTTTCATCCATGGTTGGCATAACAGATATCCCTGATTGGTGTTACGTGGAATCTTACG
GAACCAATGGAAAAACTATCTTTAC
>Acyl-CoA-IBR3_exon300_0
GCCATGTTCGGAGCTGCCACCTTCGCCATTGCAATAGTTCCACGGGCTTTCTTGTTTCCAAGTCGATCGAGTTGG
TCGGCTGCTTCAAGAACCAATAACC
>Acyl-CoA-IBR3_exon300_9
GGAGCTGCCACCTTCGCCATTGCAATAGTTCCACGGGCTTTCTTGTTTCCAAGTCGATCGAGTTGGTCGGCTGCT
TCAAGAACCAATAACCTAGCTTGCT
>Acyl-CoA-IBR3_exon300_25
CCATTGCAATAGTTCCACGGGCTTTCTTGTTTCCAAGTCGATCGAGTTGGTCGGCTGCTTCAAGAACCAATAACC
TAGCTTGCTCTAGTTCTATTCGACA
>Acyl-CoA-IBR3_exon500_0
ATCCGAAAGGAATGAACCATGTTGAGCAATATACTTTCCAAAAGTTTTCCGATTAAGAGCCCTTTGAGCCATTAA
CTGCATGCCCCTCTCAGCGGCACCA
>Acyl-CoA-IBR3_exon500_15
ACCATGTTGAGCAATATACTTTCCAAAAGTTTTCCGATTAAGAGCCCTTTGAGCCATTAACTGCATGCCCCTCTC
AGCGGCACCAATCAGTCTCATGCAG
>Acyl-CoA-IBR3_exon500_29
TATACTTTCCAAAAGTTTTCCGATTAAGAGCCCTTTGAGCCATTAACTGCATGCCCCTCTCAGCGGCACCAATCA
GTCTCATGCAGTGGTGCAGTCTTCC
>Acyl-CoA-IBR3_exon1000_0
AGAATATTCTTTGCTGGCACACGGACATTATCAAAAGAAACTTCTGCATGTCCATGAGGTGCATCGTCAAAGCCG
AAGACTGTTAGTGGTCTCTTTATGG
>Acyl-CoA-IBR3_exon1000_17
CACACGGACATTATCAAAAGAAACTTCTGCATGTCCATGAGGTGCATCGTCAAAGCCGAAGACTGTTAGTGGTCT
CTTTATGGTTATCCCTGGAGTCTTA
>Acyl-CoA-IBR3_exon1000_34
AAGAAACTTCTGCATGTCCATGAGGTGCATCGTCAAAGCCGAAGACTGTTAGTGGTCTCTTTATGGTTATCCCTG
GAGTCTTAATGTCCACTAAGATCAT
>Acyl-CoA-IBR3_exon1700_0
CTTTTAATAGAGCACTCGATGTTGGTTGCATCTGAGGATGCAACTTGTGGTTCAGTCATTGCAAATCCAGAGCGA
ATTGTCCCATCCAGCAATGGAACAA
>Acyl-CoA-IBR3_exon1700_16
CGATGTTGGTTGCATCTGAGGATGCAACTTGTGGTTCAGTCATTGCAAATCCAGAGCGAATTGTCCCATCCAGCA
ATGGAACAAGCCATTCATTTAGTTG
>Acyl-CoA-IBR3_exon1700_31
CTGAGGATGCAACTTGTGGTTCAGTCATTGCAAATCCAGAGCGAATTGTCCCATCCAGCAATGGAACAAGCCATT
CATTTAGTTGTTCTTTAGTCCCATA
>Acyl-CoA-IBR3_exon2200_0
ACCTCCATATTACCAGTGTCTGGTGCACCACAGTTAAAGATCTGCGGAGCACAAAGAGAGCGACCCATAATCTCA
CAAAGATATCCATATTCAAGGTTGG

>Acyl-CoA-IBR3_exon2200_14
AGTGTCTGGTGCACCACAGTTAAAGATCTGCGGAGCACAAAGAGAGCGACCCATAATCTCACAAAGATATCCATA
TTCAAGGTTGGAGAGGCCAGCACCA
>Acyl-CoA-IBR3_exon2200_27
CCACAGTTAAAGATCTGCGGAGCACAAAGAGAGCGACCCATAATCTCACAAAGATATCCATATTCAAGGTTGGAG
AGGCCAGCACCAAGAAAACGATCAT
>Acyl-CoA-IBR3_exon2600_0
GGGATAAATGTGTTCTTCCATGAATTGAATCAATCTGTTTCTCAATTCCTGAACCTTTTTACTTGGAACAAACCT
ACCGCTTCCTTCTGGAAGGCCTTGA
>Acyl-CoA-IBR3_exon2600_9
GTGTTCTTCCATGAATTGAATCAATCTGTTTCTCAATTCCTGAACCTTTTTACTTGGAACAAACCTACCGCTTCC
TTCTGGAAGGCCTTGAACTTTGTTC
>Acyl-CoA-IBR3_exon2600_26
GAATCAATCTGTTTCTCAATTCCTGAACCTTTTTACTTGGAACAAACCTACCGCTTCCTTCTGGAAGGCCTTGAA
CTTTGTTCTCAGTCCCATATTGCCT
>Acyl-CoA-IBR3_exon3060_0
CAGAGGGGGGTTGCTCCGGAAGCACAGTCTTTTTTGCAATAAAAGCCAATGCAGCATCTATGAGACTATTAGCAT
GACGTCCTGTATGTTCAGCACGTTT
>Acyl-CoA-IBR3_exon3060_10
TTGCTCCGGAAGCACAGTCTTTTTTGCAATAAAAGCCAATGCAGCATCTATGAGACTATTAGCATGACGTCCTGT
ATGTTCAGCACGTTTACCTCCTGAG
>Acyl-CoA-IBR3_exon3060_20
AGCACAGTCTTTTTTGCAATAAAAGCCAATGCAGCATCTATGAGACTATTAGCATGACGTCCTGTATGTTCAGCA
CGTTTACCTCCTGAGGCATTACCCT
>Acyl-CoA-IBR3_exon3620_0
CAGCTTCATTGCAGTATTCTGCCAGAAACTCAGCCTGTGTAGGAATCCCTTCAGGAATTCCAATGATCTCAAAAC
CTTTAGCCAGTTCAACTGTATCAAG
>Acyl-CoA-IBR3_exon3620_11
CAGTATTCTGCCAGAAACTCAGCCTGTGTAGGAATCCCTTCAGGAATTCCAATGATCTCAAAACCTTTAGCCAGT
TCAACTGTATCAAGCCCGACTTGTG
>Acyl-CoA-IBR3_exon3620_21
CCAGAAACTCAGCCTGTGTAGGAATCCCTTCAGGAATTCCAATGATCTCAAAACCTTTAGCCAGTTCAACTGTAT
CAAGCCCGACTTGTGCAATGTAAGG
>Acyl-CoA-IBR3_exon4300_0
GCCTCCTGTTGCACCTGAAGCATCTTCTGGAGGAATATTCTTTCGCAGCCAATCAACCAGCTCAAACATCTTGGG
ATTGCTCTCGGGTTTTCCCTCACTA
>Acyl-CoA-IBR3_exon4300_18
AGCATCTTCTGGAGGAATATTCTTTCGCAGCCAATCAACCAGCTCAAACATCTTGGGATTGCTCTCGGGTTTTCC
CTCACTAGTTGAAGCAAGGTATTGC
>Acyl-CoA-IBR3_exon4300_35
TATTCTTTCGCAGCCAATCAACCAGCTCAAACATCTTGGGATTGCTCTCGGGTTTTCCCTCACTAGTTGAAGCAA
GGTATTGCTTAAACCATCTCTCTAT
>Acyl-CoA-IBR3_exon4600_0
CTGTCTTTTACAATAGTTGTCTCGGCGCCCATAGTTTCCCAAACCAATGGCATCAACATTAGCAGAATGTAACGA
CGCCAAAACTTTCGCAGTCGCCAGA
>Acyl-CoA-IBR3_exon4600_10
CAATAGTTGTCTCGGCGCCCATAGTTTCCCAAACCAATGGCATCAACATTAGCAGAATGTAACGACGCCAAAACT
TTCGCAGTCGCCAGATATATCGCCT
>Acyl-CoA-IBR3_exon4600_20
CTCGGCGCCCATAGTTTCCCAAACCAATGGCATCAACATTAGCAGAATGTAACGACGCCAAAACTTTCGCAGTCG
CCAGATATATCGCCTGCCTCCTCTC
>Acyl-CoA-IBR3_exon4900_0
CCGGTAGACTCGGGTCTACAAATATCCGACCTTCCAGGAACTCCATGATATAAAATGCAGTTCCAATCACATTCG
GATCGTTACACAAACAGAAAACCTT
>Acyl-CoA-IBR3_exon4900_10
CGGGTCTACAAATATCCGACCTTCCAGGAACTCCATGATATAAAATGCAGTTCCAATCACATTCGGATCGTTACA
CAAACAGAAAACCTTTGGAACAGGC

>Acyl-CoA-IBR3_exon4900_20
AATATCCGACCTTCCAGGAACTCCATGATATAAAATGCAGTTCCAATCACATTCGGATCGTTACACAAACAGAAA
ACCTTTGGAACAGGCACTTTTGTAT
>ADH_exon50_0
ATATATACAACCATTCGAAGCTGCTTTTCTCACTACACTCAGGAGAGAATCTTGTTTAAAATATAAGAAAAAAAA
TCATACGCAATGAGCACTGCTGGTC
>ADH_exon50_8
AACCATTCGAAGCTGCTTTTCTCACTACACTCAGGAGAGAATCTTGTTTAAAATATAAGAAAAAAAATCATACGC
AATGAGCACTGCTGGTCAGGTTATT
>ADH_exon50_16
GAAGCTGCTTTTCTCACTACACTCAGGAGAGAATCTTGTTTAAAATATAAGAAAAAAAATCATACGCAATGAGCA
CTGCTGGTCAGGTTATTCGTTGCAA
>ADH_exon1800_0
AGGAGCTGGGGCTGGATATGGACAAGTTCATCACTCACTCTGTCCCTTTTGCCGACATCAACAAGGCTTTCGACT
ACATGCTGCAAGGCATAGGTCTGAG
>ADH_exon1800_9
GGCTGGATATGGACAAGTTCATCACTCACTCTGTCCCTTTTGCCGACATCAACAAGGCTTTCGACTACATGCTGC
AAGGCATAGGTCTGAGATGCATGAT
>ADH_exon1800_26
TTCATCACTCACTCTGTCCCTTTTGCCGACATCAACAAGGCTTTCGACTACATGCTGCAAGGCATAGGTCTGAGA
TGCATGATTCATTTGGATGCTTAAG
>AglucanP_exon330_0
AGGAAATAATCACCTCGACCATAACCCGAGTTACCCTCTAGAGAGTCAAGAAGTGGGTTGTAGTCATAACTTCCA
AATGCTCCACCTCTTATGTATTGCT
>AglucanP_exon330_16
GACCATAACCCGAGTTACCCTCTAGAGAGTCAAGAAGTGGGTTGTAGTCATAACTTCCAAATGCTCCACCTCTTA
TGTATTGCTTCGCCTCTTCAAACCG
>AglucanP_exon330_31
TACCCTCTAGAGAGTCAAGAAGTGGGTTGTAGTCATAACTTCCAAATGCTCCACCTCTTATGTATTGCTTCGCCT
CTTCAAACCGAGGATCGGGTTTGAA
>AglucanP_exon800_0
ATTATGAGGCAACCATTTAGAGCAAATTTCATGTTGCTTGTCCCACTAGCTTCCATGCCTGCAGTACTAATATGC
TGGGAAAGTTCGCTTCCTGGAATAA
>AglucanP_exon800_19
GAGCAAATTTCATGTTGCTTGTCCCACTAGCTTCCATGCCTGCAGTACTAATATGCTGGGAAAGTTCGCTTCCTG
GAATAAGCATTTCAGCTACGGATAC
>AglucanP_exon800_37
TTGTCCCACTAGCTTCCATGCCTGCAGTACTAATATGCTGGGAAAGTTCGCTTCCTGGAATAAGCATTTCAGCTA
CGGATACATTGTAGTTTGGAACAAA
>AglucanP_exon1400_0
TAGCTATTGACTTCAGGATCACTGTTGACTACTTCTCCAACATCATTCACCAATTTGACTATTCGTTTAGCATTT
GTATAAGTAGCAAATGCTTTCCCTC
>AglucanP_exon1400_26
GACTACTTCTCCAACATCATTCACCAATTTGACTATTCGTTTAGCATTTGTATAAGTAGCAAATGCTTTCCCTCC
TATCATAATGGTGCGAGAAGTTGCA
>AglucanP_exon1400_51
AATTTGACTATTCGTTTAGCATTTGTATAAGTAGCAAATGCTTTCCCTCCTATCATAATGGTGCGAGAAGTTGCA
TTTTTCCGCTGCTCAGGACTCATCT
>AglucanP_exon2000_0
CCTTTAACTTCTTGTACCTATAGATTGCACCCAAAATGTTCAGCAGCTGTCTCTTGTATTCATGAATACGCTTAA
CTTGTATGTCAAATAAACTGTTTGG
>AglucanP_exon2000_17
CTATAGATTGCACCCAAAATGTTCAGCAGCTGTCTCTTGTATTCATGAATACGCTTAACTTGTATGTCAAATAAA
CTGTTTGGATCAATGCTTACTCCTG
>AglucanP_exon2000_34
AATGTTCAGCAGCTGTCTCTTGTATTCATGAATACGCTTAACTTGTATGTCAAATAAACTGTTTGGATCAATGCT
TACTCCTGTTACACGCAATATGTAT

>AglucanP_exon2330_0
TGCAAAACCGAAGCCATCGACGAGGAGTAATACCATTAGTCTTATTTTGGAACTTGGTGGGCCATATTGAGACGT
AATCTGCAAACAACTCAGCCTTTAA
>AglucanP_exon2330_16
TCGACGAGGAGTAATACCATTAGTCTTATTTTGGAACTTGGTGGGCCATATTGAGACGTAATCTGCAAACAACTC
AGCCTTTAAGATGTCACTGTGCAGC
>AglucanP_exon2330_31
ACCATTAGTCTTATTTTGGAACTTGGTGGGCCATATTGAGACGTAATCTGCAAACAACTCAGCCTTTAAGATGTC
ACTGTGCAGCTGCGCTACACCATTC
>AglucanP_exon2560_0
GTATGTGCGGATACCACACATAAGTTTGCCATCCTCACAACTGGTTTCTGGGGATTATGATCCAAGATGCGCATG
CTAGGAAGCTTATGCTCAAGATCAG
>AglucanP_exon2560_14
CACACATAAGTTTGCCATCCTCACAACTGGTTTCTGGGGATTATGATCCAAGATGCGCATGCTAGGAAGCTTATG
CTCAAGATCAGGACGTGTGTCCTTG
>AglucanP_exon2560_28
CCATCCTCACAACTGGTTTCTGGGGATTATGATCCAAGATGCGCATGCTAGGAAGCTTATGCTCAAGATCAGGAC
GTGTGTCCTTGATCATTGCAAGAAA
>AglucanP_exon2770_0
CCTTTTGTCAATTTCTTCTATGATTTCCATGTGGCGAGGAAGAAGCTTCCACATGACAGGTTGAGACCACTTCTC
AAGTGCTTCAGGAAGGACAGTATGA
>AglucanP_exon2770_10
ATTTCTTCTATGATTTCCATGTGGCGAGGAAGAAGCTTCCACATGACAGGTTGAGACCACTTCTCAAGTGCTTCA
GGAAGGACAGTATGATTGGTATAAG
>AglucanP_exon2770_20
TGATTTCCATGTGGCGAGGAAGAAGCTTCCACATGACAGGTTGAGACCACTTCTCAAGTGCTTCAGGAAGGACAG
TATGATTGGTATAAGCAATTGTCCT
>AglucanP_exon3000_0
CCTTTTTGTCACATCCCAAGCCTCATCCCACCCAAGGCCTTCATCATCCATTAGTACGCGCATCAGCTCAGGAAT
TGCAAGCGTAGGATGAGTATCATTC
>AglucanP_exon3000_17
AAGCCTCATCCCACCCAAGGCCTTCATCATCCATTAGTACGCGCATCAGCTCAGGAATTGCAAGCGTAGGATGAG
TATCATTCAGTTGTACAGCTACCTG
>AglucanP_exon3000_34
AGGCCTTCATCATCCATTAGTACGCGCATCAGCTCAGGAATTGCAAGCGTAGGATGAGTATCATTCAGTTGTACA
GCTACCTGGCTGGGAAATTCAGACC
>Ankyrin_exon130_0
AAGGTGGAGATATGGATCTGGAGAAAGGACCGGTGAGTCCACATCCAGGTCATAATTCTGGCATGGAAGCTTCGC
CAATACCATCACCGTCCTCAACTGC
>Ankyrin_exon130_14
GATCTGGAGAAAGGACCGGTGAGTCCACATCCAGGTCATAATTCTGGCATGGAAGCTTCGCCAATACCATCACCG
TCCTCAACTGCAACAACGCGGACTC
>Ankyrin_exon130_28
ACCGGTGAGTCCACATCCAGGTCATAATTCTGGCATGGAAGCTTCGCCAATACCATCACCGTCCTCAACTGCAAC
AACGCGGACTCCGGCTTTGATCCTA
>Ankyrin_exon770_0
CTTGTTTACTGCTGCAAAGAAAGGGTACCTCGATGTGGTCAAGGAGTTGCTAAAATACTCGAACAAAGAGACTGT
TACTAAGAAGGATAAATCCGGGTTT
>Ankyrin_exon770_16
AAGAAAGGGTACCTCGATGTGGTCAAGGAGTTGCTAAAATACTCGAACAAAGAGACTGTTACTAAGAAGGATAAA
TCCGGGTTTGATCCGTTGCATATTG
>Ankyrin_exon770_32
ATGTGGTCAAGGAGTTGCTAAAATACTCGAACAAAGAGACTGTTACTAAGAAGGATAAATCCGGGTTTGATCCGT
TGCATATTGCTGCAAGCCAAGGGCA
>Ankyrin_exon1500_0
GATCATGATCCTGGTCTATGCCAAACATTTGGGCCATCGAATGCAACCCCTCTTGTCTCAGCAGCTACCAAAGGG
CATATGGCAGTAGTCAATGAGCTGC

>Ankyrin_exon1500_11
TGGTCTATGCCAAACATTTGGGCCATCGAATGCAACCCCTCTTGTCTCAGCAGCTACCAAAGGGCATATGGCAGT
AGTCAATGAGCTGCTATTGAGAGAT
>Ankyrin_exon1500_22
AAACATTTGGGCCATCGAATGCAACCCCTCTTGTCTCAGCAGCTACCAAAGGGCATATGGCAGTAGTCAATGAGC
TGCTATTGAGAGATGGTGGCTTGCT
>Ankyrin_exon1750_0
ACGGCATTGCACATGGCTGTTAAAGGACAGAGCTCTGAGGTGGTGAGGTTGCTTCTCGAGGCAGATGCTGCTATT
GTGATGCTCCCGGACAAGTTCGGGA
>Ankyrin_exon1750_20
TAAAGGACAGAGCTCTGAGGTGGTGAGGTTGCTTCTCGAGGCAGATGCTGCTATTGTGATGCTCCCGGACAAGTT
CGGGAACACTGCGTTACATGTAGCT
>Ankyrin_exon1750_39
GTGGTGAGGTTGCTTCTCGAGGCAGATGCTGCTATTGTGATGCTCCCGGACAAGTTCGGGAACACTGCGTTACAT
GTAGCTACTCGGAAAAAGAGAGCAG
>Ankyrin_exon2480_0
GTTGTTGTCCCTTCCTGATATGAATGTCAATGCACTCACCAGAGACCATAAAACAGCTCTCGACATAGCTGAAGG
GCTTCCACTCTCTGCAGAATCCACG
>Ankyrin_exon2480_8
CCCTTCCTGATATGAATGTCAATGCACTCACCAGAGACCATAAAACAGCTCTCGACATAGCTGAAGGGCTTCCAC
TCTCTGCAGAATCCACGGAAATAAA
>Ankyrin_exon2480_23
ATGTCAATGCACTCACCAGAGACCATAAAACAGCTCTCGACATAGCTGAAGGGCTTCCACTCTCTGCAGAATCCA
CGGAAATAAAGAGCTGCCTTTCTCG
>ATP-DHX36_exon50_0
TACAGGTTAACTCCATATAATCAAATTGATGATTATGGTCAAGAAAAGATGTGGAAAATGCAGAGACAGGCACAA
TCCTTTAAAAAGAGGAAGAGTCAGT
>ATP-DHX36_exon50_8
AACTCCATATAATCAAATTGATGATTATGGTCAAGAAAAGATGTGGAAAATGCAGAGACAGGCACAATCCTTTAA
AAAGAGGAAGAGTCAGTTAACTTCT
>ATP-DHX36_exon50_15
TATAATCAAATTGATGATTATGGTCAAGAAAAGATGTGGAAAATGCAGAGACAGGCACAATCCTTTAAAAAGAGG
AAGAGTCAGTTAACTTCTGCAGTTG
>ATP-DHX36_exon350_0
CGAGAATCTTTATCCTGTTGGAATCCTGACTCAATTGGTTTCAATCTAATTGAGCATGTTCTTTGCCACATAACA
AAGAAAGAGAGACCTGGTGCTGTTT
>ATP-DHX36_exon350_8
TTTATCCTGTTGGAATCCTGACTCAATTGGTTTCAATCTAATTGAGCATGTTCTTTGCCACATAACAAAGAAAGA
GAGACCTGGTGCTGTTTTGGTTTTC
>ATP-DHX36_exon350_23
TCCTGACTCAATTGGTTTCAATCTAATTGAGCATGTTCTTTGCCACATAACAAAGAAAGAGAGACCTGGTGCTGT
TTTGGTTTTCATGACTGGTTGGGAT
>ATP-DHX36_exon800_0
GCTACTAATATGGCTGAGACTAGCATCACCATCAATGATGTGGTTTTTGTGGTAGATTGTGGAAAGGCAAAAGAA
ACATCCTATGATGCACTAAATAATA
>ATP-DHX36_exon800_24
ATCACCATCAATGATGTGGTTTTTGTGGTAGATTGTGGAAAGGCAAAAGAAACATCCTATGATGCACTAAATAAT
ACTCCTTGTTTGCTTCCATCTTGGA
>ATP-DHX36_exon800_47
TGTGGTAGATTGTGGAAAGGCAAAAGAAACATCCTATGATGCACTAAATAATACTCCTTGTTTGCTTCCATCTTG
GATATCAAAGGCTGCTGCCCGGCAA
>ATP-DHX36_exon1550_0
AGAAGGGGAAGAGCTGGTCGTGTTCAACCTGGCGAGTGTTACCATCTTTATCCCAAATGTGTTTATGATACTTTT
TCTGATTATCAACTTCCAGAGCTTT
>ATP-DHX36_exon1550_15
GGTCGTGTTCAACCTGGCGAGTGTTACCATCTTTATCCCAAATGTGTTTATGATACTTTTTCTGATTATCAACTT
CCAGAGCTTTTAAGGACACCGTTAC

>ATP-DHX36_exon1550_30
GGCGAGTGTTACCATCTTTATCCCAAATGTGTTTATGATACTTTTTCTGATTATCAACTTCCAGAGCTTTTAAGG
ACACCGTTACAGTCTTTATGTTTGC
>ATP-DHX36_exon2030_0
CGCAACTTGTCAATGCTTCCGGTTGAGCCTAAACTTGGGAAAATGCTGATCCTGGGAGCTATTTTCAACTGTTTA
GATCCAATAATGACTGTTGTTGCTG
>ATP-DHX36_exon2030_24
GAGCCTAAACTTGGGAAAATGCTGATCCTGGGAGCTATTTTCAACTGTTTAGATCCAATAATGACTGTTGTTGCT
GGGCTCAGTGTCAGAGATCCATTCC
>ATP-DHX36_exon2030_47
GATCCTGGGAGCTATTTTCAACTGTTTAGATCCAATAATGACTGTTGTTGCTGGGCTCAGTGTCAGAGATCCATT
CCTGATGCCATTTGATAAGAAGGAT
>ATP-DHX36_exon2750_0
GAGCAGTCTGGTTATGAGTATTGTTGGAAGAATTTTCTTTCTGCACAAACTCTCAAGGCTATTCACTCTCTACGA
AAGCAATTCTTCTATTTGCTCAAGG
>ATP-DHX36_exon2750_9
GGTTATGAGTATTGTTGGAAGAATTTTCTTTCTGCACAAACTCTCAAGGCTATTCACTCTCTACGAAAGCAATTC
TTCTATTTGCTCAAGGATACTGGTC
>ATP-DHX36_exon2750_25
GGAAGAATTTTCTTTCTGCACAAACTCTCAAGGCTATTCACTCTCTACGAAAGCAATTCTTCTATTTGCTCAAGG
ATACTGGTCTCGTTGATCAAAATGT
>ATP-DHX36_exon3500_0
TTCCATACCCTTGGCTGGTTTTCAATGAAAAGGTAAAAGTGAATGCGGTATTCCTTCGTGATTCAACTGGTGTTT
CTGATTCTATACTGCTCTTATTTGG
>ATP-DHX36_exon3500_9
CTTGGCTGGTTTTCAATGAAAAGGTAAAAGTGAATGCGGTATTCCTTCGTGATTCAACTGGTGTTTCTGATTCTA
TACTGCTCTTATTTGGAGGGAACAT
>ATP-DHX36_exon3500_25
TGAAAAGGTAAAAGTGAATGCGGTATTCCTTCGTGATTCAACTGGTGTTTCTGATTCTATACTGCTCTTATTTGG
AGGGAACATTTCTAGAGGTGGACTA
>ATP-DHX36_exon4350_0
ATCTTTAACGGGTTGGATTTTATGGGGCAGCCTTGCAGCAACAAGAAGCTTGCCGAAAAGGATGCAGCTGCTCAG
GCTCTGCTGTGGTTGAGAGGTGAAG
>ATP-DHX36_exon4350_14
GGATTTTATGGGGCAGCCTTGCAGCAACAAGAAGCTTGCCGAAAAGGATGCAGCTGCTCAGGCTCTGCTGTGGTT
GAGAGGTGAAGACCATTCATCTTCC
>ATP-DHX36_exon4350_28
AGCCTTGCAGCAACAAGAAGCTTGCCGAAAAGGATGCAGCTGCTCAGGCTCTGCTGTGGTTGAGAGGTGAAGACC
ATTCATCTTCCAGAGATATTGACCA
>ATP-FtsH_exon280_0
GCAAGGTTAAATTGTTGACTTCCAATGAGGTTTCTGGTGTTTCATACGTTGGTAGGTATAGGGACTTTGTTGTAG
ATTTGGAGAGTATTCCTGGTGACAA
>ATP-FtsH_exon280_10
ATTGTTGACTTCCAATGAGGTTTCTGGTGTTTCATACGTTGGTAGGTATAGGGACTTTGTTGTAGATTTGGAGAG
TATTCCTGGTGACAAGAACTTGCAA
>ATP-FtsH_exon280_20
TCCAATGAGGTTTCTGGTGTTTCATACGTTGGTAGGTATAGGGACTTTGTTGTAGATTTGGAGAGTATTCCTGGT
GACAAGAACTTGCAAAGGACTAAAT
>ATP-FtsH_exon1150_0
GTCTTGGGTGGGTAAAGTACCAGAGTATCCTAATCCAGTGGCATCTTCCATATCCAGCAGAGTGATGGTAGAGCT
TGGAATGGTGACAGCTGTAATGGCC
>ATP-FtsH_exon1150_10
GGTAAAGTACCAGAGTATCCTAATCCAGTGGCATCTTCCATATCCAGCAGAGTGATGGTAGAGCTTGGAATGGTG
ACAGCTGTAATGGCCGCTGCAGCAG
>ATP-FtsH_exon1150_20
CAGAGTATCCTAATCCAGTGGCATCTTCCATATCCAGCAGAGTGATGGTAGAGCTTGGAATGGTGACAGCTGTAA
TGGCCGCTGCAGCAGTTCTTGTTGG

>ATP-FtsH_exon1500_0
CACTTTTGGTGGTGTATCTGCTAGTATTGAGATGCTAAAACCAATCACAATTGTCCTTTTAACCATGGTTCTTCT
TGTCCGTTTCACACTTTCAAGAAGA
>ATP-FtsH_exon1500_10
GGTGTATCTGCTAGTATTGAGATGCTAAAACCAATCACAATTGTCCTTTTAACCATGGTTCTTCTTGTCCGTTTC
ACACTTTCAAGAAGACCTAAGAACT
>ATP-FtsH_exon1500_20
CTAGTATTGAGATGCTAAAACCAATCACAATTGTCCTTTTAACCATGGTTCTTCTTGTCCGTTTCACACTTTCAA
GAAGACCTAAGAACTTCAGGAAATG
>ATP-FtsH_exon2250_0
AGACCTTAGTCGCCAAGGCTATAGCTGGTGAAGCAGGTGTTCCATTTTACCAAATGGCAGGCTCAGAATTTGTGG
AAGGTTTAGTGGGTGTTGGTTCGGC
>ATP-FtsH_exon2250_15
AGGCTATAGCTGGTGAAGCAGGTGTTCCATTTTACCAAATGGCAGGCTCAGAATTTGTGGAAGGTTTAGTGGGTG
TTGGTTCGGCTCGTATCAGGGATCT
>ATP-FtsH_exon2250_29
GAAGCAGGTGTTCCATTTTACCAAATGGCAGGCTCAGAATTTGTGGAAGGTTTAGTGGGTGTTGGTTCGGCTCGT
ATCAGGGATCTGTTTAAGAGAGCCA
>ATP-FtsH_exon3110_0
AGACGTCAAGGGATTTTCAAGGAAACAACAGACCAGCTGTATAATGCAGCCACTCAGGAGCGGGAAACTACTTTG
AATCAGCTACTAATAGAACTTGATG
>ATP-FtsH_exon3110_10
GGATTTTCAAGGAAACAACAGACCAGCTGTATAATGCAGCCACTCAGGAGCGGGAAACTACTTTGAATCAGCTAC
TAATAGAACTTGATGGGTTTGATAC
>ATP-FtsH_exon3110_20
GGAAACAACAGACCAGCTGTATAATGCAGCCACTCAGGAGCGGGAAACTACTTTGAATCAGCTACTAATAGAACT
TGATGGGTTTGATACTGGGAAAGGT
>ATP-FtsH_exon3660_0
AGATAAGAATTCGTCCTCCAAATGCAAAGGGGCGATTGCAAATTTTGAAAATTCACGCAAGCAAAGTGAAGATTT
CAGAGTCTGTAGATTTGTCTTCTTA
>ATP-FtsH_exon3660_10
TCGTCCTCCAAATGCAAAGGGGCGATTGCAAATTTTGAAAATTCACGCAAGCAAAGTGAAGATTTCAGAGTCTGT
AGATTTGTCTTCTTATGCAAATAAC
>ATP-FtsH_exon3660_20
AATGCAAAGGGGCGATTGCAAATTTTGAAAATTCACGCAAGCAAAGTGAAGATTTCAGAGTCTGTAGATTTGTCT
TCTTATGCAAATAACTTGCCTGGTA
>ATP-FtsH_exon4000_0
GGACCTAAGCGTGTTGGTATTGATTTGGGTCATCAGGGACAGTGTCGTAGAGCAACCACTGAAGTCGGAGTTGCT
ATGACTTCTCATCTACTTAGGCGAT
>ATP-FtsH_exon4000_10
GTGTTGGTATTGATTTGGGTCATCAGGGACAGTGTCGTAGAGCAACCACTGAAGTCGGAGTTGCTATGACTTCTC
ATCTACTTAGGCGATATGAAAATGC
>ATP-FtsH_exon4000_20
TGATTTGGGTCATCAGGGACAGTGTCGTAGAGCAACCACTGAAGTCGGAGTTGCTATGACTTCTCATCTACTTAG
GCGATATGAAAATGCTGAAGTTGAA
>ATP-FtsH_exon4530_0
AGGTTTTTCTTGGGGGAAGAGCTGCAGAAGAGGTCATTTATGGGCAGGACACCTCGAGGGCATCACTTAACTATC
TTGCAGATGCATCTTGGCTAGCTCG
>ATP-FtsH_exon4530_10
TGGGGGAAGAGCTGCAGAAGAGGTCATTTATGGGCAGGACACCTCGAGGGCATCACTTAACTATCTTGCAGATGC
ATCTTGGCTAGCTCGCAAAATTTTA
>ATP-FtsH_exon4530_20
GCTGCAGAAGAGGTCATTTATGGGCAGGACACCTCGAGGGCATCACTTAACTATCTTGCAGATGCATCTTGGCTA
GCTCGCAAAATTTTAACCATGTTAG
>ATP-FtsH_exon4700_0
ATGGAACTTGGAGAATCCAATGGTCATACATGGAGAACCACCACCTTGGAGAAAGAAAGTTAAATTCGTAGGCCC
ACGTCTAGACTTTGAAGGATCTCTC

>ATP-FtsH_exon4700_10
GAGAATCCAATGGTCATACATGGAGAACCACCACCTTGGAGAAAGAAAGTTAAATTCGTAGGCCCACGTCTAGAC
TTTGAAGGATCTCTCTACGATGACT
>ATP-FtsH_exon4700_20
TGGTCATACATGGAGAACCACCACCTTGGAGAAAGAAAGTTAAATTCGTAGGCCCACGTCTAGACTTTGAAGGAT
CTCTCTACGATGACTACAACCTTAT
>ATP-FtsH_exon5100_0
AGGTTCTTCTGAATCATAAGGAGATCAATGGAGAGGAAATAGACTTCATTTTAAACAAGTACCCTCCTCAAACTC
CGTGGAATCTTGTTTTAGAAGAGGA
>ATP-FtsH_exon5100_10
GAATCATAAGGAGATCAATGGAGAGGAAATAGACTTCATTTTAAACAAGTACCCTCCTCAAACTCCGTGGAATCT
TGTTTTAGAAGAGGAAAACCCAGGC
>ATP-FtsH_exon5100_20
GAGATCAATGGAGAGGAAATAGACTTCATTTTAAACAAGTACCCTCCTCAAACTCCGTGGAATCTTGTTTTAGAA
GAGGAAAACCCAGGCAATCTTCCTT
>Auxin-BIG_exon700_0
TTTGAATCTAATGTTGAAGAGGATATATATGCTGCTACAGTGTTTGCCATGTTACGCCTCTGAGAGGAGCCTCCC
TGTTCGAGAAGGTGACGAGCCATTT
>Auxin-BIG_exon700_8
TAATGTTGAAGAGGATATATATGCTGCTACAGTGTTTGCCATGTTACGCCTCTGAGAGGAGCCTCCCTGTTCGAG
AAGGTGACGAGCCATTTGGATCATG
>Auxin-BIG_exon700_24
ATATATGCTGCTACAGTGTTTGCCATGTTACGCCTCTGAGAGGAGCCTCCCTGTTCGAGAAGGTGACGAGCCATT
TGGATCATGAACGGCAAAAAGCGGG
>Auxin-BIG_exon1000_0
CAGAACAATATCATAAGTCAACAGCCGGAGCCGACTTCCATCAGCACGACCAAGAGCATTGAGGTTATCCCAGTA
TTGATCAACATAGCGAACATACTGT
>Auxin-BIG_exon1000_16
GTCAACAGCCGGAGCCGACTTCCATCAGCACGACCAAGAGCATTGAGGTTATCCCAGTATTGATCAACATAGCGA
ACATACTGTGCTAGTGGAACTGATG
>Auxin-BIG_exon1000_32
GACTTCCATCAGCACGACCAAGAGCATTGAGGTTATCCCAGTATTGATCAACATAGCGAACATACTGTGCTAGTG
GAACTGATGGTCCTCTCACAGGAAA
>Auxin-BIG_exon1400_0
CCTTCTCGACACACCATACAAGCCATCCCATCTTCCTCCTCCTCCACATCTTCCAAACCTTCCAGAAGAGGCCGA
GCAACAACTATTCGCTCACCACCAT
>Auxin-BIG_exon1400_15
ATACAAGCCATCCCATCTTCCTCCTCCTCCACATCTTCCAAACCTTCCAGAAGAGGCCGAGCAACAACTATTCGC
TCACCACCATCTGAAGCTAATTCTT
>Auxin-BIG_exon1400_30
TCTTCCTCCTCCTCCACATCTTCCAAACCTTCCAGAAGAGGCCGAGCAACAACTATTCGCTCACCACCATCTGAA
GCTAATTCTTGTCGCATTCCAAGTC
>Auxin-BIG_exon1900_0
CCTGAAGCAACTCTTCCCTTTTCCTTAGAGCTCGCCGTCTCATTTCATCTCTGGTTGCATGCCGCAACTTACGCA
CCTTCTCCTCCAAGAATCCATCTCC
>Auxin-BIG_exon1900_15
CCCTTTTCCTTAGAGCTCGCCGTCTCATTTCATCTCTGGTTGCATGCCGCAACTTACGCACCTTCTCCTCCAAGA
ATCCATCTCCTTTGCCTTCCTTATC
>Auxin-BIG_exon1900_29
GCTCGCCGTCTCATTTCATCTCTGGTTGCATGCCGCAACTTACGCACCTTCTCCTCCAAGAATCCATCTCCTTTG
CCTTCCTTATCTGATAGAGTGTCCA
>Auxin-BIG_exon2400_0
AATGGTTTCATCTTTAGGATTGTCTTGATGCTGTTTCTGCAATCGGTCAAATTCTCCCCAGTCTTGCAAGTAGGG
GTTAAAATGCTGGATAAGAGCTTCC
>Auxin-BIG_exon2400_8
CATCTTTAGGATTGTCTTGATGCTGTTTCTGCAATCGGTCAAATTCTCCCCAGTCTTGCAAGTAGGGGTTAAAAT
GCTGGATAAGAGCTTCCATAGCATC

>Auxin-BIG_exon2400_23
CTTGATGCTGTTTCTGCAATCGGTCAAATTCTCCCCAGTCTTGCAAGTAGGGGTTAAAATGCTGGATAAGAGCTT
CCATAGCATCAGGTTCACCATAAGT
>Auxin-BIG_exon2800_0
GCCTAAAGCTCCGAGCCTTAATAAGGCCCTCCTATTCTCTCTAATCTTGCAGCAGTACATGAGGAGATTGAGCAC
AGCAACCAACTGCTCCTGGTTGGAC
>Auxin-BIG_exon2800_10
CCGAGCCTTAATAAGGCCCTCCTATTCTCTCTAATCTTGCAGCAGTACATGAGGAGATTGAGCACAGCAACCAAC
TGCTCCTGGTTGGACTTGAAGTCAT
>Auxin-BIG_exon2800_20
ATAAGGCCCTCCTATTCTCTCTAATCTTGCAGCAGTACATGAGGAGATTGAGCACAGCAACCAACTGCTCCTGGT
TGGACTTGAAGTCATCTCGCAAACG
>Auxin-BIG_exon3100_0
CAACAAGATTTCCAACCCATCATACTCACGAACCGCACCAGCTATTGCAAACTCAACCTCAGGGTCTTGTGATTC
TTCTCTATCTTCCTCCAGTTCTTTT
>Auxin-BIG_exon3100_16
CCATCATACTCACGAACCGCACCAGCTATTGCAAACTCAACCTCAGGGTCTTGTGATTCTTCTCTATCTTCCTCC
AGTTCTTTTATCATAGGCTCAGTAG
>Auxin-BIG_exon3100_31
ACCGCACCAGCTATTGCAAACTCAACCTCAGGGTCTTGTGATTCTTCTCTATCTTCCTCCAGTTCTTTTATCATA
GGCTCAGTAGCTTCACCATCTAAAC
>Auxin-BIG_exon3500_0
CCTGAAGTCGGTATGTCACTATCATGGGCGAGCATTCTCTATTTGATGAAACAGCACCAGAGGATAAAAGCGAAG
AGTTAGACAGAGAATTTGAAGATTG
>Auxin-BIG_exon3500_18
CTATCATGGGCGAGCATTCTCTATTTGATGAAACAGCACCAGAGGATAAAAGCGAAGAGTTAGACAGAGAATTTG
AAGATTGACTGTTTGATTTCTTCCA
>Auxin-BIG_exon3500_36
CTCTATTTGATGAAACAGCACCAGAGGATAAAAGCGAAGAGTTAGACAGAGAATTTGAAGATTGACTGTTTGATT
TCTTCCAGACTTGCTCATAAACCTG
>Bgalactosidase8_exon100_0
GCTATTACCAGTTCTGCTCCCTGCAAGCTTCCCATTTATAAAAGCATGAAGAGCATGCCCAAGTGATTCGACATG
GAGAACAGTTTGAGATCCATCTTGA
>Bgalactosidase8_exon100_15
GCTCCCTGCAAGCTTCCCATTTATAAAAGCATGAAGAGCATGCCCAAGTGATTCGACATGGAGAACAGTTTGAGA
TCCATCTTGAAGGAAAGGCTCATCT
>Bgalactosidase8_exon100_30
CCCATTTATAAAAGCATGAAGAGCATGCCCAAGTGATTCGACATGGAGAACAGTTTGAGATCCATCTTGAAGGAA
AGGCTCATCTCCTTTGATATCCATG
>Bgalactosidase8_exon360_0
CTTAATGAATACCACAAGTAGTCACTTTTATCAGCAGTAGTGTTTATTTGCTCTAACAATCCAAGTCTACTAAAT
GCACTAGCCTTTGAGATGCCCACAG
>Bgalactosidase8_exon360_16
AGTAGTCACTTTTATCAGCAGTAGTGTTTATTTGCTCTAACAATCCAAGTCTACTAAATGCACTAGCCTTTGAGA
TGCCCACAGGTTCGTTTATCCAACT
>Bgalactosidase8_exon360_31
CAGCAGTAGTGTTTATTTGCTCTAACAATCCAAGTCTACTAAATGCACTAGCCTTTGAGATGCCCACAGGTTCGT
TTATCCAACTCCAGCCAGACCCGAT
>Bgalactosidase8_exon1150_0
ATCTTGGCAGTATTCAGAGCTACATTCTTGCAATCTGGTAAGATGCTGACAGACCATGCAGGCAAATGGTATGAA
TTGCCATTGAATTTAACAGTTGCAT
>Bgalactosidase8_exon1150_19
CTACATTCTTGCAATCTGGTAAGATGCTGACAGACCATGCAGGCAAATGGTATGAATTGCCATTGAATTTAACAG
TTGCATCAGATTTGGGGTCCACATT
>Bgalactosidase8_exon1150_37
GTAAGATGCTGACAGACCATGCAGGCAAATGGTATGAATTGCCATTGAATTTAACAGTTGCATCAGATTTGGGGT
CCACATTGGCAAGAAAAGCAGAACA

>Bgalactosidase8_exon1700_0
CCTCCAAGTTTGAACCCAAGGAGGAAATTGTAGGATCAGTGGCTATCAATGCTTCTTCGCAAAGCTTTATAGCCT
TATGAACATCTCTTAGGTGACCCCA
>Bgalactosidase8_exon1700_10
TGAACCCAAGGAGGAAATTGTAGGATCAGTGGCTATCAATGCTTCTTCGCAAAGCTTTATAGCCTTATGAACATC
TCTTAGGTGACCCCACTTAGGTTGT
>Bgalactosidase8_exon1700_19
GGAGGAAATTGTAGGATCAGTGGCTATCAATGCTTCTTCGCAAAGCTTTATAGCCTTATGAACATCTCTTAGGTG
ACCCCACTTAGGTTGTCTAACAAGT
>B-galactosidase_exon230_0
ACCTCAATGTTATCCCCTTTGATAAGTTCTTCATTCCGCACTGCCCGATCGAGCTCCACGGTGCTATAATAACTT
GCATTCAGTTGCATGGGTGGAGATC
>B-galactosidase_exon230_9
TTATCCCCTTTGATAAGTTCTTCATTCCGCACTGCCCGATCGAGCTCCACGGTGCTATAATAACTTGCATTCAGT
TGCATGGGTGGAGATCTTCCGAACG
>B-galactosidase_exon230_26
TTCTTCATTCCGCACTGCCCGATCGAGCTCCACGGTGCTATAATAACTTGCATTCAGTTGCATGGGTGGAGATCT
TCCGAACGTTGAAGCATACATTCCA
>B-galactosidase_exon800_0
TTTGTATAGAACAGCTTTCGGCTAGAAAATCTATCTCGTCGATAAGGGCAGCTTTCCACTTAGAGTAATAACTGC
TCGAACCTCCCCCTTTGTCATTATC
>B-galactosidase_exon800_8
GAACAGCTTTCGGCTAGAAAATCTATCTCGTCGATAAGGGCAGCTTTCCACTTAGAGTAATAACTGCTCGAACCT
CCCCCTTTGTCATTATCTGTCGGTG
>B-galactosidase_exon800_24
GAAAATCTATCTCGTCGATAAGGGCAGCTTTCCACTTAGAGTAATAACTGCTCGAACCTCCCCCTTTGTCATTAT
CTGTCGGTGCTCGCCAAAAGCATGG
>B-galactosidase_exon1500_0
ATGAGGTACGATATCTCTTTTGGCAGGCAACTGGACTTGTGTCGATGAGACAACATGACCAGCTTCAACCCACCG
TTTGGGGTGCAAAAGCTTAGCGGTG
>B-galactosidase_exon1500_17
TTTTGGCAGGCAACTGGACTTGTGTCGATGAGACAACATGACCAGCTTCAACCCACCGTTTGGGGTGCAAAAGCT
TAGCGGTGATTGTTAAAATTATTTC
>B-galactosidase_exon1500_34
ACTTGTGTCGATGAGACAACATGACCAGCTTCAACCCACCGTTTGGGGTGCAAAAGCTTAGCGGTGATTGTTAAA
ATTATTTCTACAGAATCTGAGGAAG
>B-galactosidase_exon2050_0
TGTAAGGTAGGATGAGGAGTTCGGTCCGGCCATGTAATACCATTCAAGCAAAAGTTTAAATCATTCGGAGAATCA
CCAAAGTCACCACCATATGCCCAGA
>B-galactosidase_exon2050_16
GAGTTCGGTCCGGCCATGTAATACCATTCAAGCAAAAGTTTAAATCATTCGGAGAATCACCAAAGTCACCACCAT
ATGCCCAGAATTTGCCACCCTTTCC
>B-galactosidase_exon2050_32
TGTAATACCATTCAAGCAAAAGTTTAAATCATTCGGAGAATCACCAAAGTCACCACCATATGCCCAGAATTTGCC
ACCCTTTCCATTGTCCTTCAATAGG
>B-galactosidase_exon2750_0
TTCATTTGGATCATTTGCAATCTTTACTATGTCCCAAACGCGCATATACATTGGGCATATAATATCAGTGGATGG
GGTTCTAGATCCGCCACCTTCATAA
>B-galactosidase_exon2750_18
AATCTTTACTATGTCCCAAACGCGCATATACATTGGGCATATAATATCAGTGGATGGGGTTCTAGATCCGCCACC
TTCATAATGTAATGCCCGTGAAGGG
>B-galactosidase_exon2750_36
AACGCGCATATACATTGGGCATATAATATCAGTGGATGGGGTTCTAGATCCGCCACCTTCATAATGTAATGCCCG
TGAAGGGTCCCTTCCACGAATCCAT
>B-galactosidase_exon3300_0
CCAGCAGAAGCAGAATGATTAGGTCCATATCCAGCCTCATTTCCTAAGGACCACGAAAAAATGCATGCATGATTT
TTGTCCCTTTCAACCATGCCTATAA

>B-galactosidase_exon3300_9
GCAGAATGATTAGGTCCATATCCAGCCTCATTTCCTAAGGACCACGAAAAAATGCATGCATGATTTTTGTCCCTT
TCAACCATGCCTATAACACGGTCCA
>B-galactosidase_exon3300_25
CATATCCAGCCTCATTTCCTAAGGACCACGAAAAAATGCATGCATGATTTTTGTCCCTTTCAACCATGCCTATAA
CACGGTCCATCATTGCAGCAGCCCA
>B-galactosidase_exon3700_0
CCTTAACCATGCAAGCCTCTATGTTAGTCTTCCCCAAACGCGGATGATGCTCATGCCTGTTCACACCTCTTATTA
CAACCGGATGCCCATTAATAAGTAA
>B-galactosidase_exon3700_9
TGCAAGCCTCTATGTTAGTCTTCCCCAAACGCGGATGATGCTCATGCCTGTTCACACCTCTTATTACAACCGGAT
GCCCATTAATAAGTAACTGCTTCGG
>B-galactosidase_exon3700_26
GTCTTCCCCAAACGCGGATGATGCTCATGCCTGTTCACACCTCTTATTACAACCGGATGCCCATTAATAAGTAAC
TGCTTCGGGGCTTTTGATACTTGTC
>B-galactosidase_exon4400_0
ATGTTCAGCAGACCATAGCTTGGGCTTTTCTAGTTTCCCCACAAGCATATTGCCATGGAATCCTAGAGTTTGAGT
GGGGAAGCGGTTGAGCTGTATGTTA
>B-galactosidase_exon4400_16
AGCTTGGGCTTTTCTAGTTTCCCCACAAGCATATTGCCATGGAATCCTAGAGTTTGAGTGGGGAAGCGGTTGAGC
TGTATGTTAGCCACATTCGTCGAAA
>B-galactosidase_exon4400_32
GTTTCCCCACAAGCATATTGCCATGGAATCCTAGAGTTTGAGTGGGGAAGCGGTTGAGCTGTATGTTAGCCACAT
TCGTCGAAAGCAGATCAACATTTCC
>C3H_exon299-415_0
GGCCTATGGTCCGGTCATTTCAGTGTGGTTTGGCTCGACGCTGAACGTGATCGTTTCCAATACGGAACTGGCGAG
GGAAGTTCTGAAAGAGCGTGATCAG
>C3H_exon299-415_9
TCCGGTCATTTCAGTGTGGTTTGGCTCGACGCTGAACGTGATCGTTTCCAATACGGAACTGGCGAGGGAAGTTCT
GAAAGAGCGTGATCAGCAGTTGGCT
>C3H_exon299-415_17
TTTCAGTGTGGTTTGGCTCGACGCTGAACGTGATCGTTTCCAATACGGAACTGGCGAGGGAAGTTCTGAAAGAGC
GTGATCAGCAGTTGGCTGATAGGCA
>C3H_exon507_615_0
TGCACGCTGGAGCTTTTCTCTCCAAAGCGGCTTGAAGCCTTGAGACCCATCAGAGAAGACGAGGTTACTGCCATG
GTTGAATCCATCTTCATCGACTCCA
>C3H_exon507_615_5
GCTGGAGCTTTTCTCTCCAAAGCGGCTTGAAGCCTTGAGACCCATCAGAGAAGACGAGGTTACTGCCATGGTTGA
ATCCATCTTCATCGACTCCACCAAT
>C3H_exon507_615_9
GAGCTTTTCTCTCCAAAGCGGCTTGAAGCCTTGAGACCCATCAGAGAAGACGAGGTTACTGCCATGGTTGAATCC
ATCTTCATCGACTCCACCAATCCTG
>C3H_exon2800_0
TGGGAGCAGTAGCATTCAACAACATAACCAGGCTAGCGTTTGGGAAGCGTTTTGTCAACTCCGAGGGCATAATGG
ACGAGCAAGGCCATGAGTTCAAGGC
>C3H_exon2800_17
AACAACATAACCAGGCTAGCGTTTGGGAAGCGTTTTGTCAACTCCGAGGGCATAATGGACGAGCAAGGCCATGAG
TTCAAGGCCATTGTGGCTAATGGAC
>C3H_exon2800_33
TAGCGTTTGGGAAGCGTTTTGTCAACTCCGAGGGCATAATGGACGAGCAAGGCCATGAGTTCAAGGCCATTGTGG
CTAATGGACTAAAGCTGGGTGCATC
>Cactin_exon1300_0
CATTTTGATCAAAGCAAAGTCAGATCAGAGATTAGATTGCGTGAAGGCCGTATGAAGCCTATTGATGTTTTGTCC
AAGCACCTTAATGGGTCGGATGATA
>Cactin_exon1300_18
GTCAGATCAGAGATTAGATTGCGTGAAGGCCGTATGAAGCCTATTGATGTTTTGTCCAAGCACCTTAATGGGTCG
GATGATATGGATATAGAGTTAAACG

>Cactin_exon1300_36
TTGCGTGAAGGCCGTATGAAGCCTATTGATGTTTTGTCCAAGCACCTTAATGGGTCGGATGATATGGATATAGAG
TTAAACGAACCATACATGGTTTTCA
>Cactin_exon1760_0
TTTCTGATTCAGGGCTTGACAGTTAAAGAGATGGAAGAGCTTCGTGATGACATCAAAATGCATCTCGATTTGGAC
AGGGCAACTCCAACACACATAGTTT
>Cactin_exon1760_8
TCAGGGCTTGACAGTTAAAGAGATGGAAGAGCTTCGTGATGACATCAAAATGCATCTCGATTTGGACAGGGCAAC
TCCAACACACATAGTTTACTGGGAG
>Cactin_exon1760_15
TTGACAGTTAAAGAGATGGAAGAGCTTCGTGATGACATCAAAATGCATCTCGATTTGGACAGGGCAACTCCAACA
CACATAGTTTACTGGGAGGTATTGC
>Cactin_exon2160_0
GCTAGGGTGCGTGGGGAGGAACCTCCTGCTGAGTTGCTAGCAGAAGAAAGGGGTCTGCATTCTAGTATTGAAGAA
GATGTCAAGAATCTCTTGGAAGGGA
>Cactin_exon2160_20
ACCTCCTGCTGAGTTGCTAGCAGAAGAAAGGGGTCTGCATTCTAGTATTGAAGAAGATGTCAAGAATCTCTTGGA
AGGGAAGACCCACAGCGAGCTGGAG
>Cactin_exon2160_40
CAGAAGAAAGGGGTCTGCATTCTAGTATTGAAGAAGATGTCAAGAATCTCTTGGAAGGGAAGACCCACAGCGAGC
TGGAGGCATTACAATCCCAGATTGA
>Cactin_exon2160_58
ATTCTAGTATTGAAGAAGATGTCAAGAATCTCTTGGAAGGGAAGACCCACAGCGAGCTGGAGGCATTACAATCCC
AGATTGAGTCACAGATGCGAACTGG
>Cactin_exon2500_0
AGGCCTGTTTGAAGGAAATACACGCTAAAATGCTGCGTAAACATTTACAACGCCTGGAGCAGCCTTCAGAAGGTG
AAGATAGGGTGGAGTCTGATAGTGG
>Cactin_exon2500_18
TACACGCTAAAATGCTGCGTAAACATTTACAACGCCTGGAGCAGCCTTCAGAAGGTGAAGATAGGGTGGAGTCTG
ATAGTGGTTTAAGACCTGTGGAGGA
>Cactin_exon2500_35
CGTAAACATTTACAACGCCTGGAGCAGCCTTCAGAAGGTGAAGATAGGGTGGAGTCTGATAGTGGTTTAAGACCT
GTGGAGGAGGCTAGTGATCATGATG
>Cactin_exon2730_0
AGATGCTGAAACATTTTCTCCGGAACCGATAATACAAGAAGAGACTCATGAGGTGGAAGAAGAGGCTGGATCCTT
TTCACCAGAACTGTTGCATGGTGAT
>Cactin_exon2730_19
CCGGAACCGATAATACAAGAAGAGACTCATGAGGTGGAAGAAGAGGCTGGATCCTTTTCACCAGAACTGTTGCAT
GGTGATGAAAATGAAGAAGCTATTG
>Cactin_exon2730_38
AAGAGACTCATGAGGTGGAAGAAGAGGCTGGATCCTTTTCACCAGAACTGTTGCATGGTGATGAAAATGAAGAAG
CTATTGACCCAGAGGAGGACAGGGC
>Cactin_exon3000_0
GTTAGAGGAGCAGCAAAGACGGATGCAAGAAGCTTTGGCGTCGAAACCTGCCCCTTCAGAAGACAACTTCGAGCT
AAAAGCTATGAAAGCTATGGGAGCA
>Cactin_exon3000_16
AGACGGATGCAAGAAGCTTTGGCGTCGAAACCTGCCCCTTCAGAAGACAACTTCGAGCTAAAAGCTATGAAAGCT
ATGGGAGCATTGGAGGAGGGTGATG
>Cactin_exon3000_32
CTTTGGCGTCGAAACCTGCCCCTTCAGAAGACAACTTCGAGCTAAAAGCTATGAAAGCTATGGGAGCATTGGAGG
AGGGTGATGCTGTATTTGGCTCTGG
>Cactin_exon3260_0
CAGGTGTATTGGTGGCATGACAAATACCGGCCTAGAAAACCAAAATATTTCAACCGTGTTCACACTGGATATGAG
TGGAACAAATACAATCAGACCCATT
>Cactin_exon3260_11
GTGGCATGACAAATACCGGCCTAGAAAACCAAAATATTTCAACCGTGTTCACACTGGATATGAGTGGAACAAATA
CAATCAGACCCATTATGATCATGAT

>Cactin_exon3260_22
AATACCGGCCTAGAAAACCAAAATATTTCAACCGTGTTCACACTGGATATGAGTGGAACAAATACAATCAGACCC
ATTATGATCATGATAATCCTCCTCC
>CAD_exon100_0
ATGCAGTTTTTTGATCAAAGACCTAAAATGGGTAGCCTTGAAACTGAGAGAAAAACTACGGGGTGGGCAGCTAGA
GACCCTTCAGGCATCTTGTCTCCTT
>CAD_exon100_9
TTTGATCAAAGACCTAAAATGGGTAGCCTTGAAACTGAGAGAAAAACTACGGGGTGGGCAGCTAGAGACCCTTCA
GGCATCTTGTCTCCTTACACTTACT
>CAD_exon100_18
AGACCTAAAATGGGTAGCCTTGAAACTGAGAGAAAAACTACGGGGTGGGCAGCTAGAGACCCTTCAGGCATCTTG
TCTCCTTACACTTACTCTCTCAGGT
>CAD_exon300_0
AGAAACACTGGTCCGGAAGATGTTTTCATCAAGGTTATCTGCTGTGGAATCTGCCATACTGATATTCATCAAGCC
AAAAACGATCTTGGCATGTCAAACT
>CAD_exon300_9
GGTCCGGAAGATGTTTTCATCAAGGTTATCTGCTGTGGAATCTGCCATACTGATATTCATCAAGCCAAAAACGAT
CTTGGCATGTCAAACTACCCGATGG
>CAD_exon300_18
GATGTTTTCATCAAGGTTATCTGCTGTGGAATCTGCCATACTGATATTCATCAAGCCAAAAACGATCTTGGCATG
TCAAACTACCCGATGGTTCCCGGGT
>CAD_exon600_0
CATGAAGTGGTTGGTGAGGTGGTGGAAGTAGGATCAGATGTAGCCAAGTTCGCCGCAGGCGAAATCGTCGGCGTC
GGTTTACTTGTTGGATGTTGCAGAA
>CAD_exon600_22
TGGAAGTAGGATCAGATGTAGCCAAGTTCGCCGCAGGCGAAATCGTCGGCGTCGGTTTACTTGTTGGATGTTGCA
GAAATTGCAGGCCATGTGACACAGA
>CAD_exon600_44
CAAGTTCGCCGCAGGCGAAATCGTCGGCGTCGGTTTACTTGTTGGATGTTGCAGAAATTGCAGGCCATGTGACAC
AGACCGTGAACAATATTGTGCTAAG
>CAD_exon600_66
GTCGGCGTCGGTTTACTTGTTGGATGTTGCAGAAATTGCAGGCCATGTGACACAGACCGTGAACAATATTGTGCT
AAGAAGATCTACTCGTACAACGATG
>CAD_exon600_88
GATGTTGCAGAAATTGCAGGCCATGTGACACAGACCGTGAACAATATTGTGCTAAGAAGATCTACTCGTACAACG
ATGTCTACACCGACGGCAATCCCAC
>CAD_exon600_110
ATGTGACACAGACCGTGAACAATATTGTGCTAAGAAGATCTACTCGTACAACGATGTCTACACCGACGGCAATCC
CACTCGAGGTGGCTTTGCCGGTTCC
>CAD_exon600_129
CAATATTGTGCTAAGAAGATCTACTCGTACAACGATGTCTACACCGACGGCAATCCCACTCGAGGTGGCTTTGCC
GGTTCCATGGTTGTTGATCAAAAGT
>CAD_exon1200_0
TACAGCCCACTGAACCACTTCGGTTTAACCGCGAGTGGTTTAAGTGGAGGAGTATTAGGACTCGGAGGAGTAGGC
CACATGGGGGTGAAGATAGCGAAAG
>CAD_exon1200_5
CCCACTGAACCACTTCGGTTTAACCGCGAGTGGTTTAAGTGGAGGAGTATTAGGACTCGGAGGAGTAGGCCACAT
GGGGGTGAAGATAGCGAAAGCAATG
>CAD_exon1200_10
TGAACCACTTCGGTTTAACCGCGAGTGGTTTAAGTGGAGGAGTATTAGGACTCGGAGGAGTAGGCCACATGGGGG
TGAAGATAGCGAAAGCAATGGGACA
>calcium11_exon50_0
TTCACACCCATTCCAAAGGCATCAGCTATATTCTTGTTGAGGTTGCTTCTCATGCTTCTGCCTGTTCCTATCCCA
TCTCCCTTTCTCATCATTGCTGCAA
>calcium11_exon50_8
CATTCCAAAGGCATCAGCTATATTCTTGTTGAGGTTGCTTCTCATGCTTCTGCCTGTTCCTATCCCATCTCCCTT
TCTCATCATTGCTGCAAATTCCCCA

>calcium11_exon50_24
GCTATATTCTTGTTGAGGTTGCTTCTCATGCTTCTGCCTGTTCCTATCCCATCTCCCTTTCTCATCATTGCTGCA
AATTCCCCATAATCTATTCTCCCAT
>calcium11_exon330_0
ACTCTTTGCAAGCTTGTTGAAGTTCATCTATGGTGATATAGCCACTACCGTCTTTGTCGAAATAGGTAAAAGCAG
TAAGTATATTTTCCTCTCTCTCCGT
>calcium11_exon330_26
TCTATGGTGATATAGCCACTACCGTCTTTGTCGAAATAGGTAAAAGCAGTAAGTATATTTTCCTCTCTCTCCGTC
TTATTCATGTGCAGTGTCGCAGCGA
>calcium11_exon330_51
CTTTGTCGAAATAGGTAAAAGCAGTAAGTATATTTTCCTCTCTCTCCGTCTTATTCATGTGCAGTGTCGCAGCGA
TGAATTCACCATAGTCGATTGTTCC
>calcium11_exon600_0
TCCATTAATTCAGAGCCCACTTTCTTTAAACCATCTTTGAGTTCCTGAAATGTTATTGTCCCACTGTTGTCTGTG
TCAATCATTTTGAACAACTCTTTCA
>calcium11_exon600_9
TCAGAGCCCACTTTCTTTAAACCATCTTTGAGTTCCTGAAATGTTATTGTCCCACTGTTGTCTGTGTCAATCATT
TTGAACAACTCTTTCAGACCACCGA
>calcium11_exon600_26
TAAACCATCTTTGAGTTCCTGAAATGTTATTGTCCCACTGTTGTCTGTGTCAATCATTTTGAACAACTCTTTCAG
ACCACCGATTTCTTCTTCGGAGAGT
>calcium11_exon900_0
ACATACACGCAAAGCCAACTTTTTAACTTTGTTCATTGCCGAGAACTGCTTCAGGCGTGACAGCACGGCAGAATC
CAAAGGTTTGTCCGGGGCAACTCTA
>calcium11_exon900_10
AAAGCCAACTTTTTAACTTTGTTCATTGCCGAGAACTGCTTCAGGCGTGACAGCACGGCAGAATCCAAAGGTTTG
TCCGGGGCAACTCTATCATCGACAA
>calcium11_exon900_20
TTTTAACTTTGTTCATTGCCGAGAACTGCTTCAGGCGTGACAGCACGGCAGAATCCAAAGGTTTGTCCGGGGCAA
CTCTATCATCGACAATCCACGGGTG
>calcium11_exon1160_0
GGGTGCCTCTCAAGCATCTTTCGTATTAGATCTTTTGCACTTTCAGAGATAGTAGGCCATGGGTCGGATGAGAAG
TCGAGATTCCCATGTAAAATCTGTC
>calcium11_exon1160_11
AAGCATCTTTCGTATTAGATCTTTTGCACTTTCAGAGATAGTAGGCCATGGGTCGGATGAGAAGTCGAGATTCCC
ATGTAAAATCTGTCTGAATATCCCT
>calcium11_exon1160_21
CGTATTAGATCTTTTGCACTTTCAGAGATAGTAGGCCATGGGTCGGATGAGAAGTCGAGATTCCCATGTAAAATC
TGTCTGAATATCCCTGTTTCCGTTT
>calcium11_exon1460_0
CCTCTGGTGCAACATAAAAGGGACTCCCAACCACATCAGTATAATGTTGCCCTGGCTTATAGAAGATAGACAAGC
CAAAATCTGTGGCCTTGAGAACGGC
>calcium11_exon1460_8
GCAACATAAAAGGGACTCCCAACCACATCAGTATAATGTTGCCCTGGCTTATAGAAGATAGACAAGCCAAAATCT
GTGGCCTTGAGAACGGCATCATCAC
>calcium11_exon1460_24
TCCCAACCACATCAGTATAATGTTGCCCTGGCTTATAGAAGATAGACAAGCCAAAATCTGTGGCCTTGAGAACGG
CATCATCACCAGGGGAATCGAACAA
>Calcium-ATPase1_exon1500_0
GTGGAATGGATGCTAATCTTGAAACGATTGCCAAGATTTCTGCTATTTGTAATGATGCTGGTGTAACACATTCTG
ACCATAAGTTTTTAGCTCATGGAAT
>Calcium-ATPase1_exon1500_10
TGCTAATCTTGAAACGATTGCCAAGATTTCTGCTATTTGTAATGATGCTGGTGTAACACATTCTGACCATAAGTT
TTTAGCTCATGGAATGCCTACTGAG
>Calcium-ATPase1_exon1500_20
GAAACGATTGCCAAGATTTCTGCTATTTGTAATGATGCTGGTGTAACACATTCTGACCATAAGTTTTTAGCTCAT
GGAATGCCTACTGAGGCAGCCATAA

>Calcium-ATPase1_exon1900_0
GTTGTTGCCTATGGTGGAATGAGTATGAGCATCGTATTGCAACTCTTGAGTTTGATCGTGATAGGAAGTCCATGG
GTGTTATTGTGAAGTCCAAATCGGG
>Calcium-ATPase1_exon1900_10
ATGGTGGAATGAGTATGAGCATCGTATTGCAACTCTTGAGTTTGATCGTGATAGGAAGTCCATGGGTGTTATTGT
GAAGTCCAAATCGGGAAGAAGGTCA
>Calcium-ATPase1_exon1900_20
GAGTATGAGCATCGTATTGCAACTCTTGAGTTTGATCGTGATAGGAAGTCCATGGGTGTTATTGTGAAGTCCAAA
TCGGGAAGAAGGTCATTGTTAGTGA
>Calcium-ATPase1_exon2300_0
AGGGAGCTGTAGAGAACTTACTTGAGAGAAGCTCAAAGATTCAGTTGCTTGATGGTTCTGTTGTACCACTTGAAC
AAAACTCAAGGATCCTTGTCTCCAA
>Calcium-ATPase1_exon2300_11
GAGAACTTACTTGAGAGAAGCTCAAAGATTCAGTTGCTTGATGGTTCTGTTGTACCACTTGAACAAAACTCAAGG
ATCCTTGTCTCCAATGCTCTTCAAG
>Calcium-ATPase1_exon2300_21
TTGAGAGAAGCTCAAAGATTCAGTTGCTTGATGGTTCTGTTGTACCACTTGAACAAAACTCAAGGATCCTTGTCT
CCAATGCTCTTCAAGATATGTCAAG
>Calcium-ATPase1_exon2700_0
GGACCCTCCACGTCAAGAGGTCCATCAGGCAATTGAGGACTGTAAAGCAGCTGGTATTCGGGTTATGGTTATCAC
TGGAGATAACAAAAATACAGCAGAA
>Calcium-ATPase1_exon2700_15
AGAGGTCCATCAGGCAATTGAGGACTGTAAAGCAGCTGGTATTCGGGTTATGGTTATCACTGGAGATAACAAAAA
TACAGCAGAAGCTATATGCCGCGAA
>Calcium-ATPase1_exon2700_29
CAATTGAGGACTGTAAAGCAGCTGGTATTCGGGTTATGGTTATCACTGGAGATAACAAAAATACAGCAGAAGCTA
TATGCCGCGAAATAGGTGTTTTTGG
>Calcium-ATPase1_exon3000_0
GAGATAGTGAGATTGCTAAAGGAAGATGGAGAAGTGGTTGCTATGACTGGTGATGGAGTAAATGATGCACCTGCT
TTGAAGCTGGCTGATATTGGGATTG
>Calcium-ATPase1_exon3000_10
GATTGCTAAAGGAAGATGGAGAAGTGGTTGCTATGACTGGTGATGGAGTAAATGATGCACCTGCTTTGAAGCTGG
CTGATATTGGGATTGCCATGGGCAT
>Calcium-ATPase1_exon3000_20
GGAAGATGGAGAAGTGGTTGCTATGACTGGTGATGGAGTAAATGATGCACCTGCTTTGAAGCTGGCTGATATTGG
GATTGCCATGGGCATCGCTGGAACA
>Calcium-ATPase1_exon3450_0
CGTCTGCCAGGTTGCAAAGGAAGCTTCTGACATGGTGTTGGCAGATGACAATTTTAGTACAATTGTTGCTGCTGT
TGGTGAAGGCAGATCCATTTACAAC
>Calcium-ATPase1_exon3450_10
GTTGCAAAGGAAGCTTCTGACATGGTGTTGGCAGATGACAATTTTAGTACAATTGTTGCTGCTGTTGGTGAAGGC
AGATCCATTTACAACAACATGAAAG
>Calcium-ATPase1_exon3450_20
AAGCTTCTGACATGGTGTTGGCAGATGACAATTTTAGTACAATTGTTGCTGCTGTTGGTGAAGGCAGATCCATTT
ACAACAACATGAAAGCTTTTATCAG
>Calcium-ATPase1_exon3800_0
GGATGGACCACCTGCTACAGCTTTAGGATTCAATCCTCCGGACAAAGACATCATGAAGAAACCTCCTCGGAGAAG
TGACGATTCACTCATCACTGCTTGG
>Calcium-ATPase1_exon3800_10
CCTGCTACAGCTTTAGGATTCAATCCTCCGGACAAAGACATCATGAAGAAACCTCCTCGGAGAAGTGACGATTCA
CTCATCACTGCTTGGATTTTATTCC
>Calcium-ATPase1_exon3800_20
CTTTAGGATTCAATCCTCCGGACAAAGACATCATGAAGAAACCTCCTCGGAGAAGTGACGATTCACTCATCACTG
CTTGGATTTTATTCCGCTATCTGGT
>Calcium-ATPase3_exon1530_0
AAACGAATGGCTCGATTGAATGCTATTGTTCGATCTTTGCCATCAGTAGAAACTTTAGGCTGCACCACAGTGATT
TGCAGTGACAAGACAGGAACTCTGA

>Calcium-ATPase3_exon1530_8
GGCTCGATTGAATGCTATTGTTCGATCTTTGCCATCAGTAGAAACTTTAGGCTGCACCACAGTGATTTGCAGTGA
CAAGACAGGAACTCTGACGACAAAT
>Calcium-ATPase3_exon1530_24
ATTGTTCGATCTTTGCCATCAGTAGAAACTTTAGGCTGCACCACAGTGATTTGCAGTGACAAGACAGGAACTCTG
ACGACAAATATGATGTCTGTTTCAA
>Calcium-ATPase3_exon2050_0
TATCTCAGATATGTGTGTTCCATTCCATGCAAAATGGTCCCGAAGTTGCTGAATTTGGTGTCAGTGGGACAACTT
ATGCTCCAGAAGGTTTTATATTTGA
>Calcium-ATPase3_exon2050_10
ATGTGTGTTCCATTCCATGCAAAATGGTCCCGAAGTTGCTGAATTTGGTGTCAGTGGGACAACTTATGCTCCAGA
AGGTTTTATATTTGACAGCACTGGC
>Calcium-ATPase3_exon2050_20
CATTCCATGCAAAATGGTCCCGAAGTTGCTGAATTTGGTGTCAGTGGGACAACTTATGCTCCAGAAGGTTTTATA
TTTGACAGCACTGGCGTTCAGGTAT
>Calcium-ATPase3_exon2600_0
GAATTCCCTGCTCAATTACCTTGTCTTCTTCACATAGCAATGTGCTCAGCTCTATGCAATGAGTCTCTCTTACAG
TATAATCCAGACAAGGGAAACTATG
>Calcium-ATPase3_exon2600_17
ACCTTGTCTTCTTCACATAGCAATGTGCTCAGCTCTATGCAATGAGTCTCTCTTACAGTATAATCCAGACAAGGG
AAACTATGAAAAAATTGGCGAGTCA
>Calcium-ATPase3_exon2600_34
TAGCAATGTGCTCAGCTCTATGCAATGAGTCTCTCTTACAGTATAATCCAGACAAGGGAAACTATGAAAAAATTG
GCGAGTCAACTGAAGTAGCTCTGCG
>Calcium-ATPase3_exon3000_0
TTTGATTCAATGCCTTCTGCTCTGTACATGCTGAGCAAACATGAGAGAGCTTCCTACTGTAATCACTATTGGGAA
AACCAATTTAAAAAGGTTTCTGTTT
>Calcium-ATPase3_exon3000_8
AATGCCTTCTGCTCTGTACATGCTGAGCAAACATGAGAGAGCTTCCTACTGTAATCACTATTGGGAAAACCAATT
TAAAAAGGTTTCTGTTTTAGAGTTC
>Calcium-ATPase3_exon3000_24
TACATGCTGAGCAAACATGAGAGAGCTTCCTACTGTAATCACTATTGGGAAAACCAATTTAAAAAGGTTTCTGTT
TTAGAGTTCTCCCGGGATCGTAAAA
>Callose_exon2700_0
AATATATAAGGGGCAAAAAGCCATGAGATAACAAGGAACCAGCTGCTAAGAGTAAGCAACACAAAGGAAACAGCA
CCCCCGTCCGTGTAACCATATGCGA
>Callose_exon2700_16
AAAGCCATGAGATAACAAGGAACCAGCTGCTAAGAGTAAGCAACACAAAGGAAACAGCACCCCCGTCCGTGTAAC
CATATGCGATGTATACTATGAGAAG
>Callose_exon2700_31
CAAGGAACCAGCTGCTAAGAGTAAGCAACACAAAGGAAACAGCACCCCCGTCCGTGTAACCATATGCGATGTATA
CTATGAGAAGCAATGCCACTTCAAG
>Callose_exon3200_0
TTTCGCACCGCCATGGAGGATAGTTCGTCCAAAATAATGAGTTCTAGTTCCCAATGAGAATGTGAAGAAGACAGA
ACATAACTGAAGCTGCATTGTAATA
>Callose_exon3200_10
CCATGGAGGATAGTTCGTCCAAAATAATGAGTTCTAGTTCCCAATGAGAATGTGAAGAAGACAGAACATAACTGA
AGCTGCATTGTAATAAAACTGAATA
>Callose_exon3200_20
TAGTTCGTCCAAAATAATGAGTTCTAGTTCCCAATGAGAATGTGAAGAAGACAGAACATAACTGAAGCTGCATTG
TAATAAAACTGAATACAGCCTGCAT
>Callose_exon3500_0
CCTTCAGCAATCCCAATTCAAGTATAAAACCCATTATCATAGGGACAGCGGTAAAAACTCCAATCTGAACCAAGA
ATTGAGCATTCAGAGCTGCATCCAA
>Callose_exon3500_17
TCAAGTATAAAACCCATTATCATAGGGACAGCGGTAAAAACTCCAATCTGAACCAAGAATTGAGCATTCAGAGCT
GCATCCAATGCTGTGTTACCCGAAA

>Callose_exon3500_33
TTATCATAGGGACAGCGGTAAAAACTCCAATCTGAACCAAGAATTGAGCATTCAGAGCTGCATCCAATGCTGTGT
TACCCGAAATCTTAGCTTGCCTAGC
>Callose_exon4100_0
AGACATAATAACCAACAGTTGTGAAGAAGAAAGATAGCATTCTAAAGAAATCAAAAAGTTGTCCGAGTCTGTACA
CATCCCTACTCAGGACTTGCTCTCC
>Callose_exon4100_18
TTGTGAAGAAGAAAGATAGCATTCTAAAGAAATCAAAAAGTTGTCCGAGTCTGTACACATCCCTACTCAGGACTT
GCTCTCCATTTCCTCCAGCAACTTT
>Callose_exon4100_36
GCATTCTAAAGAAATCAAAAAGTTGTCCGAGTCTGTACACATCCCTACTCAGGACTTGCTCTCCATTTCCTCCAG
CAACTTTCCCCTCAAATAAAGCAAT
>CesA1_exon383_535_0
ATGATGGAATCTGGGGCTCCGACTTGCCACACTTGTGGTGAGAATGTGGGGTTGAATGCCAATGGTGAACCCTTT
GTGGCTTGCCATGAATGCAGTTTCC
>CesA1_exon383_535_27
CACACTTGTGGTGAGAATGTGGGGTTGAATGCCAATGGTGAACCCTTTGTGGCTTGCCATGAATGCAGTTTCCCC
ATTTGCAAGACTTGTTTCGAGTATG
>CesA1_exon383_535_53
GAATGCCAATGGTGAACCCTTTGTGGCTTGCCATGAATGCAGTTTCCCCATTTGCAAGACTTGTTTCGAGTATGA
CCTCAAGGAAGGTCGAAAAGCTTGC
>CesA1_exon770_0
TGAACTCCTCAGAGAACTTGTTGGACGATGTCGAGAAGGCATCTGCCGATCAATCCACGATGGCTGCACACTTGA
GCAAGCCTCAAGTATTGTTTTTTTtt
>CesA1_exon864_975_0
GCTTTCTTACATTCATGTTGGTTTACACAGGAAGTTGGGATTCATGCAAGACATATAAGCAGTGTGTCTACATTG
GATAGTGGTATGTAATGTCAATTTC
>CesA1_exon864_975_6
TTACATTCATGTTGGTTTACACAGGAAGTTGGGATTCATGCAAGACATATAAGCAGTGTGTCTACATTGGATAGT
GGTATGTAATGTCAATTTCTTCTAA
>CesA1_exon864_975_12
TCATGTTGGTTTACACAGGAAGTTGGGATTCATGCAAGACATATAAGCAGTGTGTCTACATTGGATAGTGGTATG
TAATGTCAATTTCTTCTAATATTTT
>CesA1_exon1040_1184_0
AAATGACCGAGGAGAACGGGAACCCGATTTGGAAGAATCGGGTGGAAAGTTGGAAAGAAAAGAAGAGCAAGAAGA
AAAAGCCTGCAAAAACTAAGGCTGA
>CesA1_exon1040_1184_22
CCCGATTTGGAAGAATCGGGTGGAAAGTTGGAAAGAAAAGAAGAGCAAGAAGAAAAAGCCTGCAAAAACTAAGGC
TGACACAGAGGCTCAAGTCCCACTG
>CesA1_exon1040_1184_44
GAAAGTTGGAAAGAAAAGAAGAGCAAGAAGAAAAAGCCTGCAAAAACTAAGGCTGACACAGAGGCTCAAGTCCCA
CTGAGCAACAAATGGAAGATAAACC
>CesA1_exon1315_1414_0
GGCACTGGATGCTTCCCAGCCCCTCTCGACTATAATTCCAATCCCGAAAAGCAGACTTGCACCATACCGCACTGT
GATCGTTATGCGATTGATCATTCTC
>CesA1_exon1482_1581_0
TAATAAGTGAAATCTGGTTTGCCTTTTCCTGGGTGCTGGATCAGTTCCCTAAGTGGTATCCTATTAACAGGGAAA
CATACATTGACAGACTATCTGCAAG
>CesA1_exon1695_1794_0
ATACGAAAGAGAAGGTGAACTTTCTGAACTTGCTGCAGTGGACTTCTTTGTGAGTACAGTGGATCCATTGAAAGA
GCCTCCATTGATTACTGCCAATACT
>CesA1_exon1931_2040_0
AAAAGTTCTCCATCGAACCACGGGCACCGGAGTTTTACTTCTCACAGAAGATCGATTACTTGAAGGACAAAGTGC
AGCCCTCATTCGTAAAAGAACGTAG
>CesA1_exon1931_2040_5
TTCTCCATCGAACCACGGGCACCGGAGTTTTACTTCTCACAGAAGATCGATTACTTGAAGGACAAAGTGCAGCCC
TCATTCGTAAAAGAACGTAGAGCAA

>CesA1_exon1931_2040_10
CATCGAACCACGGGCACCGGAGTTTTACTTCTCACAGAAGATCGATTACTTGAAGGACAAAGTGCAGCCCTCATT
CGTAAAAGAACGTAGAGCAATGAAA
>CesA1_exon2129_2228_0
AGAGACTATGAAGAGTTCAAAATCCGAATTAATGCTTTAGTTGCAAAGGCTCAGAAAACACCTGAAGAAGGATGG
ACAATGCAAGATGGAACTCCTTGGC
>CesA1_exon2293_2392_0
TGACATCGAAGGAAATGAACTTCCCCGACTGGTTTATGTCTCTCGAGAGAAGAGACCGGGGTACCAACACCACAA
AAAAGCTGGTGCTGAAAATGCTTTG
>Cinnamate4_hydroxide_exon193_312_0
AAACTGGCTTCAAGTCGGCGATGACTTGAACCACCGCAACCTCACTGACTTGACCAAGAAGTTCGGCGACATCTT
CTTGCTCCGCATGGGACAACGTAAC
>Cinnamate4_hydroxide_exon193_312_10
CAAGTCGGCGATGACTTGAACCACCGCAACCTCACTGACTTGACCAAGAAGTTCGGCGACATCTTCTTGCTCCGC
ATGGGACAACGTAACCTCGTCGTCG
>Cinnamate4_hydroxide_exon193_312_20
ATGACTTGAACCACCGCAACCTCACTGACTTGACCAAGAAGTTCGGCGACATCTTCTTGCTCCGCATGGGACAAC
GTAACCTCGTCGTCGTCTCTTCCCC
>Cinnamate4_hydroxide_exon402_512_0
GACAGGACATGGTCTTCACCGTCTACGGTGAGCACTGGCGCAAGATGAGGAGGATCATGACCGTTCCTTTCTTCA
CCAACAAGGTCGTCCAACAGTACCG
>Cinnamate4_hydroxide_exon402_512_6
ACATGGTCTTCACCGTCTACGGTGAGCACTGGCGCAAGATGAGGAGGATCATGACCGTTCCTTTCTTCACCAACA
AGGTCGTCCAACAGTACCGACACGG
>Cinnamate4_hydroxide_exon402_512_11
GTCTTCACCGTCTACGGTGAGCACTGGCGCAAGATGAGGAGGATCATGACCGTTCCTTTCTTCACCAACAAGGTC
GTCCAACAGTACCGACACGGATGGG
>Cinnamate4_hydroxide_exon722_852_0
GAGTACAACTATGGCGATTTCATCCCCATTTTGAGGCCTTTCCTGAGAGGATACTTGAAATTGTGCAAGGAAGTG
AAGGAGATGAGATTGCAGCTTTTCA
>Cinnamate4_hydroxide_exon722_852_16
ATTTCATCCCCATTTTGAGGCCTTTCCTGAGAGGATACTTGAAATTGTGCAAGGAAGTGAAGGAGATGAGATTGC
AGCTTTTCAAGGACTATTTCCTCGA
>Cinnamate4_hydroxide_exon722_852_31
TGAGGCCTTTCCTGAGAGGATACTTGAAATTGTGCAAGGAAGTGAAGGAGATGAGATTGCAGCTTTTCAAGGACT
ATTTCCTCGAGGAAAGGAAGTAAGT
>Cinnamate4_hydroxide_exon944_1089_0
TTGCAGGAAGCTTGCAAGCACAACGAGAAGCGACAACAATGCTCTTAAATGTGCCATCGATCATATTCTTGATGC
TCAGCAGAAAGGAGAGATCAATGAA
>Cinnamate4_hydroxide_exon944_1089_23
CGAGAAGCGACAACAATGCTCTTAAATGTGCCATCGATCATATTCTTGATGCTCAGCAGAAAGGAGAGATCAATG
AAGACAATGTTCTTTACATTGTTGA
>Cinnamate4_hydroxide_exon944_1089_46
AAATGTGCCATCGATCATATTCTTGATGCTCAGCAGAAAGGAGAGATCAATGAAGACAATGTTCTTTACATTGTT
GAGAACATTAATGTTGCTGGTAAGA
>Cinnamate4_hydroxide_exon1867_2002_0
CAGCCATTGAAACAACCTTATGGTCAATTGAATGGGGCATTGCTGAGCTTGTTAACCATCCCCGGATCCAGCAGA
AGCTCCGCGATGAGATCGACACCGT
>Cinnamate4_hydroxide_exon1867_2002_18
TATGGTCAATTGAATGGGGCATTGCTGAGCTTGTTAACCATCCCCGGATCCAGCAGAAGCTCCGCGATGAGATCG
ACACCGTACTCGGACCCGGTGTGCA
>Cinnamate4_hydroxide_exon1867_2002_36
GCATTGCTGAGCTTGTTAACCATCCCCGGATCCAGCAGAAGCTCCGCGATGAGATCGACACCGTACTCGGACCCG
GTGTGCAGGTTACCGAACCCGACAC
>Cinnamoyl-CoA_exon731_840_0
CAATGGCATGACCGTCTGTGTCACCGGTGCCGGCGGCTTCATCGCTTCTTGGATGGTCAAGCTTCTCCTTGAAAA
GGGTTACTCCGTCAAAGGCACCGTA

```
>Cinnamoyl-CoA_exon731_840_5
GCATGACCGTCTGTGTCACCGGTGCCGGCGGCTTCATCGCTTCTTGGATGGTCAAGCTTCTCCTTGAAAAGGGTT
ACTCCGTCAAAGGCACCGTAAGGAA
>Cinnamoyl-CoA_exon731_840_10
ACCGTCTGTGTCACCGGTGCCGGCGGCTTCATCGCTTCTTGGATGGTCAAGCTTCTCCTTGAAAAGGGTTACTCC
GTCAAAGGCACCGTAAGGAACCCAG
>Cinnamoyl-CoA_exon967_1121_0
ATGATCCCAAGAATTCTCATTTGAGAGAGCTTGAAGGTGCAAAGGAGAGACTATCTCTTCACAGAGCCGATCTTC
TTGATTACCCATCTCTTAAGGAAGC
>Cinnamoyl-CoA_exon967_1121_28
GCTTGAAGGTGCAAAGGAGAGACTATCTCTTCACAGAGCCGATCTTCTTGATTACCCATCTCTTAAGGAAGCCAT
TAGTGGTTGTGATGGAGTTTTCCAT
>Cinnamoyl-CoA_exon967_1121_55
TCTTCACAGAGCCGATCTTCTTGATTACCCATCTCTTAAGGAAGCCATTAGTGGTTGTGATGGAGTTTTCCATAC
TGCTTCGCCTGTGACTGATGATCCT
>Cinnamoyl-CoA_exon1664_1851_0
GAACAAATGGTGGAGCCGGCCGTGAATGGCACAAAGAATGTGATAATGGCAGCGGCGGAGGCCAAGGTTCGACGT
GTGGTATTCACGTCTTCGATCGGCG
>Cinnamoyl-CoA_exon1664_1851_44
AATGGCAGCGGCGGAGGCCAAGGTTCGACGTGTGGTATTCACGTCTTCGATCGGCGCAGTGTACATGGACCCCAA
CCGGAGCCCTGATGTGGTTGTTGAT
>Cinnamoyl-CoA_exon1664_1851_66
GTTCGACGTGTGGTATTCACGTCTTCGATCGGCGCAGTGTACATGGACCCCAACCGGAGCCCTGATGTGGTTGTT
GATGAGTCTTGCTGGAGTGATCTGG
>Cinnamoyl-CoA_exon1664_1851_88
CTTCGATCGGCGCAGTGTACATGGACCCCAACCGGAGCCCTGATGTGGTTGTTGATGAGTCTTGCTGGAGTGATC
TGGAGTTCTGCAAAAATACTAAGGT
>Cinnamoyl-CoA_exon2410_2529_0
AATTGGTATTGTTATGGGAAGGCAGTGGCAGAGCAGGCAGCTTGGGAAACCGCCAAGGAAAAAGGGGTGGACCTG
GTGGTGGTAACCCCAGTTCTGGTGT
>Cinnamoyl-CoA_exon2410_2529_10
GTTATGGGAAGGCAGTGGCAGAGCAGGCAGCTTGGGAAACCGCCAAGGAAAAAGGGGTGGACCTGGTGGTGGTAA
CCCCAGTTCTGGTGTTGGGTCCATT
>Cinnamoyl-CoA_exon2410_2529_19
AGGCAGTGGCAGAGCAGGCAGCTTGGGAAACCGCCAAGGAAAAAGGGGTGGACCTGGTGGTGGTAACCCCAGTTC
TGGTGTTGGGTCCATTGCTGCAATC
>DEAD-ATP_exon3000_0
TCTCCCACTCCAATACAAGCACAAACATGGCCCATTGCACTACAGAGTCGGGACATTGTGGCAATTGCTAAAACT
GGTTCTGGTAAAACATTGGGCTACT
>DEAD-ATP_exon3000_16
AAGCACAAACATGGCCCATTGCACTACAGAGTCGGGACATTGTGGCAATTGCTAAAACTGGTTCTGGTAAAACAT
TGGGCTACTTGATTCCTGCTTTCAT
>DEAD-ATP_exon3000_31
CCATTGCACTACAGAGTCGGGACATTGTGGCAATTGCTAAAACTGGTTCTGGTAAAACATTGGGCTACTTGATTC
CTGCTTTCATGCTTTTGAGGCAACG
>DEAD-ATP_exon3360_0
GTGTTTGTATGGTGGAGCACCAAAGGCTAATCAGTTGAAAGAGTTAGATCGAGGAGCTGATATTGTAGTGGCAAC
TCCTGGTCGGCTAAATGACATCCTT
>DEAD-ATP_exon3360_10
GGTGGAGCACCAAAGGCTAATCAGTTGAAAGAGTTAGATCGAGGAGCTGATATTGTAGTGGCAACTCCTGGTCGG
CTAAATGACATCCTTGAAATGAAGA
>DEAD-ATP_exon3360_20
CAAAGGCTAATCAGTTGAAAGAGTTAGATCGAGGAGCTGATATTGTAGTGGCAACTCCTGGTCGGCTAAATGACA
TCCTTGAAATGAAGAAAATTGAGTT
>DEAD-ATP_exon3600_0
CTGATGTACACAGCAACGTGGCCCAAAGAAGTTAGAAAAATAGCGGGTGACCTCCTTGTCAATCCTGTCCAGGTG
AACATTGGCAGTGTTGACCAGCTTG
```

>DEAD-ATP_exon3600_10
CAGCAACGTGGCCCAAAGAAGTTAGAAAAATAGCGGGTGACCTCCTTGTCAATCCTGTCCAGGTGAACATTGGCA
GTGTTGACCAGCTTGCTGCCAATAA
>DEAD-ATP_exon3600_20
GCCCAAAGAAGTTAGAAAAATAGCGGGTGACCTCCTTGTCAATCCTGTCCAGGTGAACATTGGCAGTGTTGACCA
GCTTGCTGCCAATAAGTCTATCACC
>DEAD-ATP_exon4030_0
CAGTATGTTGAGGTTGTCCCCCAGATGGAGAAGGAGAGGCGCCTGAGGCAGATTCTCCAATCTCAAGAACGTGGT
TCAAAGATTATTATTTTTTGCTCCA
>DEAD-ATP_exon4030_10
AGGTTGTCCCCCAGATGGAGAAGGAGAGGCGCCTGAGGCAGATTCTCCAATCTCAAGAACGTGGTTCAAAGATTA
TTATTTTTTGCTCCACTAAGAGGTT
>DEAD-ATP_exon4030_20
CCAGATGGAGAAGGAGAGGCGCCTGAGGCAGATTCTCCAATCTCAAGAACGTGGTTCAAAGATTATTATTTTTTG
CTCCACTAAGAGGTTGTGTGACCAG
>DEAD-ATP_exon4200_0
TTCCATGGAGATAAATCACAGAATGAAAGGGACTGGGTATTGAATCAGTTCCGATCTGGGAAATCCCCAATATTG
GTTGCCACTGATGTTGCTGCCCGTG
>DEAD-ATP_exon4200_10
ATAAATCACAGAATGAAAGGGACTGGGTATTGAATCAGTTCCGATCTGGGAAATCCCCAATATTGGTTGCCACTG
ATGTTGCTGCCCGTGGGCTTGACAT
>DEAD-ATP_exon4200_20
GAATGAAAGGGACTGGGTATTGAATCAGTTCCGATCTGGGAAATCCCCAATATTGGTTGCCACTGATGTTGCTGC
CCGTGGGCTTGACATCAAAGATATA
>DEAD-ATP_exon4530_0
GACTGGAAGTATGCTCCTGACCTGATTCAAGTTCTGGAGAGAGCTAACCAGCATGTGCCTCCTGATGTGCGAGAG
ATAGCTTCTCGTGGTGGGCCTGGTT
>DEAD-ATP_exon4530_8
GTATGCTCCTGACCTGATTCAAGTTCTGGAGAGAGCTAACCAGCATGTGCCTCCTGATGTGCGAGAGATAGCTTC
TCGTGGTGGGCCTGGTTTTGGGAAG
>DEAD-ATP_exon4530_24
ATTCAAGTTCTGGAGAGAGCTAACCAGCATGTGCCTCCTGATGTGCGAGAGATAGCTTCTCGTGGTGGGCCTGGT
TTTGGGAAGGAGCGAGGTGGAATGA
>DNA-Vsubunit_exon1550_0
CAGTGAATGTTGTTCCATCCAGCAATCATGTGGGGACAAAGGGCTTGACATGCCCTGCTTGATGTTCTCTTTCCG
AAATACAAGTGATGATAATCTAGAT
>DNA-Vsubunit_exon1550_18
CCAGCAATCATGTGGGGACAAAGGGCTTGACATGCCCTGCTTGATGTTCTCTTTCCGAAATACAAGTGATGATAA
TCTAGATAGTGCTATACAAATCCTG
>DNA-Vsubunit_exon1550_36
CAAAGGGCTTGACATGCCCTGCTTGATGTTCTCTTTCCGAAATACAAGTGATGATAATCTAGATAGTGCTATACA
AATCCTGGCTGACATCATCTACCCT
>DNA-Vsubunit_exon2250_0
CAACTTGGATAAGAAATCCAAGCAAGACTCAGAAGGGTGAATTGGCTTTGGAAGTTGTTTTGGAGAAATCTGCTG
TGAAGCAGAACGGTGATGCCTGGAG
>DNA-Vsubunit_exon2250_10
AAGAAATCCAAGCAAGACTCAGAAGGGTGAATTGGCTTTGGAAGTTGTTTTGGAGAAATCTGCTGTGAAGCAGAA
CGGTGATGCCTGGAGGACTGTCATT
>DNA-Vsubunit_exon2250_20
AGCAAGACTCAGAAGGGTGAATTGGCTTTGGAAGTTGTTTTGGAGAAATCTGCTGTGAAGCAGAACGGTGATGCC
TGGAGGACTGTCATTGACTGTTGTC
>DNA-Vsubunit_exon2730_0
CAGCGTCTCTCCACTTCTGTGTCAATGGTGACAAGAGGTGTTTTAAAGGAACACCTCATGCTCTTAGCTAACAGC
ATGACATGTTGTGGAAACTTGATTG
>DNA-Vsubunit_exon2730_10
CCACTTCTGTGTCAATGGTGACAAGAGGTGTTTTAAAGGAACACCTCATGCTCTTAGCTAACAGCATGACATGTT
GTGGAAACTTGATTGGCTTCAACTC

```
>DNA-Vsubunit_exon2730_20
GTCAATGGTGACAAGAGGTGTTTTAAAGGAACACCTCATGCTCTTAGCTAACAGCATGACATGTTGTGGAAACTT
GATTGGCTTCAACTCTGGTGGTTAC
>DNA-Vsubunit_exon3000_0
GCTGCAGAGAAATGCTATGATGATTCCTTGTCGAGCATAGTTGCATCCTGTTCTTGGGGTAAACGGGTGTCTGTT
GGAACAGGTTCAAGGTTTGACCTCC
>DNA-Vsubunit_exon3000_10
AATGCTATGATGATTCCTTGTCGAGCATAGTTGCATCCTGTTCTTGGGGTAAACGGGTGTCTGTTGGAACAGGTT
CAAGGTTTGACCTCCTGTGGAATCA
>DNA-Vsubunit_exon3000_20
TGATTCCTTGTCGAGCATAGTTGCATCCTGTTCTTGGGGTAAACGGGTGTCTGTTGGAACAGGTTCAAGGTTTGA
CCTCCTGTGGAATCAGGAGGTATGT
>DNA-Vsubunit_exon3800_0
AGTTTGGATCTGATCAAACGAGTGGGATGGATGTCTACAACTTTCTGCACATGGTTAGTGGTGCTGGTGGAAACA
ATTCTAATACTGCTTGCCTAGGAGA
>DNA-Vsubunit_exon3800_11
GATCAAACGAGTGGGATGGATGTCTACAACTTTCTGCACATGGTTAGTGGTGCTGGTGGAAACAATTCTAATACT
GCTTGCCTAGGAGAAGAAGTTGATG
>DNA-Vsubunit_exon3800_22
TGGGATGGATGTCTACAACTTTCTGCACATGGTTAGTGGTGCTGGTGGAAACAATTCTAATACTGCTTGCCTAGG
AGAAGAAGTTGATGATCTAATGGAT
>E3ubiquitin_exon400_0
TCGGACGAATCGTTGACGGAGAAGGAGAAGGCGAGGAAGAGGCAGGAGCTGGTGAGTGGGCGAGTTGAAGAAGAT
GATGATAAGGAGAAGAAAGGGAAAG
>E3ubiquitin_exon400_30
GCGAGGAAGAGGCAGGAGCTGGTGAGTGGGCGAGTTGAAGAAGATGATGATAAGGAGAAGAAAGGGAAAGGGGAG
GAGAGCTCATTTTTGGATGTTCTTG
>E3ubiquitin_exon400_60
CGAGTTGAAGAAGATGATGATAAGGAGAAGAAAGGGAAAGGGGAGGAGAGCTCATTTTTGGATGTTCTTGATGGA
AGTTTAAACTGTTCGTTTTGCATGC
>E3ubiquitin_exon2000_0
GATGGCAGTGGAGGTAGGGATCTCAGTGGAAATAAGCGGACAAACAAGAAACAGTCATTTGATCAAAAATTTGTG
AACATGAACGAGGCTTTGCGAGTAA
>E3ubiquitin_exon2000_16
GGGATCTCAGTGGAAATAAGCGGACAAACAAGAAACAGTCATTTGATCAAAAATTTGTGAACATGAACGAGGCTT
TGCGAGTAAGCTGCAAAATGGGTTA
>E3ubiquitin_exon2000_32
TAAGCGGACAAACAAGAAACAGTCATTTGATCAAAAATTTGTGAACATGAACGAGGCTTTGCGAGTAAGCTGCAA
AATGGGTTATCCTGTCCGAGTTGTA
>E3ubiquitin_exon3800_0
TGTGCCCACAACTTCTGCAAATCTTGTTTTGAAGCTGCATTTGCCGGTAAGACTACTGTAAGAGAGAGGAGCAGA
GGTGGCCGGACACTTCGATCGCAAA
>E3ubiquitin_exon3800_30
GAAGCTGCATTTGCCGGTAAGACTACTGTAAGAGAGAGGAGCAGAGGTGGCCGGACACTTCGATCGCAAAAAGTT
GTGCTCCATTGTCCTTCTTGCCCTA
>E3ubiquitin_exon3800_60
AGAGAGAGGAGCAGAGGTGGCCGGACACTTCGATCGCAAAAAGTTGTGCTCCATTGTCCTTCTTGCCCTACTGAT
ATCTCTGATTTTCTTCAAAACCTTC
>EIF-2B_exon450_0
ACCTAGCTCATTAAAGCAGATTGAATCAAGCTGCACCCTTTCATGAAATTTATATGCTTCACAGCAGACCAAAAC
CGGAACATGAAATGCATGAGCAACC
>EIF-2B_exon450_8
CATTAAAGCAGATTGAATCAAGCTGCACCCTTTCATGAAATTTATATGCTTCACAGCAGACCAAAACCGGAACAT
GAAATGCATGAGCAACCATAGCAAC
>EIF-2B_exon450_24
ATCAAGCTGCACCCTTTCATGAAATTTATATGCTTCACAGCAGACCAAAACCGGAACATGAAATGCATGAGCAAC
CATAGCAACAGAAGCAGTTCCAACC
```

>EIF-2B_exon950_0
TTTTGCTTCTGATTCAGAGAGAGTTAGTGGTAACCTGGCAATGCGACTCTTCAGAAACCTGATTGCATTTCCCAT
GCTCATAGAAAGTGGGCGGCATTCT
>EIF-2B_exon950_23
TTAGTGGTAACCTGGCAATGCGACTCTTCAGAAACCTGATTGCATTTCCCATGCTCATAGAAAGTGGGCGGCATT
CTATCAGAAAAGAAACAAAACCACC
>EIF-2B_exon950_45
ACTCTTCAGAAACCTGATTGCATTTCCCATGCTCATAGAAAGTGGGCGGCATTCTATCAGAAAAGAAACAAAACC
ACCAATTTTGGAAGTTAAATCTCTG
>EIF-2B_exon1850_0
CATTTTTGTGTGGCGGCAGATGGCGAAAGAGTTCAACTCTATTCCTAGCTTCAGGTTGATTCAACAGAGATCTCC
TTTTAGCCTTACCCACACGACTCTC
>EIF-2B_exon1850_26
AAGAGTTCAACTCTATTCCTAGCTTCAGGTTGATTCAACAGAGATCTCCTTTTAGCCTTACCCACACGACTCTCA
TCGTCAAATTGCATACGTGGAGGAG
>EIF-2B_exon1850_51
CAGGTTGATTCAACAGAGATCTCCTTTTAGCCTTACCCACACGACTCTCATCGTCAAATTGCATACGTGGAGGAG
GAAGATCTTTCTTCCTCTCTTTCTC
>elong_exon500_0
GTAGTGGCGGTTATGTCGAAGCCGCATCGATGCCCGCATATTGCAACAACGGGGAATATTTGTGTTTATTGCCCA
GGTGGACCGGACTCGGATTTTGAGT
>elong_exon500_29
ATGCCCGCATATTGCAACAACGGGGAATATTTGTGTTTATTGCCCAGGTGGACCGGACTCGGATTTTGAGTATAG
TACGCAGTCATACACGGGGTATGAG
>elong_exon500_58
TTTGTGTTTATTGCCCAGGTGGACCGGACTCGGATTTTGAGTATAGTACGCAGTCATACACGGGGTATGAGCCAA
CTAGCATGCGAGCAATTCGAGCCAG
>elong_exon1600_0
GTTGAATTCATCTTGATGGGTGGTACTTTCATGTCACTGCCGGCAGAGTACCGAGACTATTTTATTAGAAATCTT
CATGATGCTTTATCAGGACACACTT
>elong_exon1600_38
GCCGGCAGAGTACCGAGACTATTTTATTAGAAATCTTCATGATGCTTTATCAGGACACACTTCTGCGAATGTTGA
AGAGGCAGTTGCCTACTCTGAGCAC
>elong_exon1600_73
TTCATGATGCTTTATCAGGACACACTTCTGCGAATGTTGAAGAGGCAGTTGCCTACTCTGAGCACAGTGCTGTAA
AGTGCATTGGAATGACAATTGAAAC
>elong_exon2200_0
TGCGCCAAATGCTTTCTTATGGTTGTACACGACTGGAGATTGGAGTTCAAAGCACATATGAGGATGTTGCTCGTG
ATACTAATAGAGGGCACACCGTTGC
>elong_exon2200_23
TGTACACGACTGGAGATTGGAGTTCAAAGCACATATGAGGATGTTGCTCGTGATACTAATAGAGGGCACACCGTT
GCTGCTGTAGCTGATTGTTTTTGCT
>elong_exon2200_46
TCAAAGCACATATGAGGATGTTGCTCGTGATACTAATAGAGGGCACACCGTTGCTGCTGTAGCTGATTGTTTTTG
CTTGGCAAAGGATGCTGGTTTTAAG
>elong_exon2700_0
GTTGTTGCTCATATGATGCCTGATCTTCCTAATGTTGGTGTTGAGAGAGACTTGGAAAGTTTCAAGGAATTTTTT
GAGAGCCCTTTATTTCGAGCTGATG
>elong_exon2700_22
ATCTTCCTAATGTTGGTGTTGAGAGAGACTTGGAAAGTTTCAAGGAATTTTTTGAGAGCCCTTTATTTCGAGCTG
ATGGGCTTAAAATTTACCCGACCCT
>elong_exon2700_43
AGAGAGACTTGGAAAGTTTCAAGGAATTTTTTGAGAGCCCTTTATTTCGAGCTGATGGGCTTAAAATTTACCCGA
CCCTTGTAATCCGTGGAACTGGCCT
>elong_exon3050_0
GCCATGGTACCTCCTTGGACACGTGTTTATAGGGTTCAGCGTGATATTCCGATGCCTCTGGTAACTTCAGGTGTT
GAGAAAGGAAATCTTCGTGAACTAG

>elong_exon3050_19
CACGTGTTTATAGGGTTCAGCGTGATATTCCGATGCCTCTGGTAACTTCAGGTGTTGAGAAAGGAAATCTTCGTG
AACTAGCTTTAGCTCGTATGGATGA
>elong_exon3050_37
AGCGTGATATTCCGATGCCTCTGGTAACTTCAGGTGTTGAGAAAGGAAATCTTCGTGAACTAGCTTTAGCTCGTA
TGGATGACTTGGGCTTGAAGTGTCG
>elong_exon3300_0
ATGGATACAGGACATTCACCACAAAATTAAACCTGAAGAAGTTGAGCTCGTTCGGCGAGATTATACAGCAAATGA
AGGTTGGGAAACATTTCTGTCATAC
>elong_exon3300_9
GGACATTCACCACAAAATTAAACCTGAAGAAGTTGAGCTCGTTCGGCGAGATTATACAGCAAATGAAGGTTGGGA
AACATTTCTGTCATACGAAGATACA
>elong_exon3300_25
ATTAAACCTGAAGAAGTTGAGCTCGTTCGGCGAGATTATACAGCAAATGAAGGTTGGGAAACATTTCTGTCATAC
GAAGATACACGTCAGGTACTGCAAG
>elong_exon3700_0
GATATTCTTGTTGGGTTGTTGCGACTGCGAAAATGTGGCCGGAATACTACTTGCCCTGAGCTAATGGGAAAATGT
TCTATTGTCCGTGAACTCCATGTAT
>elong_exon3700_9
GTTGGGTTGTTGCGACTGCGAAAATGTGGCCGGAATACTACTTGCCCTGAGCTAATGGGAAAATGTTCTATTGTC
CGTGAACTCCATGTATATGGAACTG
>elong_exon3700_25
TGCGAAAATGTGGCCGGAATACTACTTGCCCTGAGCTAATGGGAAAATGTTCTATTGTCCGTGAACTCCATGTAT
ATGGAACTGCTGTTCCAGTTCATGG
>elong_exon4000_0
TTGATGGAAGAAGCGGAGCGAATAGCAGGAAGGGAGCATAGATCGAAGAAAATAGCAGTGATATCGGGGGTGGGA
CCCCGCCATTATTATAGGAAATTGG
>elong_exon4000_21
ATAGCAGGAAGGGAGCATAGATCGAAGAAAATAGCAGTGATATCGGGGGTGGGACCCCGCCATTATTATAGGAAA
TTGGGGTATGAGCTTGAAGGGCCTT
>elong_exon4000_42
TCGAAGAAAATAGCAGTGATATCGGGGGTGGGACCCCGCCATTATTATAGGAAATTGGGGTATGAGCTTGAAGGG
CCTTACATGGTGAAATCTCTTGTTT
>F5H_exon408_519_0
CCATCAAATATCTAACGTACGACCGAGCGGACATGGCGTTTGCTCATTACGGACCCTTCTGGAGACAGATGAGGA
AGATTTGCGTGATGAAGGTTTTCAG
>F5H_exon408_519_6
AATATCTAACGTACGACCGAGCGGACATGGCGTTTGCTCATTACGGACCCTTCTGGAGACAGATGAGGAAGATTT
GCGTGATGAAGGTTTTCAGTCGGAA
>F5H_exon408_519_12
TAACGTACGACCGAGCGGACATGGCGTTTGCTCATTACGGACCCTTCTGGAGACAGATGAGGAAGATTTGCGTGA
TGAAGGTTTTCAGTCGGAAAAGAGC
>F5H_exon800_0
ACATGGGTCGATCCTCAAGGGCTCAACACCAGGCTGAAGAACGCTCGTGGTGCATTGGACAAGTTCATCGACACC
ATCATCGACGAACACATCCAAAAGA
>F5H_exon800_8
CGATCCTCAAGGGCTCAACACCAGGCTGAAGAACGCTCGTGGTGCATTGGACAAGTTCATCGACACCATCATCGA
CGAACACATCCAAAAGAGGAAGAGA
>F5H_exon800_16
AAGGGCTCAACACCAGGCTGAAGAACGCTCGTGGTGCATTGGACAAGTTCATCGACACCATCATCGACGAACACA
TCCAAAAGAGGAAGAGAAACGTCGA
>F5H_exon1509_1658_0
GGATGTGATGTTCGGCGGGACGGAGACGGTGGCATCGGCGATTGAGTGGGCCTTGTCGGAGCTGATGAAAAGTCC
GGAGGATATGAAGAGAGTCCAGCAG
>F5H_exon1509_1658_25
ACGGTGGCATCGGCGATTGAGTGGGCCTTGTCGGAGCTGATGAAAAGTCCGGAGGATATGAAGAGAGTCCAGCAG
GAGTTGGCGGAGGTGGTGGGTCTCG

>F5H_exon1509_1658_50
CCTTGTCGGAGCTGATGAAAAGTCCGGAGGATATGAAGAGAGTCCAGCAGGAGTTGGCGGAGGTGGTGGGTCTCG
ATCGCCGTGTGGAAGAATCCGATAT
>F5H_exon1848_1979_0
TTTCAAGCCATCGAGATTCCTGAAAGAAGGTGTAGCGGACTTTAAAGGGAGCAACTTCGAGTTCATCCCGTTCGG
GTCGGGTAGGAGGTCGTGTCCGGGT
>F5H_exon1848_1979_16
TTCCTGAAAGAAGGTGTAGCGGACTTTAAAGGGAGCAACTTCGAGTTCATCCCGTTCGGGTCGGGTAGGAGGTCG
TGTCCGGGTATGGGACTGGGGTTAT
>F5H_exon1848_1979_32
TAGCGGACTTTAAAGGGAGCAACTTCGAGTTCATCCCGTTCGGGTCGGGTAGGAGGTCGTGTCCGGGTATGGGAC
TGGGGTTATACGCGCTTGATTTGGC
>Formin2_exon50_0
CAGTAAGAATGGAGTCAAGCTCAAGAAGGGACTTTCATGGTGAGAACTTTGGCAGCCGAAGCTTTAACTCAAGAA
CAGCTTCTTACCCTTTATCTAACTC
>Formin2_exon50_15
CAAGCTCAAGAAGGGACTTTCATGGTGAGAACTTTGGCAGCCGAAGCTTTAACTCAAGAACAGCTTCTTACCCTT
TATCTAACTCTTGTTCACCTACAAA
>Formin2_exon50_30
ACTTTCATGGTGAGAACTTTGGCAGCCGAAGCTTTAACTCAAGAACAGCTTCTTACCCTTTATCTAACTCTTGTT
CACCTACAAATTCATTCTTGAACTC
>Formin2_exon500_0
GATGGCTTAGAGAAAAGTGAAGAGACCCCAAAACCGAAACTGAAGCCTTTGCATTGGGATAAAGTTCGGGCGAGT
TCGGATCGGGCCATGGTGTGGGATC
>Formin2_exon500_11
GAAAAGTGAAGAGACCCCAAAACCGAAACTGAAGCCTTTGCATTGGGATAAAGTTCGGGCGAGTTCGGATCGGGC
CATGGTGTGGGATCAGATAAAAGCA
>Formin2_exon500_22
AGACCCCAAAACCGAAACTGAAGCCTTTGCATTGGGATAAAGTTCGGGCGAGTTCGGATCGGGCCATGGTGTGGG
ATCAGATAAAAGCAAGCTCTTTTCA
>Formin2_exon900_0
GCCGATTCTGTCCGCTGTGAATCAGGAAAATCATGTGCTTGATCCAAAGAAGTCTCAGAACATTGCCATTTTATT
GAGGGCACTAAATGTGACCATTGAT
>Formin2_exon900_8
TGTCCGCTGTGAATCAGGAAAATCATGTGCTTGATCCAAAGAAGTCTCAGAACATTGCCATTTTATTGAGGGCAC
TAAATGTGACCATTGATGAAGTCTG
>Formin2_exon900_23
AGGAAAATCATGTGCTTGATCCAAAGAAGTCTCAGAACATTGCCATTTTATTGAGGGCACTAAATGTGACCATTG
ATGAAGTCTGCGAAGCACTTATGGA
>Formin2_exon1700_0
GCAATTCGGACACTCTTGGGACTGAGCTCTTGGAAAGCCTGTTAAAGATGGCTCCAACCAACGAAGAAGAGCGTA
AACTGAAGGGGTTCAATGATGAATC
>Formin2_exon1700_10
CACTCTTGGGACTGAGCTCTTGGAAAGCCTGTTAAAGATGGCTCCAACCAACGAAGAAGAGCGTAAACTGAAGGG
GTTCAATGATGAATCCCCTTTTAAG
>Formin2_exon1700_20
ACTGAGCTCTTGGAAAGCCTGTTAAAGATGGCTCCAACCAACGAAGAAGAGCGTAAACTGAAGGGGTTCAATGAT
GAATCCCCTTTTAAGCTAGGTCCAG
>Formin2_exon2170_0
GCTGCTTGTGGAGAGTTGAAAAACAGTAGAATGTTTCTGAAGCTTTTGGAGGCAGTGCTTAAAACTGGGAACCGC
ATGAACGTCGGCACCAACCGTGGCG
>Formin2_exon2170_9
GGAGAGTTGAAAAACAGTAGAATGTTTCTGAAGCTTTTGGAGGCAGTGCTTAAAACTGGGAACCGCATGAACGTC
GGCACCAACCGTGGCGATGCCCATG
>Formin2_exon2170_25
GTAGAATGTTTCTGAAGCTTTTGGAGGCAGTGCTTAAAACTGGGAACCGCATGAACGTCGGCACCAACCGTGGCG
ATGCCCATGCCTTCAAGCTTGACAC

>Formin2_exon2540_0
AAGGCTGCTGCTTTGGATTCCGATGTCCTCAGCATTGATGTTGCAAAACTTGCTACCGGTATCTCTAAAATCAGA
GAAGTTATAAAGCTGAATGAAGGGA
>Formin2_exon2540_10
CTTTGGATTCCGATGTCCTCAGCATTGATGTTGCAAAACTTGCTACCGGTATCTCTAAAATCAGAGAAGTTATAA
AGCTGAATGAAGGGATTGCTCTGAA
>Formin2_exon2540_20
CGATGTCCTCAGCATTGATGTTGCAAAACTTGCTACCGGTATCTCTAAAATCAGAGAAGTTATAAAGCTGAATGA
AGGGATTGCTCTGAAAGACAGTAGC
>glutamine_exon70_0
CCTTGGAGAGATAGACCAATTGCTGAGTCATTAAAACTCTTTGATGACATGAAGAGAGGTCTGATAGAAGAGGGC
AAAGCAACACTTAGGATGAAACAGG
>glutamine_exon70_26
GTCATTAAAACTCTTTGATGACATGAAGAGAGGTCTGATAGAAGAGGGCAAAGCAACACTTAGGATGAAACAGGA
TATGCAGAGTGATAACTTTAATATG
>glutamine_exon70_51
AAGAGAGGTCTGATAGAAGAGGGCAAAGCAACACTTAGGATGAAACAGGATATGCAGAGTGATAACTTTAATATG
TATGACCTTATTGCTTATCGTATCA
>glutamine_exon290_0
CTCTATTTCCAGTTTACTCCTCATCCGCATGCTGGTGACAAGTGGTGCATTTATCCAAGCTATGATTATGCTCAC
TGCATCGTGGACTCTCTTGAGAATA
>glutamine_exon290_10
AGTTTACTCCTCATCCGCATGCTGGTGACAAGTGGTGCATTTATCCAAGCTATGATTATGCTCACTGCATCGTGG
ACTCTCTTGAGAATATCACACATTC
>glutamine_exon290_20
TCATCCGCATGCTGGTGACAAGTGGTGCATTTATCCAAGCTATGATTATGCTCACTGCATCGTGGACTCTCTTGA
GAATATCACACATTCAGTATGTTTT
>glutamine_exon850_0
TGTACACTTGAATTTGAGACAAGGCGTGCTTCATACTATTGGCTGTTACATGTACTGGACCTTTCCCAGCCATAT
GTGTGGGAATACTCACGACTGAATG
>glutamine_exon850_14
TGAGACAAGGCGTGCTTCATACTATTGGCTGTTACATGTACTGGACCTTTCCCAGCCATATGTGTGGGAATACTC
ACGACTGAATGTTACAAACACTGTG
>glutamine_exon850_27
GCTTCATACTATTGGCTGTTACATGTACTGGACCTTTCCCAGCCATATGTGTGGGAATACTCACGACTGAATGTT
ACAAACACTGTGATGTCTAAACGGA
>glutamine_exon1070_0
AGTTAAACTACATTGTGACGAACAAATATGTTGATGGTTGGGATGATCCCCGTCTCATGACATTAGCTGGTTTAC
GGCGTAGGGGCGTGACTTCAACTGC
>glutamine_exon1070_19
GAACAAATATGTTGATGGTTGGGATGATCCCCGTCTCATGACATTAGCTGGTTTACGGCGTAGGGGCGTGACTTC
AACTGCGATAAATTCTTTTGTTCGA
>glutamine_exon1070_37
TTGGGATGATCCCCGTCTCATGACATTAGCTGGTTTACGGCGTAGGGGCGTGACTTCAACTGCGATAAATTCTTT
TGTTCGAGGAATTGGAATCACTAGA
>glutamine_exon1320_0
CTTGTGCCGCAGTGATTGTAGTATGATTCGAGTGGATCGCCTTGAGTATCACATAAGAGAAGAATTGAACAAGAC
AGCTCCTCGTGTATTGGTTGTGCTG
>glutamine_exon1320_10
AGTGATTGTAGTATGATTCGAGTGGATCGCCTTGAGTATCACATAAGAGAAGAATTGAACAAGACAGCTCCTCGT
GTATTGGTTGTGCTGCATCCTTTGA
>glutamine_exon1320_20
GTATGATTCGAGTGGATCGCCTTGAGTATCACATAAGAGAAGAATTGAACAAGACAGCTCCTCGTGTATTGGTTG
TGCTGCATCCTTTGAAGGTCGTCAT
>glutamine_exon1800_0
TCTAATTTTAGGTGCCCTTTTCGAATGTTGTGTATATTGAGCGCTCAGATTTCCGGATGAAAGATTCTAAAGATT
ACTATGGATTGGCTCCTGGCAAGTC

>glutamine_exon1800_10
GGTGCCCTTTTCGAATGTTGTGTATATTGAGCGCTCAGATTTCCGGATGAAAGATTCTAAAGATTACTATGGATT
GGCTCCTGGCAAGTCAGCACTGCTA
>glutamine_exon1800_20
TCGAATGTTGTGTATATTGAGCGCTCAGATTTCCGGATGAAAGATTCTAAAGATTACTATGGATTGGCTCCTGGC
AAGTCAGCACTGCTAAGGTATGCAT
>glutamine_exon2000_0
TTGCCGATATGCATTCCCCATTAAGTGTACAGACGTAATATTGGCAGATGATAAAGAGACCTTGCTTGAGATTCG
AGCCGAATATGATCCTTCTAAGAAA
>glutamine_exon2000_10
GCATTCCCCATTAAGTGTACAGACGTAATATTGGCAGATGATAAAGAGACCTTGCTTGAGATTCGAGCCGAATAT
GATCCTTCTAAGAAAAGCAAGCCAA
>glutamine_exon2000_19
ATTAAGTGTACAGACGTAATATTGGCAGATGATAAAGAGACCTTGCTTGAGATTCGAGCCGAATATGATCCTTCT
AAGAAAAGCAAGCCAAAGGTTCTTA
>glutamine_exon2450_0
AGAATCCTGCTGAACTTGATGATTGGCTTGCTGATCTCAACCCGAATTCCAAAGTGGTGGGACCTGCTGCATATG
CAGTACCATCACTTCGGAATGCTGC
>glutamine_exon2450_11
GAACTTGATGATTGGCTTGCTGATCTCAACCCGAATTCCAAAGTGGTGGGACCTGCTGCATATGCAGTACCATCA
CTTCGGAATGCTGCCATAGGGGACA
>glutamine_exon2450_21
ATTGGCTTGCTGATCTCAACCCGAATTCCAAAGTGGTGGGACCTGCTGCATATGCAGTACCATCACTTCGGAATG
CTGCCATAGGGGACACATTTCAGTT
>GPDH_exon600_0
ATGCTAGCTGCACCACCAACTGTCTTGCTCCATTGGCTAAGGTGATCAACGATAGATTTGGAATTGTTGAGGGTC
TTATGACCACTGTCCATTCGATAAC
>GPDH_exon600_10
CACCACCAACTGTCTTGCTCCATTGGCTAAGGTGATCAACGATAGATTTGGAATTGTTGAGGGTCTTATGACCAC
TGTCCATTCGATAACTGGTAAGTTC
>GPDH_exon600_20
TGTCTTGCTCCATTGGCTAAGGTGATCAACGATAGATTTGGAATTGTTGAGGGTCTTATGACCACTGTCCATTCG
ATAACTGGTAAGTTCTTGAGCTGTC
>GPDH_exon800_0
CCTTATTCAGCTACACAAAAGACTGTTGATGGTCCGTCAATGAAGGACTGGAGAGGTGGTAGAGCTGCTTCCTTC
AATATCATTCCTAGCAGCACTGGTG
>GPDH_exon800_10
CTACACAAAAGACTGTTGATGGTCCGTCAATGAAGGACTGGAGAGGTGGTAGAGCTGCTTCCTTCAATATCATTC
CTAGCAGCACTGGTGCGGCCAAGGT
>GPDH_exon800_20
GACTGTTGATGGTCCGTCAATGAAGGACTGGAGAGGTGGTAGAGCTGCTTCCTTCAATATCATTCCTAGCAGCAC
TGGTGCGGCCAAGGTATTATATTAT
>GPDH_exon1000_0
GCTGTTGGAAAAGTGTTGCCAGCATTGAATGGCAAGCTGACTGGAATGGCCTTCCGTGTTCCCACTGTTGATGTC
TCTGTGGTTGACCTCACTGTGAGAC
>GPDH_exon1000_22
CATTGAATGGCAAGCTGACTGGAATGGCCTTCCGTGTTCCCACTGTTGATGTCTCTGTGGTTGACCTCACTGTGA
GACTTGAGAAGAAGGCTACCTATGA
>GPDH_exon1000_43
GAATGGCCTTCCGTGTTCCCACTGTTGATGTCTCTGTGGTTGACCTCACTGTGAGACTTGAGAAGAAGGCTACCT
ATGAAGATATTAAGGCTGCTATCAA
>GPDH_exon1200_0
CCCTTTTTCTGTGCATAGAGCGGAATCTGAAACCAACTTGAAGGGAATTCTTGGTTATGTGGAGGAAGATTTGGT
GTCAACTGACTTTGTTGGAGACAGC
>GPDH_exon1200_10
GTGCATAGAGCGGAATCTGAAACCAACTTGAAGGGAATTCTTGGTTATGTGGAGGAAGATTTGGTGTCAACTGAC
TTTGTTGGAGACAGCAGGTATGGTT

>GPDH_exon1200_20
CGGAATCTGAAACCAACTTGAAGGGAATTCTTGGTTATGTGGAGGAAGATTTGGTGTCAACTGACTTTGTTGGAG
ACAGCAGGTATGGTTTTGTTAATGC
>GPDH_exon1500_0
GATGATGAAATTTGAACTTGTGTAATTTATTTTCAGGTCGAGCATTTTTGATGCCAAGGCTGGAATTGCTTTGAA
TGACAACTTTGTTAAGCTTGTGACT
>GPDH_exon1500_10
TTTGAACTTGTGTAATTTATTTTCAGGTCGAGCATTTTTGATGCCAAGGCTGGAATTGCTTTGAATGACAACTTT
GTTAAGCTTGTGACTTGGTATGACA
>GPDH_exon1500_20
TGTAATTTATTTTCAGGTCGAGCATTTTTGATGCCAAGGCTGGAATTGCTTTGAATGACAACTTTGTTAAGCTTG
TGACTTGGTATGACAACGAATGGGG
>GRIP_exon1400_0
TGCAGGTGCTTTTGGCAGAGAAAGAGTCTAAAATTGCTGAGATCGATGCAGCTTCGACTGGTGAAGCTGCACGAT
TAAGAGCTGCTGTGGAATCTATTAG
>GRIP_exon1400_11
TTGGCAGAGAAAGAGTCTAAAATTGCTGAGATCGATGCAGCTTCGACTGGTGAAGCTGCACGATTAAGAGCTGCT
GTGGAATCTATTAGAGGAGAGCTTA
>GRIP_exon1400_22
AGAGTCTAAAATTGCTGAGATCGATGCAGCTTCGACTGGTGAAGCTGCACGATTAAGAGCTGCTGTGGAATCTAT
TAGAGGAGAGCTTACACATGTGAAA
>GRIP_exon1600_0
GAGAAGGAAAAGGAGAGCTGGGAAGCTGCCTCGCAGGCATTCAAAACAAAACTGGAAGTTGCTGAGAGTAGCTGC
ATTCGTGCTGAAATAGAAGCTGCTA
>GRIP_exon1600_10
AGGAGAGCTGGGAAGCTGCCTCGCAGGCATTCAAAACAAAACTGGAAGTTGCTGAGAGTAGCTGCATTCGTGCTG
AAATAGAAGCTGCTAAAATGAGAAG
>GRIP_exon1600_20
GGAAGCTGCCTCGCAGGCATTCAAAACAAAACTGGAAGTTGCTGAGAGTAGCTGCATTCGTGCTGAAATAGAAGC
TGCTAAAATGAGAAGTATAGAAATT
>GRIP_exon2000_0
AGATTAGCCGTCTTGAAAGGGAATTTTCCTCTTATAAAATTCGTGCACATGCACTACTCCAGAAAAAGGATGCAG
AACTAGCAGCAGCTAAAGAGTCTGA
>GRIP_exon2000_16
AAGGGAATTTTCCTCTTATAAAATTCGTGCACATGCACTACTCCAGAAAAAGGATGCAGAACTAGCAGCAGCTAA
AGAGTCTGAACAAACAAAAGCGCTT
>GRIP_exon2000_31
TTATAAAATTCGTGCACATGCACTACTCCAGAAAAAGGATGCAGAACTAGCAGCAGCTAAAGAGTCTGAACAAAC
AAAAGCGCTTGAAGAAGCTTTAAAA
>GRIP_exon2500_0
AGAGATGCAGCACTTGACAATGCCAAACAGCAGATCAAAAGTTTAGAGACCAATCTTCATTCTGCTAATGCTCGC
CACCAATCAGAGAAAGCTGCATGGG
>GRIP_exon2500_18
AATGCCAAACAGCAGATCAAAAGTTTAGAGACCAATCTTCATTCTGCTAATGCTCGCCACCAATCAGAGAAAGCT
GCATGGGAAATGGACCTTAAAAATT
>GRIP_exon2500_36
AAAAGTTTAGAGACCAATCTTCATTCTGCTAATGCTCGCCACCAATCAGAGAAAGCTGCATGGGAAATGGACCTT
AAAAATTTGGAAGAAACTTGGCGAT
>GRIP_exon3000_0
AGGAGGAGCATGCATCATTACGAAATCTTGCTGATAGAATGATTGAGGAGAAGGATAATGAAATTTCTAGACTTT
TGGATGATAACAAGAATCTTCAACG
>GRIP_exon3000_11
GCATCATTACGAAATCTTGCTGATAGAATGATTGAGGAGAAGGATAATGAAATTTCTAGACTTTTGGATGATAAC
AAGAATCTTCAACGATCTCTAGAGT
>GRIP_exon3000_21
GAAATCTTGCTGATAGAATGATTGAGGAGAAGGATAATGAAATTTCTAGACTTTTGGATGATAACAAGAATCTTC
AACGATCTCTAGAGTCGAGACAACT

>HIPL1_exon430_0
GGAGCACCCGTGAATTCGAAATTCACCAAGTTGACCGAACTTTGGCAGTCGAAAACAGAATTCTGCAATGTCTTT
GGTGGAGCATCTACTGCTGGGTCAG
>HIPL1_exon430_11
GAATTCGAAATTCACCAAGTTGACCGAACTTTGGCAGTCGAAAACAGAATTCTGCAATGTCTTTGGTGGAGCATC
TACTGCTGGGTCAGTTTGCTATGAC
>HIPL1_exon430_22
TCACCAAGTTGACCGAACTTTGGCAGTCGAAAACAGAATTCTGCAATGTCTTTGGTGGAGCATCTACTGCTGGGT
CAGTTTGCTATGACGGTGAACCTGT
>HIPL1_exon820_0
ATTGCATTTCATCCAAACTTTGCACAAAATGGCAGATTCTTTGCTTCATTCAATTGTGACAAGGGTAAATCACCA
GGATGTACCGGAAGATGTTCATGTA
>HIPL1_exon820_16
ACTTTGCACAAAATGGCAGATTCTTTGCTTCATTCAATTGTGACAAGGGTAAATCACCAGGATGTACCGGAAGAT
GTTCATGTAATTCGGATGTGAACTG
>HIPL1_exon820_31
GCAGATTCTTTGCTTCATTCAATTGTGACAAGGGTAAATCACCAGGATGTACCGGAAGATGTTCATGTAATTCGG
ATGTGAACTGTGATCCCTCAAAACT
>HIPL1_exon1100_0
GGCAGAAAATGCTAAACCATCAGAAGTGAGAAGGATATTCACTATGGGCCTTCCCTTTACTTCTCAACATGGTGG
ACAGATACTTTTTGGACCTACAGAT
>HIPL1_exon1100_8
ATGCTAAACCATCAGAAGTGAGAAGGATATTCACTATGGGCCTTCCCTTTACTTCTCAACATGGTGGACAGATAC
TTTTTGGACCTACAGATGGTTATCT
>HIPL1_exon1100_23
AAGTGAGAAGGATATTCACTATGGGCCTTCCCTTTACTTCTCAACATGGTGGACAGATACTTTTTGGACCTACAG
ATGGTTATCTGTACTTTATGATGGG
>HIPL1_exon1550_0
AATCCATTTAGACAAGATGAAGAGTTGCTGCCTGAAATATGGGCTTTTGGATTAAGGAATCCTTGGCGATGCAGT
TTCGATTCAGAGAGGCCTTCGTATT
>HIPL1_exon1550_11
ACAAGATGAAGAGTTGCTGCCTGAAATATGGGCTTTTGGATTAAGGAATCCTTGGCGATGCAGTTTCGATTCAGA
GAGGCCTTCGTATTTTATGTGCGGG
>HIPL1_exon1550_21
GAGTTGCTGCCTGAAATATGGGCTTTTGGATTAAGGAATCCTTGGCGATGCAGTTTCGATTCAGAGAGGCCTTCG
TATTTTATGTGCGGGGATGTTGGCG
>HIPL1_exon1970_0
AATTCCATAAGCCCCATTCTCCCTGTTATGGGATACAATCACTCTGAAGTAAACAAGAAAATAGGATCAGCCTCG
ATCATAGGTGGCCATTTTTACCGGT
>HIPL1_exon1970_9
AGCCCCATTCTCCCTGTTATGGGATACAATCACTCTGAAGTAAACAAGAAAATAGGATCAGCCTCGATCATAGGT
GGCCATTTTTACCGGTCCAACACCG
>HIPL1_exon1970_26
TATGGGATACAATCACTCTGAAGTAAACAAGAAAATAGGATCAGCCTCGATCATAGGTGGCCATTTTTACCGGTC
CAACACCGATCCGTGTATGTACGGA
>HIPL1_exon2230_0
AGGTACTTGTATGCAGATTTGTATGCCGGTGCAATATGGTCAGCAACAGAAGACCCGGAAAACAGTGGCAACTTC
TCAACTAGCACTATTCCTTTTAGTT
>HIPL1_exon2230_20
GTATGCCGGTGCAATATGGTCAGCAACAGAAGACCCGGAAAACAGTGGCAACTTCTCAACTAGCACTATTCCTTT
TAGTTGTGCAAGAGACTCACCTCTA
>HIPL1_exon2230_39
TCAGCAACAGAAGACCCGGAAAACAGTGGCAACTTCTCAACTAGCACTATTCCTTTTAGTTGTGCAAGAGACTCA
CCTCTACAATGCAGCACCGTGCCAG
>Histidine1_exon560_0
TCAGAAGAAACCACTGTCTGCATTAAAGACAAAGTTGAAGCATCATGGAAACCATATGAAGAAGGCCAGCAAGAG
AGACAAGAAAATTGTTCTTTGGTTC

>Histidine1_exon560_11
CACTGTCTGCATTAAAGACAAAGTTGAAGCATCATGGAAACCATATGAAGAAGGCCAGCAAGAGAGACAAGAAAA
TTGTTCTTTGGTTCGAAGTTGATGA
>Histidine1_exon560_21
ATTAAAGACAAAGTTGAAGCATCATGGAAACCATATGAAGAAGGCCAGCAAGAGAGACAAGAAAATTGTTCTTTG
GTTCGAAGTTGATGACACCGGCAGT
>Histidine1_exon1240_0
GGTTAATAAGATGGGTGGAGAAATCAAGGTTGTAAAAAAGAATGGTCCAGGAACTCTAATAAGACTATTCTTGCT
ACTCACTACTCCTGCAGATGGCACA
>Histidine1_exon1240_21
AATCAAGGTTGTAAAAAAGAATGGTCCAGGAACTCTAATAAGACTATTCTTGCTACTCACTACTCCTGCAGATGG
CACAGAGCATCATGGTCTAATGGAT
>Histidine1_exon1240_41
ATGGTCCAGGAACTCTAATAAGACTATTCTTGCTACTCACTACTCCTGCAGATGGCACAGAGCATCATGGTCTAA
TGGATTTTGCAAAGCACAGTGTAGC
>Histidine1_exon1640_0
GGTGATCCTTGCCCTACACGGCAGCATGGGTAGATTGATTATGTCGAAGTGGCTGTCTAGAAATGGAGTGCCAGC
TTTGGAAGCATCCGAGTGGAATGAA
>Histidine1_exon1640_15
ACACGGCAGCATGGGTAGATTGATTATGTCGAAGTGGCTGTCTAGAAATGGAGTGCCAGCTTTGGAAGCATCCGA
GTGGAATGAACTGACACAGATCCTT
>Histidine1_exon1640_30
TAGATTGATTATGTCGAAGTGGCTGTCTAGAAATGGAGTGCCAGCTTTGGAAGCATCCGAGTGGAATGAACTGAC
ACAGATCCTTCATGAACTGTTTCAT
>Histidine1_exon2000_0
TAAAGATTGAGCTCCGCAGGAAGGGACATGTATTGATGGTTAATAGACCTTTGTACAAGGCAAAAATGCTTCATA
TTTTGGAAGCTGTCATAAAGGAGAG
>Histidine1_exon2000_8
GAGCTCCGCAGGAAGGGACATGTATTGATGGTTAATAGACCTTTGTACAAGGCAAAAATGCTTCATATTTTGGAA
GCTGTCATAAAGGAGAGACATGCTG
>Histidine1_exon2000_23
GGACATGTATTGATGGTTAATAGACCTTTGTACAAGGCAAAAATGCTTCATATTTTGGAAGCTGTCATAAAGGAG
AGACATGCTGAAATTCACAAGAGAA
>Histidine1_exon2540_0
CTACGGATACTGCTTGCTGAAGATACACCTGTTCTCCAAAGAGTAGCGACCATCATGCTGGAAAAAAATGGGAGCT
ACGGTAATTGCTGTTGGGGATGGAC
>Histidine1_exon2540_9
CTGCTTGCTGAAGATACACCTGTTCTCCAAAGAGTAGCGACCATCATGCTGGAAAAAAATGGGAGCTACGGTAATT
GCTGTTGGGGATGGACTGCAGGCAG
>Histidine1_exon2540_25
CACCTGTTCTCCAAAGAGTAGCGACCATCATGCTGGAAAAAAATGGGAGCTACGGTAATTGCTGTTGGGGATGGAC
TGCAGGCAGTAGACGCACTGAACTG
>Histidine1_exon2940_0
AAGATGGATGGATATGAAGCAACAAAAGCAATCAGGAAATCAGAAGCAGGGATGGGTTGGCACATTCCTATTGTT
GCCTTGACAGCCCATGCAATGTCAT
>Histidine1_exon2940_14
TGAAGCAACAAAAGCAATCAGGAAATCAGAAGCAGGGATGGGTTGGCACATTCCTATTGTTGCCTTGACAGCCCA
TGCAATGTCATCGGATGAGGCAAAA
>Histidine1_exon2940_28
CAATCAGGAAATCAGAAGCAGGGATGGGTTGGCACATTCCTATTGTTGCCTTGACAGCCCATGCAATGTCATCGG
ATGAGGCAAAATGCTTGGAGGTTGG
>Importin4_exon4050_0
GGAGCAAAAGCGCTTTTTGTATGCTGGGCAAATAAGCCAAGAGCTTGAGTTGCAGCTGCCTTTTCGTCCAATACA
CCAGTTCTTATACTGATATTTCTAA
>Importin4_exon4050_14
TTTTGTATGCTGGGCAAATAAGCCAAGAGCTTGAGTTGCAGCTGCCTTTTCGTCCAATACACCAGTTCTTATACT
GATATTTCTAACCCTTGGTTCATCA

>Importin4_exon4050_28
CAAATAAGCCAAGAGCTTGAGTTGCAGCTGCCTTTTCGTCCAATACACCAGTTCTTATACTGATATTTCTAACCC
TTGGTTCATCATGAGCTTCATCATC
>Importin4_exon4650_0
ACAAGCTCAGTGGCCCTTGCTCGGGCACGCAAGTCCTCATCATTAGTAAGAACCATAAATACTTTCATCAACTCC
AGAACCCTTTCAGCATATGGAATGA
>Importin4_exon4650_20
TCGGGCACGCAAGTCCTCATCATTAGTAAGAACCATAAATACTTTCATCAACTCCAGAACCCTTTCAGCATATGG
AATGAATGCTTGCTCTGCAGCAGCT
>Importin4_exon4650_40
CATTAGTAAGAACCATAAATACTTTCATCAACTCCAGAACCCTTTCAGCATATGGAATGAATGCTTGCTCTGCAG
CAGCTGCAACGGAACCAATTGCAGA
>Importin4_exon5000_0
CATGCATGTTTCTTGCAAATTACGTGAACTATTTTGGAGGGCAGCCAGTAGCTTTCCCATTAAAGGATCCAGAAA
AGGTAGGATTTCCATGCCCATATCT
>Importin4_exon5000_17
AATTACGTGAACTATTTTGGAGGGCAGCCAGTAGCTTTCCCATTAAAGGATCCAGAAAAGGTAGGATTTCCATGC
CCATATCTTCACAAAAGGCTGCCAA
>Importin4_exon5000_34
TGGAGGGCAGCCAGTAGCTTTCCCATTAAAGGATCCAGAAAAGGTAGGATTTCCATGCCCATATCTTCACAAAAG
GCTGCCAAAGCATAATATGACTTTT
>Importin4_exon5430_0
GGGTCCCTCAATGCTCCTAAAACAATCTGAAGAACAGGCTCCAACTTATCCTTCATCAGCTCTGCACAACCCTCT
GAAACAACACCTAAAGCTGTGACAG
>Importin4_exon5430_23
AATCTGAAGAACAGGCTCCAACTTATCCTTCATCAGCTCTGCACAACCCTCTGAAACAACACCTAAAGCTGTGAC
AGAAGCCTCCCTAAACTTTGGATTC
>Importin4_exon5430_45
TTATCCTTCATCAGCTCTGCACAACCCTCTGAAACAACACCTAAAGCTGTGACAGAAGCCTCCCTAAACTTTGGA
TTCGCATTCTGACTGCTTACAGAAG
>Kinesin-KCA2_exon2800_0
TTGGTTAAAGCAGGTTCAGATAAAGTTAAAACTACGCCTGCTGGAGAATACCTTACTGCTGCTCTGAATGACTTT
GATCCTGACCAATATGATGGCATTG
>Kinesin-KCA2_exon2800_19
ATAAAGTTAAAACTACGCCTGCTGGAGAATACCTTACTGCTGCTCTGAATGACTTTGATCCTGACCAATATGATG
GCATTGCTGCGATTTCAGATGGAGC
>Kinesin-KCA2_exon2800_37
CTGCTGGAGAATACCTTACTGCTGCTCTGAATGACTTTGATCCTGACCAATATGATGGCATTGCTGCGATTTCAG
ATGGAGCAAACAAGCTTTTGATGCT
>Kinesin-KCA2_exon3550_0
GCAGCTGTCATTAAAGCAGGTGCTTCTAGAGAGCATGAAATACTTGCTGAAATTAGAGATGCCGTTTTTGCTTTT
ATCCGGAAAATGGAACCAAAGAGAG
>Kinesin-KCA2_exon3550_14
AGCAGGTGCTTCTAGAGAGCATGAAATACTTGCTGAAATTAGAGATGCCGTTTTTGCTTTTATCCGGAAAATGGA
ACCAAAGAGAGTAATGGATACCATG
>Kinesin-KCA2_exon3550_28
GAGAGCATGAAATACTTGCTGAAATTAGAGATGCCGTTTTTGCTTTTATCCGGAAAATGGAACCAAAGAGAGTAA
TGGATACCATGCTTGTTTCCCGTGT
>Kinesin-KCA2_exon3800_0
CTTTCTAGAAAAGCCTAATTCAGGACGTAGTAGAAGTTCCAGCCGGAGTAACAGTCCTGGAAGATCACCTGTGCG
TTATGTTGATGAGCAGATCCAGGGT
>Kinesin-KCA2_exon3800_9
AAAGCCTAATTCAGGACGTAGTAGAAGTTCCAGCCGGAGTAACAGTCCTGGAAGATCACCTGTGCGTTATGTTGA
TGAGCAGATCCAGGGTTTTAAAGTA
>Kinesin-KCA2_exon3800_26
GTAGTAGAAGTTCCAGCCGGAGTAACAGTCCTGGAAGATCACCTGTGCGTTATGTTGATGAGCAGATCCAGGGTT
TTAAAGTAAATATAAAGCAAGAAAA

>Kinesin-KCA2_exon4400_0
GGGGATGAAGCATCTGGGGGAACCACTGGTCAGTTGGAGCTTCTTTCAACTGCTATTATGGATGGTTGGATGGCT
GGACTTGGTGCTGCACTCCCTCCTA
>Kinesin-KCA2_exon4400_22
CCACTGGTCAGTTGGAGCTTCTTTCAACTGCTATTATGGATGGTTGGATGGCTGGACTTGGTGCTGCACTCCCTC
CTAATACGGATGCTCTTGGTCAGCT
>Kinesin-KCA2_exon4400_43
TTTCAACTGCTATTATGGATGGTTGGATGGCTGGACTTGGTGCTGCACTCCCTCCTAATACGGATGCTCTTGGTC
AGCTTTTATCGGAGTATGCAAAGCG
>Kinesin-KP1_exon660_0
TCAGAAAGCAATGTGATTGCAGCATCGGTTTTCTGAAGTCTTGCAGGCGGTTGGTGCTTTGTTTTAGCCTTACTC
TCAACCTTGCTTTTCCTAATTCCAC
>Kinesin-KP1_exon660_10
ATGTGATTGCAGCATCGGTTTTCTGAAGTCTTGCAGGCGGTTGGTGCTTTGTTTTAGCCTTACTCTCAACCTTGC
TTTTCCTAATTCCACCCTGTCTAAC
>Kinesin-KP1_exon660_20
AGCATCGGTTTTCTGAAGTCTTGCAGGCGGTTGGTGCTTTGTTTTAGCCTTACTCTCAACCTTGCTTTTCCTAAT
TCCACCCTGTCTAACATTAATGGCT
>Kinesin-KP1_exon950_0
GTACTCTAGCCGGAAATGGTACTTTCGATACTGGCTGATTGTCAACTGTGTCAGCCTTGATCCTGCTTCTAATTA
AGGCTCCTCTATCAGTGGATTAGGG
>Kinesin-KP1_exon950_10
CGGAAATGGTACTTTCGATACTGGCTGATTGTCAACTGTGTCAGCCTTGATCCTGCTTCTAATTAAGGCTCCTCT
ATCAGTGGATTAGGGCCTTCGAACA
>Kinesin-KP1_exon950_20
ACTTTCGATACTGGCTGATTGTCAACTGTGTCAGCCTTGATCCTGCTTCTAATTAAGGCTCCTCTATCAGTGGAT
TAGGGCCTTCGAACAGGAGGAGACG
>Kinesin-KP1_exon1370_0
AATTTGGAAAGGTGAAACCGCTCTTCCTCGTTGAGTTTCAGTCGTGCTTCGAGCATTTCCACCTTTCAGTTGTTC
TAATTCTGCTTCCTTATTTTCCAAT
>Kinesin-KP1_exon1370_10
GGTGAAACCGCTCTTCCTCGTTGAGTTTCAGTCGTGCTTCGAGCATTTCCACCTTTCAGTTGTTCTAATTCTGCT
TCCTTATTTTCCAATGCAAGTTTAA
>Kinesin-KP1_exon1370_20
CTCTTCCTCGTTGAGTTTCAGTCGTGCTTCGAGCATTTCCACCTTTCAGTTGTTCTAATTCTGCTTCCTTATTTT
CCAATGCAAGTTTAAGATTTGATAT
>Kinesin-KP1_exon1600_0
ATTTCACCGGTTTCCTTGTTGGATCGAGCTGCCCCGAGTTCTATGGAAGCAACCCTCTCAGCAAATTTCAGTGTG
CTAACTGTTTCTCCAATGGAATTGA
>Kinesin-KP1_exon1600_10
TTTCCTTGTTGGATCGAGCTGCCCCGAGTTCTATGGAAGCAACCCTCTCAGCAAATTTCAGTGTGCTAACTGTTT
CTCCAATGGAATTGACTTCAGGACT
>Kinesin-KP1_exon1600_20
GGATCGAGCTGCCCCGAGTTCTATGGAAGCAACCCTCTCAGCAAATTTCAGTGTGCTAACTGTTTCTCCAATGGA
ATTGACTTCAGGACTTATATGTACA
>Kinesin-KP1_exon1830_0
ACCTAAAGAATCCTGTAATACTTGAGTAAGCTTGCTGTTTCTGTAAGGAATATGTGCACTCTTTTGTGCAAGTGC
TGCAATGACATCTCCTAGTGCCGAG
>Kinesin-KP1_exon1830_10
TCCTGTAATACTTGAGTAAGCTTGCTGTTTCTGTAAGGAATATGTGCACTCTTTTGTGCAAGTGCTGCAATGACA
TCTCCTAGTGCCGAGAGTGATTTAT
>Kinesin-KP1_exon1830_20
CTTGAGTAAGCTTGCTGTTTCTGTAAGGAATATGTGCACTCTTTTGTGCAAGTGCTGCAATGACATCTCCTAGTG
CCGAGAGTGATTTATTGATGTGTTG
>Kinesin-KP1_exon2100_0
TCAAAATACTGTGAGAACGGCTACTTCGCTCGTTTAAAGCCGTAGCACCTACAGCACGATTCTTTTGACCGATTC
TCATCAAGTCAAGAACATCTTGCGT

>Kinesin-KP1_exon2100_10
GTGAGAACGGCTACTTCGCTCGTTTAAAGCCGTAGCACCTACAGCACGATTCTTTTGACCGATTCTCATCAAGTC
AAGAACATCTTGCGTACTTGCAACC
>Kinesin-KP1_exon2100_20
CTACTTCGCTCGTTTAAAGCCGTAGCACCTACAGCACGATTCTTTTGACCGATTCTCATCAAGTCAAGAACATCT
TGCGTACTTGCAACCGGAACCCAAC
>Leukotriene_exon300_Pacbio_0
GTTTTCTCCACCTCACTGTAGAAATTTCTGCACTTGGATAGAATTGCTAGTTGAAGAAAAGCCACTTTTACCTCA
TAATCCTTTGATTCTGATAGTCTGT
>Leukotriene_exon300_Pacbio_10
CCTCACTGTAGAAATTTCTGCACTTGGATAGAATTGCTAGTTGAAGAAAAGCCACTTTTACCTCATAATCCTTTG
ATTCTGATAGTCTGTATCGTTCGTC
>Leukotriene_exon300_Pacbio_20
GAAATTTCTGCACTTGGATAGAATTGCTAGTTGAAGAAAAGCCACTTTTACCTCATAATCCTTTGATTCTGATAG
TCTGTATCGTTCGTCCAAGGCTAAG
>Leukotriene_exon1480_Pacbio_0
ACCTGAGAAGCCTCGAGAACTCTAGGCAGGTTCTCTAAGTAGAGCTCCCATTCCTGTCCACGCCAATCTGCAACT
TCATCCTCCCTCGGCATCCTCCCAA
>Leukotriene_exon1480_Pacbio_19
CTCTAGGCAGGTTCTCTAAGTAGAGCTCCCATTCCTGTCCACGCCAATCTGCAACTTCATCCTCCCTCGGCATCC
TCCCAAGTTTGAATTCATTTGCCAA
>Leukotriene_exon1480_Pacbio_37
AGTAGAGCTCCCATTCCTGTCCACGCCAATCTGCAACTTCATCCTCCCTCGGCATCCTCCCAAGTTTGAATTCAT
TTGCCAATGACAAAATTTTGGTGTA
>Leukotriene_exon2000_0
TGATTGTTTTTGAGCTTGGTGAATTCCAAATTATCCTTAAATTTCTCCACTTCCTCCTTTAAACCCTTCCAACCA
ATTCCAGTGTTCAGTATAGCTCTGT
>Leukotriene_exon2000_24
TCCAAATTATCCTTAAATTTCTCCACTTCCTCCTTTAAACCCTTCCAACCAATTCCAGTGTTCAGTATAGCTCTG
TCTTCACCCTGCACAGCCTCAACAA
>Leukotriene_exon2000_48
ACTTCCTCCTTTAAACCCTTCCAACCAATTCCAGTGTTCAGTATAGCTCTGTCTTCACCCTGCACAGCCTCAACA
ATTCTCCTTTCTGCATATGTGGTAA
>Leukotriene_exon2400_0
GGATTCTCCATTCCACCGTATGGGAAACTCGGCGGCAGAACAAGCAAATCGAATCTCTCCCAAGCATATTCCCCA
AACAACTTCTCCCCTTGCCTGATCA
>Leukotriene_exon2400_20
TGGGAAACTCGGCGGCAGAACAAGCAAATCGAATCTCTCCCAAGCATATTCCCCAAACAACTTCTCCCCTTGCCT
GATCATCTCCTCTGTCCCGGCGAAC
>Leukotriene_exon2400_40
CAAGCAAATCGAATCTCTCCCAAGCATATTCCCCAAACAACTTCTCCCCTTGCCTGATCATCTCCTCTGTCCCGG
CGAACTCCTTCGCCGCAGCATCCAA
>Leukotriene_exon3000_0
ATGGAAAGCTGGGTTCCTTTGATTGGGTCAGGGGAAGAGGAAAGAGAGAAAGGGAGCGGCGAAAGGTCGTTGGGA
GAGAGGACTTGGTGGATGGTAAGAG
>Leukotriene_exon3000_9
TGGGTTCCTTTGATTGGGTCAGGGGAAGAGGAAAGAGAGAAAGGGAGCGGCGAAAGGTCGTTGGGAGAGAGGACT
TGGTGGATGGTAAGAGAGCGGGTGT
>Leukotriene_exon3000_26
GTCAGGGGAAGAGGAAAGAGAGAAAGGGAGCGGCGAAAGGTCGTTGGGAGAGAGGACTTGGTGGATGGTAAGAGA
GCGGGTGTCGAAGGAAAGTTGACCG
>LOC105761646_exon50_0
CCTCATTCCAACACTCCTGAAGGGGCTACAGTTGCCCATCAAAAACCTATTGATTTCACCAAATTAGACATTAAT
CTGCTTCCCACGGTCATGATTATTG
>LOC105761646_exon50_18
GAAGGGGCTACAGTTGCCCATCAAAAACCTATTGATTTCACCAAATTAGACATTAATCTGCTTCCCACGGTCATG
ATTATTGGTCGCCCCAATGTAGGGA

>LOC105761646_exon50_36
CATCAAAAACCTATTGATTTCACCAAATTAGACATTAATCTGCTTCCCACGGTCATGATTATTGGTCGCCCCAAT
GTAGGGAAGTCTGCATTGTTTAACC
>LOC105761646_exon300_0
GCTTGATACGGAGGAGAGAGGCTCTTGTGTACAACACACCTGATGATCATGTCACTAGAGACATAAGGGAAGGGC
TTGCTAAATTGGGGGATTTGCGGTT
>LOC105761646_exon300_11
AGGAGAGAGGCTCTTGTGTACAACACACCTGATGATCATGTCACTAGAGACATAAGGGAAGGGCTTGCTAAATTG
GGGGATTTGCGGTTTACAGTATTGG
>LOC105761646_exon300_21
CTCTTGTGTACAACACACCTGATGATCATGTCACTAGAGACATAAGGGAAGGGCTTGCTAAATTGGGGGATTTGC
GGTTTACAGTATTGGATTCTGCTGG
>LOC105761646_exon1600_0
AGAACTGGGCTACATCCATTGGATCAGGAGGTTGGAAGGTGGTTACGCAAGCATGCACCAGGAATCACTCCTATA
GTAGCCATGAATAAATCTGAATCGC
>LOC105761646_exon1600_20
GGATCAGGAGGTTGGAAGGTGGTTACGCAAGCATGCACCAGGAATCACTCCTATAGTAGCCATGAATAAATCTGA
ATCGCTCCATAATGATCCAAACTCT
>LOC105761646_exon1600_40
GGTTACGCAAGCATGCACCAGGAATCACTCCTATAGTAGCCATGAATAAATCTGAATCGCTCCATAATGATCCAA
ACTCTTTTGCTGAAGCTGCTACAGA
>LOC105761646_exon2500_0
GGGAAGTCGACATTACTAAATGCATTGTTACAAGAAGATCGTGTTTTGGTTGGTCCGGAAGCTGGTTTGACTAGA
GATTCAGTGAGAGCTCAATTTCAAT
>LOC105761646_exon2500_11
ATTACTAAATGCATTGTTACAAGAAGATCGTGTTTTGGTTGGTCCGGAAGCTGGTTTGACTAGAGATTCAGTGAG
AGCTCAATTTCAATATGAAGGGAGA
>LOC105761646_exon2500_22
CATTGTTACAAGAAGATCGTGTTTTGGTTGGTCCGGAAGCTGGTTTGACTAGAGATTCAGTGAGAGCTCAATTTC
AATATGAAGGGAGAACGGTTTATCT
>LOC105761646_exon3000_0
GGTTGATACTGCTGGTTGGTTGCAAAGATGTGATCGGGATAAAGGTCCCGCTTCATTGAGCATCATGCAATCGAG
AAAAAATCTGATGAGAGCTCATGTT
>LOC105761646_exon3000_9
TGCTGGTTGGTTGCAAAGATGTGATCGGGATAAAGGTCCCGCTTCATTGAGCATCATGCAATCGAGAAAAAATCT
GATGAGAGCTCATGTTGTTGCTTTG
>LOC105761646_exon3000_25
AGATGTGATCGGGATAAAGGTCCCGCTTCATTGAGCATCATGCAATCGAGAAAAAATCTGATGAGAGCTCATGTT
GTTGCTTTGGTTCTTGATGCTGAAG
>LOC105761646_exon3700_0
AGGTTGCAAAAGCTTGGCGTAGCTTGACACATGCTGAAGTAGTTATAGCAAGGCAAGCTGTGGAAGAAGGTCGTG
GTCTGGTTGTAATTGTAAACAAGAT
>LOC105761646_exon3700_8
AAAGCTTGGCGTAGCTTGACACATGCTGAAGTAGTTATAGCAAGGCAAGCTGTGGAAGAAGGTCGTGGTCTGGTT
GTAATTGTAAACAAGATGGATCTTC
>LOC105761646_exon3700_24
TGACACATGCTGAAGTAGTTATAGCAAGGCAAGCTGTGGAAGAAGGTCGTGGTCTGGTTGTAATTGTAAACAAGA
TGGATCTTCTAAAGGGGAAAAGAAA
>LOC105761646_exon4000_0
CAGATAACAGGGATACCAGTTGTGTTCATCTCAGCAATAGACGGAAAAGGGCGTTCTGCTGTTATGTCTCAAGTC
ATTGATACGTACGAGAAGTGGTGTT
>LOC105761646_exon4000_17
AGTTGTGTTCATCTCAGCAATAGACGGAAAAGGGCGTTCTGCTGTTATGTCTCAAGTCATTGATACGTACGAGAA
GTGGTGTTTAAGATTGTCCACTGCA
>LOC105761646_exon4000_34
CAATAGACGGAAAAGGGCGTTCTGCTGTTATGTCTCAAGTCATTGATACGTACGAGAAGTGGTGTTTAAGATTGT
CCACTGCACGTCTTAACCGTTGGTT

>LOC105761646_exon4300_0
AGGTTACGAGCCGGCATTCTTGGAAAGACCAAGGTGCTCAAACCAAGATCAAGTACTTCACTCAAGTGAAAGCCA
GGCCTCCAACTTTTGTTGCCTTTGT
>LOC105761646_exon4300_33
GTGCTCAAACCAAGATCAAGTACTTCACTCAAGTGAAAGCCAGGCCTCCAACTTTTGTTGCCTTTGTGAGTGGGA
ACACCAAGCTCTCCGATACATATGT
>LOC105761646_exon4300_66
TGAAAGCCAGGCCTCCAACTTTTGTTGCCTTTGTGAGTGGGAACACCAAGCTCTCCGATACATATGTAAGGTTTC
TAACCAAATCTTTGAAGGAAGATTT
>LOC105762837_exon300_0
TTCAAGCAGCTGGCACTGAGGTCGAAAGGACCACGTATGACAAATTTTGGAGTCCATCCTGGATTTTGCTCACAG
CAATGGTCTGATCACCACGGAGATG
>LOC105762837_exon300_15
CTGAGGTCGAAAGGACCACGTATGACAAATTTTGGAGTCCATCCTGGATTTTGCTCACAGCAATGGTCTGATCAC
CACGGAGATGTTCAGAGAAGGAGCT
>LOC105762837_exon300_30
CCACGTATGACAAATTTTGGAGTCCATCCTGGATTTTGCTCACAGCAATGGTCTGATCACCACGGAGATGTTCAG
AGAAGGAGCTAGCTTTCTCCTGGAT
>LOC105762837_exon700_0
ATCTTCAGGCCAATCGATACTCATCATATCGCCATCAGCATCTTCATCTTGAACTTTGTTAGAACTAGATATGAC
AGATTTACCCAGCATCAGGAGATGA
>LOC105762837_exon700_16
ATACTCATCATATCGCCATCAGCATCTTCATCTTGAACTTTGTTAGAACTAGATATGACAGATTTACCCAGCATC
AGGAGATGAGACATCGCTAAACAGC
>LOC105762837_exon700_32
CATCAGCATCTTCATCTTGAACTTTGTTAGAACTAGATATGACAGATTTACCCAGCATCAGGAGATGAGACATCG
CTAAACAGCACATTTCTGAAAGCCT
>LOC105762837_exon900_0
AAAACATCTCAATACATGAACCCCCTCTGAGTGCAGTGACTCTAGCCTTCCAGTGGTTCTTTGAATTACATCATT
AACGGCCAATCCTGCCAATGCATTT
>LOC105762837_exon900_10
AATACATGAACCCCCTCTGAGTGCAGTGACTCTAGCCTTCCAGTGGTTCTTTGAATTACATCATTAACGGCCAAT
CCTGCCAATGCATTTGTGAACCTGA
>LOC105762837_exon900_19
ACCCCCTCTGAGTGCAGTGACTCTAGCCTTCCAGTGGTTCTTTGAATTACATCATTAACGGCCAATCCTGCCAAT
GCATTTGTGAACCTGAAAATGTGGA
>LOC105762837_exon1500_0
CAGCCTTGCTGACACTTGAATCATGTAAAATTTTCATCTCATCATGACTGCCCTCTGTTCCAGTCTCTATTTTCT
TCCCTTTGGCCGACTCTGGACCAGT
>LOC105762837_exon1500_26
AAAATTTTCATCTCATCATGACTGCCCTCTGTTCCAGTCTCTATTTTCTTCCCTTTGGCCGACTCTGGACCAGTT
CCATCCATTTCAGCATCTAAACTGA
>LOC105762837_exon1500_51
CCTCTGTTCCAGTCTCTATTTTCTTCCCTTTGGCCGACTCTGGACCAGTTCCATCCATTTCAGCATCTAAACTGA
AAATTTGTTGGATTTGTTTAAGCTT
>LOC105762837_exon1900_0
CCTCCAACTGCTCTGGACCTCCATATATGTAGAAGCATCGATCAAAGCTAACTTCCTCTAACGACTGATCCTCAT
CAGATGGCTGTTCAGTGCCCTCAGC
>LOC105762837_exon1900_19
TCCATATATGTAGAAGCATCGATCAAAGCTAACTTCCTCTAACGACTGATCCTCATCAGATGGCTGTTCAGTGCC
CTCAGCATTGTTTTCGACCTCTATA
>LOC105762837_exon1900_38
CGATCAAAGCTAACTTCCTCTAACGACTGATCCTCATCAGATGGCTGTTCAGTGCCCTCAGCATTGTTTTCGACC
TCTATACCAGTTTCATTGATCAGTA
>LOC105768717_exon6200_0
GTATCAGATTCAGCAGCAGCCGGTTTGGATTCATAAACAAGATCAGAAATTCCAGTGGAAACAGCTGGATCACTC
AAACCAGTTGATGCACTGATATTAT

>LOC105768717_exon6200_16
CAGCCGGTTTGGATTCATAAACAAGATCAGAAATTCCAGTGGAAACAGCTGGATCACTCAAACCAGTTGATGCAC
TGATATTATGCCGTGTGCGATATAT
>LOC105768717_exon6200_32
ATAAACAAGATCAGAAATTCCAGTGGAAACAGCTGGATCACTCAAACCAGTTGATGCACTGATATTATGCCGTGT
GCGATATATGTCGATAAGTTTTGCA
>LOC105768717_exon6450_0
CTTATTGGCCCTAAAGGGAGATACTTAGCTCGTGGCACGTAACAAAACAGTGAAACAAGATCCAGCAGCCTCTCA
TGGGTTTCGTATAATTTCTTAAGCT
>LOC105768717_exon6450_17
GAGATACTTAGCTCGTGGCACGTAACAAAACAGTGAAACAAGATCCAGCAGCCTCTCATGGGTTTCGTATAATTT
CTTAAGCTCCTCATCTTTCCATTCT
>LOC105768717_exon6450_34
GCACGTAACAAAACAGTGAAACAAGATCCAGCAGCCTCTCATGGGTTTCGTATAATTTCTTAAGCTCCTCATCTT
TCCATTCTTTATTTGCATTATCATG
>LOC105768717_exon6800_0
AGATGCATTTCAGATAACTGAGACTGCACACCACCCTGTGCTAAGGCATGTAAAAATTCATAGATCTGTAGCCTA
AATGGCTCACCAGACCGGATTGCTA
>LOC105768717_exon6800_16
ACTGAGACTGCACACCACCCTGTGCTAAGGCATGTAAAAATTCATAGATCTGTAGCCTAAATGGCTCACCAGACC
GGATTGCTACAGTTGTAAGCGCTTG
>LOC105768717_exon6800_32
ACCCTGTGCTAAGGCATGTAAAAATTCATAGATCTGTAGCCTAAATGGCTCACCAGACCGGATTGCTACAGTTGT
AAGCGCTTGAGCAGCAATAATGCGA
>LOC105768717_exon8130_0
ACCTCCCAGCTACCGCTGAAAGCACACCTCTGGAGTCGAGTCAATGCACCTGCTAGTGTGGGATTACGGGAGCTG
GCAGCAGCAACCATTTTATCCGCAT
>LOC105768717_exon8130_17
GAAAGCACACCTCTGGAGTCGAGTCAATGCACCTGCTAGTGTGGGATTACGGGAGCTGGCAGCAGCAACCATTTT
ATCCGCATCTAACATCATCAATGCA
>LOC105768717_exon8130_34
GTCGAGTCAATGCACCTGCTAGTGTGGGATTACGGGAGCTGGCAGCAGCAACCATTTTATCCGCATCTAACATCA
TCAATGCAGTACCCGGAGGCGTTGC
>LOC105768717_exon8400_0
ACAGCTGCTTGGACTAATCTCTGTAATGCTAGAGCCGACTTTGGGTCAGATGCAGATACCCTGTCTACTGATGTA
AATCCAGACATTGATCCAGGCTGTG
>LOC105768717_exon8400_17
TCTCTGTAATGCTAGAGCCGACTTTGGGTCAGATGCAGATACCCTGTCTACTGATGTAAATCCAGACATTGATCC
AGGCTGTGGGGGTGGGAGGTCTAGG
>LOC105768717_exon8400_34
CCGACTTTGGGTCAGATGCAGATACCCTGTCTACTGATGTAAATCCAGACATTGATCCAGGCTGTGGGGGTGGGA
GGTCTAGGACAATAGTGACTGCTTC
>LOC105768736_exon1100_0
TCGCCGGCTGTTGGGGATTCGAAGAAGAAGATGGCAACGATTGTTGCTGGGATTTCAAAGGTGTCCGAAGCTCTT
GTGGGGTCGGCAAAGAGTAGTAGAA
>LOC105768736_exon1100_29
GATGGCAACGATTGTTGCTGGGATTTCAAAGGTGTCCGAAGCTCTTGTGGGGTCGGCAAAGAGTAGTAGAAAGGG
ATGGGATGAGCAGCCAGAGAAAGGT
>LOC105768736_exon1100_58
AGGTGTCCGAAGCTCTTGTGGGGTCGGCAAAGAGTAGTAGAAAGGGATGGGATGAGCAGCCAGAGAAAGGTTCAG
GAGAACACAAGGAGAAAGCTTCTGT
>LOC105768736_exon1600_0
CTGAAGATGGTCCGGCCCCAGAGAAACCAACATGTGGGGGTGGGGCAGCGGGAATCACCATTCATGAAAAGAAAT
GGACTGATGGCAGTGTTTCATGGGA
>LOC105768736_exon1600_16
CCCAGAGAAACCAACATGTGGGGGTGGGGCAGCGGGAATCACCATTCATGAAAAGAAATGGACTGATGGCAGTGT
TTCATGGGATATCATTTCTGCTGAT

>LOC105768736_exon1600_32
TGTGGGGGTGGGGCAGCGGGAATCACCATTCATGAAAAGAAATGGACTGATGGCAGTGTTTCATGGGATATCATT
TCTGCTGATCTTGCAAACCTTGGAA
>LOC105768736_exon2360_0
GTCCTAATGCAGGAGGCTATGCAAAGGAGAGCTCTTGCCTCCAAAGCTGCAGCAGAGGCACTGGAAGAGGCCATT
GCTACCGAGTCTGTTGTTAGGAATT
>LOC105768736_exon2360_6
ATGCAGGAGGCTATGCAAAGGAGAGCTCTTGCCTCCAAAGCTGCAGCAGAGGCACTGGAAGAGGCCATTGCTACC
GAGTCTGTTGTTAGGAATTTAAGGT
>LOC105768736_exon2360_12
GAGGCTATGCAAAGGAGAGCTCTTGCCTCCAAAGCTGCAGCAGAGGCACTGGAAGAGGCCATTGCTACCGAGTCT
GTTGTTAGGAATTTAAGGTTAGGGT
>LOC105768736_exon3100_0
TTGGACTGTGGCTCCATTGCAGGCGTTTTATCACAGTTGAAACGGGTCAATGATTGGTTAGACCGGGCAGTATCA
AACGGGGAAGAACCGTTGACGGACA
>LOC105768736_exon3100_16
TTGCAGGCGTTTTATCACAGTTGAAACGGGTCAATGATTGGTTAGACCGGGCAGTATCAAACGGGGAAGAACCGT
TGACGGACAAGGTTGAGAAGTTGAA
>LOC105768736_exon3100_31
CACAGTTGAAACGGGTCAATGATTGGTTAGACCGGGCAGTATCAAACGGGGAAGAACCGTTGACGGACAAGGTTG
AGAAGTTGAAGAGGAAAATCTATGG
>LOC105769859_exon620_0
CTTGAATAAAGTGTCGAAGAGAGCTAATTTATTTCCTTGTATTAAGTGTGAGAAGAAGGATGAACCAATTGAGCA
TGTGTCTGTTGAGCGTCCTCCATAT
>LOC105769859_exon620_10
GTGTCGAAGAGAGCTAATTTATTTCCTTGTATTAAGTGTGAGAAGAAGGATGAACCAATTGAGCATGTGTCTGTT
GAGCGTCCTCCATATTATAGCTATA
>LOC105769859_exon620_20
GAGCTAATTTATTTCCTTGTATTAAGTGTGAGAAGAAGGATGAACCAATTGAGCATGTGTCTGTTGAGCGTCCTC
CATATTATAGCTATATGGATTCGAC
>LOC105769859_exon1120_0
ATTGATCCAAAAGTGAAGAAACCAATGGAGGAACCACTTACAAGTGAAGAACTATGGTGGAATTGGAGAAAGCCA
GAAAAAGAACAATGGTCTAGATGGC
>LOC105769859_exon1120_9
AAAGTGAAGAAACCAATGGAGGAACCACTTACAAGTGAAGAACTATGGTGGAATTGGAGAAAGCCAGAAAAAGAA
CAATGGTCTAGATGGCAAAGGAGAC
>LOC105769859_exon1120_25
TGGAGGAACCACTTACAAGTGAAGAACTATGGTGGAATTGGAGAAAGCCAGAAAAAGAACAATGGTCTAGATGGC
AAAGGAGACGACCTGATGTTGAAAC
>LOC105769859_exon1840_0
GGTTTTTCTGAAAGCAATGGCTGAGACTGGGCAAGTGAAGCTCTATGGTGAAATTCCAACATTGACTGAAACTTC
CCTTTACAGAGCTAGGCGCCATCTT
>LOC105769859_exon1840_10
AAAGCAATGGCTGAGACTGGGCAAGTGAAGCTCTATGGTGAAATTCCAACATTGACTGAAACTTCCCTTTACAGA
GCTAGGCGCCATCTTTTCAAGGAAG
>LOC105769859_exon1840_20
CTGAGACTGGGCAAGTGAAGCTCTATGGTGAAATTCCAACATTGACTGAAACTTCCCTTTACAGAGCTAGGCGCC
ATCTTTTCAAGGAAGAAAGGTTTAA
>LOC105769859_exon2380_0
CTAGAAAGAATTGGTCCAATGGCGTACTACTCTGAATGGGTGAAAGCATGGAAGAGAGACACATCACGTGAAGCT
ATTCAGAAACATTTTGAAGAGACTG
>LOC105769859_exon2380_18
ATGGCGTACTACTCTGAATGGGTGAAAGCATGGAAGAGAGACACATCACGTGAAGCTATTCAGAAACATTTTGAA
GAGACTGGTGAAGATGAAAATGCTC
>LOC105769859_exon2380_35
ATGGGTGAAAGCATGGAAGAGAGACACATCACGTGAAGCTATTCAGAAACATTTTGAAGAGACTGGTGAAGATGA
AATGCTCAACTGATTGAAATGTTC

>LOC105769859_exon3150_0
TTTGGGGTGGAGATCCCGTTTACCCAACCGTGAACTACATTCAAGATCCAGATGAAGTGATTGATTATAGGGGAC
CAGATTTTCATGAACCAACACCAAA
>LOC105769859_exon3150_11
GATCCCGTTTACCCAACCGTGAACTACATTCAAGATCCAGATGAAGTGATTGATTATAGGGGACCAGATTTTCAT
GAACCAACACCAAATATGCTGGCCC
>LOC105769859_exon3150_22
CCCAACCGTGAACTACATTCAAGATCCAGATGAAGTGATTGATTATAGGGGACCAGATTTTCATGAACCAACACC
AAATATGCTGGCCCATCTTAAGGAG
>LOC105777240_exon600_0
AGAGGTGCCCCTATTTGAGGTGGAAGCAACAGCTGTGGAGCCAGTATTCCAAAAGCTATATTCATATATATTTGA
CATGGATAGTGGGGGATATTCCGCT
>LOC105777240_exon600_8
CCCTATTTGAGGTGGAAGCAACAGCTGTGGAGCCAGTATTCCAAAAGCTATATTCATATATATTTGACATGGATA
GTGGGGGATATTCCGCTAAAGAGAT
>LOC105777240_exon600_24
AGCAACAGCTGTGGAGCCAGTATTCCAAAAGCTATATTCATATATATTTGACATGGATAGTGGGGGATATTCCGC
TAAAGAGATGGATAGACCTGTGCCA
>LOC105777240_exon900_0
AGGTCAGAATGGATCCTAGGAATAAGGACGTTGATCTTGATAATTTGATGTATAGCAAACTTGCCTCACTGACTA
ATGAAGAAATGAAACAACAAGAGGG
>LOC105777240_exon900_14
CCTAGGAATAAGGACGTTGATCTTGATAATTTGATGTATAGCAAACTTGCCTCACTGACTAATGAAGAAATGAAA
CAACAAGAGGGAGATTATATATATC
>LOC105777240_exon900_27
ACGTTGATCTTGATAATTTGATGTATAGCAAACTTGCCTCACTGACTAATGAAGAAATGAAACAACAAGAGGGAG
ATTATATATATCGATATCGATACAA
>LOC105777240_exon1350_0
AGTATCTTATTTCCAGGTGGTATGGCTTCTGTCAGTAGTCACGCTACCCATGATAATTTCATGGGACATCTTGCT
GCTTTGGTGGCAAACACAGTTGAGC
>LOC105777240_exon1350_11
TCCAGGTGGTATGGCTTCTGTCAGTAGTCACGCTACCCATGATAATTTCATGGGACATCTTGCTGCTTTGGTGGC
AAACACAGTTGAGCATGTTATAGCT
>LOC105777240_exon1350_21
ATGGCTTCTGTCAGTAGTCACGCTACCCATGATAATTTCATGGGACATCTTGCTGCTTTGGTGGCAAACACAGTT
GAGCATGTTATAGCTCCAGATGTTC
>LOC105777240_exon1660_0
GTTTGAAACTGTTGATCTGACCACAAGGCTGCTTATACCTATTATTGTCCTCCAAAACCATAACCGATATAATAT
TATGGTAAAAGGTCATAACTACAGC
>LOC105777240_exon1660_9
TGTTGATCTGACCACAAGGCTGCTTATACCTATTATTGTCCTCCAAAACCATAACCGATATAATATTATGGTAAA
AGGTCATAACTACAGCATAGATATT
>LOC105777240_exon1660_25
AGGCTGCTTATACCTATTATTGTCCTCCAAAACCATAACCGATATAATATTATGGTAAAAGGTCATAACTACAGC
ATAGATATTGGGGAGATTGAAGCAG
>LOC105777240_exon2200_0
GTGAAGAAGTTGGTTCATGATGACCAAGAAGTAGTGATTATTGGGGGATCACATGCACTGCACCATCATGAGAAG
TTGGCTATTGCTGTGTCAAAAGCTA
>LOC105777240_exon2200_9
TTGGTTCATGATGACCAAGAAGTAGTGATTATTGGGGGATCACATGCACTGCACCATCATGAGAAGTTGGCTATT
GCTGTGTCAAAAGCTATGCGGGGCC
>LOC105777240_exon2200_26
AGAAGTAGTGATTATTGGGGGATCACATGCACTGCACCATCATGAGAAGTTGGCTATTGCTGTGTCAAAAGCTAT
GCGGGGCCATTCCTTCCAAGAAACA
>LOC105777240_exon2940_0
GTCTTGAAGCATAAACCTCTATGGGCTAGTTACAACTCGAAAGTTGGCAAGGATAAGAAAACGAAAAAACAGAGG
AAAAGAGGGGATCTGCACCCAACTT

>LOC105777240_exon2940_11
TAAACCTCTATGGGCTAGTTACAACTCGAAAGTTGGCAAGGATAAGAAAACGAAAAAACAGAGGAAAAGAGGGGA
TCTGCACCCAACTTATGGAACTAGA
>LOC105777240_exon2940_22
GGGCTAGTTACAACTCGAAAGTTGGCAAGGATAAGAAAACGAAAAAACAGAGGAAAAGAGGGGATCTGCACCCAA
CTTATGGAACTAGAGTTATTCCTGT
>LOC105777240_exon3800_0
CTTTGTGCTATCTTTGGCTGATGTGGATCCACAACTTATGATGGAAGATGAAAGCTTTGTTTGGACTGGCAATGA
TGTTGTCATAGTACTTGAGCACCAA
>LOC105777240_exon3800_10
TCTTTGGCTGATGTGGATCCACAACTTATGATGGAAGATGAAAGCTTTGTTTGGACTGGCAATGATGTTGTCATA
GTACTTGAGCACCAAAGTAAAAATA
>LOC105777240_exon3800_20
ATGTGGATCCACAACTTATGATGGAAGATGAAAGCTTTGTTTGGACTGGCAATGATGTTGTCATAGTACTTGAGC
ACCAAAGTAAAAATATACCTTTGAG
>LOC105777240_exon4170_0
CTTGCTTCTGCTGTGGGTGGGTTGAGTGCACCTTATGAAAAAGCTTCTCATGTACATGAAAGGCCCTTGGTGAAT
TGGCTCTGGGCTGCGGGTTGTCACC
>LOC105777240_exon4170_9
GCTGTGGGTGGGTTGAGTGCACCTTATGAAAAAGCTTCTCATGTACATGAAAGGCCCTTGGTGAATTGGCTCTGG
GCTGCGGGTTGTCACCCGTTTGGAC
>LOC105777240_exon4170_25
GTGCACCTTATGAAAAAGCTTCTCATGTACATGAAAGGCCCTTGGTGAATTGGCTCTGGGCTGCGGGTTGTCACC
CGTTTGGACCCTTCTCAAATACATC
>LOC105777240_exon4740_0
AAGGGAAAGAAGAACAAGTCAACTACTGAGCTATGGGTGGAGAAATTTTACAAGAAAACAACTAACCTGCCCGAA
CCTTTCCCACATGAATTAGTTGAGC
>LOC105777240_exon4740_10
AGAACAAGTCAACTACTGAGCTATGGGTGGAGAAATTTTACAAGAAAACAACTAACCTGCCCGAACCTTTCCCAC
ATGAATTAGTTGAGCGAATAGAGAA
>LOC105777240_exon4740_20
AACTACTGAGCTATGGGTGGAGAAATTTTACAAGAAAACAACTAACCTGCCCGAACCTTTCCCACATGAATTAGT
TGAGCGAATAGAGAAATACTTGGAT
>LOC105777240_exon5240_0
AGCGAGAGGGAGAGGATGAGATGCTGTAATATCGAATTCAAGTATCCAGTGCATTCTTCACAAACTTTCGTATAT
GGTGGAATTCTTCTCGCCGGGTTCT
>LOC105777240_exon5240_19
GATGCTGTAATATCGAATTCAAGTATCCAGTGCATTCTTCACAAACTTTCGTATATGGTGGAATTCTTCTCGCCG
GGTTCTTTGTATATTTCATTGTCAT
>LOC105777240_exon5240_37
TCAAGTATCCAGTGCATTCTTCACAAACTTTCGTATATGGTGGAATTCTTCTCGCCGGGTTCTTTGTATATTTCA
TTGTCATTTTCTTTTCATCACCCCC
>LOC105792102_exon3400_0
GAATGAAGCTGGAGAACTAAGTAGTCTACAAGAATCTGGTGAAGCATCTTATGAACATTCTCTCCAAGATGGTGA
AAGAAATCGGACCTATGGGTTAATA
>LOC105792102_exon3400_10
GGAGAACTAAGTAGTCTACAAGAATCTGGTGAAGCATCTTATGAACATTCTCTCCAAGATGGTGAAAGAAATCGG
ACCTATGGGTTAATAAATGTTGTAG
>LOC105792102_exon3400_20
GTAGTCTACAAGAATCTGGTGAAGCATCTTATGAACATTCTCTCCAAGATGGTGAAAGAAATCGGACCTATGGGT
TAATAAATGTTGTAGAAAATGTGGA
>LOC105792102_exon4000_0
AGGAGAAGTGTGTCTAATCTTCTTCGTAGTGGTTTTCGTGAGAGTCTTGACCATTTGATACAATCATACGTGGAA
AGGCAAAATCAAGGTTCTACTGGCT
>LOC105792102_exon4000_10
TGTCTAATCTTCTTCGTAGTGGTTTTCGTGAGAGTCTTGACCATTTGATACAATCATACGTGGAAAGGCAAAATC
AAGGTTCTACTGGCTGGGACCTAAA

>LOC105792102_exon4000_20
TCTTCGTAGTGGTTTTCGTGAGAGTCTTGACCATTTGATACAATCATACGTGGAAAGGCAAAATCAAGGTTCTAC
TGGCTGGGACCTAAATGAAGCAGCT
>LOC105792102_exon4830_0
GACTGCAGCAAAGAATGAATAACATGCAGATGATGCTGGAGGCCTGCATGGATATGCAACTTGAGTTGCAGCGAT
CAATAAGACAAGAAGTTTCTGCTGC
>LOC105792102_exon4830_10
AAGAATGAATAACATGCAGATGATGCTGGAGGCCTGCATGGATATGCAACTTGAGTTGCAGCGATCAATAAGACA
AGAAGTTTCTGCTGCCCTGAACCGG
>LOC105792102_exon4830_20
AACATGCAGATGATGCTGGAGGCCTGCATGGATATGCAACTTGAGTTGCAGCGATCAATAAGACAAGAAGTTTCT
GCTGCCCTGAACCGGTCAGCTGGTT
>LOC105792102_exon5270_0
AGATATGATTTATGACAACTTGCCCGAGGATGCATCATCTAACTGGGATAATGTTCGGAAAGGAATCTGCTGTAT
ATGCTGTGAAGGCAATATCGATACA
>LOC105792102_exon5270_10
TATGACAACTTGCCCGAGGATGCATCATCTAACTGGGATAATGTTCGGAAAGGAATCTGCTGTATATGCTGTGAA
GGCAATATCGATACATTACTATACA
>LOC105792102_exon5270_20
TGCCCGAGGATGCATCATCTAACTGGGATAATGTTCGGAAAGGAATCTGCTGTATATGCTGTGAAGGCAATATCG
ATACATTACTATACAGGTAAATCTA
>LOC105792102_exon5480_0
AGATGTGGACACATGTGCGCATGCTCGAAATGCGCCAATGAGTTGGTTCAAGGTGGAGGGAAGTGTCCGATGTGT
CGTGCACCAGTAGTTGAGGTCATAC
>LOC105792102_exon5480_10
ACATGTGCGCATGCTCGAAATGCGCCAATGAGTTGGTTCAAGGTGGAGGGAAGTGTCCGATGTGTCGTGCACCAG
TAGTTGAGGTCATACGTGCTTACTC
>LOC105792102_exon5480_20
ATGCTCGAAATGCGCCAATGAGTTGGTTCAAGGTGGAGGGAAGTGTCCGATGTGTCGTGCACCAGTAGTTGAGGT
CATACGTGCTTACTCAATCCTTTAA
>LOC105794602_exon1900_0
ATCTCAATTATCAGATGATTTCTCCCGGGATGGTTCGAGCAATAGGTCAGAAACCTCTGGTTTACTACCATCTCT
AAGTACAACAGGCAAGGATCTAGAG
>LOC105794602_exon1900_24
CCGGGATGGTTCGAGCAATAGGTCAGAAACCTCTGGTTTACTACCATCTCTAAGTACAACAGGCAAGGATCTAGA
GATGCCAATGCCACCTCTGGCTCTT
>LOC105794602_exon1900_48
AGAAACCTCTGGTTTACTACCATCTCTAAGTACAACAGGCAAGGATCTAGAGATGCCAATGCCACCTCTGGCTCT
TGCCCCATTACAAGAACATGGGATC
>LOC105794602_exon2550_0
CAGCCTTTGCAAGTAGCTATAGAAGAACCTGCTTTGCGCCAAGCCTTGAGCAATTTGATAGAGGGTGCATTATTA
CGTACCCGGGTTGGGGGGAAGATTG
>LOC105794602_exon2550_18
ATAGAAGAACCTGCTTTGCGCCAAGCCTTGAGCAATTTGATAGAGGGTGCATTATTACGTACCCGGGTTGGGGGG
AAGATTGAAATTGTTTCTACCAGTG
>LOC105794602_exon2550_35
GCGCCAAGCCTTGAGCAATTTGATAGAGGGTGCATTATTACGTACCCGGGTTGGGGGGAAGATTGAAATTGTTTC
TACCAGTGCACCAGCAGGTGGTGCT
>LOC105794602_exon3000_0
GACACAGATGCATTCCCTCACACCGTTTGGTGCCGAACTCTTCTCGGAAAACATGATTGAAGACAACATGACATG
GAATTTTGTCGCCGGGCTAACTGTA
>LOC105794602_exon3000_8
TGCATTCCCTCACACCGTTTGGTGCCGAACTCTTCTCGGAAAACATGATTGAAGACAACATGACATGGAATTTTG
TCGCCGGGCTAACTGTAGCACGTGA
>LOC105794602_exon3000_23
CGTTTGGTGCCGAACTCTTCTCGGAAAACATGATTGAAGACAACATGACATGGAATTTTGTCGCCGGGCTAACTG
TAGCACGTGAAATACTTGAAAGTTA

>LOC105794602_exon3100_0
CTCTCGGAGCAGGTGGGACTCGTGTAGAACTATGGCTTCCTTCATTTTCTGCTTTATCCGACATGAACAACCTTA
GTCAGGAGGCATAACAAGAAAGTGG
>LOC105794602_exon3100_10
AGGTGGGACTCGTGTAGAACTATGGCTTCCTTCATTTTCTGCTTTATCCGACATGAACAACCTTAGTCAGGAGGC
ATAACAAGAAAGTGGAATAGCATGT
>LOC105794602_exon3100_20
CGTGTAGAACTATGGCTTCCTTCATTTTCTGCTTTATCCGACATGAACAACCTTAGTCAGGAGGCATAACAAGAA
AGTGGAATAGCATGTTCTTTTGATT
>LOC105799519_exon300_0
TGCATGCTCATGGTTTCCATTCACTGCTCCATTGGCAACAATTGTTGGTGACAATGGCAATTTATCAATTATTGA
TGTAAGTGGAGAATTCAATTCCTCC
>LOC105799519_exon300_10
TGGTTTCCATTCACTGCTCCATTGGCAACAATTGTTGGTGACAATGGCAATTTATCAATTATTGATGTAAGTGGA
GAATTCAATTCCTCCAGTTCCTCGT
>LOC105799519_exon300_20
TCACTGCTCCATTGGCAACAATTGTTGGTGACAATGGCAATTTATCAATTATTGATGTAAGTGGAGAATTCAATT
CCTCCAGTTCCTCGTCACTGGTATA
>LOC105799519_exon800_0
CTCAGCATTCAAGGCTTCCAATACAGCACCTGGAACAGGATCGGGACAAAATTCATCGGGAGTGAAGTTGCAAAG
AATGCGTTTAACCAATGAAAGACCT
>LOC105799519_exon800_10
AAGGCTTCCAATACAGCACCTGGAACAGGATCGGGACAAAATTCATCGGGAGTGAAGTTGCAAAGAATGCGTTTA
ACCAATGAAAGACCTATTGATGGGC
>LOC105799519_exon800_20
ATACAGCACCTGGAACAGGATCGGGACAAAATTCATCGGGAGTGAAGTTGCAAAGAATGCGTTTAACCAATGAAA
GACCTATTGATGGGCACACCTAAAG
>LOC105799519_exon1000_0
CTCTTTTCTAACTGAACGGTCCAAAAGCATGTCCTTTGGAAGCATTAGAAGATCACTCAGGGAATTAAGAAAGGG
GAAGGATTTAGGTTCCCCATCTCCA
>LOC105799519_exon1000_10
ACTGAACGGTCCAAAAGCATGTCCTTTGGAAGCATTAGAAGATCACTCAGGGAATTAAGAAAGGGGAAGGATTTA
GGTTCCCCATCTCCATTTTGTCTGA
>LOC105799519_exon1000_20
CCAAAAGCATGTCCTTTGGAAGCATTAGAAGATCACTCAGGGAATTAAGAAAGGGGAAGGATTTAGGTTCCCCAT
CTCCATTTTGTCTGAAATCATCTTG
>LOC105799519_exon1900_0
ACAGCATTTTTTAGCTGTGCACCAGATCCAAAGCTAAGATCTCCAGCTGGAATAGGCAGAACCTTTGAATCTACA
ATAGGATCAGATACAGGATCTGTTG
>LOC105799519_exon1900_10
TTAGCTGTGCACCAGATCCAAAGCTAAGATCTCCAGCTGGAATAGGCAGAACCTTTGAATCTACAATAGGATCAG
ATACAGGATCTGTTGGGATCTCATG
>LOC105799519_exon1900_20
ACCAGATCCAAAGCTAAGATCTCCAGCTGGAATAGGCAGAACCTTTGAATCTACAATAGGATCAGATACAGGATC
TGTTGGGATCTCATGAGCAGACTCC
>LOC105799519_exon2300_0
GGCAAGCAACCACACTCATGACCGCCTGCTCTAACGGGACAGAGTCGTTGAAAAGCATCTTTGAAAGCAGTTTTC
CACAAGTTGATAGAGAAACTTCCTT
>LOC105799519_exon2300_20
ACCGCCTGCTCTAACGGGACAGAGTCGTTGAAAAGCATCTTTGAAAGCAGTTTTCCACAAGTTGATAGAGAAACT
TCCTTGCTGTTGGTCGCCCAAGACT
>LOC105799519_exon2300_39
CAGAGTCGTTGAAAAGCATCTTTGAAAGCAGTTTTCCACAAGTTGATAGAGAAACTTCCTTGCTGTTGGTCGCCC
AAGACTGGCCCCAACAACTTTCCAA
>LOC105799519_exon2600_0
CCTGCCACCACACTGACTCAACTATTCGAGAGAATATCCAAGACTCCACTTTTTCTAGTGCAGATATGAAGGTTT
CGGTCTCTTGCCAATCATCGACAAG

>LOC105799519_exon2600_16
CTCAACTATTCGAGAGAATATCCAAGACTCCACTTTTTCTAGTGCAGATATGAAGGTTTCGGTCTCTTGCCAATC
ATCGACAAGCGGCCCAAAAGTGTTT
>LOC105799519_exon2600_31
GAATATCCAAGACTCCACTTTTTCTAGTGCAGATATGAAGGTTTCGGTCTCTTGCCAATCATCGACAAGCGGCCC
AAAAGTGTTTACTTGTTTACTACCA
>LOC105799519_exon3000_0
ACCTTGAAACATCATTACCGCACGATTTTGCAATCAAAATAAGCCCAGACACTGTGTTTTTAGCAATTGTGGCTC
GTTTGTTTTGAGTCCAATGCTTGCA
>LOC105799519_exon3000_20
CACGATTTTGCAATCAAAATAAGCCCAGACACTGTGTTTTTAGCAATTGTGGCTCGTTTGTTTTGAGTCCAATGC
TTGCAAGCATGTATATAGAGTCTAG
>LOC105799519_exon3000_39
TAAGCCCAGACACTGTGTTTTTAGCAATTGTGGCTCGTTTGTTTTGAGTCCAATGCTTGCAAGCATGTATATAGA
GTCTAGCAAGACGTCTAGCAGGTGT
>LOC105802744_exon360_0
CCGGGAACTGGTACTCGGTGCCGGACCTCCGACTCCGGGACCACCGATTTATGGCCCCTCTCGATTATAATCGCG
ATGCTTCTTCGAAGATATCCATTTT
>LOC105802744_exon360_10
GTACTCGGTGCCGGACCTCCGACTCCGGGACCACCGATTTATGGCCCCTCTCGATTATAATCGCGATGCTTCTTC
GAAGATATCCATTTTCGCTCGCGAA
>LOC105802744_exon360_20
CCGGACCTCCGACTCCGGGACCACCGATTTATGGCCCCTCTCGATTATAATCGCGATGCTTCTTCGAAGATATCC
ATTTTCGCTCGCGAAGTCGTTGCTG
>LOC105802744_exon660_0
TTGGGAAAGAAGATCAGCCAATGCCATACCTTTTATATCTTCAAGGAGGGCCTGGTTTTGAATGCCCCCGTCCAA
CTGAAGGTGGTGGATGGATACTTAA
>LOC105802744_exon660_10
AGATCAGCCAATGCCATACCTTTTATATCTTCAAGGAGGGCCTGGTTTTGAATGCCCCCGTCCAACTGAAGGTGG
TGGATGGATACTTAAAGCATGCGAA
>LOC105802744_exon660_20
ATGCCATACCTTTTATATCTTCAAGGAGGGCCTGGTTTTGAATGCCCCCGTCCAACTGAAGGTGGTGGATGGATA
CTTAAAGCATGCGAAGAGTTTCGGG
>LOC105802744_exon900_0
CGAGGAACAGGTTTATCGACCCCTTTGACTCCATCATCTATGCAGCAAATAAAGTCTGCCCAAAAGCTGGCCGAT
TACTTGACACATTTCCGAGCCGACA
>LOC105802744_exon900_10
GTTTATCGACCCCTTTGACTCCATCATCTATGCAGCAAATAAAGTCTGCCCAAAAGCTGGCCGATTACTTGACAC
ATTTCCGAGCCGACAGCATTGTCAA
>LOC105802744_exon900_20
CCCTTTGACTCCATCATCTATGCAGCAAATAAAGTCTGCCCAAAAGCTGGCCGATTACTTGACACATTTCCGAGC
CGACAGCATTGTCAACGATGCTGAA
>LOC105802744_exon1200_0
AGTGCTTTTAACAGGAGGGATACCTCCAATTGGAGATGGATGTACTGCAGATTCTGTTTATAGAGCTTGCTTTGA
ACAGATTATTCATCAGAATGAAAAA
>LOC105802744_exon1200_10
ACAGGAGGGATACCTCCAATTGGAGATGGATGTACTGCAGATTCTGTTTATAGAGCTTGCTTTGAACAGATTATT
CATCAGAATGAAAAATACTATAAGA
>LOC105802744_exon1200_20
TACCTCCAATTGGAGATGGATGTACTGCAGATTCTGTTTATAGAGCTTGCTTTGAACAGATTATTCATCAGAATG
AAAAATACTATAAGAGGTTCCCTCA
>LOC105802744_exon1500_0
ACCTCTTCCATGTAGGGGATACTTAACGCCCAGGGGGCTGCAATTTCTTGGTCTATCCGGTTTAGGAGGAAGTGC
AGGTTTTGAACGACTGCATTATCTG
>LOC105802744_exon1500_10
TGTAGGGGATACTTAACGCCCAGGGGGCTGCAATTTCTTGGTCTATCCGGTTTAGGAGGAAGTGCAGGTTTTGAA
CGACTGCATTATCTGTAAGACATTA

>LOC105802744_exon1500_20
ACTTAACGCCCAGGGGGCTGCAATTTCTTGGTCTATCCGGTTTAGGAGGAAGTGCAGGTTTTGAACGACTGCATT
ATCTGTAAGACATTAGAATTCACAC
>LOC105802744_exon2100_0
TGTTCTAAAGGGTGCTTCGTCGCGGTGGTCTGCTCACAGAGTAAGGGCTGACCATGAGAGCAACTTCGATGCAAT
CAAGGCTGCAAAAGAAGGGCGTCCC
>LOC105802744_exon2100_10
GGTGCTTCGTCGCGGTGGTCTGCTCACAGAGTAAGGGCTGACCATGAGAGCAACTTCGATGCAATCAAGGCTGCA
AAAGAAGGGCGTCCCGTCCTTTTTA
>LOC105802744_exon2100_20
CGCGGTGGTCTGCTCACAGAGTAAGGGCTGACCATGAGAGCAACTTCGATGCAATCAAGGCTGCAAAAGAAGGGC
GTCCCGTCCTTTTTACGGGGGAGAT
>LOC105802814_exon330_0
TGCAAAAAGGTGTGCCTTTCATGCAATTTTGACCAATGATGAACCAATCTGGCTGCTTTGGCATCCCAAAGTTTA
ACGTCTCCATCTTTGCTTCCCGTTA
>LOC105802814_exon330_14
CCTTTCATGCAATTTTGACCAATGATGAACCAATCTGGCTGCTTTGGCATCCCAAAGTTTAACGTCTCCATCTTT
GCTTCCCGTTAAGAACAAACTGGTA
>LOC105802814_exon330_28
TTGACCAATGATGAACCAATCTGGCTGCTTTGGCATCCCAAAGTTTAACGTCTCCATCTTTGCTTCCCGTTAAGA
ACAAACTGGTATGTGGGATGGTAGA
>LOC105802814_exon660_0
ATCATGGTGCCTGTGCCTTTTAGTCCTTCCAGTAGCTATGAATCGGAAGTCATGAAGTCCAACATCACCATTTTT
ACCACCAGTTACTATTAGGGGAGAA
>LOC105802814_exon660_15
CCTTTTAGTCCTTCCAGTAGCTATGAATCGGAAGTCATGAAGTCCAACATCACCATTTTTACCACCAGTTACTAT
TAGGGGAGAAACAGAACCACTTCCA
>LOC105802814_exon660_29
CAGTAGCTATGAATCGGAAGTCATGAAGTCCAACATCACCATTTTTACCACCAGTTACTATTAGGGGAGAAACAG
AACCACTTCCAATGTCATTGTCAAA
>LOC105802814_exon1170_0
ACCTTCATGGCAAACAATGGAAGCCCGGGAAGTTGCAGACGGAGCCAATGTATCCCATATAACCACATTAACACC
ATTGGAACTACATCCAGCAGCAGCT
>LOC105802814_exon1170_11
AAACAATGGAAGCCCGGGAAGTTGCAGACGGAGCCAATGTATCCCATATAACCACATTAACACCATTGGAACTAC
ATCCAGCAGCAGCTATGATTGATCC
>LOC105802814_exon1170_22
GCCCGGGAAGTTGCAGACGGAGCCAATGTATCCCATATAACCACATTAACACCATTGGAACTACATCCAGCAGCA
GCTATGATTGATCCACTTGAAGTAA
>LOC105802814_exon1600_0
TGCCACGTGCAAACAGTTCCATCTAGTGCAGCAGTAACAAATCTGTGTCCACAATGGTCAAACTGCAAAGCTGAT
ATTGAGGCAAGAGCATAAGGTGGAG
>LOC105802814_exon1600_28
CAGCAGTAACAAATCTGTGTCCACAATGGTCAAACTGCAAAGCTGATATTGAGGCAAGAGCATAAGGTGGAGCAA
CATTTGCAGCAGGCAACACTCCATA
>LOC105802814_exon1600_55
GGTCAAACTGCAAAGCTGATATTGAGGCAAGAGCATAAGGTGGAGCAACATTTGCAGCAGGCAACACTCCATAAG
TTGCTGTTGCTTTATCCTTCCCAAA
>LOC105802814_exon2100_0
CTCCCATAAGTAAATGTGTGTGTTGATAGAACCGACCAAGAACAAAGGCCTTGAGGGGTGACTAGAGAAACACCT
GGTACTGATGTTGTCTACAGTGGCT
>LOC105802814_exon2100_8
AGTAAATGTGTGTGTTGATAGAACCGACCAAGAACAAAGGCCTTGAGGGGTGACTAGAGAAACACCTGGTACTGA
TGTTGTCTACAGTGGCTGGCAGATC
>LOC105802814_exon2100_23
TGATAGAACCGACCAAGAACAAAGGCCTTGAGGGGTGACTAGAGAAACACCTGGTACTGATGTTGTCTACAGTGG
CTGGCAGATCAATAAACTCCTCAAA

>LOC105802814_exon2400_0
GTTGGAACTGGAGTAGATTCACAACCAGCCCAACCATTGTGTGGCCAGTCAGCACCGGACCAGATAAAATCTGAT
TGGTCCTTGGAAGCTATACCATCTT
>LOC105802814_exon2400_11
AGTAGATTCACAACCAGCCCAACCATTGTGTGGCCAGTCAGCACCGGACCAGATAAAATCTGATTGGTCCTTGGA
AGCTATACCATCTTCCCAGTTAAAA
>LOC105802814_exon2400_21
CAACCAGCCCAACCATTGTGTGGCCAGTCAGCACCGGACCAGATAAAATCTGATTGGTCCTTGGAAGCTATACCA
TCTTCCCAGTTAAAAAAAAGTATGC
>LOC105802814_exon3300_0
CCTAATGTCTTTGTACAATTCATCCCATCCTTTGCAAGGCTTAAAATTAAAATAGCTTGACCAATTTACTTTCTC
TTGTGCAAAACTTTCCAATATCAAA
>LOC105802814_exon3300_17
ATTCATCCCATCCTTTGCAAGGCTTAAAATTAAAATAGCTTGACCAATTTACTTTCTCTTGTGCAAAACTTTCCA
ATATCAAAGTAGGATCAGCACACAT
>LOC105802814_exon3300_33
GCAAGGCTTAAAATTAAAATAGCTTGACCAATTTACTTTCTCTTGTGCAAAACTTTCCAATATCAAAGTAGGATC
AGCACACATATTCCATAAGATATCA
>LOC105803814_exon200_0
CAGTTCTAAATTATCACTTGGAATCTCCACTCGGATTGGTAACTTTACTTCCATTTTGAGTCCCGGATCCTTTAA
GCTCAGCAAAACCTGGTGGCAACTT
>LOC105803814_exon200_10
TTATCACTTGGAATCTCCACTCGGATTGGTAACTTTACTTCCATTTTGAGTCCCGGATCCTTTAAGCTCAGCAAA
ACCTGGTGGCAACTTTGGAGGATGC
>LOC105803814_exon200_20
GAATCTCCACTCGGATTGGTAACTTTACTTCCATTTTGAGTCCCGGATCCTTTAAGCTCAGCAAAACCTGGTGGC
AACTTTGGAGGATGCACAATAGGCA
>LOC105803814_exon600_0
AGCCCCAAACTGAAGCCAGGGCTGCCTGAAATGTGGTGGCCGGTTCAACATAGCAGCATGGGTTCCAGAAGAATT
TCCATTGTTTTCTGCATCATCTTTG
>LOC105803814_exon600_10
TGAAGCCAGGGCTGCCTGAAATGTGGTGGCCGGTTCAACATAGCAGCATGGGTTCCAGAAGAATTTCCATTGTTT
TCTGCATCATCTTTGCCTCTAGCTT
>LOC105803814_exon600_20
GCTGCCTGAAATGTGGTGGCCGGTTCAACATAGCAGCATGGGTTCCAGAAGAATTTCCATTGTTTTCTGCATCAT
CTTTGCCTCTAGCTTGAAGGCCTGC
>LOC105803814_exon1000_0
TCTTCCGTTTGTCTTCTAATTGGCATGCCAGATATATAGCAGCTGCAGATATACTAATCGGATTTCGGCGAGTGC
AAAAGCATTTGTTGATCACAACTTC
>LOC105803814_exon1000_10
GTCTTCTAATTGGCATGCCAGATATATAGCAGCTGCAGATATACTAATCGGATTTCGGCGAGTGCAAAAGCATTT
GTTGATCACAACTTCTCCGATATGA
>LOC105803814_exon1000_20
TGGCATGCCAGATATATAGCAGCTGCAGATATACTAATCGGATTTCGGCGAGTGCAAAAGCATTTGTTGATCACA
ACTTCTCCGATATGAGTTGCCAGCT
>LOC105803814_exon1500_0
TCGGCATATGAACTGATATAGAATTACTATTGATAGGTTGACTTAATTGTAAAGCTTCTCCCAGGATCTTGATGT
ACTTGCCAATCTCTTTCTGTGGTAC
>LOC105803814_exon1500_10
AACTGATATAGAATTACTATTGATAGGTTGACTTAATTGTAAAGCTTCTCCCAGGATCTTGATGTACTTGCCAAT
CTCTTTCTGTGGTACATTGGCAGCA
>LOC105803814_exon1500_20
GAATTACTATTGATAGGTTGACTTAATTGTAAAGCTTCTCCCAGGATCTTGATGTACTTGCCAATCTCTTTCTGT
GGTACATTGGCAGCAATAGAGATTT
>LOC105803814_exon2400_0
CCTGGAGGGTTCGAGGCTCTTGAGCTTCCCTAATGGCCTGAACAAGAGCAGCAGTAGCAAGCGCCTCAACACTCC
GGTTCCTCAAACAGGTAGCCGAACA

>LOC105803814_exon2400_10
TCGAGGCTCTTGAGCTTCCCTAATGGCCTGAACAAGAGCAGCAGTAGCAAGCGCCTCAACACTCCGGTTCCTCAA
ACAGGTAGCCGAACAACAATCCCTA
>LOC105803814_exon2400_20
TGAGCTTCCCTAATGGCCTGAACAAGAGCAGCAGTAGCAAGCGCCTCAACACTCCGGTTCCTCAAACAGGTAGCC
GAACAACAATCCCTAAACAACTGAA
>LOC105803814_exon2700_0
CGCTCGAGCTCCGCCAGGTGGCCCGAAAAGGAGAGAGAGGAGCGGAGGAAGAGTGGATTGGGTTCGAGGGACCAT
GTGGAAAAGGCCGTAATAAATCCCG
>LOC105803814_exon2700_10
CCGCCAGGTGGCCCGAAAAGGAGAGAGAGGAGCGGAGGAAGAGTGGATTGGGTTCGAGGGACCATGTGGAAAAGG
CCGTAATAAATCCCGTGGGCTCAAA
>LOC105803814_exon2700_20
GCCCGAAAAGGAGAGAGAGGAGCGGAGGAAGAGTGGATTGGGTTCGAGGGACCATGTGGAAAAGGCCGTAATAAA
TCCCGTGGGCTCAAAGGGGTCGTCT
>LOC105803814_exon2900_0
CTTCGACGACACGTCCGCAAGAGGTGCACTCCGTTATGGACCGGCCGGTTGACGTGGTGGCGCACCGACCTTGCG
CCGCCGAGCAATACGGACATTTCAT
>LOC105803814_exon2900_10
ACGTCCGCAAGAGGTGCACTCCGTTATGGACCGGCCGGTTGACGTGGTGGCGCACCGACCTTGCGCCGCCGAGCA
ATACGGACATTTCATCGGTGATTGT
>LOC105803814_exon2900_20
GAGGTGCACTCCGTTATGGACCGGCCGGTTGACGTGGTGGCGCACCGACCTTGCGCCGCCGAGCAATACGGACAT
TTCATCGGTGATTGTTATTCACTCT
>LOC105803850a_Exon4200_0
CCCACAGGGCTAGCTGACTTGTTCGGACCAACCCAGGAGACTGCAGCACCAGCTCTAGCTCCTGCGGTTCAAGTG
TACACACTGCTTCACGATATACTAT
>LOC105803850a_Exon4200_18
TTGTTCGGACCAACCCAGGAGACTGCAGCACCAGCTCTAGCTCCTGCGGTTCAAGTGTACACACTGCTTCACGAT
ATACTATCTCCTGATGCTCAGACTA
>LOC105803850a_Exon4200_35
GGAGACTGCAGCACCAGCTCTAGCTCCTGCGGTTCAAGTGTACACACTGCTTCACGATATACTATCTCCTGATGC
TCAGACTATGTTGAGAAACTATTTG
>LOC105803850a_Exon4700_0
CAGACTGCAGCCAAAAAGAGGTGTAGGAAACACATGCTTGAAACAGATGAGTTTGTTTCAAGCAACTCTGAAGGT
TTTCTCTTGGACCCCATTACCATCT
>LOC105803850a_Exon4700_14
AAAGAGGTGTAGGAAACACATGCTTGAAACAGATGAGTTTGTTTCAAGCAACTCTGAAGGTTTTCTCTTGGACCC
CATTACCATCTCAACAGCTTATTTG
>LOC105803850a_Exon4700_28
AACACATGCTTGAAACAGATGAGTTTGTTTCAAGCAACTCTGAAGGTTTTCTCTTGGACCCCATTACCATCTCAA
CAGCTTATTTGAAGATGAAGAATCT
>LOC105803850a_Exon4700b_0
CTTGCCTGTTTCAGTCCTGTGCAGAGTGGAGTAGACTCAAGGAGTTTGTTCCACGATTACATAATGGTGTGGGTA
GAGGATATGCAGCTTAGGTTACTCG
>LOC105803850a_Exon4700b_8
TTTCAGTCCTGTGCAGAGTGGAGTAGACTCAAGGAGTTTGTTCCACGATTACATAATGGTGTGGGTAGAGGATAT
GCAGCTTAGGTTACTCGATCTGTGC
>LOC105803850a_Exon4700b_24
AGTGGAGTAGACTCAAGGAGTTTGTTCCACGATTACATAATGGTGTGGGTAGAGGATATGCAGCTTAGGTTACTC
GATCTGTGCAAGGCTGAAAAGGTGC
>LOC105803850a_Exon5000_0
AGTATATAGCACTGAGTTGTGTAAGAGGCTTACCAGTTTCCTTGCTGCATCACCTCCATCTTGTCCTGCACCACA
TGTTAATGAGCTTTTGATTGCAATT
>LOC105803850a_Exon5000_17
TGTGTAAGAGGCTTACCAGTTTCCTTGCTGCATCACCTCCATCTTGTCCTGCACCACATGTTAATGAGCTTTTGA
TTGCAATTGCTGATTTCGAGAGGGA

>LOC105803850a_Exon5000_34
AGTTTCCTTGCTGCATCACCTCCATCTTGTCCTGCACCACATGTTAATGAGCTTTTGATTGCAATTGCTGATTTC
GAGAGGGATCTTGAGTCCTGGAACA
>LOC105803850b_Exon440_0
AGGAGTATGTATTCACAGCGAAGTGCAATGTACATGCAAGTGGGAGCAGAGTATGTGCGGCATGTCTCATCACTT
GTAAAAACTGGCATGAATTCTCTTA
>LOC105803850b_Exon440_14
ACAGCGAAGTGCAATGTACATGCAAGTGGGAGCAGAGTATGTGCGGCATGTCTCATCACTTGTAAAAACTGGCAT
GAATTCTCTTAAAATAGCCTCCTTT
>LOC105803850b_Exon440_28
TGTACATGCAAGTGGGAGCAGAGTATGTGCGGCATGTCTCATCACTTGTAAAAACTGGCATGAATTCTCTTAAAA
TAGCCTCCTTTTCAGTGACGTCTGA
>LOC105803850b_Exon900_0
TTTCATATTTACAGAGCCACTATCATGCTTATTCCAGCTGAAGAGTGCTACAGAAGATACCCAAGTTGAACCAAG
TTCTGCCATCTGCTTGCACCCGGGG
>LOC105803850b_Exon900_10
ACAGAGCCACTATCATGCTTATTCCAGCTGAAGAGTGCTACAGAAGATACCCAAGTTGAACCAAGTTCTGCCATC
TGCTTGCACCCGGGGTGTGGTGATT
>LOC105803850b_Exon900_20
TATCATGCTTATTCCAGCTGAAGAGTGCTACAGAAGATACCCAAGTTGAACCAAGTTCTGCCATCTGCTTGCACC
CGGGGTGTGGTGATTACCATGTCTT
>LOC105803850b_Exon1200_0
GCTTTTAGTTTCCCAGAGACTGAAGGTGATGCCCTTTTGCTGGAAGTCCAAGACAAGAAGAAATTAGTCCAAGGC
CGAGCAACAATACCGGTTTCATCTT
>LOC105803850b_Exon1200_10
TCCCAGAGACTGAAGGTGATGCCCTTTTGCTGGAAGTCCAAGACAAGAAGAAATTAGTCCAAGGCCGAGCAACAA
TACCGGTTTCATCTTTGAGTGACAA
>LOC105803850b_Exon1200_20
TGAAGGTGATGCCCTTTTGCTGGAAGTCCAAGACAAGAAGAAATTAGTCCAAGGCCGAGCAACAATACCGGTTTC
ATCTTTGAGTGACAATCCTGTATGT
>LOC105803850b_Exon1400_0
CTGCAGAACGAGAGAATCCGGTGGTGGCCCATATACCATGAGGATGAAGAATGTGTTGGGAAGATCCAGCTATCT
ATCGGAAGTACAATTACTTGCGATG
>LOC105803850b_Exon1400_10
AGAGAATCCGGTGGTGGCCCATATACCATGAGGATGAAGAATGTGTTGGGAAGATCCAGCTATCTATCGGAAGTA
CAATTACTTGCGATGAGACGAGTCA
>LOC105803850b_Exon1400_20
GTGGTGGCCCATATACCATGAGGATGAAGAATGTGTTGGGAAGATCCAGCTATCTATCGGAAGTACAATTACTTG
CGATGAGACGAGTCAGATAAAGGTA
>LOC105803850b_Exon1900_0
AGAGTGCGCCTGTTGTGGAGACTCTAGCCTACGATCTACTCCTCGAGGCTTCAATGCGCGCACAACGTTTTCATT
CCAGAAACTTGAGGTTACAGGGACC
>LOC105803850b_Exon1900_14
GTGGAGACTCTAGCCTACGATCTACTCCTCGAGGCTTCAATGCGCGCACAACGTTTTCATTCCAGAAACTTGAGG
TTACAGGGACCGTGGCAGTGGTTAT
>LOC105803850b_Exon1900_27
CCTACGATCTACTCCTCGAGGCTTCAATGCGCGCACAACGTTTTCATTCCAGAAACTTGAGGTTACAGGGACCGT
GGCAGTGGTTATTGACTGAGTTTGC
>LOC108773630_exon2700_0
GGTTTGGAGTTGCGCTTAAAGGGCGATGGTCGTCGGTATAAACTAATTATTCGCACTAGCACCGATTGGGATACT
GTGGGTTATACAGCAAGCTTTGACA
>LOC108773630_exon2700_8
GTTGCGCTTAAAGGGCGATGGTCGTCGGTATAAACTAATTATTCGCACTAGCACCGATTGGGATACTGTGGGTTA
TACAGCAAGCTTTGACACCGTTGGA
>LOC108773630_exon2700_23
CGATGGTCGTCGGTATAAACTAATTATTCGCACTAGCACCGATTGGGATACTGTGGGTTATACAGCAAGCTTTGA
CACCGTTGGAGGCCAATGGCAGTCT

>LOC108773630_exon2900_0
TTACAGATTCGTTTGCCATTCTCTACACTGAGGCCTATATTTCGAGCACGAACTGTATCTGATGCCCCACCCTTC
GATCCAAGCAATGTTGTGTCATTTC
>LOC108773630_exon2900_4
AGATTCGTTTGCCATTCTCTACACTGAGGCCTATATTTCGAGCACGAACTGTATCTGATGCCCCACCCTTCGATC
CAAGCAATGTTGTGTCATTTCAGGT
>LOC108773630_exon3000_0
CAGCTCATGTTCAGCAAGTTTGAATATGATGGGAAACTGAATCCCACTTTTGTAGAAGGGCCTTTTGAGCTTCCA
CTCTCGAGCATAAAAGCATACATGA
>LOC108773630_exon3000_11
CAGCAAGTTTGAATATGATGGGAAACTGAATCCCACTTTTGTAGAAGGGCCTTTTGAGCTTCCACTCTCGAGCAT
AAAAGCATACATGAAGGATCCTATC
>LOC108773630_exon3000_21
GAATATGATGGGAAACTGAATCCCACTTTTGTAGAAGGGCCTTTTGAGCTTCCACTCTCGAGCATAAAAGCATAC
ATGAAGGATCCTATCACTCCCAGGT
>LOC108773630_exon3330_0
AGGTTTGTACATGTTAGTTCTGCTGGAGTTACAAGACCGGACAGGCCTGGCATTGACCTGAGCAAACAACCTCCT
GCTGTGCGCTTGAATAAGGAATTGG
>LOC108773630_exon3330_16
GTTCTGCTGGAGTTACAAGACCGGACAGGCCTGGCATTGACCTGAGCAAACAACCTCCTGCTGTGCGCTTGAATA
AGGAATTGGATTTCGTCCTAACATT
>LOC108773630_exon3330_24
GGAGTTACAAGACCGGACAGGCCTGGCATTGACCTGAGCAAACAACCTCCTGCTGTGCGCTTGAATAAGGAATTG
GATTTCGTCCTAACATTCAAGTTGA
>LOC108773630_exon4000_0
AGGGGGAAGATTTGATACGTGAGAGTGGAATTCCTTATGCAATCGTAAGGCCTTGTGCATTAACTGAAGAGCCTG
CCGGAGCAGATCTCGTTTTTGATCA
>LOC108773630_exon4000_10
TTTGATACGTGAGAGTGGAATTCCTTATGCAATCGTAAGGCCTTGTGCATTAACTGAAGAGCCTGCCGGAGCAGA
TCTCGTTTTTGATCAAGGAGACAAT
>LOC108773630_exon4000_20
GAGAGTGGAATTCCTTATGCAATCGTAAGGCCTTGTGCATTAACTGAAGAGCCTGCCGGAGCAGATCTCGTTTTT
GATCAAGGAGACAATATAACGGTAT
>LRR_At1g67720_exon70_0
AACCAACTGTTCCTCGTGCCACACTTGATACATGGGTTAAATCTTCCTCAGCTTGTCTTGAAAGTCCAAAATCAG
ACACTTTAGCTCTCATGTTAATGTC
>LRR_At1g67720_exon70_18
CCACACTTGATACATGGGTTAAATCTTCCTCAGCTTGTCTTGAAAGTCCAAAATCAGACACTTTAGCTCTCATGT
TAATGTCTAGAAGAATGTTGCTTGT
>LRR_At1g67720_exon70_36
TTAAATCTTCCTCAGCTTGTCTTGAAAGTCCAAAATCAGACACTTTAGCTCTCATGTTAATGTCTAGAAGAATGT
TGCTTGTTTTTACATCTCGGTGAAT
>LRR_At1g67720_exon480_0
AACCATGTATGTGATCCCTCAAAGTTCCATTGTGCATATATTCATAAACTAATATCCGTTGATGAGCTTCTTCGC
AGTATCCAATTAGAGGAACCAAGTT
>LRR_At1g67720_exon480_14
TCCCTCAAAGTTCCATTGTGCATATATTCATAAACTAATATCCGTTGATGAGCTTCTTCGCAGTATCCAATTAGA
GGAACCAAGTTTCTATGGTGAATTC
>LRR_At1g67720_exon480_28
ATTGTGCATATATTCATAAACTAATATCCGTTGATGAGCTTCTTCGCAGTATCCAATTAGAGGAACCAAGTTTCT
ATGGTGAATTCTTGATAAGAGGGCA
>LRR_At1g67720_exon1400_0
TTTCCATCTTTCATTTTGCCATAGTAGACAGATCCAAAACTTCCCTTGCCAATCTTTTTGGTGAAATTGTTAGTT
GCTTCCTCTAGATCAGACAGTGAGA
>LRR_At1g67720_exon1400_17
GCCATAGTAGACAGATCCAAAACTTCCCTTGCCAATCTTTTTGGTGAAATTGTTAGTTGCTTCCTCTAGATCAGA
CAGTGAGATACAGTATGCCACACCT

>LRR_At1g67720_exon1400_34
CAAAACTTCCCTTGCCAATCTTTTTGGTGAAATTGTTAGTTGCTTCCTCTAGATCAGACAGTGAGATACAGTATG
CCACACCTTCGTCCAGTAAATGCCC
>LRR_At1g67720_exon1700_0
GGTGAGACATCTTCCTTCGGAAATTACGCAATAATATTAAACTTGCCAAGAAAAGAATTAATAGAACTGCCAGCA
CTCCGATTGAGGCTCCAAGTATCAA
>LRR_At1g67720_exon1700_8
ATCTTCCTTCGGAAATTACGCAATAATATTAAACTTGCCAAGAAAAGAATTAATAGAACTGCCAGCACTCCGATT
GAGGCTCCAAGTATCAACTTAAAAC
>LRR_At1g67720_exon1700_23
TTACGCAATAATATTAAACTTGCCAAGAAAAGAATTAATAGAACTGCCAGCACTCCGATTGAGGCTCCAAGTATC
AACTTAAAACGCAGTTTTCTCTGTG
>LysoBglucosidase_exon80_0
TTGTTTACTCTTGAGGATTCTTCCCCTTATTGTATGTCAATCCAAACCCTAGGGGAAACAAAGGGTCATATGAAT
CATGTCTGGGATTCACTGATAGCTG
>LysoBglucosidase_exon80_17
TTCTTCCCCTTATTGTATGTCAATCCAAACCCTAGGGGAAACAAAGGGTCATATGAATCATGTCTGGGATTCACT
GATAGCTGGTCGGTGCTTCTAAACC
>LysoBglucosidase_exon80_33
ATGTCAATCCAAACCCTAGGGGAAACAAAGGGTCATATGAATCATGTCTGGGATTCACTGATAGCTGGTCGGTGC
TTCTAAACCAAGTCATTGGTAATCG
>LysoBglucosidase_exon450_0
AAGGTTTCGGCATAAGGTGGTTCACCTACAACTACAATGGCAAAAGAGTACTGTCCTGCTAAGGAATTTGTTGAG
GGATACTGATCATAGATCACCTCCG
>LysoBglucosidase_exon450_20
TTCACCTACAACTACAATGGCAAAAGAGTACTGTCCTGCTAAGGAATTTGTTGAGGGATACTGATCATAGATCAC
CTCCGTGCTGCCTCCTACAGATTCT
>LysoBglucosidase_exon450_40
CAAAAGAGTACTGTCCTGCTAAGGAATTTGTTGAGGGATACTGATCATAGATCACCTCCGTGCTGCCTCCTACAG
ATTCTCTAATGGCATCCAAGATGGT
>LysoBglucosidase_exon870_0
CCAGCAACAAGAATTCTTTTAGCAGTCCTGTCTAATGGAAGAAAAGGGTTTTCAGGGTCCTTTCCATTTTTTAAT
AGAACCAAGGACTTCCGAACTGCTT
>LysoBglucosidase_exon870_11
AATTCTTTTAGCAGTCCTGTCTAATGGAAGAAAAGGGTTTTCAGGGTCCTTTCCATTTTTTAATAGAACCAAGGA
CTTCCGAACTGCTTCACGAGCTAGT
>LysoBglucosidase_exon870_22
CAGTCCTGTCTAATGGAAGAAAAGGGTTTTCAGGGTCCTTTCCATTTTTTAATAGAACCAAGGACTTCCGAACTG
CTTCACGAGCTAGTTCCCTGTGTAG
>LysoBglucosidase_exon1300_0
AACAAATTTCACTCTCAATATTCGTTCAACAGCATCATCGATCCTGGACATCGGTACCTCCCCAGATTCAACTAG
AAAGGTCAAATCCTCCATGAACTGT
>LysoBglucosidase_exon1300_10
ACTCTCAATATTCGTTCAACAGCATCATCGATCCTGGACATCGGTACCTCCCCAGATTCAACTAGAAAGGTCAAA
TCCTCCATGAACTGTTTATATCTGT
>LysoBglucosidase_exon1300_20
TTCGTTCAACAGCATCATCGATCCTGGACATCGGTACCTCCCCAGATTCAACTAGAAAGGTCAAATCCTCCATGA
ACTGTTTATATCTGTGAGGTACCAT
>LysoPro-X_exon450_0
TCCCGTTCCGGTCCCATCTTTCTCTACTGCGGCAACGAAGGTGACATCGATTGGTTCGCCGTCAACACCGGCTTT
GTCTGGGACATTGCACCGCGCTTCG
>LysoPro-X_exon450_11
TCCCATCTTTCTCTACTGCGGCAACGAAGGTGACATCGATTGGTTCGCCGTCAACACCGGCTTTGTCTGGGACAT
TGCACCGCGCTTCGGTGCTATGATT
>LysoPro-X_exon450_21
CTCTACTGCGGCAACGAAGGTGACATCGATTGGTTCGCCGTCAACACCGGCTTTGTCTGGGACATTGCACCGCGC
TTCGGTGCTATGATTCTGTTTCCAG

>LysoPro-X_exon1100_0
AGCATCGGTATTATGGGGAGTCAATGCCTTTTGGAAGTAGAGAAGAGGCTTACAGAAATGCCACTACTCTTTCTT
ATCTCACAGCAGAGCAAGCGCTTGC
>LysoPro-X_exon1100_23
ATGCCTTTTGGAAGTAGAGAAGAGGCTTACAGAAATGCCACTACTCTTTCTTATCTCACAGCAGAGCAAGCGCTT
GCTGATTTCGCACTTCTCATTACTG
>LysoPro-X_exon1100_45
AGGCTTACAGAAATGCCACTACTCTTTCTTATCTCACAGCAGAGCAAGCGCTTGCTGATTTCGCACTTCTCATTA
CTGATTTGAAGAAGAATTTGTCTGC
>LysoPro-X_exon1500_0
TGTTAGCCGCATGGATGAGGCTCAAGTATCCTCATATTGCTGTCGGGGCGCTTGCTTCATCTGCTCCGATTCTTC
AGTTTGAAGATATTGTGCCACCGGA
>LysoPro-X_exon1500_16
GAGGCTCAAGTATCCTCATATTGCTGTCGGGGCGCTTGCTTCATCTGCTCCGATTCTTCAGTTTGAAGATATTGT
GCCACCGGAAACATTTTATAATATT
>LysoPro-X_exon1500_32
CATATTGCTGTCGGGGCGCTTGCTTCATCTGCTCCGATTCTTCAGTTTGAAGATATTGTGCCACCGGAAACATTT
TATAATATTGTTTCCAATTCATTCA
>LysoPro-X_exon1730_0
GTAGCGGGAAAGTAGTAGCTGTTTTGACACTATTAAAAATTCATGGAGTGCACTGACATCTGAGGGTCATAAGGA
TGATGGCCTTCTGCAACTGTCAAAA
>LysoPro-X_exon1730_9
AAGTAGTAGCTGTTTTGACACTATTAAAAATTCATGGAGTGCACTGACATCTGAGGGTCATAAGGATGATGGCCT
TCTGCAACTGTCAAAAACTTTTCGC
>LysoPro-X_exon1730_18
CTGTTTTGACACTATTAAAAATTCATGGAGTGCACTGACATCTGAGGGTCATAAGGATGATGGCCTTCTGCAACT
GTCAAAAACTTTTCGCTTGTGTCGG
>LysoPro-X_exon1990_0
GGAAACTAAAGAGCGTCCAAGCCCTAGCAGACTGGTTGGATTCTGCTTATAGTTATTTGGCAATGGTTAATTACC
CGTATCCTTCAAATTTTTTAATGCC
>LysoPro-X_exon1990_8
AAGAGCGTCCAAGCCCTAGCAGACTGGTTGGATTCTGCTTATAGTTATTTGGCAATGGTTAATTACCCGTATCCT
TCAAATTTTTTAATGCCTTTGCCAG
>LysoPro-X_exon1990_23
CTAGCAGACTGGTTGGATTCTGCTTATAGTTATTTGGCAATGGTTAATTACCCGTATCCTTCAAATTTTTTAATG
CCTTTGCCAGGATATCCCATAAAAG
>LysoPro-X_exon2880_0
GATTGATGGTTCTCCTGACGATTCTAGTATTCTGGAGCGTTTATTCAATGGAGTAAGCGTGTATTACAATTATAC
TGGTGAGGTTGAATGTTTTCAACTT
>LysoPro-X_exon2880_19
GATTCTAGTATTCTGGAGCGTTTATTCAATGGAGTAAGCGTGTATTACAATTATACTGGTGAGGTTGAATGTTTT
CAACTTGATGATGATCCTCATGGCA
>LysoPro-X_exon2880_37
CGTTTATTCAATGGAGTAAGCGTGTATTACAATTATACTGGTGAGGTTGAATGTTTTCAACTTGATGATGATCCT
CATGGCATGGATGGGTGGAACTGGC
>LysoPro-X_exon3150_0
AGGCATGCACTGAGATGGTTATGCCAATGTCTAGTGATCGAAATACTAGCATGTTTCCAGCATACGATTGGGATT
ACTCTGCTTTCCGAGAAGGGTGCAG
>LysoPro-X_exon3150_20
ATGCCAATGTCTAGTGATCGAAATACTAGCATGTTTCCAGCATACGATTGGGATTACTCTGCTTTCCGAGAAGGG
TGCAGGATGGATTTCCAGGTGACAC
>LysoPro-X_exon3150_39
GAAATACTAGCATGTTTCCAGCATACGATTGGGATTACTCTGCTTTCCGAGAAGGGTGCAGGATGGATTTCCAGG
TGACACCAAGGCCTAGATGGATAAC
>MAP3K_Exon60a_0
CACAAGAACCTTCTCGGGCTTCAACTTCTCTTTCTCAGAGGTCAGATGCCTATCTGCCGGATTCGCGTTATCTTG
CTGTCGACAGCGACAGAACTCAATT

>MAP3K_Exon60a_23
ACTTCTCTTTCTCAGAGGTCAGATGCCTATCTGCCGGATTCGCGTTATCTTGCTGTCGACAGCGACAGAACTCAA
TTCAGCAGTGGAGCTGTGGATGTTT
>MAP3K_Exon60a_45
ATGCCTATCTGCCGGATTCGCGTTATCTTGCTGTCGACAGCGACAGAACTCAATTCAGCAGTGGAGCTGTGGATG
TTTCAGTTGGTTCTAAATTGGCAGA
>MAP3K_Exon450a_0
AATGAACCTCCTTCTAGACATATCTCTGATCAATTGGAGTATGTTCGCCACCTGCCAGGATCAGAGAGACATGAA
AGCATACTGCCTCTTTTACATGCTA
>MAP3K_Exon450a_27
GATCAATTGGAGTATGTTCGCCACCTGCCAGGATCAGAGAGACATGAAAGCATACTGCCTCTTTTACATGCTAAT
AATGATAGGAAAGCCAATGGTGAAT
>MAP3K_Exon450a_54
CCAGGATCAGAGAGACATGAAAGCATACTGCCTCTTTTACATGCTAATAATGATAGGAAAGCCAATGGTGAATTA
GACTTCTTGATGGCTGAATTTGCAG
>MAP3K_Exon1100_0
ATCTGGTATAGCTTCTCAGACAGCATCAGGTGTATTATCTGGCTCAGGTGTTTTAAATGCTAGACCAGGAAGCAC
GACATCATCAGGGTTACTCTCCAAC
>MAP3K_Exon1100_25
TCAGGTGTATTATCTGGCTCAGGTGTTTTAAATGCTAGACCAGGAAGCACGACATCATCAGGGTTACTCTCCAAC
ATGGTATCAACAATGAATACGGATG
>MAP3K_Exon1100_50
TTTTAAATGCTAGACCAGGAAGCACGACATCATCAGGGTTACTCTCCAACATGGTATCAACAATGAATACGGATG
TTGCCAGGGAGTACCTGGAAAAGGT
>MAP3K_Exon1400_0
AGATACTGAAGTGCATTAATCATTTGTCCACTGATCCAAACTGCTTGGAGAATCTTCAACGAGCAGATGCAATTA
AGTATTTGATCCCAAATCTAGAACT
>MAP3K_Exon1400_18
ATCATTTGTCCACTGATCCAAACTGCTTGGAGAATCTTCAACGAGCAGATGCAATTAAGTATTTGATCCCAAATC
TAGAACTTGAATGTGGACCTCTTGT
>MAP3K_Exon1400_35
CCAAACTGCTTGGAGAATCTTCAACGAGCAGATGCAATTAAGTATTTGATCCCAAATCTAGAACTTGAATGTGGA
CCTCTTGTATCTCAGATGCATCATG
>Mechanosensitive_exon230_0
TCGCCGTAGTTTTGGTGGTTCATTGTGTGCAGAACACAAAGAACCAGCTTCATCTTGTCCATGTTCTCAATCTCC
TTGAATAGCAATGTGTGTTTTGGAC
>Mechanosensitive_exon230_9
TTTTGGTGGTTCATTGTGTGCAGAACACAAAGAACCAGCTTCATCTTGTCCATGTTCTCAATCTCCTTGAATAGC
AATGTGTGTTTTGGACTCCAATATT
>Mechanosensitive_exon230_26
GTGCAGAACACAAAGAACCAGCTTCATCTTGTCCATGTTCTCAATCTCCTTGAATAGCAATGTGTGTTTTGGACT
CCAATATTTTGGCTTGCTCTCAATA
>Mechanosensitive_exon440_0
TACAATTGTATAGCTTTCTTGAGAGCATTGATGTCTTCAACCGGAGTAGACACATCGATGGTGAAATCAACAGTG
TCTCCCATGTCGGGGCTTCTTCTGA
>Mechanosensitive_exon440_11
AGCTTTCTTGAGAGCATTGATGTCTTCAACCGGAGTAGACACATCGATGGTGAAATCAACAGTGTCTCCCATGTC
GGGGCTTCTTCTGAAGTTGCTAATA
>Mechanosensitive_exon440_21
AGAGCATTGATGTCTTCAACCGGAGTAGACACATCGATGGTGAAATCAACAGTGTCTCCCATGTCGGGGCTTCTT
CTGAAGTTGCTAATAGGCTTTGTAA
>Mechanosensitive_exon900_0
AGGAGGGAGACCACGATTATGATCACAATAACGACTGCACTTGCTAATCTGTGCAGCTGTTGGACTGCTGTTTTG
GTGTCGTTTAAGGAATGAGCCAAGG
>Mechanosensitive_exon900_11
CACGATTATGATCACAATAACGACTGCACTTGCTAATCTGTGCAGCTGTTGGACTGCTGTTTTGGTGTCGTTTAA
GGAATGAGCCAAGGCTTTGCGCTCC

>Mechanosensitive_exon900_22
TCACAATAACGACTGCACTTGCTAATCTGTGCAGCTGTTGGACTGCTGTTTTGGTGTCGTTTAAGGAATGAGCCA
AGGCTTTGCGCTCCACATATGCATG
>Mechanosensitive_exon1460_0
TACCACCCAATTTCTGAAGGAAGATTTAGAGATTTTTCCAGTCTCAAGGGCTCCTTCAAGGAGAGGAAATATAGT
ATGAACTTCTTCATTACTCAAGAAC
>Mechanosensitive_exon1460_8
AATTTCTGAAGGAAGATTTAGAGATTTTTCCAGTCTCAAGGGCTCCTTCAAGGAGAGGAAATATAGTATGAACTT
CTTCATTACTCAAGAACTTCAATAG
>Mechanosensitive_exon1460_24
TTTAGAGATTTTTCCAGTCTCAAGGGCTCCTTCAAGGAGAGGAAATATAGTATGAACTTCTTCATTACTCAAGAA
CTTCAATAGATCTTCCTCTTCTATA
>MNS4_exon700_0
AGAAACGACAACTATAGCATGGCTCTGAACCACAGTATCTTTTTGTTTAGCAGAATCATCTTCACTAGGTTTATC
AACTGAAGCTAGATACGAAATGCCA
>MNS4_exon700_17
CATGGCTCTGAACCACAGTATCTTTTTGTTTAGCAGAATCATCTTCACTAGGTTTATCAACTGAAGCTAGATACG
AAATGCCATACTTCTGCGCATGAGT
>MNS4_exon700_34
GTATCTTTTTGTTTAGCAGAATCATCTTCACTAGGTTTATCAACTGAAGCTAGATACGAAATGCCATACTTCTGC
GCATGAGTTAGTCCCGGACAAAGGC
>MNS4_exon1250_0
CCTTAATCAGCCCTGATACGGGAGATGACTCGAAAGAAGAAGAGTCCAAAGGAGATCCCGTGCGAACCCAACCGT
CAGAAACTCTACTACCATTGGTTTC
>MNS4_exon1250_30
CGAAAGAAGAAGAGTCCAAAGGAGATCCCGTGCGAACCCAACCGTCAGAAACTCTACTACCATTGGTTTCTTGAG
AATCAGAAGACTTGTCCGATGCACG
>MNS4_exon1250_60
TGCGAACCCAACCGTCAGAAACTCTACTACCATTGGTTTCTTGAGAATCAGAAGACTTGTCCGATGCACGAGGCT
CTTCTTTTAAGCTGCCATGGTAACA
>MNS4_exon1250_90
CATTGGTTTCTTGAGAATCAGAAGACTTGTCCGATGCACGAGGCTCTTCTTTTAAGCTGCCATGGTAACAATAAG
CCCCCAAATATGCACAATGCTCTTG
>MNS4_exon1250_120
CCGATGCACGAGGCTCTTCTTTTAAGCTGCCATGGTAACAATAAGCCCCCAAATATGCACAATGCTCTTGTAATA
GGGATATTTGAGGAGTTGCAGGCAA
>MNS4_exon1250_146
CTGCCATGGTAACAATAAGCCCCCAAATATGCACAATGCTCTTGTAATAGGGATATTTGAGGAGTTGCAGGCAAC
AAATGGCCCTCAGTGCTAAATATGT
>MNS4_exon1850_0
ACTGTTTCAGCCAGGAAAAAGCTTTCCATGTGATCCTCCCTCTTGTGATTCTCGACATCTGATATATGGCAATAA
CCACATGGGCACCTAGCCCCATATT
>MNS4_exon1850_19
AGCTTTCCATGTGATCCTCCCTCTTGTGATTCTCGACATCTGATATATGGCAATAACCACATGGGCACCTAGCCC
CATATTGCAAGCTAGCAACTATGTC
>MNS4_exon1850_38
CCTCTTGTGATTCTCGACATCTGATATATGGCAATAACCACATGGGCACCTAGCCCCATATTGCAAGCTAGCAAC
TATGTCTCGTCCAACATCCAGATAT
>NECK1_exon340_0
TTGACCCGTAAAGACACGGAGGTGAAACTCCCTCGACCGACCCGTGTCAAAAACAAAACTCCGGCTCCAATACAG
ATTACCGCCGAACAAATCCTCCGAG
>NECK1_exon340_10
AAGACACGGAGGTGAAACTCCCTCGACCGACCCGTGTCAAAAACAAAACTCCGGCTCCAATACAGATTACCGCCG
AACAAATCCTCCGAGAAGCTCGGGA
>NECK1_exon340_20
GGTGAAACTCCCTCGACCGACCCGTGTCAAAAACAAAACTCCGGCTCCAATACAGATTACCGCCGAACAAATCCT
CCGAGAAGCTCGGGAACGGCAGGAG

>NECK1_exon700_0
ATGAAGAACAAGTTCATCAACCACGCAAGGAATGTTTGGGACCGTGCTGTCACTCTCCTCCCGCGTGTGGACCAG
CTCTGGTACAAGTACATCCACATGG
>NECK1_exon700_10
AGTTCATCAACCACGCAAGGAATGTTTGGGACCGTGCTGTCACTCTCCTCCCGCGTGTGGACCAGCTCTGGTACA
AGTACATCCACATGGAAGAGATGTT
>NECK1_exon700_20
CCACGCAAGGAATGTTTGGGACCGTGCTGTCACTCTCCTCCCGCGTGTGGACCAGCTCTGGTACAAGTACATCCA
CATGGAAGAGATGTTAGGGAATGTC
>NECK1_exon1100_0
TTTGAGGAACGGTGTAAGGAATCAGAACGTGCTCGTTGTATCTACAAGTTTGCTCTCGACCATATTCCCAAAGGG
AGGGCAGAGGATTTGTACAGAAAGT
>NECK1_exon1100_10
GGTGTAAGGAATCAGAACGTGCTCGTTGTATCTACAAGTTTGCTCTCGACCATATTCCCAAAGGGAGGGCAGAGG
ATTTGTACAGAAAGTTTGTGGCTTT
>NECK1_exon1100_20
ATCAGAACGTGCTCGTTGTATCTACAAGTTTGCTCTCGACCATATTCCCAAAGGGAGGGCAGAGGATTTGTACAG
AAAGTTTGTGGCTTTTGAGAAACAA
>NECK1_exon1330_0
ATATAAGATTGGAAGACAGCGTAGGGAATAAAGGGAGGATTAGGGAGGTTTACGAGAGGGCTATTGCTAATGTTC
CTCCAGCCGAGGAGAAGCGATATTG
>NECK1_exon1330_10
GGAAGACAGCGTAGGGAATAAAGGGAGGATTAGGGAGGTTTACGAGAGGGCTATTGCTAATGTTCCTCCAGCCGA
GGAGAAGCGATATTGGCAAAGATAT
>NECK1_exon1330_20
GTAGGGAATAAAGGGAGGATTAGGGAGGTTTACGAGAGGGCTATTGCTAATGTTCCTCCAGCCGAGGAGAAGCGA
TATTGGCAAAGATATATTTACTTGT
>NECK1_exon2330_0
AGGGAGTGCCTTAAGCTTATTCCCCACGAGAAGTTTTCTTTTGCAAAGATCTGGCTTCTTGCTGCGCAATTTGAA
ATAAGACAACTCAATCTCAAGGGTG
>NECK1_exon2330_10
TTAAGCTTATTCCCCACGAGAAGTTTTCTTTTGCAAAGATCTGGCTTCTTGCTGCGCAATTTGAAATAAGACAAC
TCAATCTCAAGGGTGCACGACAAAT
>NECK1_exon2330_20
TCCCCACGAGAAGTTTTCTTTTGCAAAGATCTGGCTTCTTGCTGCGCAATTTGAAATAAGACAACTCAATCTCAA
GGGTGCACGACAAATTTTGGGAAAT
>NECK1_exon2600_0
GAGTGGGCACCTGAGAACTGCTATGCATGGAGCAAATATGCCGAGTTGGAAAGATCATTATCTGAAACAGAGCGG
GCAAGAGCTATTTTTGAGCTTGCCA
>NECK1_exon2600_10
CTGAGAACTGCTATGCATGGAGCAAATATGCCGAGTTGGAAAGATCATTATCTGAAACAGAGCGGGCAAGAGCTA
TTTTTGAGCTTGCCATCACACAACC
>NECK1_exon2600_20
CTATGCATGGAGCAAATATGCCGAGTTGGAAAGATCATTATCTGAAACAGAGCGGGCAAGAGCTATTTTTGAGCT
TGCCATCACACAACCAGCTTTGGAC
>NF-X1-zinc_exon2840_0
AGGTGTCATGGGCCTAGACCTCCTCCTAATCCAGAATTCACCTTGAAACCCAAAAAAAAGAAATCATATCATCAG
CCTGAGTGCACTCCTGGCACGCCTT
>NF-X1-zinc_exon2840_10
GGCCTAGACCTCCTCCTAATCCAGAATTCACCTTGAAACCCAAAAAAAAGAAATCATATCATCAGCCTGAGTGCA
CTCCTGGCACGCCTTGCCCTCCTTG
>NF-X1-zinc_exon2840_20
TCCTCCTAATCCAGAATTCACCTTGAAACCCAAAAAAAAGAAATCATATCATCAGCCTGAGTGCACTCCTGGCAC
GCCTTGCCCTCCTTGCCCAGAACTT
>NF-X1-zinc_exon3140_0
GATGGTTTGCTCCAATACAACACGGTTTTCTTGTGATAATTTGTGTGGAAATCTTCTTCCTTGTGGCAATCATTA
TTGTACGAAAACTTGCCATCCCCTG

>NF-X1-zinc_exon3140_14
ATACAACACGGTTTTCTTGTGATAATTTGTGTGGAAATCTTCTTCCTTGTGGCAATCATTATTGTACGAAAACTT
GCCATCCCCTGGAGACACAGCCTTC
>NF-X1-zinc_exon3140_28
TCTTGTGATAATTTGTGTGGAAATCTTCTTCCTTGTGGCAATCATTATTGTACGAAAACTTGCCATCCCCTGGAG
ACACAGCCTTCTTCATCAGGACATC
>NF-X1-zinc_exon3570_0
ATGTCATCCCGGAGAATGCCCTCCTTGCAAAGTGCTCGTGAAACGGTCATGTCACTGTGGTGCAATGGTCCATGC
ATTTGAGTGCATATATTATAACAAT
>NF-X1-zinc_exon3570_15
ATGCCCTCCTTGCAAAGTGCTCGTGAAACGGTCATGTCACTGTGGTGCAATGGTCCATGCATTTGAGTGCATATA
TTATAACAATTTATCTGAAAAGGAT
>NF-X1-zinc_exon3570_30
AGTGCTCGTGAAACGGTCATGTCACTGTGGTGCAATGGTCCATGCATTTGAGTGCATATATTATAACAATTTATC
TGAAAAGGATCAAGTGGCTGCTCGC
>NF-X1-zinc_exon4200_0
TGGTCATGATCCCAAAGATATACCAAAGAATCAGTTTGGACTCGGACTTCTGCCTTGCAATTCGGATTGTAAGAG
CAAAGTACAGGAGGTTGAGTCAGCT
>NF-X1-zinc_exon4200_9
TCCCAAAGATATACCAAAGAATCAGTTTGGACTCGGACTTCTGCCTTGCAATTCGGATTGTAAGAGCAAAGTACA
GGAGGTTGAGTCAGCTCTACAATTG
>NF-X1-zinc_exon4200_25
AAGAATCAGTTTGGACTCGGACTTCTGCCTTGCAATTCGGATTGTAAGAGCAAAGTACAGGAGGTTGAGTCAGCT
CTACAATTGCGTAAACCTAAGGTTC
>NOB1_exon480_0
ACTGGTGACTTGCAGACTCTTTCTGATGTCGATCTTAAGCTGATTGCCTTGACTTATACGTTGGAGTCCCAAATT
CATGGAGCAAATCATCTTAGGGATG
>NOB1_exon480_14
GACTCTTTCTGATGTCGATCTTAAGCTGATTGCCTTGACTTATACGTTGGAGTCCCAAATTCATGGAGCAAATCA
TCTTAGGGATGCCCCTCCCCCTGTT
>NOB1_exon480_28
TCGATCTTAAGCTGATTGCCTTGACTTATACGTTGGAGTCCCAAATTCATGGAGCAAATCATCTTAGGGATGCCC
CTCCCCCTGTTCATGTTGTTAATGT
>NOB1_exon900_0
GAAGGGAAAAAGATGGTGGCTCATGGCATTGATGCATCTCAGGGGCAGATTGATGATAACAGTGATGATTGGCTA
CCTGCTGTAAGTCGAAGTACTCACA
>NOB1_exon900_17
GGCTCATGGCATTGATGCATCTCAGGGGCAGATTGATGATAACAGTGATGATTGGCTACCTGCTGTAAGTCGAAG
TACTCACAGGAGATTCCTTAGGAGG
>NOB1_exon900_33
GCATCTCAGGGGCAGATTGATGATAACAGTGATGATTGGCTACCTGCTGTAAGTCGAAGTACTCACAGGAGATTC
CTTAGGAGGAAAGCTAGGCGGGAAT
>NOB1_exon1440_0
GAACAGAGCTGGATGTTGAGATCCTTATCTGAGTCCACTGTAGCTTGTGTAACTGGGGACTTTGCCATGCAAAAT
GTTCTTCTGCAGATTGGTTTAAGAT
>NOB1_exon1440_19
GATCCTTATCTGAGTCCACTGTAGCTTGTGTAACTGGGGACTTTGCCATGCAAAATGTTCTTCTGCAGATTGGTT
TAAGATTACTAGCTCCAGGTGGAAT
>NOB1_exon1440_38
TGTAGCTTGTGTAACTGGGGACTTTGCCATGCAAAATGTTCTTCTGCAGATTGGTTTAAGATTACTAGCTCCAGG
TGGAATGCAGATTAGGCAGCTGCAC
>NOB1_exon1700_0
TGCTATACTGTGACTGCTGAAATTGGAAGAATTTTCTGTCCCAAGTGTGGAAATGGAGGAACTTTGCGGAAGGTA
GCTGTTACCGTTGGTGAGAATGGAA
>NOB1_exon1700_14
TGCTGAAATTGGAAGAATTTTCTGTCCCAAGTGTGGAAATGGAGGAACTTTGCGGAAGGTAGCTGTTACCGTTGG
TGAGAATGGAATTGTTTTGGCGTCC

>NOB1_exon1700_28
GAATTTTCTGTCCCAAGTGTGGAAATGGAGGAACTTTGCGGAAGGTAGCTGTTACCGTTGGTGAGAATGGAATTG
TTTTGGCGTCCCATCGCCCAAGAAT
>NOB1_exon1960_0
AATTTTCATTGCCTTTACCCCAAGGTGGAAGGGATGCCATAACCAAAAACCTCATTTTGCGTGAAGATCAACTTC
CCCAAAAGTTCCTTTACCCTAAAAC
>NOB1_exon1960_10
GCCTTTACCCCAAGGTGGAAGGGATGCCATAACCAAAAACCTCATTTTGCGTGAAGATCAACTTCCCCAAAAGTT
CCTTTACCCTAAAACTAAGAAGAAG
>NOB1_exon1960_20
CAAGGTGGAAGGGATGCCATAACCAAAAACCTCATTTTGCGTGAAGATCAACTTCCCCAAAAGTTCCTTTACCCT
AAAACTAAGAAGAAGGTTATTAAAC
>NOB1_exon2170_0
AGGGGGATGATGATCTCCTCATGGCTGGGGACACCTTCACTCACCATACCGACAAGAGGGCGCCTCTTCAGCCTC
CTGTTAGAAAAGCATTAGCGGTTTT
>NOB1_exon2170_14
CTCCTCATGGCTGGGGACACCTTCACTCACCATACCGACAAGAGGGCGCCTCTTCAGCCTCCTGTTAGAAAAGCA
TTAGCGGTTTTTAGTGGAAAAAGGA
>NOB1_exon2170_27
GGGACACCTTCACTCACCATACCGACAAGAGGGCGCCTCTTCAGCCTCCTGTTAGAAAAGCATTAGCGGTTTTTA
GTGGAAAAAGGAATCCAAACGACAA
>Ornithine_exon500_0
GCAGGAGGACCTATGGTGTTGTGGCTGAAGGCACCTCTTCTTCTTCCTCTTCCCCTTCTAACCATCTCATCAACC
TTGAACAGGAGTATAGTGCCCACAA
>Ornithine_exon500_10
CTATGGTGTTGTGGCTGAAGGCACCTCTTCTTCTTCCTCTTCCCCTTCTAACCATCTCATCAACCTTGAACAGGA
GTATAGTGCCCACAAGTTAGTTCTT
>Ornithine_exon500_20
GTGGCTGAAGGCACCTCTTCTTCTTCCTCTTCCCCTTCTAACCATCTCATCAACCTTGAACAGGAGTATAGTGCC
CACAAGTTAGTTCTTGTTAGTTCTT
>Ornithine_exon1220_0
TTACCACCCAGTTCCTGTTGTATTCTCGGAAGCAAAGGGATCGGCCATATGGGATCCAGAAGGCAAGAAATATCT
TGATTTTCTGTCTGCTTACTCTGCT
>Ornithine_exon1220_10
GTTCCTGTTGTATTCTCGGAAGCAAAGGGATCGGCCATATGGGATCCAGAAGGCAAGAAATATCTTGATTTTCTG
TCTGCTTACTCTGCTGTTAATCAGG
>Ornithine_exon1220_20
TATTCTCGGAAGCAAAGGGATCGGCCATATGGGATCCAGAAGGCAAGAAATATCTTGATTTTCTGTCTGCTTACT
CTGCTGTTAATCAGGTATAAACAGT
>Ornithine_exon1600_0
GAGCCTTCTACAATGACAAGTTTCCAGTTTTTGCTGAGCGTCTGACAAGTATGCTTGGGTACGAGATGGTGCTTC
CTATGAACACTGGTGCTGAAGGAGT
>Ornithine_exon1600_24
CAGTTTTTGCTGAGCGTCTGACAAGTATGCTTGGGTACGAGATGGTGCTTCCTATGAACACTGGTGCTGAAGGAG
TGGAGACTGCTCTGAAGTTGGCAAG
>Ornithine_exon1600_48
GTATGCTTGGGTACGAGATGGTGCTTCCTATGAACACTGGTGCTGAAGGAGTGGAGACTGCTCTGAAGTTGGCAA
GGAAATGGGGTTATGAGAAGAAAAA
>Ornithine_exon2200_0
GTCTCTTGTTGTGGTTGCTTCCATGGTCGCACATTGGCTGTCATCTCTATGAGCTGTGACAATGAGGCTACTCGT
GGATTTGGGCCATTATTGCCTGGTC
>Ornithine_exon2200_10
GTGGTTGCTTCCATGGTCGCACATTGGCTGTCATCTCTATGAGCTGTGACAATGAGGCTACTCGTGGATTTGGGC
CATTATTGCCTGGTCATGTTAAAGT
>Ornithine_exon2200_20
CCATGGTCGCACATTGGCTGTCATCTCTATGAGCTGTGACAATGAGGCTACTCGTGGATTTGGGCCATTATTGCC
TGGTCATGTTAAAGTTGATTTTGGT

>Ornithine_exon3200_0
TATAATTCCTCCAAAAGGTTATCTAAAAGCTGTCAGAGATCTTTGCTCGAAATATAACATTCTAATGATTGCTGA
TGAGATACAAAGTGGCCTAGCACGG
>Ornithine_exon3200_10
CCAAAAGGTTATCTAAAAGCTGTCAGAGATCTTTGCTCGAAATATAACATTCTAATGATTGCTGATGAGATACAA
AGTGGCCTAGCACGGTCGGGAAAAA
>Ornithine_exon3200_20
ATCTAAAAGCTGTCAGAGATCTTTGCTCGAAATATAACATTCTAATGATTGCTGATGAGATACAAAGTGGCCTAG
CACGGTCGGGAAAAATGTTGGCTTG
>Oxysterol-1D_exon100_0
TGAAGGTGAATTGCGACTGCTACTGCTACTCTGGCCGAATATATCAGGGATTCCATCCCATTTCCTTTTTTCTCT
TGCTTCCCAGTATCCTCCGATGTAT
>Oxysterol-1D_exon100_10
TTGCGACTGCTACTGCTACTCTGGCCGAATATATCAGGGATTCCATCCCATTTCCTTTTTTCTCTTGCTTCCCAG
TATCCTCCGATGTATCGATAACAAC
>Oxysterol-1D_exon100_20
TACTGCTACTCTGGCCGAATATATCAGGGATTCCATCCCATTTCCTTTTTTCTCTTGCTTCCCAGTATCCTCCGA
TGTATCGATAACAACCATCTTCATC
>Oxysterol-1D_exon670_0
CCTGTCTCTGTAACTGTTCCAGTCTCAGCTTCTCTGCATTTGCCATTTCATATTCTCCATTCTCCAAGTGTCTCT
GGTCTGGCCTTAGTCTCGAGTCCGT
>Oxysterol-1D_exon670_10
TAACTGTTCCAGTCTCAGCTTCTCTGCATTTGCCATTTCATATTCTCCATTCTCCAAGTGTCTCTGGTCTGGCCT
TAGTCTCGAGTCCGTCGGAGGCAAT
>Oxysterol-1D_exon670_20
AGTCTCAGCTTCTCTGCATTTGCCATTTCATATTCTCCATTCTCCAAGTGTCTCTGGTCTGGCCTTAGTCTCGAG
TCCGTCGGAGGCAATTTTTCCAATA
>Oxysterol-1D_exon900_0
GGGTCATACCCTTTCGGCTTCATAGTTGGATCTCCAAGAACATAATACATAGCTTCATCCCATTTGCCCACCAAC
ATGGCCACCTTTTCCCCAGTCCTAT
>Oxysterol-1D_exon900_10
CTTTCGGCTTCATAGTTGGATCTCCAAGAACATAATACATAGCTTCATCCCATTTGCCCACCAACATGGCCACCT
TTTCCCCAGTCCTATTGTCTTGCAC
>Oxysterol-1D_exon900_20
CATAGTTGGATCTCCAAGAACATAATACATAGCTTCATCCCATTTGCCCACCAACATGGCCACCTTTTCCCCAGT
CCTATTGTCTTGCACAAATCCATGA
>Oxysterol-1D_exon1400_0
ACCTGATGGGGATTTCGGTCTATGATAGACTGCTCCTTGAACTTGAGCTTGCAAGAGTAATTGCCACTGCCTTTT
ATGCGCATGGTACCATAGTGATCAC
>Oxysterol-1D_exon1400_10
GATTTCGGTCTATGATAGACTGCTCCTTGAACTTGAGCTTGCAAGAGTAATTGCCACTGCCTTTTATGCGCATGG
TACCATAGTGATCACAATAGATTTT
>Oxysterol-1D_exon1400_20
TATGATAGACTGCTCCTTGAACTTGAGCTTGCAAGAGTAATTGCCACTGCCTTTTATGCGCATGGTACCATAGTG
ATCACAATAGATTTTACCAAGGATA
>Oxysterol-1D_exon1600_0
ACACCAACTGGATCGAGTTGAATGGATCGCCCCCAAAATTTTCCTTTGAGATTGGAATCTGCCCAGAATTTCCAT
CCTCTACCCTCACAGTGACAAGCAA
>Oxysterol-1D_exon1600_10
GATCGAGTTGAATGGATCGCCCCCAAAATTTTCCTTTGAGATTGGAATCTGCCCAGAATTTCCATCCTCTACCCT
CACAGTGACAAGCAACAACCATGGG
>Oxysterol-1D_exon1600_20
AATGGATCGCCCCCAAAATTTTCCTTTGAGATTGGAATCTGCCCAGAATTTCCATCCTCTACCCTCACAGTGACA
AGCAACAACCATGGGGTGGTGACTC
>Oxysterol-1D_exon1900_0
TCCTTTATCTGGATAGTCAGCCTCATAGGTCTCCCCAAGTAATGGATTGAATGGTTTGCACTGCCGACCTTCTGT
CGATGCATAACCAGATACAGCAAAT

>Oxysterol-1D_exon1900_10
GGATAGTCAGCCTCATAGGTCTCCCCAAGTAATGGATTGAATGGTTTGCACTGCCGACCTTCTGTCGATGCATAA
CCAGATACAGCAAATGCCGCGACAT
>Oxysterol-1D_exon1900_20
CCTCATAGGTCTCCCCAAGTAATGGATTGAATGGTTTGCACTGCCGACCTTCTGTCGATGCATAACCAGATACAG
CAAATGCCGCGACATTTAGAATTCT
>PDIL5_2a_exon3500_0
TGAAGCTATTGAAAGATGAGGACAGGAAAATTGTTCTGACAATCATAGCTGATGAGAATGAAGACCAATCACAGA
ACTTGATCAAGTTATTGAGAGCTGC
>PDIL5_2a_exon3500_35
CTGACAATCATAGCTGATGAGAATGAAGACCAATCACAGAACTTGATCAAGTTATTGAGAGCTGCTGCTTCTGCA
AACCGTGATTTGGTATTTAGTTATG
>PDIL5_2a_exon3500_70
ACAGAACTTGATCAAGTTATTGAGAGCTGCTGCTTCTGCAAACCGTGATTTGGTATTTAGTTATGTTGGAGTTAA
GCAATGGGAAGACTTTGCTGATAAA
>PDIL5_2a_exon3500_105
CTGCAAACCGTGATTTGGTATTTAGTTATGTTGGAGTTAAGCAATGGGAAGACTTTGCTGATAAATTTGAGGCCA
ACGAGAAGTCAAAGTTGCCAAAAAT
>PDIL5_2a_exon3500_138
GAGTTAAGCAATGGGAAGACTTTGCTGATAAATTTGAGGCCAACGAGAAGTCAAAGTTGCCAAAAATGATTGTCT
GGAATGGAGATGTGGAGTACTTATC
>PDIL5_2a_exon5000_0
GGTTGTTGGCGTTGAAAGCCTTGATAATGAAGATCAGGGGTCTCAGATCTCACGTTTCCTCGAAGGATATAGAGA
AGGAAGAACAGAAAGAAAAACAGTT
>PDIL5_2a_exon5000_29
AAGATCAGGGGTCTCAGATCTCACGTTTCCTCGAAGGATATAGAGAAGGAAGAACAGAAAGAAAAACAGTTAAAG
GGCCATCATTTATGGACTTCATCCA
>PDIL5_2a_exon5000_58
CTCGAAGGATATAGAGAAGGAAGAACAGAAAGAAAAACAGTTAAAGGGCCATCATTTATGGACTTCATCCATTCA
CTAATCGGTATCAGAAGTGTCTACA
>PDIL5_2a_exon5000_87
AAGAAAAACAGTTAAAGGGCCATCATTTATGGACTTCATCCATTCACTAATCGGTATCAGAAGTGTCTACATAAT
TGTCTTTATTGTTGCAATAATGATG
>PDIL5_2a_exon5000_116
TGGACTTCATCCATTCACTAATCGGTATCAGAAGTGTCTACATAATTGTCTTTATTGTTGCAATAATGATGCTTA
TACAAAGCATTGGGAAAGAAGACGA
>Phosphatidylinositol_exon3280_0
AGGTTCGTGCATATGCTGTTAGTGTTCTTGAAAGGGCAGATGATGAGGAGCTTCAATGTTATTTGCTTCAACTAG
TTCAGGCACTTCGATTTGAACGCAC
>Phosphatidylinositol_exon3280_18
TTAGTGTTCTTGAAAGGGCAGATGATGAGGAGCTTCAATGTTATTTGCTTCAACTAGTTCAGGCACTTCGATTTG
AACGCACTGATAAATCTCGGCTTTC
>Phosphatidylinositol_exon3280_36
CAGATGATGAGGAGCTTCAATGTTATTTGCTTCAACTAGTTCAGGCACTTCGATTTGAACGCACTGATAAATCTC
GGCTTTCTCAATTCCTCGTGCAACG
>Phosphatidylinositol_exon3950_0
CTTTGCGTAATATTGAACTGGCTAGTTTTCTACGGTGGTTTGTTGCTGTGGAACTTCACGATCCTGCTTATGCAA
AACGATTTTATTCTACCTACGAGTT
>Phosphatidylinositol_exon3950_10
TATTGAACTGGCTAGTTTTCTACGGTGGTTTGTTGCTGTGGAACTTCACGATCCTGCTTATGCAAAACGATTTTA
TTCTACCTACGAGTTCCTAGAAGAA
>Phosphatidylinositol_exon3950_20
GCTAGTTTTCTACGGTGGTTTGTTGCTGTGGAACTTCACGATCCTGCTTATGCAAAACGATTTTATTCTACCTAC
GAGTTCCTAGAAGAAAACATGATAA
>Phosphatidylinositol_exon4350_0
CGTCAGACAGAACTCACAGCCCAGCTGTGTTCTATAATGAGAGATGTGAGGAATGTGCGTGGCAATACACAAAAG
AAGATTGAAAAGCTTAGACAGCTCC

>Phosphatidylinositol_exon4350_18
GCCCAGCTGTGTTCTATAATGAGAGATGTGAGGAATGTGCGTGGCAATACACAAAAGAAGATTGAAAAGCTTAGA
CAGCTCCTTTCCGGTCTTCTTAGTG
>Phosphatidylinositol_exon4350_36
ATGAGAGATGTGAGGAATGTGCGTGGCAATACACAAAAGAAGATTGAAAAGCTTAGACAGCTCCTTTCCGGTCTT
CTTAGTGAACTTACTTATTTTGATG
>Phosphatidylinositol_exon4590_0
AGCCAATTCGGTCACCCTTGGCTCCAAGTGTCCTTATCACTGGAATTGTACCTTCAGAGTCGTCAATATTCAAAA
GTGCATTACATCCTTTGCGACTGAC
>Phosphatidylinositol_exon4590_14
CCCTTGGCTCCAAGTGTCCTTATCACTGGAATTGTACCTTCAGAGTCGTCAATATTCAAAAGTGCATTACATCCT
TTGCGACTGACTTTTCGAACAGCCA
>Phosphatidylinositol_exon4590_27
GTGTCCTTATCACTGGAATTGTACCTTCAGAGTCGTCAATATTCAAAAGTGCATTACATCCTTTGCGACTGACTT
TTCGAACAGCCAATGGTGGACAAAG
>Phosphatidylinositol_exon4860_0
CAAATGGTGTCACTTATGGATAGATTACTGAAGTTGGAAAATCTTGATCTGCACTTAACTCCATATAAGGTACTG
GCAACTGGACAAGATGAGGGCATGC
>Phosphatidylinositol_exon4860_15
ATGGATAGATTACTGAAGTTGGAAAATCTTGATCTGCACTTAACTCCATATAAGGTACTGGCAACTGGACAAGAT
GAGGGCATGCTGGAATTCATACCAT
>Phosphatidylinositol_exon4860_29
GAAGTTGGAAAATCTTGATCTGCACTTAACTCCATATAAGGTACTGGCAACTGGACAAGATGAGGGCATGCTGGA
ATTCATACCATCCCGTTCTTTGGCA
>Phosphatidylinositol_exon5400_0
CAGATTCTTTCAGATCATCGTAGCATTATAAGCTATCTACAGAAGTTCCATCCTGATGAACATGGACCATTCGGG
ATTACAGCCACTTGTCTCGAAACCT
>Phosphatidylinositol_exon5400_20
TAGCATTATAAGCTATCTACAGAAGTTCCATCCTGATGAACATGGACCATTCGGGATTACAGCCACTTGTCTCGA
AACCTTTATAAAAAGTTGTGCTGGC
>Phosphatidylinositol_exon5400_40
AGAAGTTCCATCCTGATGAACATGGACCATTCGGGATTACAGCCACTTGTCTCGAAACCTTTATAAAAAGTTGTG
CTGGCTACTCTGTTATCACATATAT
>Phosphatidylinositol_exon5740_0
CTTCTGCTTAGAGATGATGGACGCCTCTTCCATGTTGATTTTGGTTTTATTCTTGGCCGGGATCCTAAACCATTT
CCGCCACCAATGAAACTTTGCAAAG
>Phosphatidylinositol_exon5740_15
GATGGACGCCTCTTCCATGTTGATTTTGGTTTTATTCTTGGCCGGGATCCTAAACCATTTCCGCCACCAATGAAA
CTTTGCAAAGAAATGGTTGAAGCTA
>Phosphatidylinositol_exon5740_29
CCATGTTGATTTTGGTTTTATTCTTGGCCGGGATCCTAAACCATTTCCGCCACCAATGAAACTTTGCAAAGAAAT
GGTTGAAGCTATGGGTGGAGCAGAG
>Phosphatidylinositol_exon6030_0
AGCCAATATTATACAAGGTTCAAATCCTATTGTTGTGAAGCATACAACATTCTCCGTAAATCCAGTAACCTTATT
TTAAATCTTTTCCATCTCATGGCTG
>Phosphatidylinositol_exon6030_24
TCCTATTGTTGTGAAGCATACAACATTCTCCGTAAATCCAGTAACCTTATTTTAAATCTTTTCCATCTCATGGCT
GGTTCCAACATTCGCTACATAGCAT
>Phosphatidylinositol_exon6030_48
ATTCTCCGTAAATCCAGTAACCTTATTTTAAATCTTTTCCATCTCATGGCTGGTTCCAACATTCGCTACATAGCA
TCTGATCCTGAAAAAGGCATCCTCA
>Phosphatidylinositol_exon6260_0
AGCTCCAAGAGAAGTTCCGGTTGGACTTAGATGATGAGGCCTGCATACATTTCTTCCAGGATCTTATCAATGAGA
GTGTTAGTGCATTGTTTCCTCAAAT
>Phosphatidylinositol_exon6260_17
CGGTTGGACTTAGATGATGAGGCCTGCATACATTTCTTCCAGGATCTTATCAATGAGAGTGTTAGTGCATTGTTT
CCTCAAATGGTTGAGACTATTCATC

>Phosphatidylinositol_exon6260_33
ATGAGGCCTGCATACATTTCTTCCAGGATCTTATCAATGAGAGTGTTAGTGCATTGTTTCCTCAAATGGTTGAGA
CTATTCATCGTTGGGCTCAATACTG
>Phospholipase_exon900_0
CTTGTTAGAGAGCCTACTCGGCCATTGCCTCGAGGCGGGGACTTAACGCTCGGTGAATTGCTTAAATATAAATCT
GAAGAAGGGGTTAGAGTTCTGTTGT
>Phospholipase_exon900_23
ATTGCCTCGAGGCGGGGACTTAACGCTCGGTGAATTGCTTAAATATAAATCTGAAGAAGGGGTTAGAGTTCTGTT
GTTGGTTTGGGATGATAAAACTTCA
>Phospholipase_exon900_46
CGCTCGGTGAATTGCTTAAATATAAATCTGAAGAAGGGGTTAGAGTTCTGTTGTTGGTTTGGGATGATAAAACTT
CACACGATAAATTCGGTATCCGCAC
>Phospholipase_exon1120_0
GGCCGGAGTTATGCAGACGCACGATGAAGAAACGTTGAAGTTTTTTAAGCATTCTTCTGTTACTTGTGTGCTGGC
TCCACGCTATGCTAGCAGTAAGCTT
>Phospholipase_exon1120_10
ATGCAGACGCACGATGAAGAAACGTTGAAGTTTTTTAAGCATTCTTCTGTTACTTGTGTGCTGGCTCCACGCTAT
GCTAGCAGTAAGCTTGGGTATTTCA
>Phospholipase_exon1120_20
ACGATGAAGAAACGTTGAAGTTTTTTAAGCATTCTTCTGTTACTTGTGTGCTGGCTCCACGCTATGCTAGCAGTA
AGCTTGGGTATTTCAAACAACAGGC
>Phospholipase_exon1740_0
AATAATCGAAAGATAACTGCATTTGTTGGAGGTATCGATCTCTGTGATGGCCGCTACGATACACCTGAACATCGA
CTACTTTGTGATCTTGACACTATTT
>Phospholipase_exon1740_16
CTGCATTTGTTGGAGGTATCGATCTCTGTGATGGCCGCTACGATACACCTGAACATCGACTACTTTGTGATCTTG
ACACTATTTTCAAGGATGATTTTCA
>Phospholipase_exon1740_32
TATCGATCTCTGTGATGGCCGCTACGATACACCTGAACATCGACTACTTTGTGATCTTGACACTATTTTCAAGGA
TGATTTTCATAATCCTACTTTTCCT
>Phospholipase_exon2770_0
TGGCATGATTTACATAGCAGAATTGAAGGGCCTGCTGCATATGATGTTCTTATCAACTTTGAGCAGCGGTGGAGG
AAATCAACCAGGTGGAAAGAGTTCA
>Phospholipase_exon2770_15
AGCAGAATTGAAGGGCCTGCTGCATATGATGTTCTTATCAACTTTGAGCAGCGGTGGAGGAAATCAACCAGGTGG
AAAGAGTTCAGCCTACTTTTTAAAG
>Phospholipase_exon2770_30
CCTGCTGCATATGATGTTCTTATCAACTTTGAGCAGCGGTGGAGGAAATCAACCAGGTGGAAAGAGTTCAGCCTA
CTTTTTAAAGGAAAATCTCACTGGA
>Phospholipase_exon3400_0
CAAAAAATCTGGTGATAGATAAAAGCATTCAAACTGCATATATACAGGCTATCAGGTCTGCCCAGCATTACATAT
ATATTGAAAATCAGTATTTCCTTGG
>Phospholipase_exon3400_16
AGATAAAAGCATTCAAACTGCATATATACAGGCTATCAGGTCTGCCCAGCATTACATATATATTGAAAATCAGTA
TTTCCTTGGATCTTCATATGCATGG
>Phospholipase_exon3400_31
AACTGCATATATACAGGCTATCAGGTCTGCCCAGCATTACATATATATTGAAAATCAGTATTTCCTTGGATCTTC
ATATGCATGGCCATCGTATAAAAAT
>Phospholipase_exon3750_0
GCAGGGGCTGATAATCTGATCCCTATGGAACTGGCACTGAAGATTGCTTCTAAAATCAGAGCAAGGGAGAGATTC
GCGGTATATGTCATCATTCCTTTGT
>Phospholipase_exon3750_9
GATAATCTGATCCCTATGGAACTGGCACTGAAGATTGCTTCTAAAATCAGAGCAAGGGAGAGATTCGCGGTATAT
GTCATCATTCCTTTGTGGCCCGAGG
>Phospholipase_exon3750_25
TGGAACTGGCACTGAAGATTGCTTCTAAAATCAGAGCAAGGGAGAGATTCGCGGTATATGTCATCATTCCTTTGT
GGCCCGAGGGTGATCCTAAAACTGC

```
>Phospholipase_exon4330_0
CAGAGTCAAACAATGCAAATGATGTATAATGTCGTTGCACAAGAACTCAAATCCATGCAAATTAAGGATGCACAT
CCTCGAGATTACCTCAATTTCTATT
>Phospholipase_exon4330_8
AACAATGCAAATGATGTATAATGTCGTTGCACAAGAACTCAAATCCATGCAAATTAAGGATGCACATCCTCGAGA
TTACCTCAATTTCTATTGTCTTGGT
>Phospholipase_exon4330_23
GTATAATGTCGTTGCACAAGAACTCAAATCCATGCAAATTAAGGATGCACATCCTCGAGATTACCTCAATTTCTA
TTGTCTTGGTAATCGGGAAGAGGTC
>Phospholipase_exon4800_0
ATGATAGTAGATGATGAGTATGTAATAGTGGGGTCAGCTAATATTAATCAAAGATCCATGGCTGGTACAAAAGAC
ACAGAGATAGCTATGGGTGCCTATC
>Phospholipase_exon4800_10
ATGATGAGTATGTAATAGTGGGGTCAGCTAATATTAATCAAAGATCCATGGCTGGTACAAAAGACACAGAGATAG
CTATGGGTGCCTATCAACCACATTA
>Phospholipase_exon4800_20
TGTAATAGTGGGGTCAGCTAATATTAATCAAAGATCCATGGCTGGTACAAAAGACACAGAGATAGCTATGGGTGC
CTATCAACCACATTATACATGGGCT
>Plasma_ATPase_exon100_0
ATTGCTCTTGCCAATGGAGGAGGGAAACCACCTGACTGGCAAGATTTTGTTGGGATTATTACTCTTCTTCTTATT
AATTCAACAATAAGTTTCATAGAAG
>Plasma_ATPase_exon100_20
AGGGAAACCACCTGACTGGCAAGATTTTGTTGGGATTATTACTCTTCTTCTTATTAATTCAACAATAAGTTTCAT
AGAAGAGAACAATGCTGGCAATGCA
>Plasma_ATPase_exon100_39
CAAGATTTTGTTGGGATTATTACTCTTCTTCTTATTAATTCAACAATAAGTTTCATAGAAGAGAACAATGCTGGC
AATGCAGCTGCTGCTTTGATGGCTC
>Plasma_ATPase_exon320_0
AGGTGTTTCGAGACGGCAAGTGGATTGAAGAAGATGCCTCTGTTCTTGTCCCCGGTGATATCATCAGCATTAAAC
TTGGTGACATTATTCCGGCAGATGC
>Plasma_ATPase_exon320_11
GACGGCAAGTGGATTGAAGAAGATGCCTCTGTTCTTGTCCCCGGTGATATCATCAGCATTAAACTTGGTGACATT
ATTCCGGCAGATGCTCGACTCCTCG
>Plasma_ATPase_exon320_21
GGATTGAAGAAGATGCCTCTGTTCTTGTCCCCGGTGATATCATCAGCATTAAACTTGGTGACATTATTCCGGCAG
ATGCTCGACTCCTCGATGGTGATCC
>Plasma_ATPase_exon570_0
AGGCCCCGGTGACAGTATTTACTCTGGTTCTACATGCAAACAAGGAGAGATTGAAGCTGTCGTGATCGCCACCGG
TGTTCATACGTTCTTTGGCAAAGCT
>Plasma_ATPase_exon570_22
TCTGGTTCTACATGCAAACAAGGAGAGATTGAAGCTGTCGTGATCGCCACCGGTGTTCATACGTTCTTTGGCAAA
GCTGCTCACCTTGTTGATTCCACAA
>Plasma_ATPase_exon570_43
GGAGAGATTGAAGCTGTCGTGATCGCCACCGGTGTTCATACGTTCTTTGGCAAAGCTGCTCACCTTGTTGATTCC
ACAAATCAACAAGGTCACTTTCAAA
>Plasma_ATPase_exon850_0
TCGGAATGATAACGGAAATCATCGTCATGTACCCAATTCAAGACCGTGAATATCGTCCCGGAATTGACAATTTGC
TGGTACTACTCATTGGAGGAATTCC
>Plasma_ATPase_exon850_20
ATCGTCATGTACCCAATTCAAGACCGTGAATATCGTCCCGGAATTGACAATTTGCTGGTACTACTCATTGGAGGA
ATTCCTATTGCCATGCCCACGGTTC
>Plasma_ATPase_exon850_39
AAGACCGTGAATATCGTCCCGGAATTGACAATTTGCTGGTACTACTCATTGGAGGAATTCCTATTGCCATGCCCA
CGGTTCTATCTGTTACAATGGCAAT
>Plasma_ATPase_exon1100_0
CAGGGGGCTATCACGAAAAGAATGACAGCAATAGAAGAAATGGCAGGCATGGATGTCCTTTGCAGTGATAAAACC
GGAACTTTGACATTGAATAAGTTAT
```

>Plasma_ATPase_exon1100_11
CACGAAAAGAATGACAGCAATAGAAGAAATGGCAGGCATGGATGTCCTTTGCAGTGATAAAACCGGAACTTTGAC
ATTGAATAAGTTATCAGTTGACAAA
>Plasma_ATPase_exon1100_21
ATGACAGCAATAGAAGAAATGGCAGGCATGGATGTCCTTTGCAGTGATAAAACCGGAACTTTGACATTGAATAAG
TTATCAGTTGACAAAAATCTTATTG
>Plasma_ATPase_exon1300_0
AGATCTTTGCAAAAGGAGTGGATCCCGATACCGTTGTTCTGATGGCAGCCCGTGCATCTCGGCTTGAAAACCAGG
ATGCCATAGATGCTGCTATAGTAGG
>Plasma_ATPase_exon1300_10
AAAAGGAGTGGATCCCGATACCGTTGTTCTGATGGCAGCCCGTGCATCTCGGCTTGAAAACCAGGATGCCATAGA
TGCTGCTATAGTAGGGATGTTGGCT
>Plasma_ATPase_exon1300_20
GATCCCGATACCGTTGTTCTGATGGCAGCCCGTGCATCTCGGCTTGAAAACCAGGATGCCATAGATGCTGCTATA
GTAGGGATGTTGGCTGATCCAAAGG
>Plasma_ATPase_exon1730_0
CAGATTTTGAATCTTGCACACAATAAATCGGAGTTGGAACGTCGTGTTCATGCAGTAATCGATAAATTTGCGGAG
CGGGGATTGCGATCACTTGCAGTAG
>Plasma_ATPase_exon1730_5
TTTGAATCTTGCACACAATAAATCGGAGTTGGAACGTCGTGTTCATGCAGTAATCGATAAATTTGCGGAGCGGGG
ATTGCGATCACTTGCAGTAGCATAT
>Plasma_ATPase_exon1730_9
AATCTTGCACACAATAAATCGGAGTTGGAACGTCGTGTTCATGCAGTAATCGATAAATTTGCGGAGCGGGGATTG
CGATCACTTGCAGTAGCATATCAGG
>Plasma_ATPase_exon1930_0
AAGTTCCTGATGGACAAAAGGAGAGCTCAGGGGGTCCATGGCAATTTGTTGGCCTGATGCCTTTGTTCGATCCAC
CAAGACATGACAGTGCAGATACTAT
>Plasma_ATPase_exon1930_21
AGAGCTCAGGGGGTCCATGGCAATTTGTTGGCCTGATGCCTTTGTTCGATCCACCAAGACATGACAGTGCAGATA
CTATACGAAGGGCATTGAATCTTGG
>Plasma_ATPase_exon1930_42
AATTTGTTGGCCTGATGCCTTTGTTCGATCCACCAAGACATGACAGTGCAGATACTATACGAAGGGCATTGAATC
TTGGAGTGAATGTGAAAATGATCAC
>Plasma_ATPase_exon2200_0
AGGTGATCAACTGGCGATAGCAAAGGAAACTGGACGGCGTCTTGGAATGGGAACCAACATGTACCCTTCATCATC
TTTGTTAGGACAAAACAAAGAAGAA
>Plasma_ATPase_exon2200_9
ACTGGCGATAGCAAAGGAAACTGGACGGCGTCTTGGAATGGGAACCAACATGTACCCTTCATCATCTTTGTTAGG
ACAAAACAAAGAAGAATCGATTGCT
>Plasma_ATPase_exon2200_26
AAACTGGACGGCGTCTTGGAATGGGAACCAACATGTACCCTTCATCATCTTTGTTAGGACAAAACAAAGAAGAAT
CGATTGCTGCTTTGCCGATTGATGA
>Plasma_ATPase_exon2600_0
GCTGTGGCCGATGCAACCGATGCAGCCCGTAGTGCTTCGGACATTGTCTTGACTGAACCTGGTCTTAGTGTCATT
ATCAGTGCCGTACTAACCAGTCGAG
>Plasma_ATPase_exon2600_15
ACCGATGCAGCCCGTAGTGCTTCGGACATTGTCTTGACTGAACCTGGTCTTAGTGTCATTATCAGTGCCGTACTA
ACCAGTCGAGCTATATTCCAGAGGA
>Plasma_ATPase_exon2600_29
TAGTGCTTCGGACATTGTCTTGACTGAACCTGGTCTTAGTGTCATTATCAGTGCCGTACTAACCAGTCGAGCTAT
ATTCCAGAGGATGAAAAATTACACA
>Plasma_ATPase_exon3200_0
GTGAAACCATCTCCTCTTCCAGACAGTTGGAAGTTAGCAGAGATCTTTACAACTGGAGTCGTTCTTGGTGGTTAC
TTAGCCATGATGACTGTCATTTTCT
>Plasma_ATPase_exon3200_16
TTCCAGACAGTTGGAAGTTAGCAGAGATCTTTACAACTGGAGTCGTTCTTGGTGGTTACTTAGCCATGATGACTG
TCATTTTCTTTTGGGCAGCATACAA

>Plasma_ATPase_exon3200_32
GTTAGCAGAGATCTTTACAACTGGAGTCGTTCTTGGTGGTTACTTAGCCATGATGACTGTCATTTTCTTTTGGGC
AGCATACAAGACAGACTTCTTCCCT
>Plasma_ATPase_exon3400_0
TTTGGGGTTACGAGCCTTCAGAAAAACGACCGCCAAGACATAAGAATGCTTGCTTCGGCGGTATACCTCCAAGTG
AGCATTATCAGTCAGGCTCTCATAT
>Plasma_ATPase_exon3400_15
CTTCAGAAAAACGACCGCCAAGACATAAGAATGCTTGCTTCGGCGGTATACCTCCAAGTGAGCATTATCAGTCAG
GCTCTCATATTTGTAACAAGAGCAA
>Plasma_ATPase_exon3400_29
CCGCCAAGACATAAGAATGCTTGCTTCGGCGGTATACCTCCAAGTGAGCATTATCAGTCAGGCTCTCATATTTGT
AACAAGAGCAAGGAGTTGGTCTTTC
>Plasma_ATPase_exon3800_0
GGATGGGGTTGGGCAGGCGTGATATGGCTTTACAACCTTATCTTTTACTTTCCACTCGACTTCATCAAGTTCTTC
ATCCGATACGCCCTCAGCGGAAAGG
>Plasma_ATPase_exon3800_9
TGGGCAGGCGTGATATGGCTTTACAACCTTATCTTTTACTTTCCACTCGACTTCATCAAGTTCTTCATCCGATAC
GCCCTCAGCGGAAAGGCTTGGGATC
>Plasma_ATPase_exon3800_25
GGCTTTACAACCTTATCTTTTACTTTCCACTCGACTTCATCAAGTTCTTCATCCGATACGCCCTCAGCGGAAAGG
CTTGGGATCTTGTTATCGAACAAAG
>Polysub2_exon1100_0
GAAGTTTCAAAAGATTTAGCTATGGCATCACTCATCCACTCGAGAAAACTCTTGGCATAATCAATAGTGCTTGAT
GAAACATATGTCAGAAAGTAGATGG
>Polysub2_exon1100_14
TTTAGCTATGGCATCACTCATCCACTCGAGAAAACTCTTGGCATAATCAATAGTGCTTGATGAAACATATGTCAG
AAAGTAGATGGGGAAGTTCAAAGAC
>Polysub2_exon1100_27
TCACTCATCCACTCGAGAAAACTCTTGGCATAATCAATAGTGCTTGATGAAACATATGTCAGAAAGTAGATGGGG
AAGTTCAAAGACTTCAATGACCAGT
>Polysub2_exon1300_0
CTCTTCTAGAACCAAGAGAAGTTCCAAGACACGACCAGCAGTATCGACAGGGAGTAACACATTTCCACCACCTTC
TAGAGTCCTTGAGATGATttttttt
>Polysub2_exon1560_0
CCCTGTCCCTCTCCCTTTGCGGTTTAGGAGGTTGATTGTTCAAGGCATTGTAGGCATCAGTTATAAGAACAGCAG
GTCGAACAAAAGACTCCAGAACAGT
>Polysub2_exon1560_6
CCCTCTCCCTTTGCGGTTTAGGAGGTTGATTGTTCAAGGCATTGTAGGCATCAGTTATAAGAACAGCAGGTCGAA
CAAAAGACTCCAGAACAGTTCCATT
>Polysub2_exon1560_12
CCCTTTGCGGTTTAGGAGGTTGATTGTTCAAGGCATTGTAGGCATCAGTTATAAGAACAGCAGGTCGAACAAAAG
ACTCCAGAACAGTTCCATTTAAATG
>Polysub2_exon1770_0
CTTCTCTTTGCGTCGGTTAAAGTCTACAGCATATATGACATCTTCTCCATCCTTTGTTATCTTCCAAACAGTACC
ACCCAATAGATGGCCAGCAACATGA
>Polysub2_exon1770_14
GGTTAAAGTCTACAGCATATATGACATCTTCTCCATCCTTTGTTATCTTCCAAACAGTACCACCCAATAGATGGC
CAGCAACATGAGGAGCAATCACAAT
>Polysub2_exon1770_27
AGCATATATGACATCTTCTCCATCCTTTGTTATCTTCCAAACAGTACCACCCAATAGATGGCCAGCAACATGAGG
AGCAATCACAATACCCTCACCTTTT
>Polysub2_exon2120_0
CCAGATAAATGATAATTCTGCGAGTAGGTTAACCGAGTTACGCTCTGGAAAGCAGAATCAATGTCATCCAAGGTG
AACAAATCGAACTCCGATACTTGCt
>Polysub2_exon2450_0
CTTACGAGAGAGATACTGGTCGTACATAGTGAGAAGGCCTAATCGAAAAACCGGTTCAGTGGAATAAACCGGAGC
GGAGAGTCCAAAATGTTTCATAGCA

>Polysub2_exon2450_15
CTGGTCGTACATAGTGAGAAGGCCTAATCGAAAAACCGGTTCAGTGGAATAAACCGGAGCGGAGAGTCCAAAATG
TTTCATAGCATACGGAAGAGCGCCG
>Polysub2_exon2450_29
TGAGAAGGCCTAATCGAAAAACCGGTTCAGTGGAATAAACCGGAGCGGAGAGTCCAAAATGTTTCATAGCATACG
GAAGAGCGCCGAGGTGAAGCGTGTC
>Polysub2_exon2700_0
TGGAGAGGGGTTGAAGAAGGGAGGGGTCAAAGACGTCATTCCAGCCACAGTCGATTAAGAAGTTGAAGCCGTCGA
TTGAGATTAAGTAGGAGAGAGGATT
>Polysub2_exon2700_24
GGTCAAAGACGTCATTCCAGCCACAGTCGATTAAGAAGTTGAAGCCGTCGATTGAGATTAAGTAGGAGAGAGGAT
TCTCGTTGTATACTCCGCAGAGTGG
>Polysub2_exon2700_47
CAGTCGATTAAGAAGTTGAAGCCGTCGATTGAGATTAAGTAGGAGAGAGGATTCTCGTTGTATACTCCGCAGAGT
GGGGTTACCTGAACCGACGTCCCCA
>RNF14_exon60_0
CTATCCCTCACTGTGCTGCTTGCAACCCTTGGGTCCGTAGTGTTGAGCGCTGCGCCTTACAATTTTTTTGCACAA
GTAACAGTAGTGCATTTGGCATGCC
>RNF14_exon60_14
GCTGCTTGCAACCCTTGGGTCCGTAGTGTTGAGCGCTGCGCCTTACAATTTTTTTGCACAAGTAACAGTAGTGCA
TTTGGCATGCCCAGCAGAATATGTG
>RNF14_exon60_27
CTTGGGTCCGTAGTGTTGAGCGCTGCGCCTTACAATTTTTTTGCACAAGTAACAGTAGTGCATTTGGCATGCCCA
GCAGAATATGTGATTGTTGTTTCCT
>RNF14_exon540_0
ACCTTTGCATTGAACTGACGACAATTAGGGCATGGCAGCCCACGGTCAGCAAATAGTTGAGCGTGAACTTGACCT
AACACCTGGCGTGCATTCATCCTTT
>RNF14_exon540_25
TAGGGCATGGCAGCCCACGGTCAGCAAATAGTTGAGCGTGAACTTGACCTAACACCTGGCGTGCATTCATCCTTT
CCTCCCAGTCCTGTATCATTTCTTG
>RNF14_exon540_49
CAAATAGTTGAGCGTGAACTTGACCTAACACCTGGCGTGCATTCATCCTTTCCTCCCAGTCCTGTATCATTTCTT
GTGGAAACAATTCACATGCCCCATC
>RNF14_exon830_0
CAGAAATACTGCCCGCAGTTCTCACATACCATCTTGTTACATCCCTCAGTTCTTGAGATTGCCATCTTACAAGAT
GGACATTGCTTTGCGTCACGCATTA
>RNF14_exon830_21
TCACATACCATCTTGTTACATCCCTCAGTTCTTGAGATTGCCATCTTACAAGATGGACATTGCTTTGCGTCACGC
ATTATTTCCTTCATGCTAAGAAGTT
>RNF14_exon830_41
TCCCTCAGTTCTTGAGATTGCCATCTTACAAGATGGACATTGCTTTGCGTCACGCATTATTTCCTTCATGCTAAG
AAGTTCGTTGATCATCTCACGCTCC
>RNF14_exon1420_0
GCTATGCCGACATGACGTCGCTCCCTGCAAAGTGTACAAAAGCTAAAGAAGCACTTGGAGCATTGTGCATGTTGC
TCTTCATCCTCTATACAAGGTGTTT
>RNF14_exon1420_9
ACATGACGTCGCTCCCTGCAAAGTGTACAAAAGCTAAAGAAGCACTTGGAGCATTGTGCATGTTGCTCTTCATCC
TCTATACAAGGTGTTTCACATCTTG
>RNF14_exon1420_25
TGCAAAGTGTACAAAAGCTAAAGAAGCACTTGGAGCATTGTGCATGTTGCTCTTCATCCTCTATACAAGGTGTTT
CACATCTTGGGCAATATGCAACATC
>RNF14_exon2220_0
TACCTGCATGCTCGCTAAGACAGATTGGACATTCATGCAAGTTTTTGAGGAAGTTTTCATGGAATTTCTTATCAT
TGTAACTCCTAATAAATGGAACATC
>RNF14_exon2220_14
CTAAGACAGATTGGACATTCATGCAAGTTTTTGAGGAAGTTTTCATGGAATTTCTTATCATTGTAACTCCTAATA
AATGGAACATCAACATCTGGAGAGA

>RNF14_exon2220_27
GACATTCATGCAAGTTTTTGAGGAAGTTTTCATGGAATTTCTTATCATTGTAACTCCTAATAAATGGAACATCAA
CATCTGGAGAGACACTTCCCGAAAT
>RNF14_exon2500_0
ATTCTAGCAGGATGCAACCACTGAGCAGAGAGAGTAAAATAGGGAGGAAGATGGCTGGGGTATGATCTAGGCAGT
AAACAAGTCAAGACGAGCGGTGGAA
>RNF14_exon2500_20
CTGAGCAGAGAGAGTAAAATAGGGAGGAAGATGGCTGGGGTATGATCTAGGCAGTAAACAAGTCAAGACGAGCGG
TGGAAGATGTTGGACTTTGAAAGAG
>RNF14_exon2500_39
TAGGGAGGAAGATGGCTGGGGTATGATCTAGGCAGTAAACAAGTCAAGACGAGCGGTGGAAGATGTTGGACTTTG
AAAGAGTATGAAAAGTCATCAGAAG
>RNF14_exon3270_0
CTCGTCCTCCTGCAACTGCTCATTGTTTCTCAGTTGTTCCTCAGGCAGCTCGGGTTCTTCCCCCCTTAACCGCAG
CTCCTCCAACCTACTCACTACATAA
>RNF14_exon3270_16
TGCTCATTGTTTCTCAGTTGTTCCTCAGGCAGCTCGGGTTCTTCCCCCCTTAACCGCAGCTCCTCCAACCTACTC
ACTACATAATCCGCACCGCTATGTG
>RNF14_exon3270_32
GTTGTTCCTCAGGCAGCTCGGGTTCTTCCCCCCTTAACCGCAGCTCCTCCAACCTACTCACTACATAATCCGCAC
CGCTATGTGATTCATCTCCTTCAAT
>rpb2_exon_4_2k_0
GAAAGCCCAGACGACGGTGGCTGGCATGATCTTGTAGCGAAGGGTTTTATTGAATATATTGACACAGAAGAAGAG
GAGACAACAATGATTTCCATGACCA
>rpb2_exon_4_2k_5
CCCAGACGACGGTGGCTGGCATGATCTTGTAGCGAAGGGTTTTATTGAATATATTGACACAGAAGAAGAGGAGAC
AACAATGATTTCCATGACCATCAAT
>rpb2_exon_4_4k_0
GATCTTGTCCAAGCAAGAATCAATCCAGAGGAAGCATATTCTGAAACTTATACCCATTGTGAGATCCATCCTTCG
TTGATTTTGGGTGTTTGTGCATCAA
>rpb2_exon_4_4k_9
CAAGCAAGAATCAATCCAGAGGAAGCATATTCTGAAACTTATACCCATTGTGAGATCCATCCTTCGTTGATTTTG
GGTGTTTGTGCATCAATTATACCGT
>rpb2_exon_4_4k_26
AGAGGAAGCATATTCTGAAACTTATACCCATTGTGAGATCCATCCTTCGTTGATTTTGGGTGTTTGTGCATCAAT
TATACCGTTCCCTGATCATAATCAG
>rpb2_exon_4_5k_0
AGTCCCCACGTAATACCTACCAATCTGCTATGGGTAAGCAAGCAATGGGTATATATGTCACCAACTACCAATTTC
GAATGGTtttttttttttttttttt
>rpb2_exon_4k_0
GTTGATGTCAACACTGAAGTTGGTGTTGTTCGAGATATTCGTTTAAAAGAACTTCGAATATATACTGACTATGGT
CGTTGCAGTCGGCCGTTGTTCATTG
>rpb2_exon_4k_28
TTCGAGATATTCGTTTAAAAGAACTTCGAATATATACTGACTATGGTCGTTGCAGTCGGCCGTTGTTCATTGTGG
AGAAGCAAAGGCTTCTGATAAAAAA
>rpb2_exon_4k_56
AATATATACTGACTATGGTCGTTGCAGTCGGCCGTTGTTCATTGTGGAGAAGCAAAGGCTTCTGATAAAAAAGAA
AGACATCCATGCACTGCAACAAAGG
>RRP5_exon2100_0
TGGAGAAGGATGTACCGAGAACTGCCGATGAATTTGAGAAACTTGTTAGGAGCTCTCCTAATAGCAGTTTTGTGT
GGATTCAATACATGGCTTTCATGCT
>RRP5_exon2100_20
ACTGCCGATGAATTTGAGAAACTTGTTAGGAGCTCTCCTAATAGCAGTTTTGTGTGGATTCAATACATGGCTTTC
ATGCTTAATTCAGCTGATATCGAGA
>RRP5_exon2100_39
AACTTGTTAGGAGCTCTCCTAATAGCAGTTTTGTGTGGATTCAATACATGGCTTTCATGCTTAATTCAGCTGATA
TCGAGAAAGCCCGTGCTATTGCTGA

>RRP5_exon2900_0
GGCGTTGAGAACCATAAACATCCGAGAAGAAACAGAGAAGCTAAATATCTGGGTGGCTTACTTTAATTTGGAGAA
TCAGTATGGGAATCCTCCAGAGttt
>RRP5_exon3100_0
GTATTTCAAAGGGCATTGCAGTATTGTGATCCCAAAAAGGTACATTTTGCACTTCTGGGCATGTACGACAGGACG
GAGCAACATAACTTGGCCGATGTGC
>RRP5_exon3100_17
GCAGTATTGTGATCCCAAAAAGGTACATTTTGCACTTCTGGGCATGTACGACAGGACGGAGCAACATAACTTGGC
CGATGTGCTACTTGACAAAATGAGC
>RRP5_exon3100_34
AAAAGGTACATTTTGCACTTCTGGGCATGTACGACAGGACGGAGCAACATAACTTGGCCGATGTGCTACTTGACA
AAATGAGCAAGAAGTTTAAGCACTC
>RRP5_exon3400_0
CAACAGCAGGATGGTGTGCAACCTGTTGTAAACCGTGCTTTACTATGCCTTCCACGCCATAAACACATAAAGTTC
ATTTCACAGGCAGCTATACTTGAGT
>RRP5_exon3400_22
CTGTTGTAAACCGTGCTTTACTATGCCTTCCACGCCATAAACACATAAAGTTCATTTCACAGGCAGCTATACTTG
AGTTCAAATCTGGGGTTCCTGATAG
>RRP5_exon3400_43
TATGCCTTCCACGCCATAAACACATAAAGTTCATTTCACAGGCAGCTATACTTGAGTTCAAATCTGGGGTTCCTG
ATAGAGGCAGATCTATGTTTGAGGG
>SART-1_exon2340_0
AAGATTTCATCAGATTATTACACCCAAGAGGAAATGCTTCGATTTAAAAAGCCCAAAAAGAAGAAAGCTTTGCGG
AAGAAAGAAAAGTTGGATATAGATG
>SART-1_exon2340_8
ATCAGATTATTACACCCAAGAGGAAATGCTTCGATTTAAAAAGCCCAAAAAGAAGAAAGCTTTGCGGAAGAAAGA
AAAGTTGGATATAGATGCCCTTGAA
>SART-1_exon2340_23
CCAAGAGGAAATGCTTCGATTTAAAAAGCCCAAAAAGAAGAAAGCTTTGCGGAAGAAAGAAAAGTTGGATATAGA
TGCCCTTGAAGCAGAAGCCATCTCT
>SART-1_exon2650_0
AACCTGAGGAAGATGAAAATCAAGTTTTTGCTGATGATGAAGAGGATCTTTATAAGTCGCTTGAAAAAGCAAGGA
GGTTAGCTCTTAAAAAGCAAGAAGA
>SART-1_exon2650_14
GAAAATCAAGTTTTTGCTGATGATGAAGAGGATCTTTATAAGTCGCTTGAAAAAGCAAGGAGGTTAGCTCTTAAA
AAGCAAGAAGAAAAAATCAGGTCCGC
>SART-1_exon2650_28
TGCTGATGATGAAGAGGATCTTTATAAGTCGCTTGAAAAAGCAAGGAGGTTAGCTCTTAAAAAGCAAGAAGAAAA
ATCAGGTCCGCAAGCTATTGCGCTT
>SART-1_exon3450_0
GTTGGCAAAGGATTATCAGGTGCACTGAAGCTGCTTAAAGATCGAGGAACACTTAAAGAAACTATCGAATGGGGT
GGTCGGAACATGGACAAGAAAAAGA
>SART-1_exon3450_9
GGATTATCAGGTGCACTGAAGCTGCTTAAAGATCGAGGAACACTTAAAGAAACTATCGAATGGGGTGGTCGGAAC
ATGGACAAGAAAAAGAGCAAACTTG
>SART-1_exon3450_25
TGAAGCTGCTTAAAGATCGAGGAACACTTAAAGAAACTATCGAATGGGGTGGTCGGAACATGGACAAGAAAAAGA
GCAAACTTGTTGGCATTGTAGACGA
>SART-1_exon3900_0
ATGAAGCAATATCAGGAAGAATTGAAGCTGAAGCAAATGAAAAAATTCAGATACACCTTCACTTTCAGTGGAGAGG
ATGAGGGAAGCTCAAGCTCAGCTGA
>SART-1_exon3900_20
ATTGAAGCTGAAGCAAATGAAAAAATTCAGATACACCTTCACTTTCAGTGGAGAGGATGAGGGAAGCTCAAGCTCA
GCTGAAAACACCCTACCTTGTCCTT
>SART-1_exon3900_39
AAAAATTCAGATACACCTTCACTTTCAGTGGAGAGGATGAGGGAAGCTCAAGCTCAGCTGAAAACACCCTACCTT
GTCCTTAGTGGTCATGTCAAACCAG

>SBT3-5_exon350_0
AGGTTGAGGTTCAACTCCTTCTGCCTATTTTTGGTGCAGTCAACTTTGGTTTTGGTTAAACCACTCACGGACTTG
CTGCTGAAGCCAGTGCCGCAAAGGA
>SBT3-5_exon350_8
GTTCAACTCCTTCTGCCTATTTTTGGTGCAGTCAACTTTGGTTTTGGTTAAACCACTCACGGACTTGCTGCTGAA
GCCAGTGCCGCAAAGGAACTTGACA
>SBT3-5_exon350_23
CCTATTTTTGGTGCAGTCAACTTTGGTTTTGGTTAAACCACTCACGGACTTGCTGCTGAAGCCAGTGCCGCAAAG
GAACTTGACATAATCGTTTGTTTCA
>SBT3-5_exon750_0
GATTTGATTAGAGCTGTTATGCCTGCAACATGGGGGCAAGACATTGAAGTTCCTGACATTAGTGCGTATCCACTG
GTTTTTTCTGCGCCAAATGGTATAT
>SBT3-5_exon750_20
GCCTGCAACATGGGGGCAAGACATTGAAGTTCCTGACATTAGTGCGTATCCACTGGTTTTTTCTGCGCCAAATGG
TATATATGCAGCTACGATGTCCACT
>SBT3-5_exon750_40
ACATTGAAGTTCCTGACATTAGTGCGTATCCACTGGTTTTTTCTGCGCCAAATGGTATATATGCAGCTACGATGT
CCACTCCCGGTGCAGCTATGTCGGG
>SBT3-5_exon990_0
AATTTACCTTCAGCACAGCTGGGGAAATTGAACTAGGTCCTCTGGCTGAGAAATCTGCAACTCTAGGAGATAGCC
ATTTCCCAACGACTGTCTTTGGAAT
>SBT3-5_exon990_10
CAGCACAGCTGGGGAAATTGAACTAGGTCCTCTGGCTGAGAAATCTGCAACTCTAGGAGATAGCCATTTCCCAAC
GACTGTCTTTGGAATACTCAGCTTG
>SBT3-5_exon990_20
GGGGAAATTGAACTAGGTCCTCTGGCTGAGAAATCTGCAACTCTAGGAGATAGCCATTTCCCAACGACTGTCTTT
GGAATACTCAGCTTGGCAATTGGAG
>SBT3-5_exon1290_0
TGCGCAAAAATAAGCCCCACCCCTCCTGCTTCCGCTACAGAAATCGCAGCACTAAATATACTCTGTGTTCTTGAT
TGTGAAAAACAAAGTATGATCTTCC
>SBT3-5_exon1290_19
CCCCTCCTGCTTCCGCTACAGAAATCGCAGCACTAAATATACTCTGTGTTCTTGATTGTGAAAAACAAAGTATGA
TCTTCCCTGCTGCTAATGTTGCATT
>SBT3-5_exon1290_38
AGAAATCGCAGCACTAAATATACTCTGTGTTCTTGATTGTGAAAAACAAAGTATGATCTTCCCTGCTGCTAATGT
TGCATTGAGACTTCCTCGCTTGCAC
>SBT3-5_exon1700_0
AGGGTGCGATTATTTCCTAGTGTAATGGCTGTTTGGAAGGCCCTGTCTATGGTGGTAGCGGCAACGGTAATAATC
CAGGGAGCAGTGTTTTCAACTGTCA
>SBT3-5_exon1700_9
TTATTTCCTAGTGTAATGGCTGTTTGGAAGGCCCTGTCTATGGTGGTAGCGGCAACGGTAATAATCCAGGGAGCA
GTGTTTTCAACTGTCATTGCAGTAG
>SBT3-5_exon1700_25
TGGCTGTTTGGAAGGCCCTGTCTATGGTGGTAGCGGCAACGGTAATAATCCAGGGAGCAGTGTTTTCAACTGTCA
TTGCAGTAGGACCTTCATTCCCTGC
>SBT3-5_exon2240_0
ATAAGTTTCTTGTTACAGTTTGAGGAGTTGAAAAGCTCTCCCTCTTGGCATATACCTTTCCAACGAGATGGAATT
GGACTCATGCCCTTATCATTGAAAC
>SBT3-5_exon2240_8
CTTGTTACAGTTTGAGGAGTTGAAAAGCTCTCCCTCTTGGCATATACCTTTCCAACGAGATGGAATTGGACTCAT
GCCCTTATCATTGAAACTCTCAGAT
>SBT3-5_exon2240_23
GGAGTTGAAAAGCTCTCCCTCTTGGCATATACCTTTCCAACGAGATGGAATTGGACTCATGCCCTTATCATTGAA
ACTCTCAGATTCTGGCCACACTCCT
>SD1-1_Exon300_0
CTATCCTCGGGTCGTTTTTGAACACATAAAAGACCCACATGAATGGATCTTAGAACTTCGGATACCATGAAACAA
TCTCCCAAGTTACGGTCAACGAACT

>SD1-1_Exon300_17
TTGAACACATAAAAGACCCACATGAATGGATCTTAGAACTTCGGATACCATGAAACAATCTCCCAAGTTACGGTC
AACGAACTGCAATGGCTTCAGTTCC
>SD1-1_Exon300_33
CCCACATGAATGGATCTTAGAACTTCGGATACCATGAAACAATCTCCCAAGTTACGGTCAACGAACTGCAATGGC
TTCAGTTCCATCCACAGTTTCCATG
>SD1-1_Exon500_0
TCTGGGTGGAAGAATCCTCTGTTCTTCTTCCCACTTATGATCTCGAGTACTAACACACCGAAGCTAAACACATCG
GATTTTATCGAAAATAGTCCATCTA
>SD1-1_Exon500_9
AAGAATCCTCTGTTCTTCTTCCCACTTATGATCTCGAGTACTAACACACCGAAGCTAAACACATCGGATTTTATC
GAAAATAGTCCATCTACAGCATACT
>SD1-1_Exon500_26
CTTCCCACTTATGATCTCGAGTACTAACACACCGAAGCTAAACACATCGGATTTTATCGAAAATAGTCCATCTAC
AGCATACTCAGGAGGCATATAACCA
>SD1-1_Exon800_0
TCACCCCAAATAGTTCTTGCCAAGCCAAAATCTGAAATCTTGGGGCACATATCATTGTCTAGTAAGACGTTGCTG
GCTTTGAGATCTCTATGAATAATCC
>SD1-1_Exon800_17
TGCCAAGCCAAAATCTGAAATCTTGGGGCACATATCATTGTCTAGTAAGACGTTGCTGGCTTTGAGATCTCTATG
AATAATCCTTAGTCTAGAGTCTTGA
>SD1-1_Exon800_34
AAATCTTGGGGCACATATCATTGTCTAGTAAGACGTTGCTGGCTTTGAGATCTCTATGAATAATCCTTAGTCTAG
AGTCTTGATGAAGATAAAGAAGTCC
>SD1-1_Exon1100_0
CGAAAATAAAGTAGTCCAAGCTTTTGTTCGGCATGTACTCATAGATCAACATCTTTTCATCTCCTTGAACGCTGC
AACCGAGAAGCCTCACGAGGTTGCG
>SD1-1_Exon1100_23
TTGTTCGGCATGTACTCATAGATCAACATCTTTTCATCTCCTTGAACGCTGCAACCGAGAAGCCTCACGAGGTTG
CGATGCTGTAGCTTTGCAATCAATA
>SD1-1_Exon1100_45
TCAACATCTTTTCATCTCCTTGAACGCTGCAACCGAGAAGCCTCACGAGGTTGCGATGCTGTAGCTTTGCAATCA
ATACGACTTCATTTTTAAACTCTTC
>SD1-1_Exon1400_0
TGGGTATATTTTCTTAAATTTACCTTGTGAACAGGTCCAAATCCACCTTGTCCTAGCTTATTACTGTCTGAAAAG
TTATCAGTGGCTTTCACAATAGTGC
>SOx_exon200_0
ATGCCAGGAATAAAGGGACCTTCTGATTATTCACAAGAACCACCACGCCATCCTTGTCTTCAAATCAACTCCAAG
GAGCCCTTCAACGCTGAGCCGCCGC
>SOx_exon200_4
CAGGAATAAAGGGACCTTCTGATTATTCACAAGAACCACCACGCCATCCTTGTCTTCAAATCAACTCCAAGGAGC
CCTTCAACGCTGAGCCGCCGCGTTC
>SOx_exon450_0
TGGACTGCAGATATTGTTTCGATATATCTGGTTTAATTCAAACTCCCAAAAAGCTGTATATGAGAGATGTCAGGT
GAGTGATGtttttttttttttttt
>SOx_exon800_0
TTTTATTCAGTGTGCAGGTAATAGGAGGACTGCCATGAGCAAAACCCGAAAAGTGAGAGGAGTTGGCTGGGATGT
TTCTGCTATAGGGAATGGTTTGTTT
>SOx_exon800_2
TTATTCAGTGTGCAGGTAATAGGAGGACTGCCATGAGCAAAACCCGAAAAGTGAGAGGAGTTGGCTGGGATGTTT
CTGCTATAGGGAATGGTTTGTTTTC
>SOx_exon1200_0
AGCTGTCTGGGGTGGTGCCAAACTGGCTGATGTACTCGAGCTCGTTGGGATACCAAAGTTGACAAGTAGAACCCA
GTCTGGTGGAAAGCATGTCGAATTT
>SOx_exon1200_8
GGGGTGGTGCCAAACTGGCTGATGTACTCGAGCTCGTTGGGATACCAAAGTTGACAAGTAGAACCCAGTCTGGTG
GAAAGCATGTCGAATTTGTGAGCAT

>SOx_exon1200_23
TGGCTGATGTACTCGAGCTCGTTGGGATACCAAAGTTGACAAGTAGAACCCAGTCTGGTGGAAAGCATGTCGAAT
TTGTGAGCATTGATAAGTGTAAGGT
>SOx_exon1400_0
AAAAATGTAGGAGGAGAATGGAGGCCCATACAAGGCATCAATTCCACTTATTCAAGCCACTAACCCTGAAGCAGA
TGTTTTACTTGCTTATGAGATGAAT
>SOx_exon1400_8
AGGAGGAGAATGGAGGCCCATACAAGGCATCAATTCCACTTATTCAAGCCACTAACCCTGAAGCAGATGTTTTAC
TTGCTTATGAGATGAATGGAGAGGT
>SOx_exon1400_16
AATGGAGGCCCATACAAGGCATCAATTCCACTTATTCAAGCCACTAACCCTGAAGCAGATGTTTTACTTGCTTAT
GAGATGAATGGAGAGGTAAGAAAGT
>SOx_exon1700_0
CATGCTAAAGCCTCTCAACAGGGATCATGGTTATCCGTTGAGAGTAATTGTGCCCGGTGTTATAGGTGCACGTTC
TGTCAAGTGGCTCGATTCTATCAAC
>SOx_exon1700_16
AACAGGGATCATGGTTATCCGTTGAGAGTAATTGTGCCCGGTGTTATAGGTGCACGTTCTGTCAAGTGGCTCGAT
TCTATCAACATACTAGCAGAAGAAT
>SOx_exon1700_31
TATCCGTTGAGAGTAATTGTGCCCGGTGTTATAGGTGCACGTTCTGTCAAGTGGCTCGATTCTATCAACATACTA
GCAGAAGAATGCCAGGTAGAAGAGA
>SOx_exon1900_0
AGGGCTCCTTCATGCAAAAGGATTACAAGATGTTTCCACCATCTGTCGATTGGGATAACATCAACTGGTCTACCA
GGAGGCCACAAATGGATTTCCCTGT
>SOx_exon1900_6
CCTTCATGCAAAAGGATTACAAGATGTTTCCACCATCTGTCGATTGGGATAACATCAACTGGTCTACCAGGAGGC
CACAAATGGATTTCCCTGTTCAGGT
>SOx_exon2720_0
TTGGTTGGCAAGTCTGTAATTTGTTCTTTGGAAGACGTGCAGTCAATTAAACCCGGAAAGGTTAGTTCTCCTttt
tttttttttttttttttttttttt
>SOx_exon3500_0
ATAACAATTAGtGGATATGCAGCATCTGGAGGGGGCCGAGGAATCGAGAGAGTAGATGTGTCTATTGATGGTGGC
AAAACTTGGTTAGAAGCCTCTAGAT
>SOx_exon3500_21
GCATCTGGAGGGGGCCGAGGAATCGAGAGAGTAGATGTGTCTATTGATGGTGGCAAAACTTGGTTAGAAGCCTCT
AGATCTCAGAAAACCGGTATCCCTT
>SOx_exon3500_42
ATCGAGAGAGTAGATGTGTCTATTGATGGTGGCAAAACTTGGTTAGAAGCCTCTAGATCTCAGAAAACCGGTATC
CCTTACATATCAGATCACGAAAGCA
>StuctProt2-1_exon600_0
AACAGAGTAAGGTAGATGAAATAAACAAGCTTCTTGATCAGGAAATACTGCCTGCTTTGGAGAAGTTAAGAAAAG
AACGGATGCAGTATATGCAATGGGC
>StuctProt2-1_exon600_21
TAAACAAGCTTCTTGATCAGGAAATACTGCCTGCTTTGGAGAAGTTAAGAAAAGAACGGATGCAGTATATGCAAT
GGGCTAATGGAAATGCGGAGCTGGA
>StuctProt2-1_exon600_42
AAATACTGCCTGCTTTGGAGAAGTTAAGAAAAGAACGGATGCAGTATATGCAATGGGCTAATGGAAATGCGGAGC
TGGATCGACTTAAAAGGTTTTGTGT
>StuctProt2-1_exon900_0
TGACTGCTCAAAAGGAAGCTACTATGGGCGGGGAAGTTAAAAATTTGTCCGATGAAGTGGATTCAGTATCTAAAT
CTCTTGTGGCAAGAAGTGTTTGTGC
>StuctProt2-1_exon900_24
TGGGCGGGGAAGTTAAAAATTTGTCCGATGAAGTGGATTCAGTATCTAAATCTCTTGTGGCAAGAAGTGTTTGTG
CTGGAGAGTAAAGAGGATACTCTCA
>StuctProt2-1_exon900_48
CCGATGAAGTGGATTCAGTATCTAAATCTCTTGTGGCAAGAAGTGTTTGTGCTGGAGAGTAAAGAGGATACTCTC
AAGGGTGAGAAAGAGAACGCTGAAA

```
>StuctProt2-1_exon1250_0
ATTGAAGATTTGAAACAGTCTATAGAAGATAAAGCCAGTGCAGTGCAGAAGTCTGAACAAGGGGCAGCTGATCTG
AAGAAGAGATTTGAGGATCTTTCTA
>StuctProt2-1_exon1250_15
CAGTCTATAGAAGATAAAGCCAGTGCAGTGCAGAAGTCTGAACAAGGGGCAGCTGATCTGAAGAAGAGATTTGAG
GATCTTTCTAAGAGTTTGGGAGAGC
>StuctProt2-1_exon1250_29
TAAAGCCAGTGCAGTGCAGAAGTCTGAACAAGGGGCAGCTGATCTGAAGAAGAGATTTGAGGATCTTTCTAAGAG
TTTGGGAGAGCTTGAAAAGGAACAC
>StuctProt2-1_exon1550_0
GATGCCAAAGTTGCGGTTGGGACTGCTGAAACAGAATTGAAGCAGCTGAAAACAAAAATTAGCCATTGTGAAAAG
GAGCTGGGAGAGAAAACACGTCAAT
>StuctProt2-1_exon1550_14
GGTTGGGACTGCTGAAACAGAATTGAAGCAGCTGAAAACAAAAATTAGCCATTGTGAAAAGGAGCTGGGAGAGAA
AACACGTCAATTAAAGTCGAAGCGT
>StuctProt2-1_exon1550_28
AAACAGAATTGAAGCAGCTGAAAACAAAAATTAGCCATTGTGAAAAGGAGCTGGGAGAGAAAACACGTCAATTAA
AGTCGAAGCGTGCTGAAGCAGTTGA
>StuctProt2-1_exon1900_0
CACTTAGCAAATGTTCAGTTCAATTATCACGATCCTGTGAAAAATTTTGACCGGACAAAGGTGAAAGGAGTAGTT
GCAAAACTTATAAAAGTGAAGGATA
>StuctProt2-1_exon1900_11
TGTTCAGTTCAATTATCACGATCCTGTGAAAAATTTTGACCGGACAAAGGTGAAAGGAGTAGTTGCAAAACTTAT
AAAAGTGAAGGATAGCTCGGCAATG
>StuctProt2-1_exon1900_21
AATTATCACGATCCTGTGAAAAATTTTGACCGGACAAAGGTGAAAGGAGTAGTTGCAAAACTTATAAAAGTGAAG
GATAGCTCGGCAATGACAGCATTAG
>StuctProt2-1_exon2200_0
GGAAAAACTGCTTCAGAATGTTGATCTACGAAGAAGAGTAACAATTATACCTTTAAACAAAATCCAACCTAATAA
TGTACACCCCAGGGCTCAGCAGGCT
>StuctProt2-1_exon2200_6
ACTGCTTCAGAATGTTGATCTACGAAGAAGAGTAACAATTATACCTTTAAACAAAATCCAACCTAATAATGTACA
CCCCAGGGCTCAGCAGGCTGCTACT
>StuctProt2-1_exon2200_11
TTCAGAATGTTGATCTACGAAGAAGAGTAACAATTATACCTTTAAACAAAATCCAACCTAATAATGTACACCCCA
GGGCTCAGCAGGCTGCTACTAGATT
>StuctProt2-1_exon3440_0
AGATTGCAGACCTTCTGCCTCTTCAAAGAAAGTTCACAGACCTTAAAACGCAGTTAGAGCTTAAAATGCATGACC
TTTCCTTGTTCCAGAGCAGGGCAGA
>StuctProt2-1_exon3440_10
CCTTCTGCCTCTTCAAAGAAAGTTCACAGACCTTAAAACGCAGTTAGAGCTTAAAATGCATGACCTTTCCTTGTT
CCAGAGCAGGGCAGAGAAAAATGAG
>StuctProt2-1_exon3440_19
TCTTCAAAGAAAGTTCACAGACCTTAAAACGCAGTTAGAGCTTAAAATGCATGACCTTTCCTTGTTCCAGAGCAG
GGCAGAGAAAAATGAGCATCATAAG
>TFIID_exon50_0
TGGCTTTCAGATGATGAATGATTTCAAAAGTGTTATCTGATTAAGCTTCCCCTATACCTGCTTCAGCTTCATCTA
AGCTATGAGCATACTCATGCAGCAA
>TFIID_exon50_9
GATGATGAATGATTTCAAAAGTGTTATCTGATTAAGCTTCCCCTATACCTGCTTCAGCTTCATCTAAGCTATGAG
CATACTCATGCAGCAAGTAGTCACA
>TFIID_exon50_25
AAAAGTGTTATCTGATTAAGCTTCCCCTATACCTGCTTCAGCTTCATCTAAGCTATGAGCATACTCATGCAGCAA
GTAGTCACAAAGCTCCAAAAGCTGA
>TFIID_exon340_0
TTTACAATGTTCAGGTAGTCAGGGGCCTCCTTCTTGGACACTGGTTTTAAGAACAGATAGGACACCTCAGTATTA
TCTCTAAGTGTTTCTACAATCCGCT
```

>TFIID_exon340_10
TCAGGTAGTCAGGGGCCTCCTTCTTGGACACTGGTTTTAAGAACAGATAGGACACCTCAGTATTATCTCTAAGTG
TTTCTACAATCCGCTCTAAGATGTT
>TFIID_exon340_20
AGGGGCCTCCTTCTTGGACACTGGTTTTAAGAACAGATAGGACACCTCAGTATTATCTCTAAGTGTTTCTACAAT
CCGCTCTAAGATGTTGGCCAGACCA
>TFIID_exon1400_0
ACCTCTCCCCCTCTGCGTCGCTTTGTTGGAGGAACAAACTCTGCACCATATCTTCCTAACTCAACAGGTTTTCTT
TTTGCACCTCGATCTCTTTCCACCG
>TFIID_exon1400_17
TCGCTTTGTTGGAGGAACAAACTCTGCACCATATCTTCCTAACTCAACAGGTTTTCTTTTTGCACCTCGATCTCT
TTCCACCGTTCTTCTGTCAGTTCTT
>TFIID_exon1400_34
CAAACTCTGCACCATATCTTCCTAACTCAACAGGTTTTCTTTTTGCACCTCGATCTCTTTCCACCGTTCTTCTGT
CAGTTCTTCTTGTCCTGTAGTCTTC
>TFIID_exon2000_0
CAGAGGGTTTATGAGGTTCGACTTGGTCCCTCTCCGTATTTGCTGGTGGCTTTATAACAATAGATTTCTTATGAG
ATTCAGCTTGGCCTCTATCGGTATC
>TFIID_exon2000_10
ATGAGGTTCGACTTGGTCCCTCTCCGTATTTGCTGGTGGCTTTATAACAATAGATTTCTTATGAGATTCAGCTTG
GCCTCTATCGGTATCCACTTGATGC
>TFIID_exon2000_20
ACTTGGTCCCTCTCCGTATTTGCTGGTGGCTTTATAACAATAGATTTCTTATGAGATTCAGCTTGGCCTCTATCG
GTATCCACTTGATGCCGTATAACAA
>TFIID_exon2430_0
TGGGACTGACTGGAGGGTTCCAGCGACGTTGATTTGCCTGGAGTTTTTTCCAAATCAGTAGCTTCAATCTGCATT
TCTGGATCTTCTCCATACTTGGGGC
>TFIID_exon2430_14
GGGTTCCAGCGACGTTGATTTGCCTGGAGTTTTTTCCAAATCAGTAGCTTCAATCTGCATTTCTGGATCTTCTCC
ATACTTGGGGCAGTTCTTGTTGGTC
>TFIID_exon2430_28
TTGATTTGCCTGGAGTTTTTTCCAAATCAGTAGCTTCAATCTGCATTTCTGGATCTTCTCCATACTTGGGGCAGT
TCTTGTTGGTCCTCATGTGTCCAAG
>TFIID_exon2900_0
CTTAACATTGTCTCCCAATATTTTGACCTTAGTTAATGGTCCTGAGCTTGGCATACCATTCTTTTTCATTCCTTT
CAACTTTCCAGACGCGCTCCCTTTA
>TFIID_exon2900_10
TCTCCCAATATTTTGACCTTAGTTAATGGTCCTGAGCTTGGCATACCATTCTTTTTCATTCCTTTCAACTTTCCA
GACGCGCTCCCTTTAAAAATTCTAC
>TFIID_exon2900_20
TTTTGACCTTAGTTAATGGTCCTGAGCTTGGCATACCATTCTTTTTCATTCCTTTCAACTTTCCAGACGCGCTCC
CTTTAAAAATTCTACTTTCAATCTG
>TFIID_exon3270_0
GTAAACTTTCCACCCCAATACGTGGTTGTAAGCCAAAAGTCAACCCAGCATCTTCTGCAACAGCTTTATTTTTCT
TCTTCTTTTTTTTCTTTTGGTCATC
>TFIID_exon3270_5
CTTTCCACCCCAATACGTGGTTGTAAGCCAAAAGTCAACCCAGCATCTTCTGCAACAGCTTTATTTTTCTTCTTC
TTTTTTTTCTTTTGGTCATCCTCAT
>TFIID_exon3270_10
CACCCCAATACGTGGTTGTAAGCCAAAAGTCAACCCAGCATCTTCTGCAACAGCTTTATTTTTCTTCTTCTTTTT
TTTCTTTTGGTCATCCTCATCTAAC
>TFIID_exon3850_0
CATCCATGAGTAAACGACATAATTCAGCAGCTTCAGCAGCTTCGTCTTCAATTTCTTCTTCTGCCTGAGCCTTAT
ACGGACATCTTCTCATCTTCAGTCC
>TFIID_exon3850_10
TAAACGACATAATTCAGCAGCTTCAGCAGCTTCGTCTTCAATTTCTTCTTCTGCCTGAGCCTTATACGGACATCT
TCTCATCTTCAGTCCCTTAACAGCA

>TFIID_exon3850_20
AATTCAGCAGCTTCAGCAGCTTCGTCTTCAATTTCTTCTTCTGCCTGAGCCTTATACGGACATCTTCTCATCTTC
AGTCCCTTAACAGCATCTGCTTTGT
>TFIID_exon4230_0
TGTTGGATCAACCTTGACTCCTGCTGCAGCTTGTTCACTTGAAAGCTTGCGTATCATAGCAATACGGTGCCACCT
AGTTTGTTTCGCAATCACTTCATCA
>TFIID_exon4230_10
ACCTTGACTCCTGCTGCAGCTTGTTCACTTGAAAGCTTGCGTATCATAGCAATACGGTGCCACCTAGTTTGTTTC
GCAATCACTTCATCAGGAACATTGA
>TFIID_exon4230_20
CTGCTGCAGCTTGTTCACTTGAAAGCTTGCGTATCATAGCAATACGGTGCCACCTAGTTTGTTTCGCAATCACTT
CATCAGGAACATTGAACTTCAAAAG
>TFIID_exon5400_0
GCTGTTTTCTTCTTCATCACAGCATTTGAGACAGGAGCCTTTGGAGCGGTACGGACATAGCTAAATCCTAAACCT
CGACCAGAAGGATCACCAACACCAG
>TFIID_exon5400_15
ATCACAGCATTTGAGACAGGAGCCTTTGGAGCGGTACGGACATAGCTAAATCCTAAACCTCGACCAGAAGGATCA
CCAACACCAGTTATTTCCAGGCGCT
>TFIID_exon5400_30
ACAGGAGCCTTTGGAGCGGTACGGACATAGCTAAATCCTAAACCTCGACCAGAAGGATCACCAACACCAGTTATT
TCCAGGCGCTCAATATTATCTCTGT
>TFIID_exon5740_0
CCTGGCTTGTACATGCAACAAAATTGCTACTCAGATTCCAAGGAGTTATCTGCAGCTCCCGTTCAATGTGCGAGG
CAGCAGCCAGAGCTATAGCTTCATC
>TFIID_exon5740_10
ACATGCAACAAAATTGCTACTCAGATTCCAAGGAGTTATCTGCAGCTCCCGTTCAATGTGCGAGGCAGCAGCCAG
AGCTATAGCTTCATCAGGGAGTTGA
>TFIID_exon5740_20
AAATTGCTACTCAGATTCCAAGGAGTTATCTGCAGCTCCCGTTCAATGTGCGAGGCAGCAGCCAGAGCTATAGCT
TCATCAGGGAGTTGACTCATTGCAG
>TGH_exon100_0
GTTGAGTAGGGTGTGTCTGGGGGGACCTCAAAGCCCAGTTCTTTTCCCAGGGATTCTAGAAAGTCGCCTGCTATC
AAACGGTTCAGTGCTGTAGTTGCCA
>TGH_exon100_19
GGGGGACCTCAAAGCCCAGTTCTTTTCCCAGGGATTCTAGAAAGTCGCCTGCTATCAAACGGTTCAGTGCTGTAG
TTGCCACTTCAGTCTTCTTCTCTCG
>TGH_exon100_37
GTTCTTTTCCCAGGGATTCTAGAAAGTCGCCTGCTATCAAACGGTTCAGTGCTGTAGTTGCCACTTCAGTCTTCT
TCTCTCGATCCTGCGCTTTTTTGGT
>TGH_exon1300_0
TTTGAGCACCAGTTTGAGCAGCAGGAATATCTCTATTTGTGATAGCAACTTCTTCCGGTTTAGCAACTTTAACAG
AATCTGGAATGAAAAGTAGAGAATC
>TGH_exon1300_17
GCAGCAGGAATATCTCTATTTGTGATAGCAACTTCTTCCGGTTTAGCAACTTTAACAGAATCTGGAATGAAAAGT
AGAGAATCAATCTTACTTCTCACGC
>TGH_exon1300_33
TATTTGTGATAGCAACTTCTTCCGGTTTAGCAACTTTAACAGAATCTGGAATGAAAAGTAGAGAATCAATCTTAC
TTCTCACGCGTGGAGCTGGTGGTGG
>TGH_exon1740_0
CTTCCCCATAAAAGGATCAATGAGATCAAAGCGCTTGCATAAAACAGGTGAAGGTCGCCATTGAAACTCCTCCCT
TCTTGGGTACATTTTCTTCATAACC
>TGH_exon1740_11
AAGGATCAATGAGATCAAAGCGCTTGCATAAAACAGGTGAAGGTCGCCATTGAAACTCCTCCCTTCTTGGGTACA
TTTTCTTCATAACCAAATCTTCAGC
>TGH_exon1740_22
AGATCAAAGCGCTTGCATAAAACAGGTGAAGGTCGCCATTGAAACTCCTCCCTTCTTGGGTACATTTTCTTCATA
ACCAAATCTTCAGCATGGGCATTTT

>TGH_exon1950_0
CTCTAGTCCGCCAGAAGTGAACTGCATTCCACTGGCTAAAAAGTCCAAGGGCTGTGTTGAAACCATGCTTTCCTT
TCCCCGTTTTCCTTTCTCTATTGCC
>TGH_exon1950_20
ACTGCATTCCACTGGCTAAAAAGTCCAAGGGCTGTGTTGAAACCATGCTTTCCTTTCCCCGTTTTCCTTTCTCTA
TTGCCTCAGCAGCTGCCTCAAAGTC
>TGH_exon1950_40
AAGTCCAAGGGCTGTGTTGAAACCATGCTTTCCTTTCCCCGTTTTCCTTTCTCTATTGCCTCAGCAGCTGCCTCA
AAGTCCAATTTTTCACGAGCACGAG
>TGH_exon2160_0
CCTCCTTCATACTTTTCCTTAAGAAATTGCTCAAATCTTTCTTGCTTTGCAGGATCATCTTTGAATGGTTTTGCG
ACTTCCGGCAACTTACTAAATGAAT
>TGH_exon2160_23
AAATTGCTCAAATCTTTCTTGCTTTGCAGGATCATCTTTGAATGGTTTTGCGACTTCCGGCAACTTACTAAATGA
ATCAGGTTTTTTAAATGTGTCAGAT
>TGH_exon2160_46
TTGCAGGATCATCTTTGAATGGTTTTGCGACTTCCGGCAACTTACTAAATGAATCAGGTTTTTTAAATGTGTCAG
ATAGATTGAATTGAAGTTGAAACTC
>TPR_exon250_0
AGTACAGTCAATTAGTTCTAAAAGCTGTAATGCCTCACCGAGTGCTCCAGCATTTCCCGAGTTGGGCACATGAGT
TCCTTCACCCGGCTACCGTTTTTGT
>TPR_exon250_17
CTAAAAGCTGTAATGCCTCACCGAGTGCTCCAGCATTTCCCGAGTTGGGCACATGAGTTCCTTCACCCGGCTACC
GTTTTTGTATATCTTGAATGTTGGT
>TPR_exon250_34
TCACCGAGTGCTCCAGCATTTCCCGAGTTGGGCACATGAGTTCCTTCACCCGGCTACCGTTTTTGTATATCTTGA
ATGTTGGTACGATCCTCACATTCTC
>TPR_exon500_0
ACACTTACCTTAAGAAAATTTATGGATGGGTAACGACCACAAAGAGCATCCATGAATGGAGATATCTGTTTGCAT
TGCAGGTTGGAAGCCATCTTAAAAT
>TPR_exon500_10
TAAGAAAATTTATGGATGGGTAACGACCACAAAGAGCATCCATGAATGGAGATATCTGTTTGCATTGCAGGTTGG
AAGCCATCTTAAAATGGACAACAGA
>TPR_exon500_20
TATGGATGGGTAACGACCACAAAGAGCATCCATGAATGGAGATATCTGTTTGCATTGCAGGTTGGAAGCCATCTT
AAAATGGACAACAGAGATGCCTGTG
>TPR_exon700_0
CCTGGCAGAGATATTGCAGCTCTGAACTGCTCGAGACTGGATATTTCTTCTACTTCGCCACCGAACTTCATGTTA
TAAACCTCTTCACCGTGAGATTTCT
>TPR_exon700_9
GATATTGCAGCTCTGAACTGCTCGAGACTGGATATTTCTTCTACTTCGCCACCGAACTTCATGTTATAAACCTCT
TCACCGTGAGATTTCTTCAATGCAA
>TPR_exon700_25
ACTGCTCGAGACTGGATATTTCTTCTACTTCGCCACCGAACTTCATGTTATAAACCTCTTCACCGTGAGATTTCT
TCAATGCAACTTGTGCATGAAAAAG
>TPR_exon1000_0
CTTGCTATTAGATGCTGCCCTTCGGAGAAGGGCTTTTATGTAGTTTGGTTGGATACTTAAAGCTTGGTCGCAGTC
TTCAACAGATGACTCCCACCGTCCA
>TPR_exon1000_18
CCTTCGGAGAAGGGCTTTTATGTAGTTTGGTTGGATACTTAAAGCTTGGTCGCAGTCTTCAACAGATGACTCCCA
CCGTCCAAGCTTAAACCAACATGCT
>TPR_exon1000_35
TTATGTAGTTTGGTTGGATACTTAAAGCTTGGTCGCAGTCTTCAACAGATGACTCCCACCGTCCAAGCTTAAACC
AACATGCTGCTCTGTTACAATAAAG
>TPR_exon1200_0
AGATCATTCCCGCGTGCTCGTGCTCTGGCAACCAACCTCACATTGCTAAGCAGCACAGAAACTTCAACATTTCGA
GGATCAATCTGCCCAGCTTTCTCAG

>TPR_exon1200_11
GCGTGCTCGTGCTCTGGCAACCAACCTCACATTGCTAAGCAGCACAGAAACTTCAACATTTCGAGGATCAATCTG
CCCAGCTTTCTCAGCAGCTGTAACT
>TPR_exon1200_21
GCTCTGGCAACCAACCTCACATTGCTAAGCAGCACAGAAACTTCAACATTTCGAGGATCAATCTGCCCAGCTTTC
TCAGCAGCTGTAACTGCATTGTCGA
>TPR_exon1500_0
CCAAAGAACTTAATCTGTGAGGAATTGGTGCATGGTTCTAATTTAGGAACAACGGAAAGGCTTGATTCAGCATCA
TCAAGTTGATGCAGTTTTAAAAGGG
>TPR_exon1500_11
AATCTGTGAGGAATTGGTGCATGGTTCTAATTTAGGAACAACGGAAAGGCTTGATTCAGCATCATCAAGTTGATG
CAGTTTTAAAAGGGCTTCCACTCTA
>TPR_exon1500_22
AATTGGTGCATGGTTCTAATTTAGGAACAACGGAAAGGCTTGATTCAGCATCATCAAGTTGATGCAGTTTTAAAA
GGGCTTCCACTCTACACATAAAAAG
>TPR_exon1700_0
CTGAGGAGAAAAGTCTGCACCTGCAGCAATAGCATCATCAGCTTCCCTTAATGCACTTCTCCAATCCCTAATTCT
CCGGGCATCTGTACACTTGCTAAGA
>TPR_exon1700_14
CTGCACCTGCAGCAATAGCATCATCAGCTTCCCTTAATGCACTTCTCCAATCCCTAATTCTCCGGGCATCTGTAC
ACTTGCTAAGATGCCTTTCCACTGC
>TPR_exon1700_27
AATAGCATCATCAGCTTCCCTTAATGCACTTCTCCAATCCCTAATTCTCCGGGCATCTGTACACTTGCTAAGATG
CCTTTCCACTGCCTGCAACTTCTGC
>TPR_exon2600_0
CCTTAACAATAATGAAGCCAGTCTCTGGTGAGCCCTCCCATAATTAGGATCCAATCTAACAGCTTCTTCACATTC
CTTCACTGCCTCTCCTACTCTCCCT
>TPR_exon2600_19
AGTCTCTGGTGAGCCCTCCCATAATTAGGATCCAATCTAACAGCTTCTTCACATTCCTTCACTGCCTCTCCTACT
CTCCCTAAAGCCGTCAATGCGGCCG
>TPR_exon2600_38
CATAATTAGGATCCAATCTAACAGCTTCTTCACATTCCTTCACTGCCTCTCCTACTCTCCCTAAAGCCGTCAATG
CGGCCGCTCTGTTACTCCGGTAAGC
>TPR_exon3300_0
TATTCATACCCGATTCAGATCTCCTAGCGGTCGCGTTACACGTGTTCCGACCCGAAACGGATCTGGACGAACCGG
AACTGGAACTCGTGGTCGTCGTAGT
>TPR_exon3300_11
GATTCAGATCTCCTAGCGGTCGCGTTACACGTGTTCCGACCCGAAACGGATCTGGACGAACCGGAACTGGAACTC
GTGGTCGTCGTAGTGGTAGTGGTGG
>TPR_exon3300_22
CCTAGCGGTCGCGTTACACGTGTTCCGACCCGAAACGGATCTGGACGAACCGGAACTGGAACTCGTGGTCGTCGT
AGTGGTAGTGGTGGTTGTAGTCGTC
>TransportInhib_exon340_0
GTTGTAGGGACGGATTTTAGTTCGTTTGTGACCCACAACCATGGCTGCTTCATGCACTTCTGTGTTGGCAGTCTT
GCGATCTTGCTCTTCAGGGGTGCAG
>TransportInhib_exon340_14
TTTTAGTTCGTTTGTGACCCACAACCATGGCTGCTTCATGCACTTCTGTGTTGGCAGTCTTGCGATCTTGCTCTT
CAGGGGTGCAGTAAATCAAGCACGT
>TransportInhib_exon340_27
GTGACCCACAACCATGGCTGCTTCATGCACTTCTGTGTTGGCAGTCTTGCGATCTTGCTCTTCAGGGGTGCAGTA
AATCAAGCACGTGAGGCAAACTTGT
>TransportInhib_exon1000_0
AGGCATCTCCCTCGCCAGTAGCCTACAACCATTCATTGTTACATTGCAGGCTGACATCCAAAGTGACCTCATAGA
TTCGTACTTCTCCAAACCTGACAGC
>TransportInhib_exon1000_14
CCAGTAGCCTACAACCATTCATTGTTACATTGCAGGCTGACATCCAAAGTGACCTCATAGATTCGTACTTCTCCA
AACCTGACAGCAGTGCAGCATTTCC

>TransportInhib_exon1000_28
CCATTCATTGTTACATTGCAGGCTGACATCCAAAGTGACCTCATAGATTCGTACTTCTCCAAACCTGACAGCAGT
GCAGCATTTCCGAACGGGCAGTCTC
>TransportInhib_exon1500_0
TGGAGTCTTGGGCAACCAAAAGACACAGCCACAAACCCAGCTTCAGTGACTCCATGGACAATTTCATCATCAAAC
GGATCAGCAGGGAAAACTCGGAGTT
>TransportInhib_exon1500_14
ACCAAAAGACACAGCCACAAACCCAGCTTCAGTGACTCCATGGACAATTTCATCATCAAACGGATCAGCAGGGAA
AACTCGGAGTTCCTCAAGCAAAGGG
>TransportInhib_exon1500_28
CCACAAACCCAGCTTCAGTGACTCCATGGACAATTTCATCATCAAACGGATCAGCAGGGAAAACTCGGAGTTCCT
CAAGCAAAGGGCAGTTCAATCCAAC
>TransportInhib_exon2000_0
CAAAGCAGCATAGCTCAAGTTCAAGAACGTTAGATGAGTGCATGCAAGGTACAAGGCCGGGAGATATATGCCCGT
GGCATCCCACAAACCAGATAGCGTA
>TransportInhib_exon2000_20
TCAAGAACGTTAGATGAGTGCATGCAAGGTACAAGGCCGGGAGATATATGCCCGTGGCATCCCACAAACCAGATA
GCGTATGCATATTCTTGCAATTACT
>TransportInhib_exon2000_39
GCATGCAAGGTACAAGGCCGGGAGATATATGCCCGTGGCATCCCACAAACCAGATAGCGTATGCATATTCTTGCA
ATTACTTAAAGCGCTTTCAAGCTCT
>TransportInhib_exon2100_0
CTTTCAAGCTCTTCATATTCGCGGATAGTAAGTTCTTGCGAGAACAAACCCGTACCGAGCTCAGCTAATTGAGGG
GCATTAACAAGAAGCCTTTGTAATT
>TransportInhib_exon2100_14
ATATTCGCGGATAGTAAGTTCTTGCGAGAACAAACCCGTACCGAGCTCAGCTAATTGAGGGGCATTAACAAGAAG
CCTTTGTAATTGTTCCAAGGATACA
>TransportInhib_exon2100_28
TAAGTTCTTGCGAGAACAAACCCGTACCGAGCTCAGCTAATTGAGGGGCATTAACAAGAAGCCTTTGTAATTGTT
CCAAGGATACACTCCTATTCAACTT
>UBX_exon60_0
AAGATGGAAAGGTCGGAATCTTTGTCAACACTTGCATTCAAAGGCTCAATTCCTGAAGCAATTCTTGAAGCTAAG
AACCAGAAGAAGCTTTTCGTGGTCT
>UBX_exon60_10
GGTCGGAATCTTTGTCAACACTTGCATTCAAAGGCTCAATTCCTGAAGCAATTCTTGAAGCTAAGAACCAGAAGA
AGCTTTTCGTGGTCTACATTTCAGG
>UBX_exon60_20
TTTGTCAACACTTGCATTCAAAGGCTCAATTCCTGAAGCAATTCTTGAAGCTAAGAACCAGAAGAAGCTTTTCGT
GGTCTACATTTCAGGTAGCCTCTTC
>UBX_exon450_0
TCTTATCATGGTGGATTCAGGTGAAAGAGTCATTGTTAAAGTATTGCATTCTATTGCATATCCGAGGTGGAAGCA
CCGATGCTGCAAATTTTTCTGCCAT
>UBX_exon450_11
TGGATTCAGGTGAAAGAGTCATTGTTAAAGTATTGCATTCTATTGCATATCCGAGGTGGAAGCACCGATGCTGCA
AATTTTTCTGCCATATGTATCCTAA
>UBX_exon450_22
GAAAGAGTCATTGTTAAAGTATTGCATTCTATTGCATATCCGAGGTGGAAGCACCGATGCTGCAAATTTTTCTGC
CATATGTATCCTAATCCTCGATTGT
>UBX_exon1500_0
GAAACAACGGCAGCTGTCCTTAGTGCTGCACTTGCTTCAAAGAAATATGAATCATCCAGTTCTGGGGCATCTACT
GTTAACCTGTCTGAGCAAGGAAGTT
>UBX_exon1500_15
GTCCTTAGTGCTGCACTTGCTTCAAAGAAATATGAATCATCCAGTTCTGGGGCATCTACTGTTAACCTGTCTGAG
CAAGGAAGTTCTTCAAGTGCTTCTG
>UBX_exon1500_30
CTTGCTTCAAAGAAATATGAATCATCCAGTTCTGGGGCATCTACTGTTAACCTGTCTGAGCAAGGAAGTTCTTCA
AGTGCTTCTGTTCCATCAACCACAA

```
>UBX_exon1900_0
GAGAAAAAATCCTGAGTTGGTTGAAAAGAGTAGTTCTGAATCATTTAGCGCTGACAACTTGGCTAATATTGTGGAT
GAACAATGTGGTATTACCAATGAAG
>UBX_exon1900_9
CCTGAGTTGGTTGAAAAGAGTAGTTCTGAATCATTTAGCGCTGACAACTTGGCTAATATTGTGGATGAACAATGT
GGTATTACCAATGAAGATACGAGGA
>UBX_exon1900_25
AGAGTAGTTCTGAATCATTTAGCGCTGACAACTTGGCTAATATTGTGGATGAACAATGTGGTATTACCAATGAAG
ATACGAGGACAGTTGTTAGTTCTGT
>UBX_exon2300_0
CATTTAAATATCCGATTACCTGACAGTAGTAGCCTACAAGAAAAAATTTCCTGTGACATACACTCTGAGTATGATT
AAAGACTATGTGGATAGAAACCAAT
>UBX_exon2300_23
CAGTAGTAGCCTACAAGAAAAAATTTCCTGTGACATACACTCTGAGTATGATTAAAGACTATGTGGATAGAAACCA
ATCAAGTGGCATGAGCTCCTACGAT
>UBX_exon2300_45
TTTCCTGTGACATACACTCTGAGTATGATTAAAGACTATGTGGATAGAAACCAATCAAGTGGCATGAGCTCCTAC
GATCTTGCCATTCCTTATCCTCGCA
>UBX_exon3000_0
CAGGAAGCAATGGGGGATATTTTGCATACGTCAGAAGCCTTTTGTCTTATGTAAATCCTTTTTCATATCTCGGTG
GTGGTGCCAGCTCTTCAACGACTGG
>UBX_exon3000_16
ATATTTTGCATACGTCAGAAGCCTTTTGTCTTATGTAAATCCTTTTTCATATCTCGGTGGTGGTGCCAGCTCTTC
AACGACTGGACAGGAATCTCAAAGT
>UBX_exon3000_32
AGAAGCCTTTTGTCTTATGTAAATCCTTTTTCATATCTCGGTGGTGGTGCCAGCTCTTCAACGACTGGACAGGAA
TCTCAAAGTGGCATCTGGGAATACA
>UBX_exon4000_0
ATGGCAGCAATATCCACACCCTAAAACGTGATGAAGATGATGACCGTTTCAACGATAGAAATCCGTTCTGGAATG
GAAATTCTACACAGTATGGTGGTAG
>UBX_exon4000_10
TATCCACACCCTAAAACGTGATGAAGATGATGACCGTTTCAACGATAGAAATCCGTTCTGGAATGGAAATTCTAC
ACAGTATGGTGGTAGCAGTGACAGC
>UBX_exon4000_20
CTAAAACGTGATGAAGATGATGACCGTTTCAACGATAGAAATCCGTTCTGGAATGGAAATTCTACACAGTATGGT
GGTAGCAGTGACAGCAAATAATAGG
>Valine_exon3400_0
AGAAATTGTACAGAGGAGCTCAAAACAATGAGATGCGCCTTGGCCTTTGTTCAAGAAGCAATGATGTCGTGGAGC
CAATGATAAAGCCCCAGTGGTATGT
>Valine_exon3400_10
CAGAGGAGCTCAAAACAATGAGATGCGCCTTGGCCTTTGTTCAAGAAGCAATGATGTCGTGGAGCCAATGATAAA
GCCCCAGTGGTATGTTAACTGCAGT
>Valine_exon3400_20
CAAAACAATGAGATGCGCCTTGGCCTTTGTTCAAGAAGCAATGATGTCGTGGAGCCAATGATAAAGCCCCAGTGG
TATGTTAACTGCAGTAGCATGGCAA
>Valine_exon3900_0
AGGTGGCTTGACAATATCCGTGATTGGTGTATTTCAAGGCAGCTTTGGTGGGGTCACCGAATTCCTGCATGGTAT
GTTACTTTTGAAGATGATGAACAGA
>Valine_exon3900_10
ACAATATCCGTGATTGGTGTATTTCAAGGCAGCTTTGGTGGGGTCACCGAATTCCTGCATGGTATGTTACTTTTG
AAGATGATGAACAGAAGGAACTTGG
>Valine_exon3900_20
TGATTGGTGTATTTCAAGGCAGCTTTGGTGGGGTCACCGAATTCCTGCATGGTATGTTACTTTTGAAGATGATGA
ACAGAAGGAACTTGGTGCTTACAAT
>Valine_exon4200_0
CTGATTTAAAAGCATTTTATCCAACATCGGTCCTTGAAACTGGGCATGATATTCTCTTCTTTTGGGTTGCTCGGA
TGGTTATGTTGGGAATTATATTGGG
```

>Valine_exon4200_10
AGCATTTTATCCAACATCGGTCCTTGAAACTGGGCATGATATTCTCTTCTTTTGGGTTGCTCGGATGGTTATGTT
GGGAATTATATTGGGAGGTGATGTG
>Valine_exon4200_20
CCAACATCGGTCCTTGAAACTGGGCATGATATTCTCTTCTTTTGGGTTGCTCGGATGGTTATGTTGGGAATTATA
TTGGGAGGTGATGTGCCATTCAGTA
>Valine_exon4500_0
TCCTCTTGAGGTGATAAATGGGATATCACTTGAAGGTCTTCATAAGAGGCTCGAAGGGGGTAACTTGGATCCTAA
TGAGCTGGCCACTGCCAAAGAAGGG
>Valine_exon4500_10
GTGATAAATGGGATATCACTTGAAGGTCTTCATAAGAGGCTCGAAGGGGGTAACTTGGATCCTAATGAGCTGGCC
ACTGCCAAAGAAGGGCAGCGGAAAG
>Valine_exon4500_20
GGATATCACTTGAAGGTCTTCATAAGAGGCTCGAAGGGGGTAACTTGGATCCTAATGAGCTGGCCACTGCCAAAG
AAGGGCAGCGGAAAGATTTTCCTAA
>Valine_exon4800_0
GTTGGATATCGTCAATGGTGTAACAAATTATGGAATGCTGTTCGTTTTGCCATGAGCAAGCTTCCAGATGACTAT
ACTCCTCCATCATCCATAAATCCCG
>Valine_exon4800_10
GTCAATGGTGTAACAAATTATGGAATGCTGTTCGTTTTGCCATGAGCAAGCTTCCAGATGACTATACTCCTCCAT
CATCCATAAATCCCGAGGCAATGCC
>Valine_exon4800_20
TAACAAATTATGGAATGCTGTTCGTTTTGCCATGAGCAAGCTTCCAGATGACTATACTCCTCCATCATCCATAAA
TCCCGAGGCAATGCCTTTCAGTTGT
>Valine_exon5150_0
GGCCTGCGTTTGCTTCATCCGTTTATGCCATTTGTTACCGAAGAACTGTGGCAGCGCCTTCCAGGAGTAAAGAGC
CATACAAAGAAAGACTCTATAATGA
>Valine_exon5150_10
TGCTTCATCCGTTTATGCCATTTGTTACCGAAGAACTGTGGCAGCGCCTTCCAGGAGTAAAGAGCCATACAAAGA
AAGACTCTATAATGATATGCGAATA
>Valine_exon5150_20
GTTTATGCCATTTGTTACCGAAGAACTGTGGCAGCGCCTTCCAGGAGTAAAGAGCCATACAAAGAAAGACTCTAT
AATGATATGCGAATACCCATCACCA
>Valine_exon5400_0
CCTTTGCAGAGCTGGACAAATGAAAAGGTGGAATATGAGATGGACCTTGTAGAGTCAACAGTAAGGTCTCTTAGG
TCACTTCGAGCTGAGCTGCTTGCTA
>Valine_exon5400_10
GCTGGACAAATGAAAAGGTGGAATATGAGATGGACCTTGTAGAGTCAACAGTAAGGTCTCTTAGGTCACTTCGAG
CTGAGCTGCTTGCTAAGCAGAAGAA
>Valine_exon5400_20
TGAAAAGGTGGAATATGAGATGGACCTTGTAGAGTCAACAGTAAGGTCTCTTAGGTCACTTCGAGCTGAGCTGCT
TGCTAAGCAGAAGAATGAAAGGTTC
>Valine_exon5900_0
AGGTTCTACTGAGTGGTGTAGATGATGCTCCAGCCGGATGTGCAGTCGAAAATGTCAACGAAAACCTTAAAGTTT
ATCTTAAGGTTCAAGGAGCCCTAAA
>Valine_exon5900_10
GAGTGGTGTAGATGATGCTCCAGCCGGATGTGCAGTCGAAAATGTCAACGAAAACCTTAAAGTTTATCTTAAGGT
TCAAGGAGCCCTAAATGCAGAAGCA
>Valine_exon5900_20
GATGATGCTCCAGCCGGATGTGCAGTCGAAAATGTCAACGAAAACCTTAAAGTTTATCTTAAGGTTCAAGGAGCC
CTAAATGCAGAAGCAGAACGAGAAA
>WBC1_exon300_0
AGATCGATCCACTTCGACCGTCCCACATTGATTTGGAACACGTTTTCCAGGATGTATTCCCCTGGAATCTTGGGA
AGCTCTGGGGATTGATTATCAAACA
>WBC1_exon300_15
GACCGTCCCACATTGATTTGGAACACGTTTTCCAGGATGTATTCCCCTGGAATCTTGGGAAGCTCTGGGGATTGA
TTATCAAACAGCAAGCCCCTCAGAT

>WBC1_exon300_30
ATTTGGAACACGTTTTCCAGGATGTATTCCCCTGGAATCTTGGGAAGCTCTGGGGATTGATTATCAAACAGCAAG
CCCCTCAGATCATTCTGGTATTGTC
>WBC1_exon800_0
CCTGAATCCCTGCTCCTATGATGATCCCCATGAGGAAATTGGGGACGATACTGGCGATGGCCATCATCAAGCTCT
CCACAACAGTAACACTTGCGTAAAG
>WBC1_exon800_8
CCTGCTCCTATGATGATCCCCATGAGGAAATTGGGGACGATACTGGCGATGGCCATCATCAAGCTCTCCACAACA
GTAACACTTGCGTAAAGGCACAATA
>WBC1_exon800_24
TCCCCATGAGGAAATTGGGGACGATACTGGCGATGGCCATCATCAAGCTCTCCACAACAGTAACACTTGCGTAAA
GGCACAATACGAAGAACAGATAATG
>WBC1_exon1330_0
CCAGAATGGCATTGTAGCTGGTTCCAACATTGAAATAAATGGTTCCAATGCAAATAGTGACTACAACATATATCA
GCAGCCTTAGCCAATAATAGCCAAA
>WBC1_exon1330_20
GTTCCAACATTGAAATAAATGGTTCCAATGCAAATAGTGACTACAACATATATCAGCAGCCTTAGCCAATAATAG
CCAAAGTCCCGAGACATGTTTATGA
>WBC1_exon1330_39
TGGTTCCAATGCAAATAGTGACTACAACATATATCAGCAGCCTTAGCCAATAATAGCCAAAGTCCCGAGACATGT
TTATGAACGAACGCTTGGTTAAAGT
>WBC1_exon1600_0
AACTTTGGATATCTCTTCGACCTTTTCCTTTGCAGCATAACATTGCTGCGATGTCCGGTAGAAGCTAATAAGAGT
TCTGATAGCTTCAGTTGTTGTTATC
>WBC1_exon1600_15
TTCGACCTTTTCCTTTGCAGCATAACATTGCTGCGATGTCCGGTAGAAGCTAATAAGAGTTCTGATAGCTTCAGT
TGTTGTTATCTTCTCCAGGGGATCA
>WBC1_exon1600_29
TTGCAGCATAACATTGCTGCGATGTCCGGTAGAAGCTAATAAGAGTTCTGATAGCTTCAGTTGTTGTTATCTTCT
CCAGGGGATCATCACTTGCCTCAAA
>WBC1_exon1850_0
CCGCAATTTCATTGATCCTTTTAGAGTAGCCTTAACTCTATCAAAGTCAGAATTGATGCATCTAAGAAAATGATC
CGATGGATTTCTCAAGGCAGGGCAT
>WBC1_exon1850_8
TCATTGATCCTTTTAGAGTAGCCTTAACTCTATCAAAGTCAGAATTGATGCATCTAAGAAAATGATCCGATGGAT
TTCTCAAGGCAGGGCATGGAAATCC
>WBC1_exon1850_23
GAGTAGCCTTAACTCTATCAAAGTCAGAATTGATGCATCTAAGAAAATGATCCGATGGATTTCTCAAGGCAGGGC
ATGGAAATCCGGCTTGCGCGAAGAA
>WBC1_exon2090_0
AAATAAATGGTTTTGCCGTCAGATAGCAAATATAACTGGTCAAATAGCTCAAAGACTTCACTGCTGGGCTGATGA
ACGGAAGCTATTACAGTTCTACCAT
>WBC1_exon2090_14
GCCGTCAGATAGCAAATATAACTGGTCAAATAGCTCAAAGACTTCACTGCTGGGCTGATGAACGGAAGCTATTAC
AGTTCTACCATCTCTAGACAGGCCA
>WBC1_exon2090_28
AATATAACTGGTCAAATAGCTCAAAGACTTCACTGCTGGGCTGATGAACGGAAGCTATTACAGTTCTACCATCTC
TAGACAGGCCACGTAAAGTCTGGGT
>WBC1_exon2350_0
GGCCTCGTCAAAATTTCAAGAGCAATGCTGACCCTCCTCTTTTCTCCTCCACTGATCCCACGCAAATGCCAATTC
CCAATTACAGTATCAGCGCAATCTT
>WBC1_exon2350_11
AATTTCAAGAGCAATGCTGACCCTCCTCTTTTCTCCTCCACTGATCCCACGCAAATGCCAATTCCCAATTACAGT
ATCAGCGCAATCTTGCAGCCCCATC
>WBC1_exon2350_22
CAATGCTGACCCTCCTCTTTTCTCCTCCACTGATCCCACGCAAATGCCAATTCCCAATTACAGTATCAGCGCAAT
CTTGCAGCCCCATCTCGATAATCGT

```
>YSL1_exon300_0
TTTTTATACGGACATGGACCAAAGTTGTTGAGAAGGCTGGATTTACGGCCAAGCCTTTCACAAGACAAGAAAACA
CAATGATTCAAACATGTGCAGTTGC
>YSL1_exon300_11
ACATGGACCAAAGTTGTTGAGAAGGCTGGATTTACGGCCAAGCCTTTCACAAGACAAGAAAACACAATGATTCAA
ACATGTGCAGTTGCATGTTACAGCA
>YSL1_exon300_21
AAGTTGTTGAGAAGGCTGGATTTACGGCCAAGCCTTTCACAAGACAAGAAAACACAATGATTCAAACATGTGCAG
TTGCATGTTACAGCATAGCTATTGG
>YSL1_exon570_0
GAATTGTCTGGAGTACACACTGAAGGGAACTCTGCAAACGCAGTAAAAGAACCAGGATTTGGGTGGATGACTGGC
TTCCTTTTTGTAGTTTGCTTTGTTG
>YSL1_exon570_14
ACACACTGAAGGGAACTCTGCAAACGCAGTAAAAGAACCAGGATTTGGGTGGATGACTGGCTTCCTTTTTGTAGT
TTGCTTTGTTGGTCTTTTTGTTTTA
>YSL1_exon570_27
AACTCTGCAAACGCAGTAAAAGAACCAGGATTTGGGTGGATGACTGGCTTCCTTTTTGTAGTTTGCTTTGTTGGT
CTTTTTGTTTTAATTCCTCTCAGAA
>YSL1_exon1000_0
TCTTTACAGATCATGATAGTGGACCTCAAATTGACATATCCAAGTGGCTTGGCAACTGCAGTTCTCATCAATGGC
TTCCACAGCCAGGGCGATAAGTCAG
>YSL1_exon1000_6
CAGATCATGATAGTGGACCTCAAATTGACATATCCAAGTGGCTTGGCAACTGCAGTTCTCATCAATGGCTTCCAC
AGCCAGGGCGATAAGTCAGCCAAGT
>YSL1_exon1000_11
CATGATAGTGGACCTCAAATTGACATATCCAAGTGGCTTGGCAACTGCAGTTCTCATCAATGGCTTCCACAGCCA
GGGCGATAAGTCAGCCAAGTAAGCA
>YSL1_exon1200_0
GGATTCCTCCAGTATTTTTCAGCCAGTTTCCTATGGGGTTTTTTCCAATGGTTTTTCTCTGGAAAAGAAGATTGT
GGTTTCAAACAGTTCCCTACTTTCG
>YSL1_exon1200_11
GTATTTTTCAGCCAGTTTCCTATGGGGTTTTTTCCAATGGTTTTTCTCTGGAAAAGAAGATTGTGGTTTCAAACA
GTTCCCTACTTTCGGACTTAAAGCC
>YSL1_exon1200_22
CCAGTTTCCTATGGGGTTTTTTCCAATGGTTTTTCTCTGGAAAAGAAGATTGTGGTTTCAAACAGTTCCCTACTT
TCGGACTTAAAGCCTGGAAGCAAAC
>YSL1_exon1470_0
ATTCTTCTTTGATTTTAGCCTGACCTATGTGGGGGCAGGAATGATCTGTTCTCACCTCGTTAACTTGTCTTTACT
ATTTGGAGCCGTGCTTTCATACGGA
>YSL1_exon1470_11
ATTTTAGCCTGACCTATGTGGGGGCAGGAATGATCTGTTCTCACCTCGTTAACTTGTCTTTACTATTTGGAGCCG
TGCTTTCATACGGAATTATGTGGCC
>YSL1_exon1470_22
ACCTATGTGGGGGCAGGAATGATCTGTTCTCACCTCGTTAACTTGTCTTTACTATTTGGAGCCGTGCTTTCATAC
GGAATTATGTGGCCACTTATTAATC
>YSL1_exon1740_0
AGGTTTTTGTGTCTGTTGCTCTCATTCTTGGTGATGGCCTTTACAATTTCCTCAAGATATTCTGTTTCACGCTTA
TTAATGTCCATGGTAGATTGAAGGA
>YSL1_exon1740_10
GTCTGTTGCTCTCATTCTTGGTGATGGCCTTTACAATTTCCTCAAGATATTCTGTTTCACGCTTATTAATGTCCA
TGGTAGATTGAAGGACAAAGGTCGA
>YSL1_exon1740_20
CTCATTCTTGGTGATGGCCTTTACAATTTCCTCAAGATATTCTGTTTCACGCTTATTAATGTCCATGGTAGATTG
AAGGACAAAGGTCGAAATACAGGTA
>YSL1_exon2170_0
ACCGACATTAACATGGCCTATAACTATGGGAAAGTAGCCCTTTTCGTGTTGGCAGCATTGACGGGCAAGGAGAAC
GGTGTGGTAGCGGGGCTTGCCGGAT
```

>YSL1_exon2170_9
AACATGGCCTATAACTATGGGAAAGTAGCCCTTTTCGTGTTGGCAGCATTGACGGGCAAGGAGAACGGTGTGGTA
GCGGGGCTTGCCGGATGTGGTCTCA
>YSL1_exon2170_25
ATGGGAAAGTAGCCCTTTTCGTGTTGGCAGCATTGACGGGCAAGGAGAACGGTGTGGTAGCGGGGCTTGCCGGAT
GTGGTCTCATCAAATCTGTTGTTTC
>YSL1_exon2770_0
TGATGGTTCCAGCCGTTGCTTCTGGACTCATATGTGGGGAAGGGCTATGGATTCTACCAGCTTCAATTCTTGCCT
TGGCCAAAATTAGTCCTCCCATTTG
>YSL1_exon2770_14
GTTGCTTCTGGACTCATATGTGGGGAAGGGCTATGGATTCTACCAGCTTCAATTCTTGCCTTGGCCAAAATTAGT
CCTCCCATTTGCATGAAGTTCCTGC
>YSL1_exon2770_27
TCATATGTGGGGAAGGGCTATGGATTCTACCAGCTTCAATTCTTGCCTTGGCCAAAATTAGTCCTCCCATTTGCA
TGAAGTTCCTGCCTTCTTAGCAAAA
