# Supplementary material for: Gene count from target sequence capture places three whole genome duplication events in Hibiscus L. (Malvaceae)
Source: BMC Ecol Evol. 2021 Jun 2;21:107. doi: 10.1186/s12862-021-01751-7 (PMC8170824; doi:10.1186/s12862-021-01751-7)
Supplement: Supplementary file 2 — Additional file 2: Table S1. Species name and accession information. BONN stand for Botanische Gärten der Universität Bonn; The Royal Botanic Gardens, Kew; USDA National Plant Germplasm System. Hibiscus trionum is kept as a cultivar at Christchurch Botanic Gardens (NZ). Number of reads after quality trimming, in parenthesis the percentage of reduced reads from the original raw data. Percent of GC content after the trimming. Percentage of recovered loci per species and the total number of loci. Percentage of recovered probes (designed over exons) per species. Vouchers are deposited at the Gothenburg herbarium (GB). [file 12862_2021_1751_MOESM2_ESM.docx]

Additional file 2. Species name and accession information. BONN stand for Botanische Gärten der Universität Bonn; The Royal Botanic Gardens, Kew; USDA National Plant Germplasm System. *Hibiscus trionum* is kept as a cultivar at Christchurch Botanic Gardens (NZ). Number of reads after quality trimming, in parenthesis the percentage of reduced reads from the original raw data. Percent of GC content after the trimming. Percentage of recovered loci per species and the total number of loci. Percentage of recovered probes (designed over exons) per species. Vouchers are deposited at the Gothenburg herbarium (GB).

| **Species name** | **Taxon label in study** | **Accession number** | **Collection** | **Voucher barcode** | **# trimmed reads (percentage of reads after trimming)** | **% GC** | **% Recovered loci (recovered loci/tot. loci)** | **% Recovered probes** |
| --- | --- | --- | --- | --- | --- | --- | --- | --- |
| *Hibiscus cannabinus* L. | *H. cannabinus1* | XX-0-BONN-19883 | BONN | GB-0218216 | 1460168 (92%) | 41.3 | 100% (87/87) | 88% |
| *H. mechowii*  Garcke | *H. mechowii* | PI 500776 | USDA | GB-0218217 | 1892534 (92%) | 41.4 | 98.8% (86/87) | 56% |
| *H. cannabinus* | *H. cannabinus2* | PI 638930 | USDA | GB- 0218218 | 1231966 (90%) | 41.1 | 98.8% (86/87) | 60% |
| *H. trionum* L. | *H. trionum* | REF: 10 | Botanic Garden Christchurch (NZ) | GB- 0218219 | 662426 (92%) | 41.3 | 100% (87/87 | 59% |
| *H. cannabinus* | *H. cannabinus3* | PI 189210 | USDA | GB- 0218220 | 1180578 (91%) | 41.0 | 98.8% (86/87) | 59% |
| *Pavonia triloba*  Guill. & Perr. | *P. triloba* | Ref: 84822 | KEW | GB- 0218221 | 1139782 (92%) | 40.3 | 98.8% (86/87) | 58% |
| *H. syriacus* L. | *H. syriacus* | GCA_001696755.1 | GenBank |  |  |  |  |  |
| *Gossypium raimondii*  Ulbr. | *Gossypium raimondii* | GCF_000327365.1 | GenBank |  |  |  |  |  |
