# Supplementary figures and images for: Gene count from target sequence capture places three whole genome duplication events in Hibiscus L. (Malvaceae)
Source: BMC Ecol Evol. 2021 Jun 2;21:107. doi: 10.1186/s12862-021-01751-7 (PMC8170824; doi:10.1186/s12862-021-01751-7)

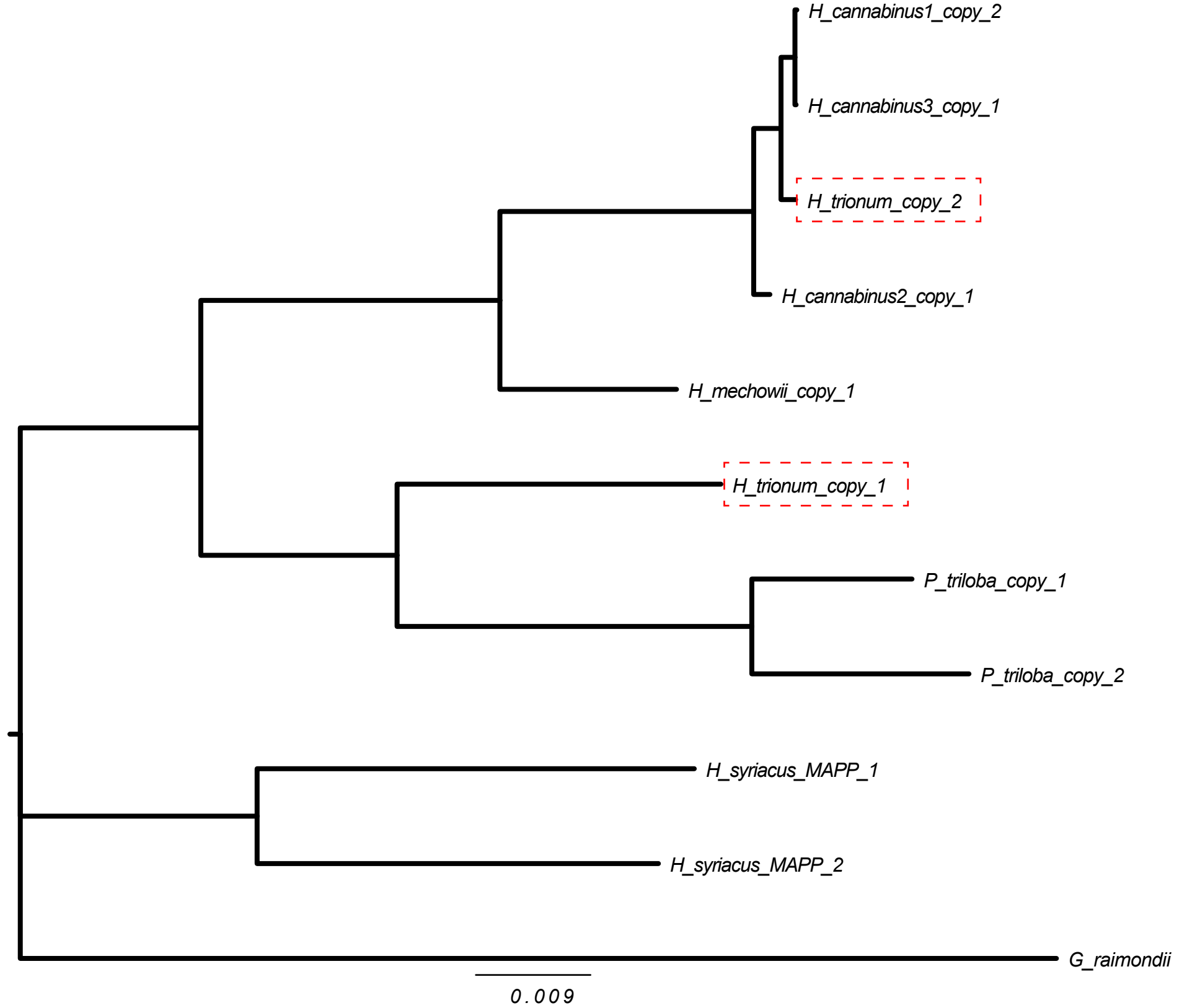

Supplement: Supplementary file 3 — Additional file 3: Fig. S1. Phylogenetic relationships of a single-copy gene (SCG) Oxysterol-D1 inferred by MrBayes. [file 12862_2021_1751_MOESM3_ESM.pdf]

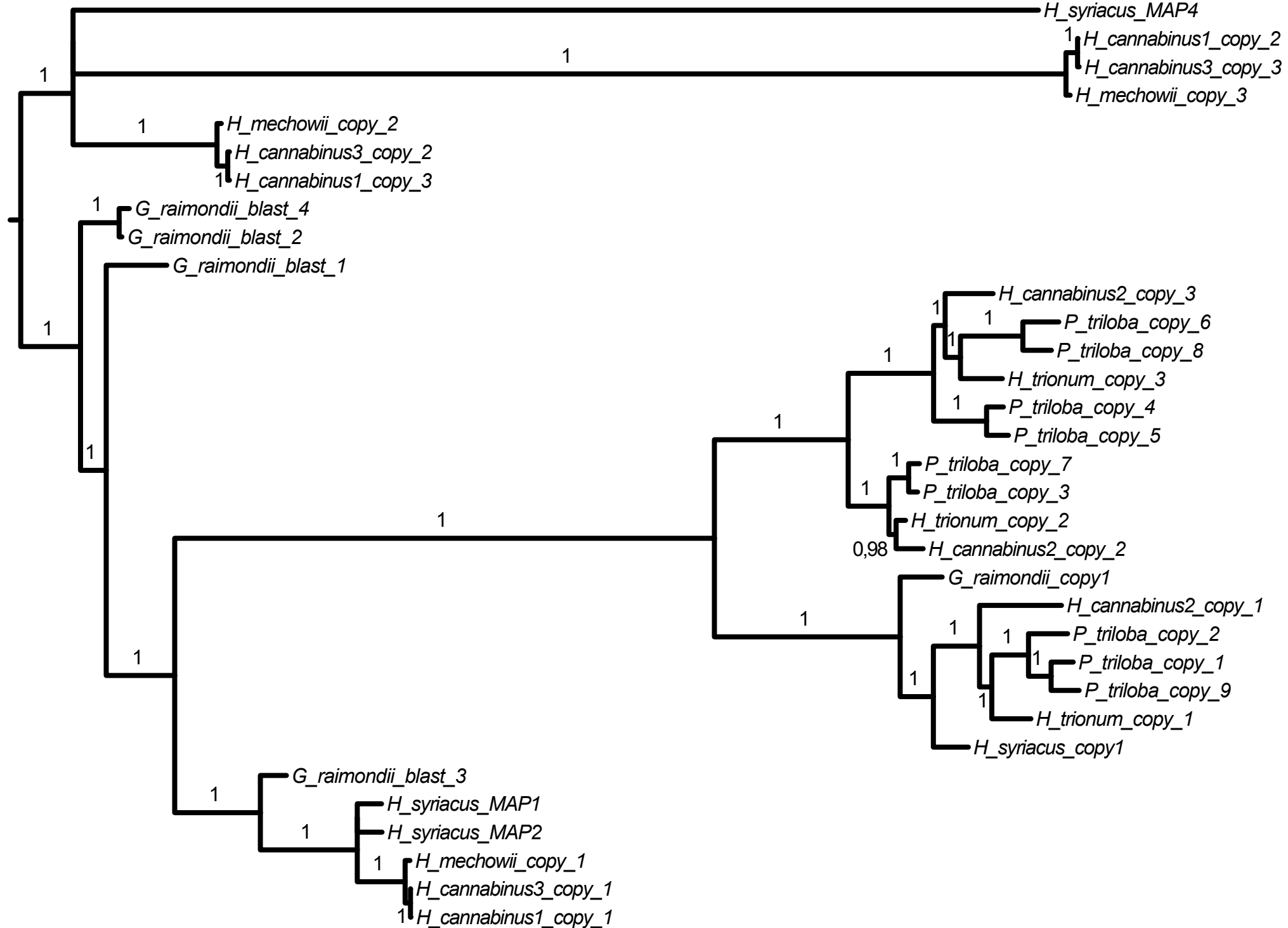

0.05

Supplement: Supplementary file 4 — Additional file 4: Fig. S2. MrBayes trees of multi-copy genes (MSC). [file 12862_2021_1751_MOESM4_ESM.pdf]

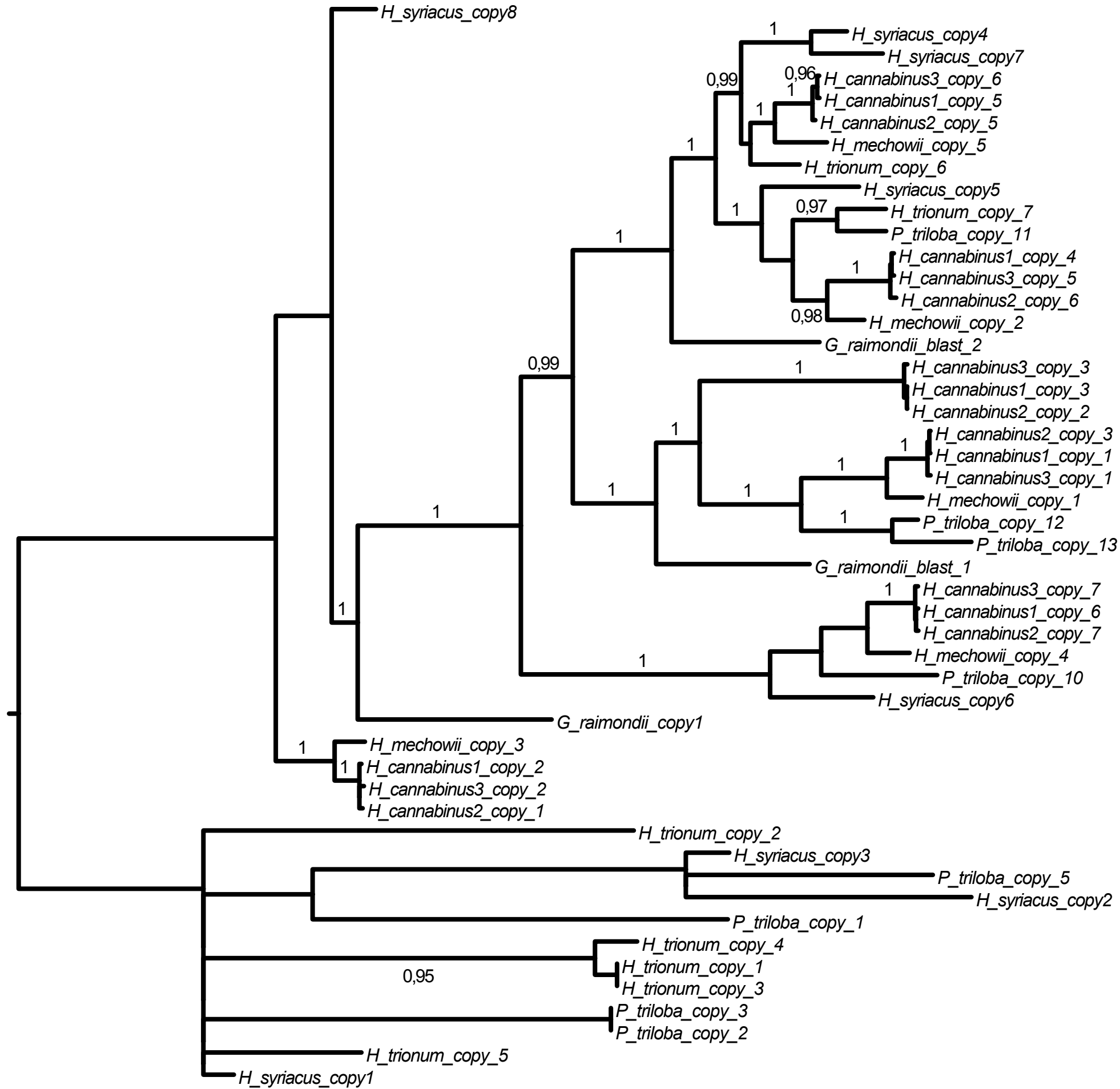

0.03

Supplement: Supplementary file 5 — Additional file 5: Fig. S3. MrBayes trees of multi-copy genes (MSC). [file 12862_2021_1751_MOESM5_ESM.pdf]

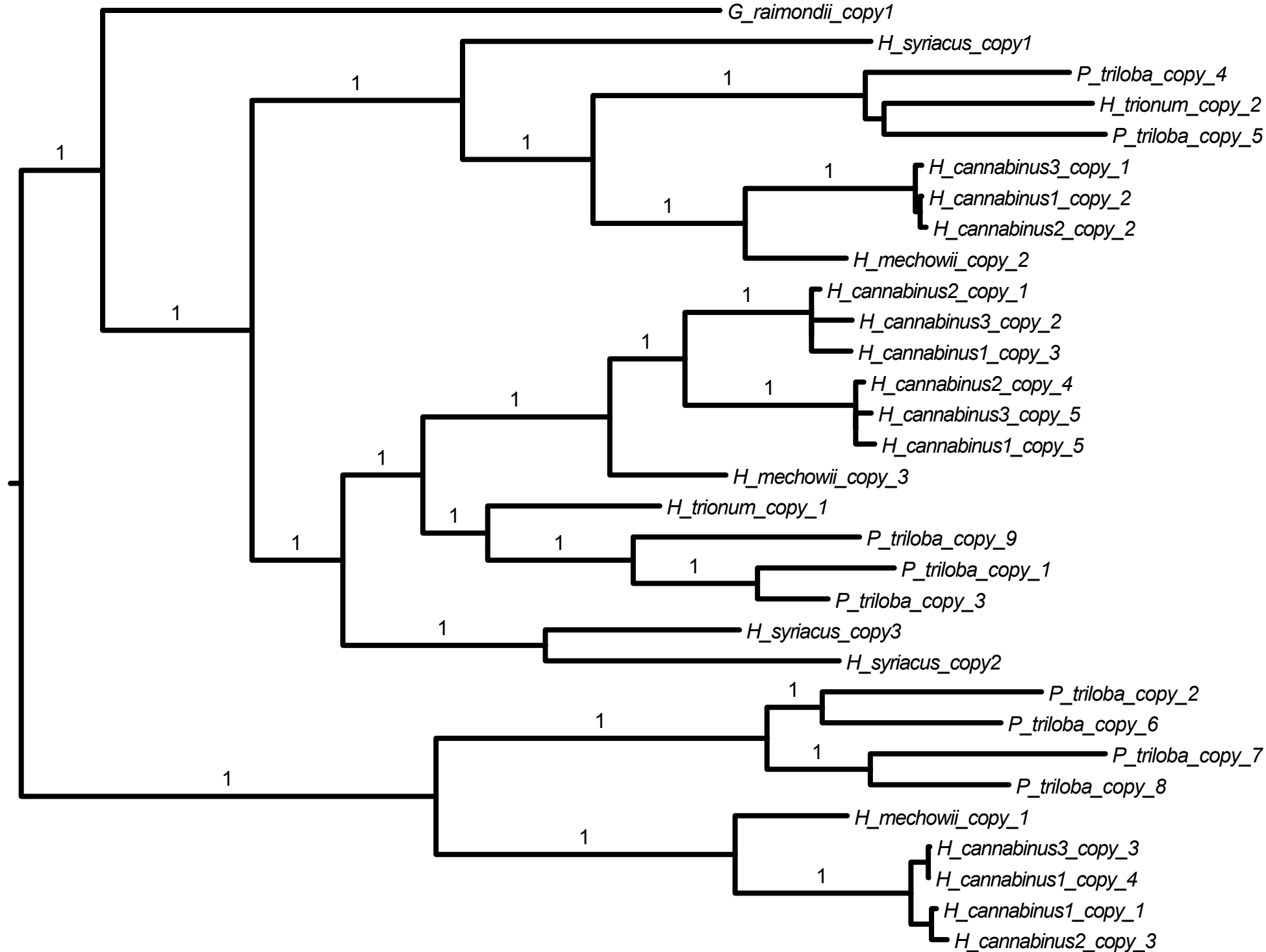

0.02

Supplement: Supplementary file 6 — Additional file 6: Fig. S4. MrBayes trees of multi-copy genes (MSC). [file 12862_2021_1751_MOESM6_ESM.pdf]

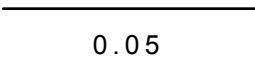

0.05

Supplement: Supplementary file 8 — Additional file 8: Fig. S6. MrBayes trees of multi-copy genes (MSC). [file 12862_2021_1751_MOESM8_ESM.pdf]

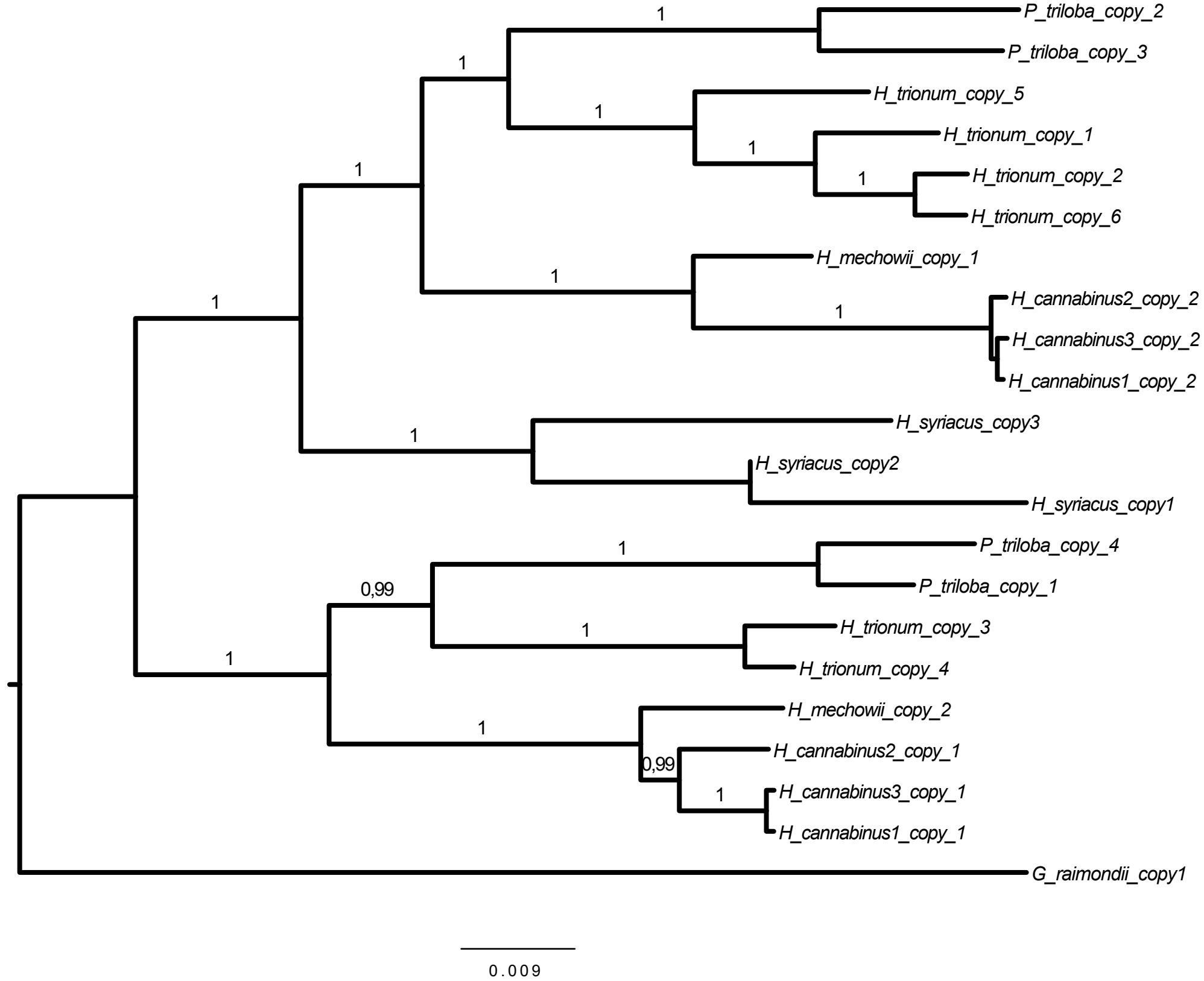

Supplement: Supplementary file 9 — Additional file 9: Fig. S7. MrBayes trees of multi-copy genes (MSC). [file 12862_2021_1751_MOESM9_ESM.pdf]

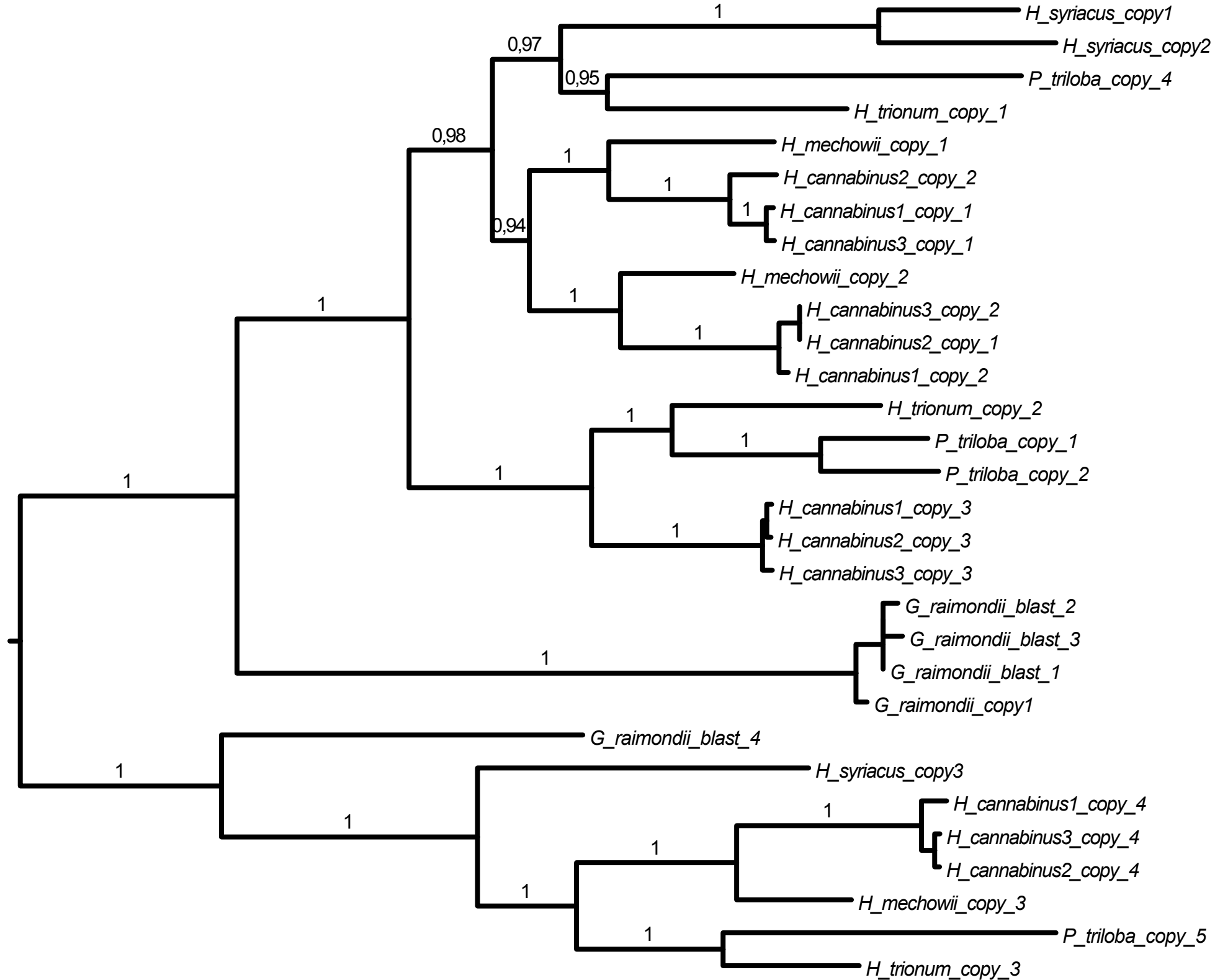

Supplement: Supplementary file 10 — Additional file 10: Fig. S8. MrBayes trees of multi-copy genes (MSC). [file 12862_2021_1751_MOESM10_ESM.pdf]

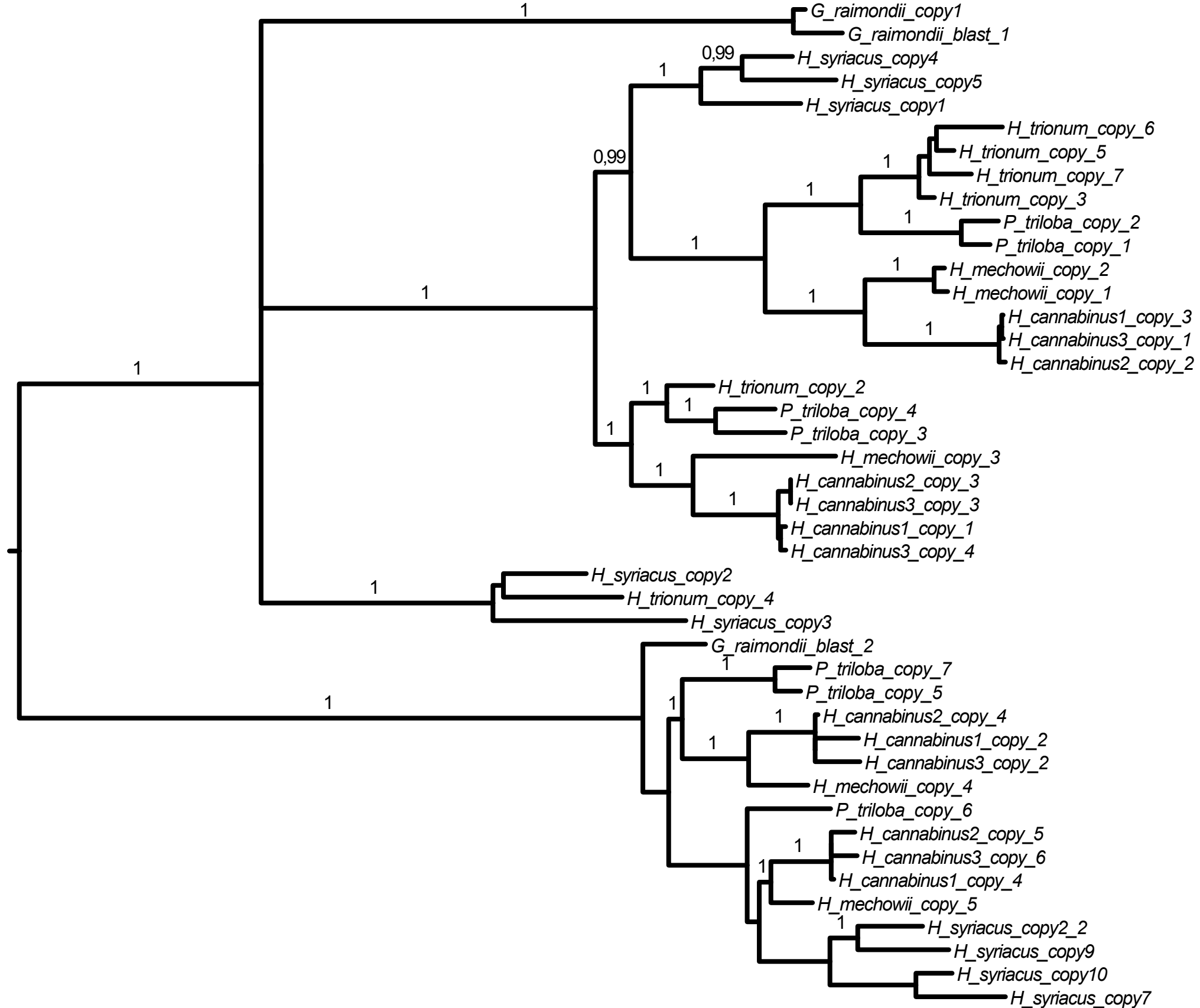

0.04

Supplement: Supplementary file 11 — Additional file 11: Fig. S9. MrBayes trees of multi-copy genes (MSC). [file 12862_2021_1751_MOESM11_ESM.pdf]

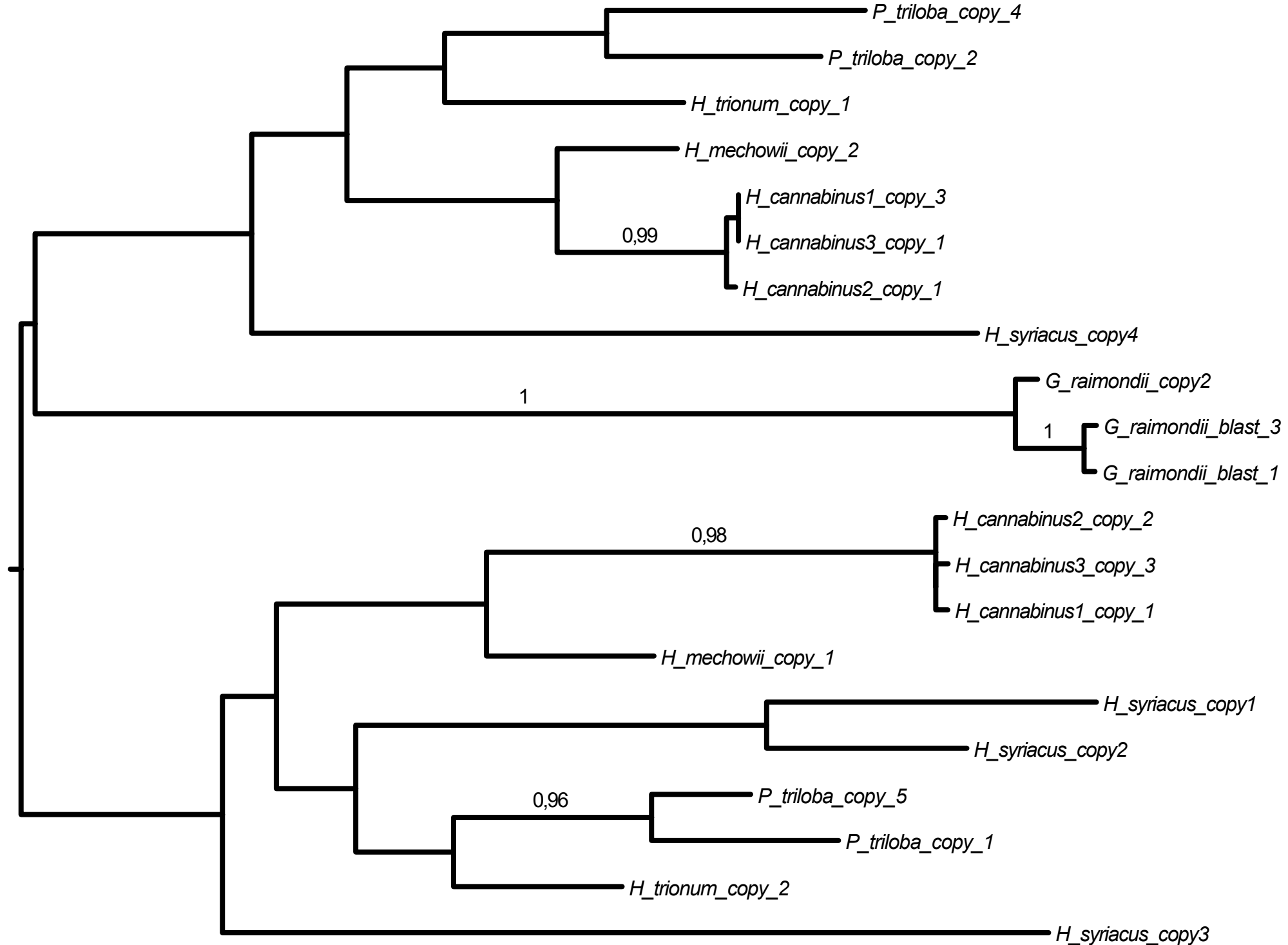

0.02

Supplement: Supplementary file 13 — Additional file 13: Fig. S11. MrBayes trees of multi-copy genes (MSC). [file 12862_2021_1751_MOESM13_ESM.pdf]

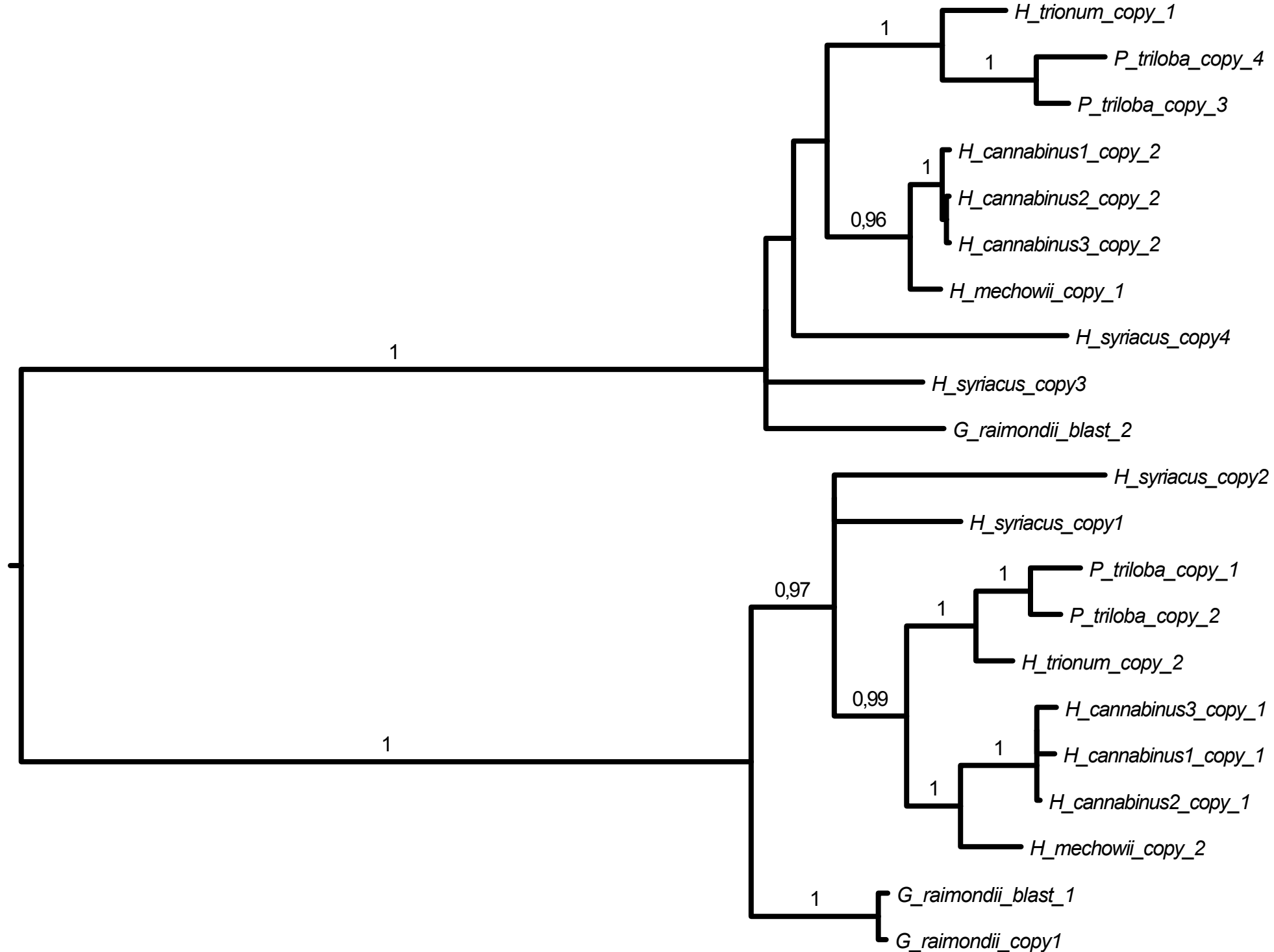

0.03

Supplement: Supplementary file 14 — Additional file 14: Fig. S12. MrBayes trees of multi-copy genes (MSC). [file 12862_2021_1751_MOESM14_ESM.pdf]

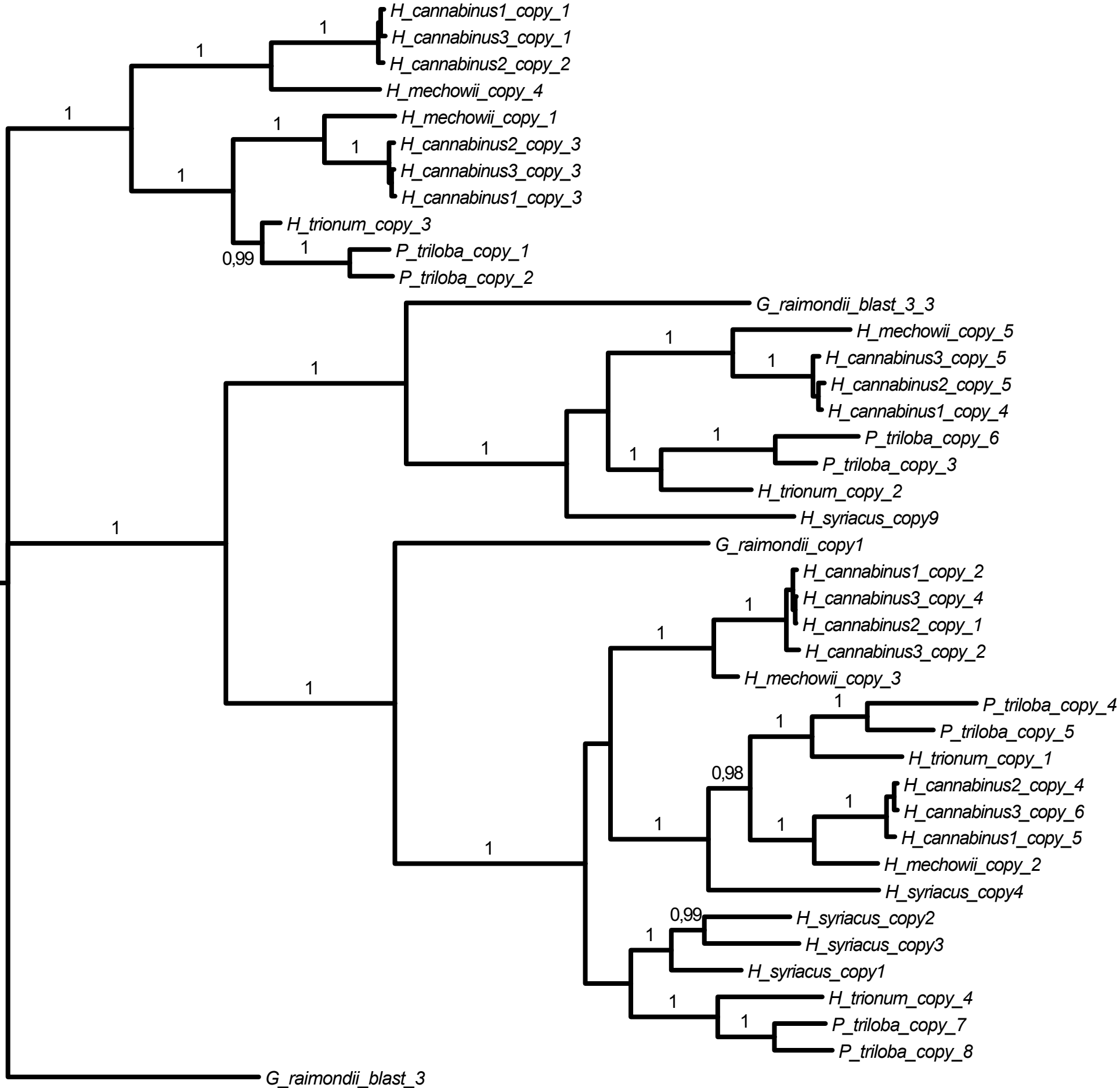

Supplement: Supplementary file 15 — Additional file 15: Fig. S13. MrBayes trees of multi-copy genes (MSC). [file 12862_2021_1751_MOESM15_ESM.pdf]

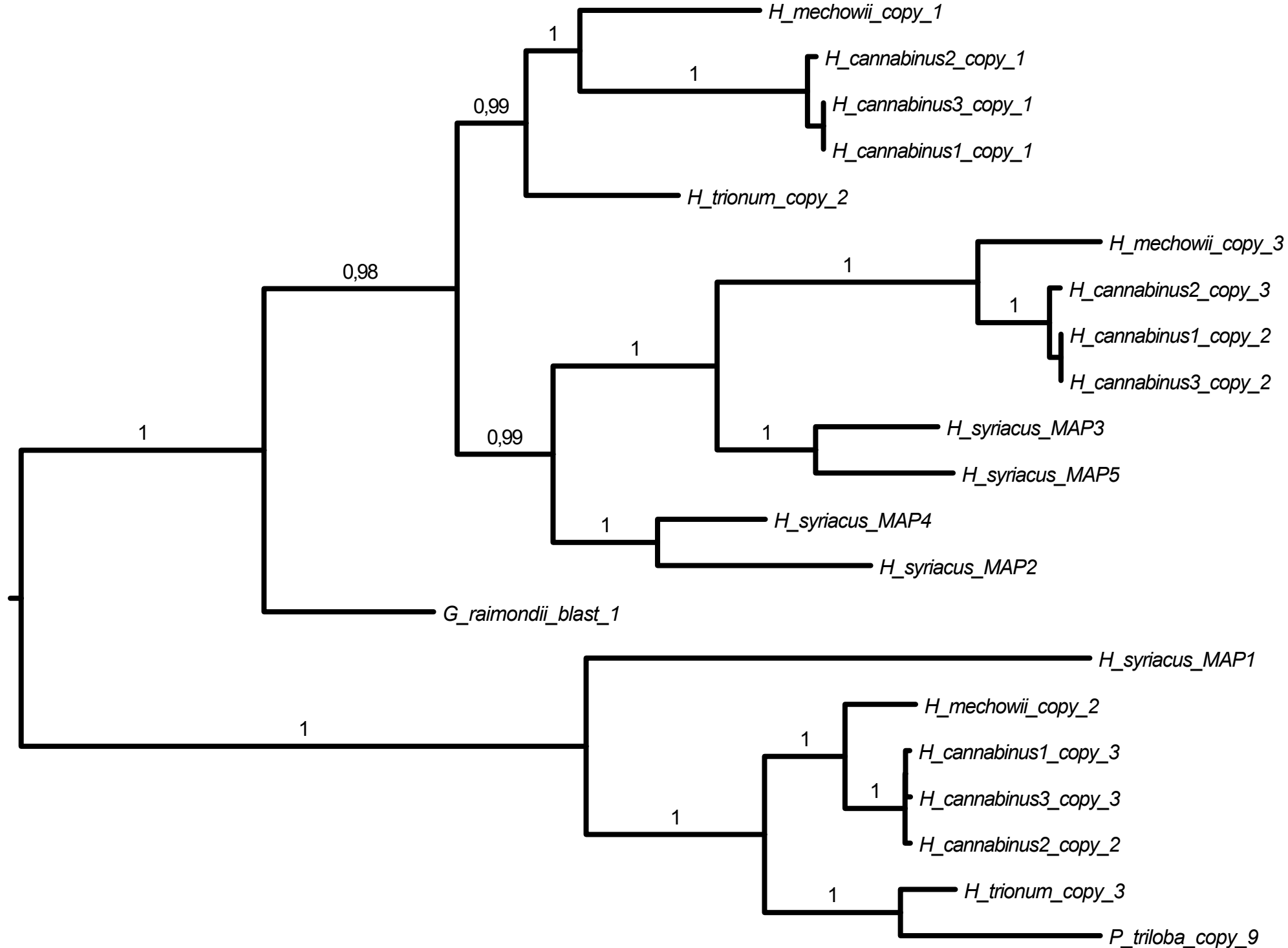

0.02

Supplement: Supplementary file 17 — Additional file 17: Fig. S15. MrBayes trees of multi-copy genes (MSC). [file 12862_2021_1751_MOESM17_ESM.pdf]

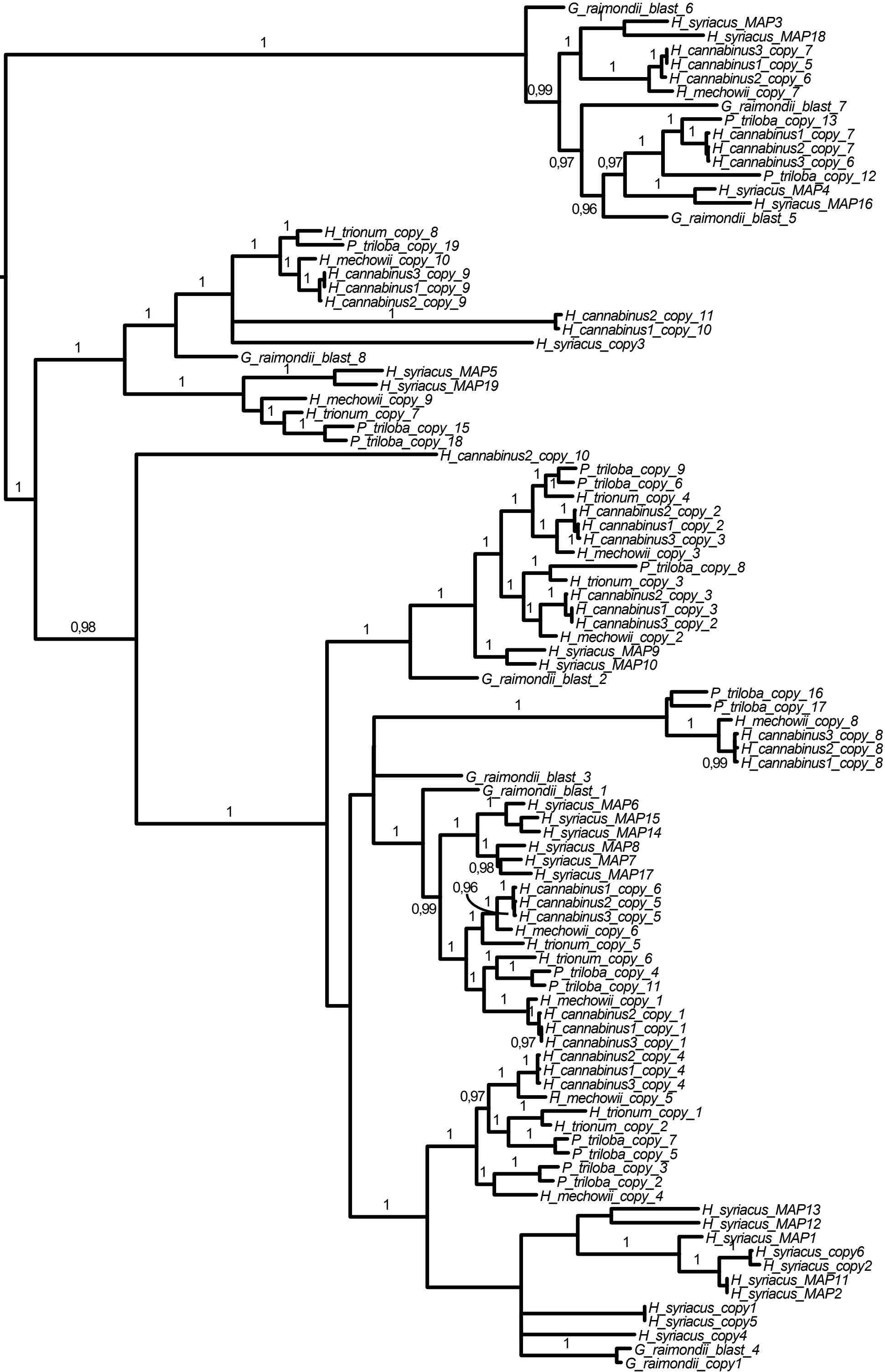

0.05

Supplement: Supplementary file 18 — Additional file 18: Fig. S16. MrBayes trees of multi-copy genes (MSC). [file 12862_2021_1751_MOESM18_ESM.pdf]

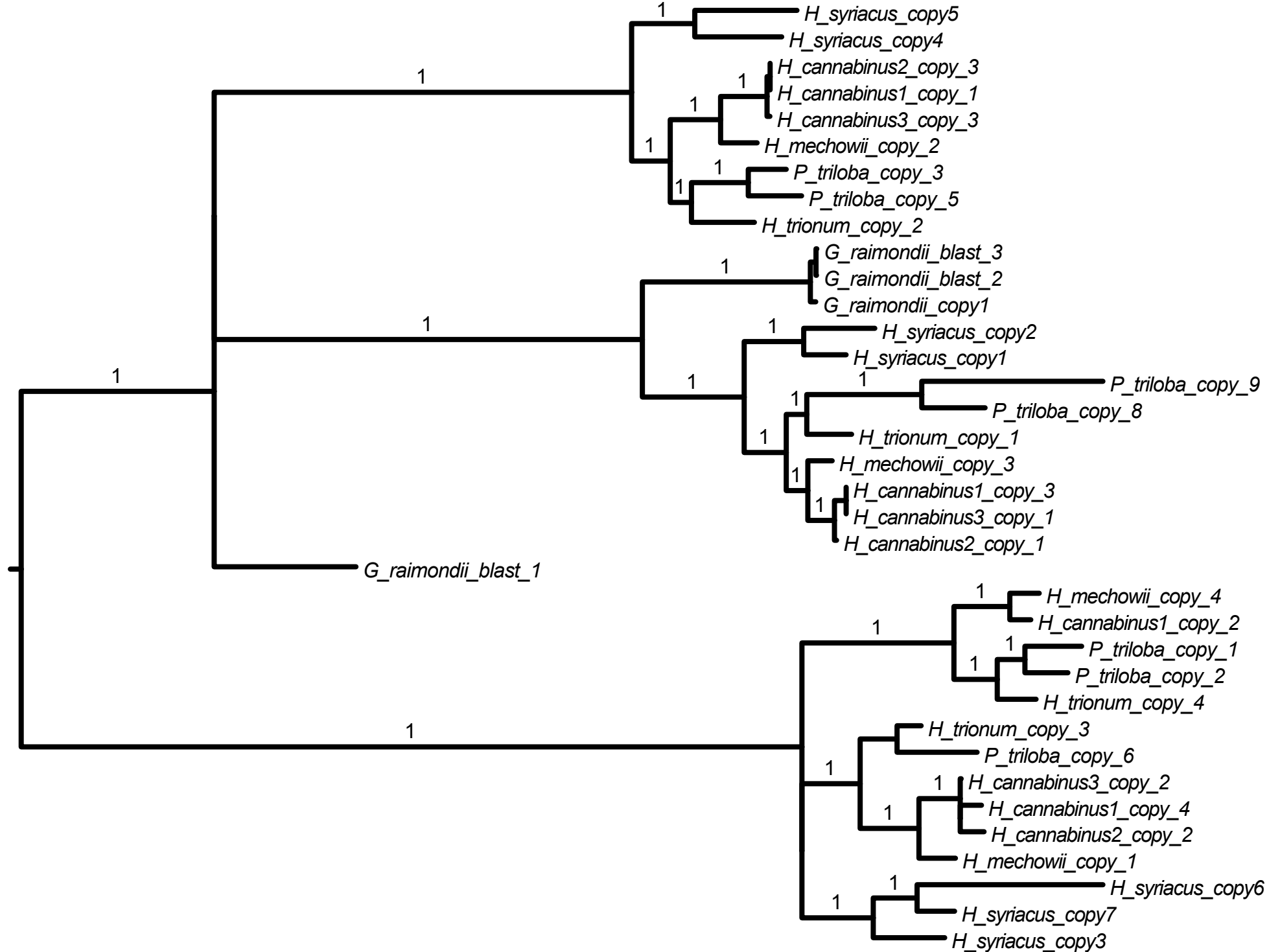

0.04

Supplement: Supplementary file 19 — Additional file 19: Fig. S17. MrBayes trees of multi-copy genes (MSC). [file 12862_2021_1751_MOESM19_ESM.pdf]

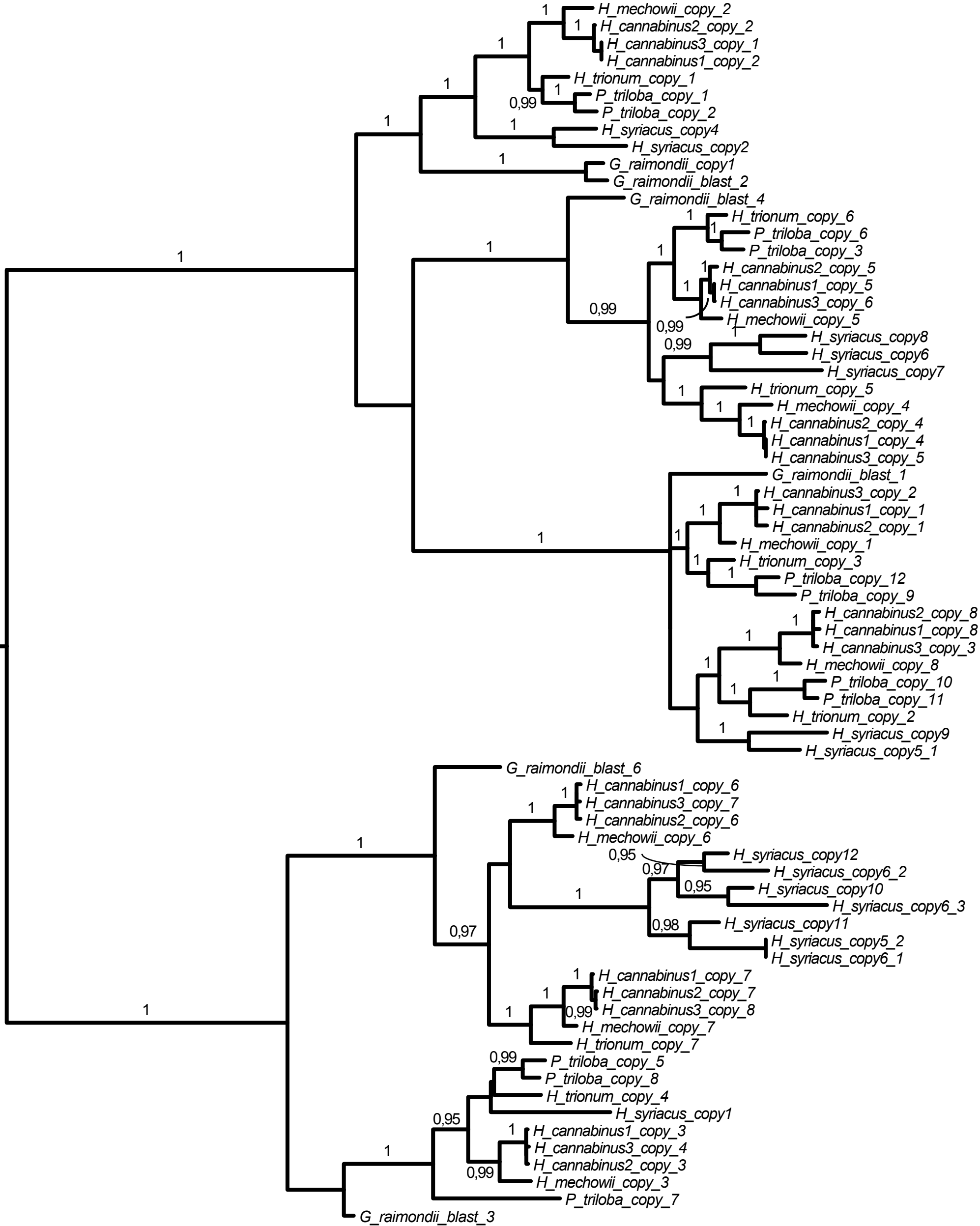

0.05

Supplement: Supplementary file 20 — Additional file 20: Fig. S18. MrBayes trees of multi-copy genes (MSC). [file 12862_2021_1751_MOESM20_ESM.pdf]

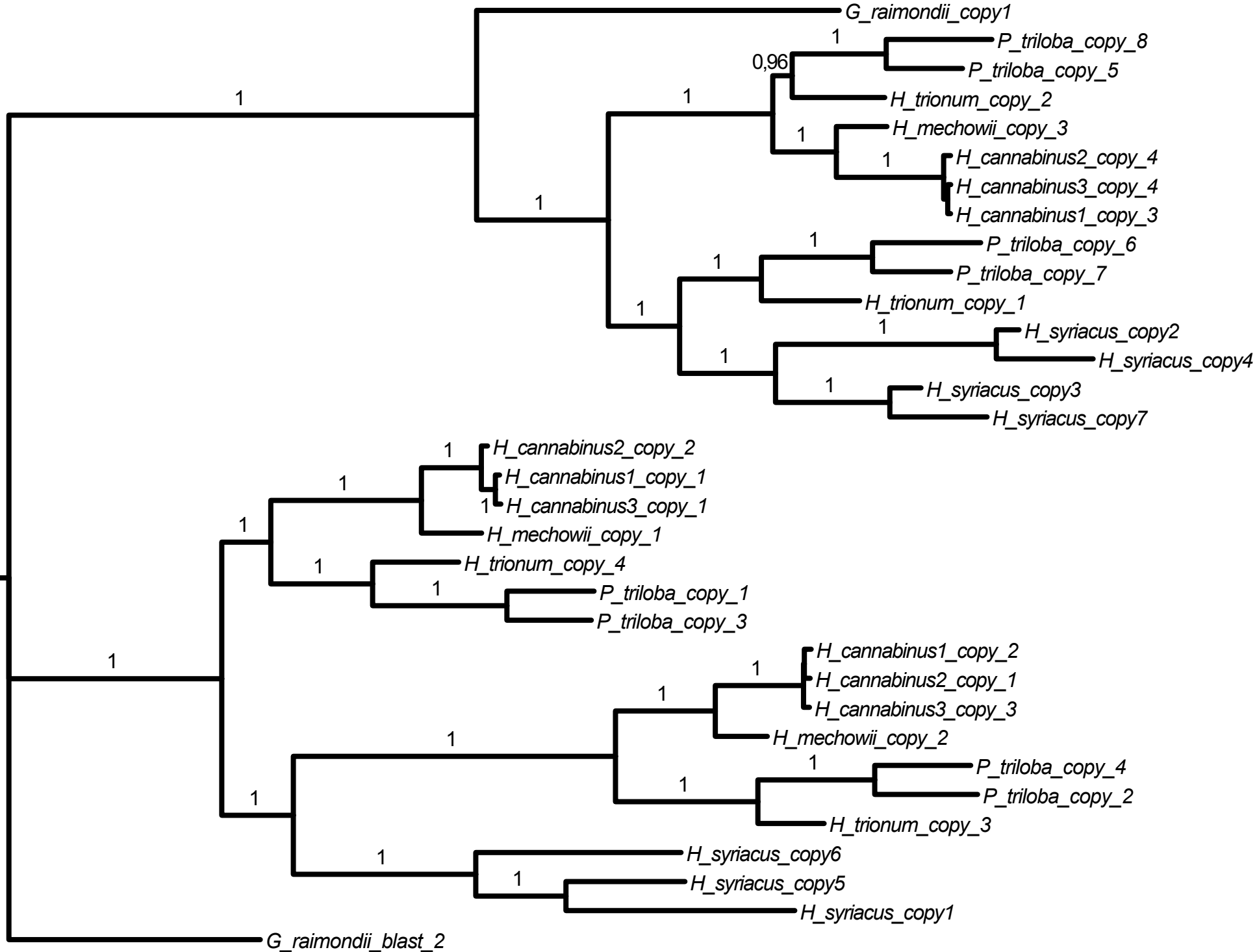

0.02

Supplement: Supplementary file 21 — Additional file 21: Fig. S19. MrBayes trees of multi-copy genes (MSC). [file 12862_2021_1751_MOESM21_ESM.pdf]

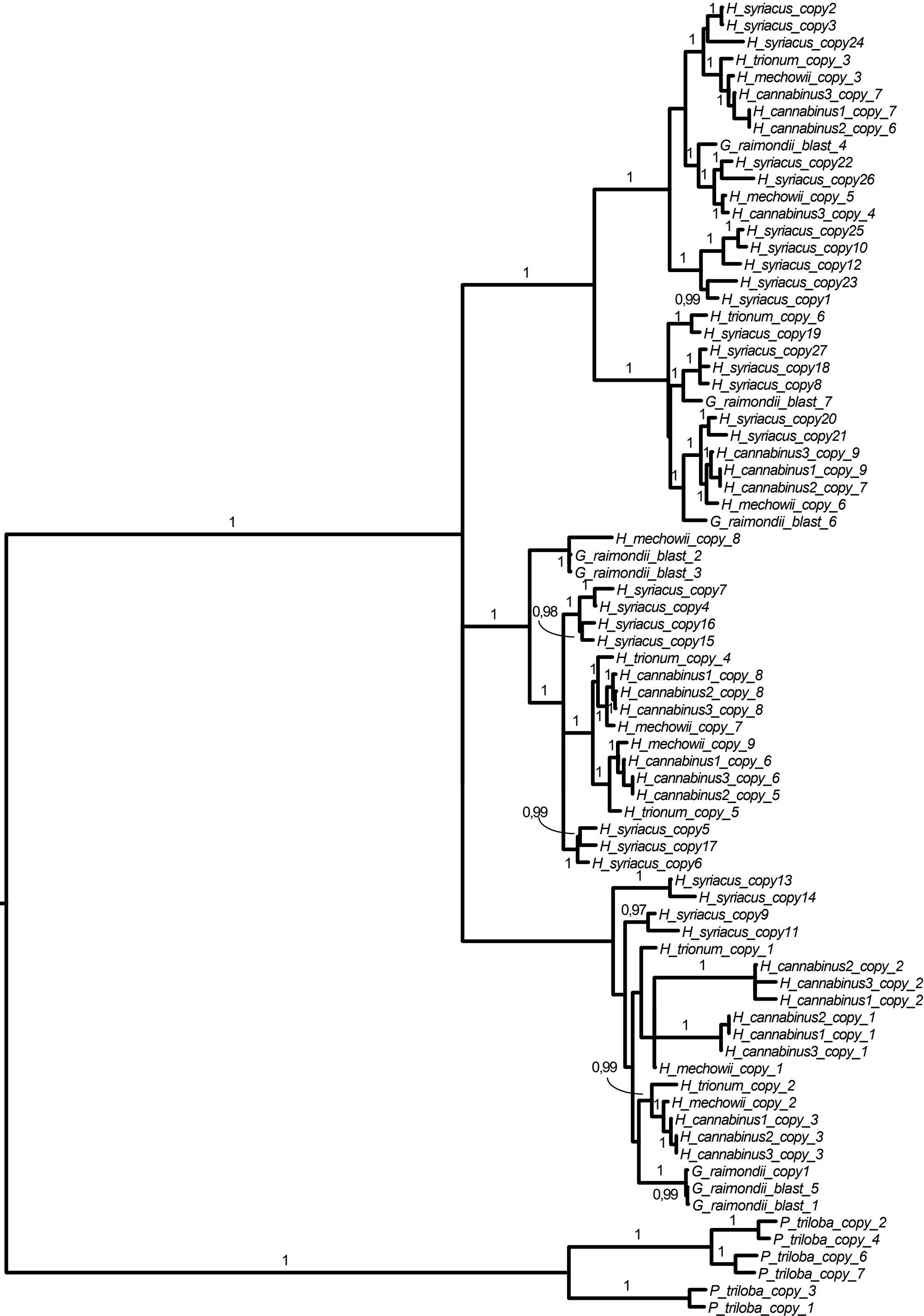

Supplement: Supplementary file 23 — Additional file 23: Fig. S21. MrBayes trees of multi-copy genes (MSC). [file 12862_2021_1751_MOESM23_ESM.pdf]
