# Supplementary material for: Gene count from target sequence capture places three whole genome duplication events in Hibiscus L. (Malvaceae)
Source: BMC Ecol Evol. 2021 Jun 2;21:107. doi: 10.1186/s12862-021-01751-7 (PMC8170824; doi:10.1186/s12862-021-01751-7)
Supplement: Supplementary file 7 — Additional file 7: Fig. S5. MrBayes trees of multi-copy genes (MSC). [file 12862_2021_1751_MOESM7_ESM.pdf]

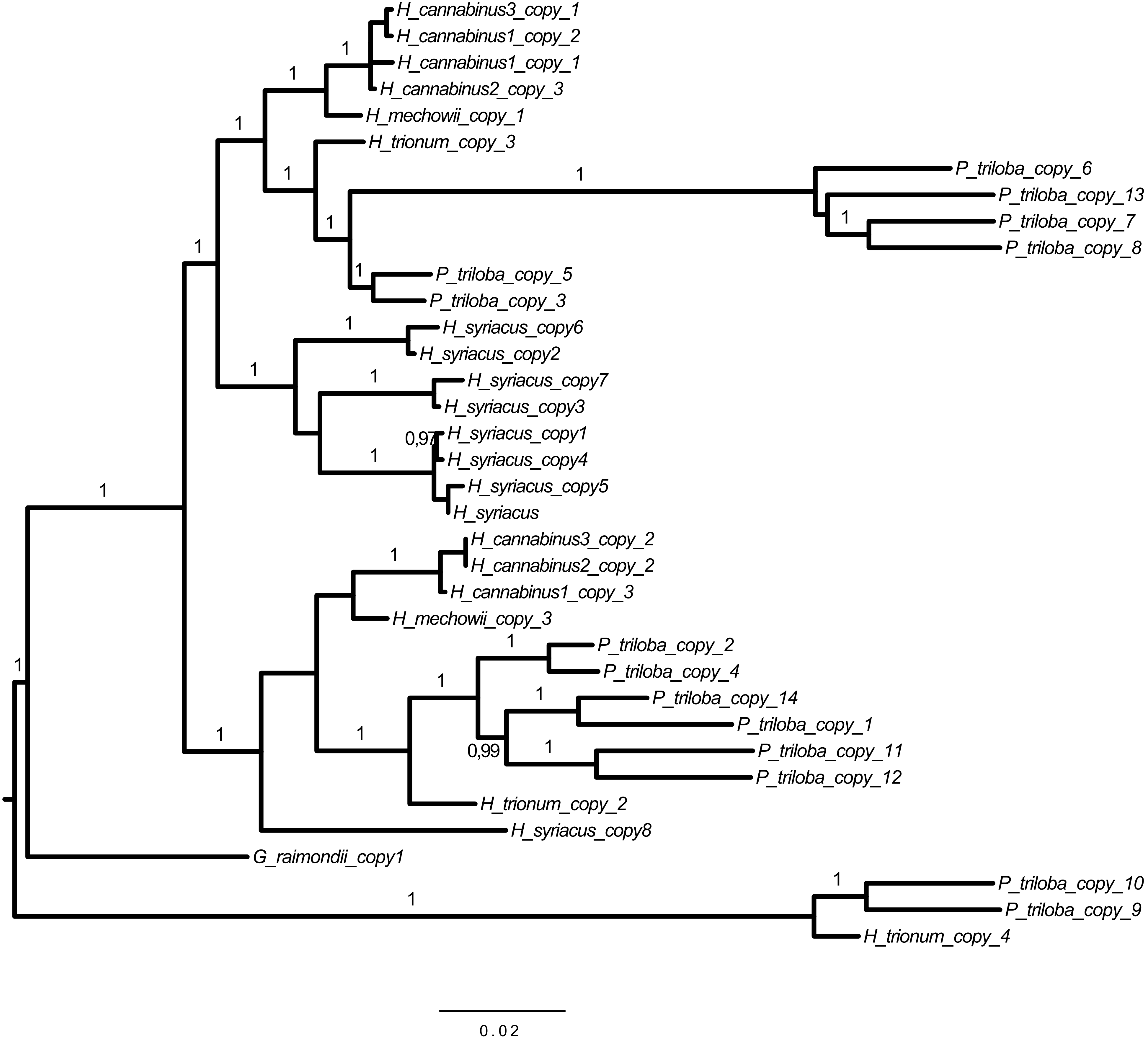
H_cannabinus3_copy_1
H_cannabinus1_copy_2
H_cannabinus1_copy_1
H_cannabinus2_copy_3
H_mechowii_copy_1
H_trionum_copy_3
P_triloba_copy_6
P_triloba_copy_13
P_triloba_copy_7
P_triloba_copy_8
P_triloba_copy_5
P_triloba_copy_3
H_syriacus_copy6
H_syriacus_copy2
H_syriacus_copy7
H_syriacus_copy3
H_syriacus_copy1
H_syriacus_copy4
H_syriacus_copy5
H_syriacus
H_cannabinus3_copy_2
H_cannabinus2_copy_2
H_cannabinus1_copy_3
H_mechowii_copy_3
P_triloba_copy_2
P_triloba_copy_4
P_triloba_copy_14
P_triloba_copy_1
P_triloba_copy_11
P_triloba_copy_12
H_trionum_copy_2
H_syriacus_copy8
G_raimondii_copy1
P_triloba_copy_10
P_triloba_copy_9
H_trionum_copy_4
0,97
0,99
0.02
