# Supplementary material for: Gene count from target sequence capture places three whole genome duplication events in Hibiscus L. (Malvaceae)
Source: BMC Ecol Evol. 2021 Jun 2;21:107. doi: 10.1186/s12862-021-01751-7 (PMC8170824; doi:10.1186/s12862-021-01751-7)
Supplement: Supplementary file 12 — Additional file 12: Fig. S10. MrBayes trees of multi-copy genes (MSC). [file 12862_2021_1751_MOESM12_ESM.pdf]

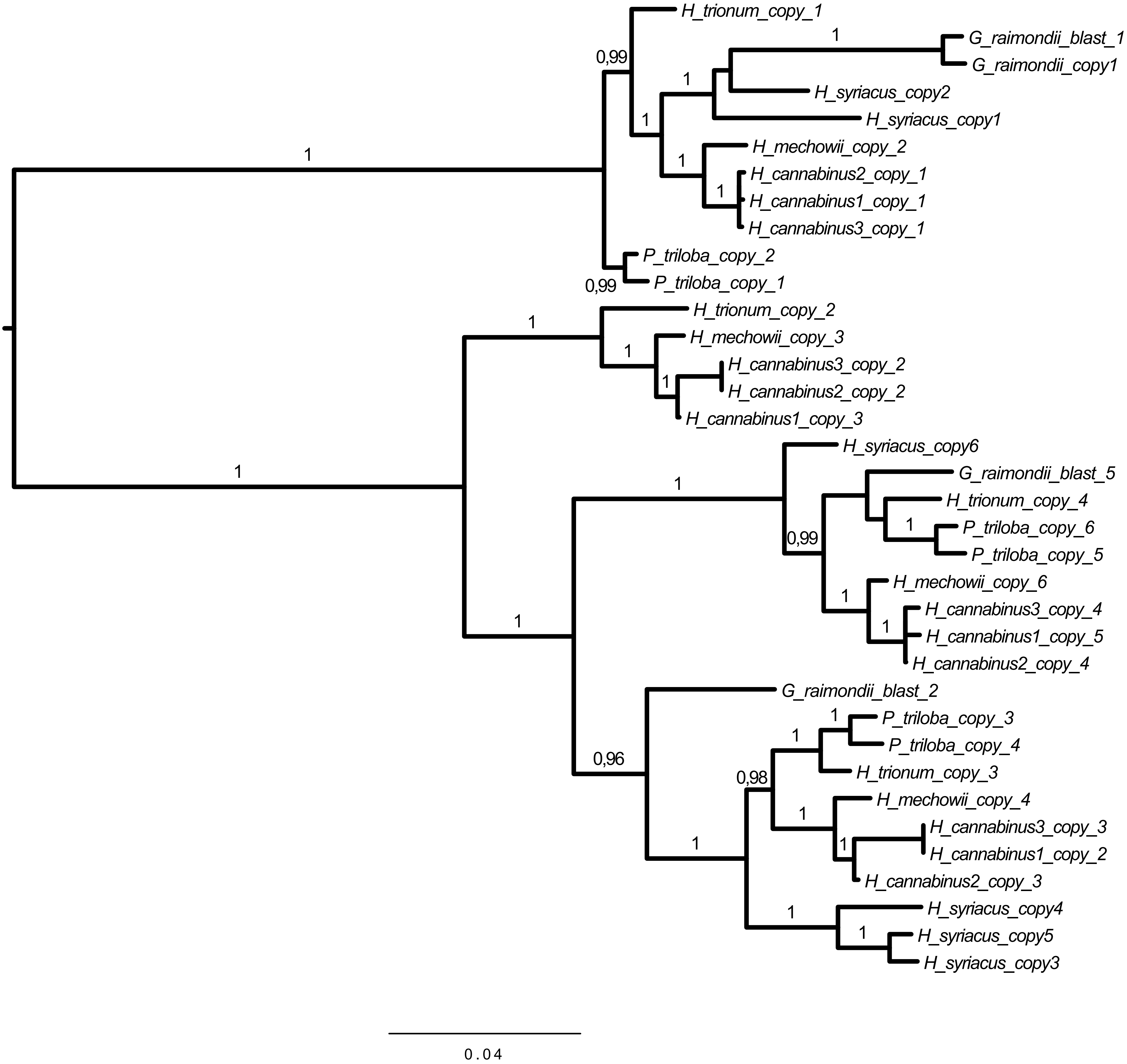
H_trionum_copy_1
G_raimondii_blast_1
G_raimondii_copy1
H_syriacus_copy2
H_syriacus_copy1
H_mechowii_copy_2
H_cannabinus2_copy_1
H_cannabinus1_copy_1
H_cannabinus3_copy_1
P_triloba_copy_2
P_triloba_copy_1
H_trionum_copy_2
H_mechowii_copy_3
H_cannabinus3_copy_2
H_cannabinus2_copy_2
H_cannabinus1_copy_3
H_syriacus_copy6
G_raimondii_blast_5
H_trionum_copy_4
P_triloba_copy_6
P_triloba_copy_5
H_mechowii_copy_6
H_cannabinus3_copy_4
H_cannabinus1_copy_5
H_cannabinus2_copy_4
G_raimondii_blast_2
P_triloba_copy_3
P_triloba_copy_4
H_trionum_copy_3
H_mechowii_copy_4
H_cannabinus3_copy_3
H_cannabinus1_copy_2
H_cannabinus2_copy_3
H_syriacus_copy4
H_syriacus_copy5
H_syriacus_copy3
0,99
0,99
0,99
0,96
0,98
0.04
