# Supplementary material for: Gene count from target sequence capture places three whole genome duplication events in Hibiscus L. (Malvaceae)
Source: BMC Ecol Evol. 2021 Jun 2;21:107. doi: 10.1186/s12862-021-01751-7 (PMC8170824; doi:10.1186/s12862-021-01751-7)
Supplement: Supplementary file 16 — Additional file 16: Fig. S14. MrBayes trees of multi-copy genes (MSC). [file 12862_2021_1751_MOESM16_ESM.pdf]

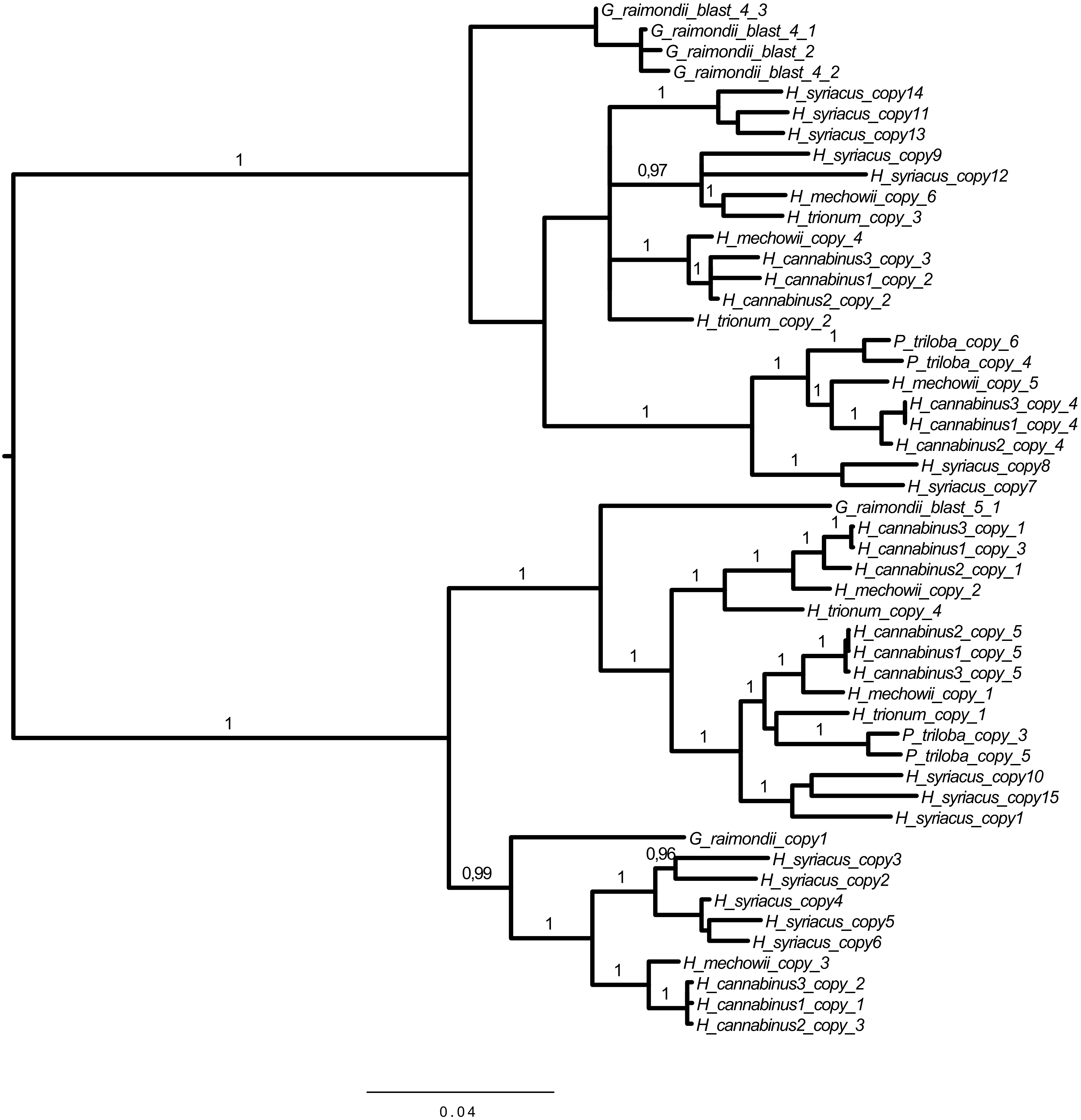
G_raimondii_blast_4_3
G_raimondii_blast_4_1
G_raimondii_blast_2
G_raimondii_blast_4_2
H_syriacus_copy14
H_syriacus_copy11
H_syriacus_copy13
H_syriacus_copy9
H_syriacus_copy12
H_mechowii_copy_6
H_trionum_copy_3
H_mechowii_copy_4
H_cannabinus3_copy_3
H_cannabinus1_copy_2
H_cannabinus2_copy_2
H_trionum_copy_2
P_triloba_copy_6
P_triloba_copy_4
H_mechowii_copy_5
H_cannabinus3_copy_4
H_cannabinus1_copy_4
H_cannabinus2_copy_4
H_syriacus_copy8
H_syriacus_copy7
G_raimondii_blast_5_1
H_cannabinus3_copy_1
H_cannabinus1_copy_3
H_cannabinus2_copy_1
H_mechowii_copy_2
H_trionum_copy_4
H_cannabinus2_copy_5
H_cannabinus1_copy_5
H_cannabinus3_copy_5
H_mechowii_copy_1
H_trionum_copy_1
P_triloba_copy_3
P_triloba_copy_5
H_syriacus_copy10
H_syriacus_copy15
H_syriacus_copy1
G_raimondii_copy1
H_syriacus_copy3
H_syriacus_copy2
H_syriacus_copy4
H_syriacus_copy5
H_syriacus_copy6
H_mechowii_copy_3
H_cannabinus3_copy_2
H_cannabinus1_copy_1
H_cannabinus2_copy_3
0,97
0,99
0,96
0.04
